# gRNA validation for wheat genome editing with the CRISPR-Cas9 system

**DOI:** 10.1186/s12896-019-0565-z

**Published:** 2019-10-30

**Authors:** Taj Arndell, Niharika Sharma, Peter Langridge, Ute Baumann, Nathan S. Watson-Haigh, Ryan Whitford

**Affiliations:** 1 Present address: CSIRO, Agriculture and Food, Canberra, ACT Australia; 2 Present address: New South Wales Department of Primary Industries, Research Excellence, Orange, NSW Australia; 3 School of Agriculture, Food & Wine, The University of Adelaide, Waite Campus, Urrbrae, SA 5064 Australia; 4 Present address: Bioinformatics Hub, School of Biological Sciences, The University of Adelaide, Adelaide, SA 5005 Australia

**Keywords:** Genome editing, CRISPR-Cas9, gRNA, Wheat, *Triticum aestivum*, Protoplasts, EPSPS

## Abstract

**Background:**

The CRISPR-Cas9 system is a powerful and versatile tool for crop genome editing. However, achieving highly efficient and specific editing in polyploid species can be a challenge. The efficiency and specificity of the CRISPR-Cas9 system depends critically on the gRNA used. Here, we assessed the activities and specificities of seven gRNAs targeting 5-enolpyruvylshikimate-3-phosphate synthase (*EPSPS*) in hexaploid wheat protoplasts. EPSPS is the biological target of the widely used herbicide glyphosate.

**Results:**

The seven gRNAs differed substantially in their on-target activities, with mean indel frequencies ranging from 0% to approximately 20%. There was no obvious correlation between experimentally determined and in silico predicted on-target gRNA activity. The presence of a single mismatch within the seed region of the guide sequence greatly reduced but did not abolish gRNA activity, whereas the presence of an additional mismatch, or the absence of a PAM, all but abolished gRNA activity. Large insertions (≥20 bp) of DNA vector-derived sequence were detected at frequencies up to 8.5% of total indels. One of the gRNAs exhibited several properties that make it potentially suitable for the development of non-transgenic glyphosate resistant wheat.

**Conclusions:**

We have established a rapid and reliable method for gRNA validation in hexaploid wheat protoplasts. The method can be used to identify gRNAs that have favourable properties. Our approach is particularly suited to polyploid species, but should be applicable to any plant species amenable to protoplast transformation.

## Background

Genome editing technologies enable the targeted and precise modification of plant genomes via the creation and subsequent repair of site-specific DNA double-strand breaks (DSBs) [1]. Over the last few years, the field of genome editing has been revolutionised by the introduction of the CRISPR (clustered regularly-interspaced short palindromic repeats)-Cas9 (CRISPR associated protein) system [2–7]. This system consists of the Cas9 endonuclease in complex with a small guide RNA (gRNA) that is engineered to target a specific site in the genome. The target site is defined by a 20 nucleotide guide sequence at the 5′ end of the gRNA, making programming of the system relatively straightforward. For the system to function, the target site must be located immediately 5′ to a protospacer adjacent motif (PAM) whose canonical form is 5′-NGG-3′ (for SpCas9 from *Streptococcus pyogenes*). The PAM is on the strand opposite to the strand bound by the gRNA. Site-specific DSBs generated by Cas9 are typically repaired through either of two competing pathways: non-homologous end joining (NHEJ) or homology directed repair (HDR). NHEJ, which is the predominant repair pathway in somatic plant cells [8], is error-prone and often produces small insertions/deletions (indels) that result in gene knockout (e.g. through frame shift or the creation of a premature stop codon) [9–12]. Alternatively, if an exogenous DNA donor template with homologous ends is delivered to the cell, then precise modifications (sequence insertion or replacement) can be made through HDR [9, 13–18]. If the homologous ends of the donor template are short (5–25 bp), then the DSB may be repaired through microhomology-mediated end joining (MMEJ) [19]. Due to its simplicity, flexibility, and high specificity, the CRISPR-Cas9 system has been and continues to be rapidly adopted by the plant research community for basic research and crop improvement.

Although the CRISPR-Cas9 system has been successfully applied to many model and crop plants, editing efficiencies have varied greatly and in many cases have been lower than one would desire. In particular, gene knockout via NHEJ tends to be relatively inefficient in polyploidy species due to genetic redundancy [20–22]. Furthermore, the inherent low frequency of HDR remains a major challenge in plant genome editing [23]. In addition, despite the high specificity of the CRISPR-Cas9 system, off-target mutations can occur at sites that have sequence similarity to the target site [24–26], especially when there are no mismatches in the PAM-proximal 8–12 nucleotide ‘seed region’ of the guide sequence [27–30]. Such off-target sites may be present in non-target genes or non-target alleles. Off-target mutations are undesirable as they may confound results and/or produce impaired phenotypes, in which case they must be removed by backcrossing. Consequently, much effort has been directed toward improving the efficiency and specificity of the CRISPR-Cas9 system in plants and other organisms.

It is well-established that the efficiency and specificity of the CRISPR-Cas9 system depends critically on target site selection, as well as certain sequence features of the gRNA. Thus, an effective strategy for achieving efficient and specific editing is to use gRNAs that exhibit high activity and specificity. A number of bioinformatics tools have been developed for the in silico prediction of on-target and/or off-target gRNA activity [31–36]. Some of these tools can provide reliable predictions for potential off-target sites in a limited number of species, and predictions for on-target gRNA activity can reduce the time spent on gRNA screening [37]. However, the predictions are not always accurate, and the development and independent validation of these tools has been based on data obtained from non-plant species. Therefore, it is prudent to carry out experimental validation of gRNAs prior to commencing plant transformation experiments that require substantial investment of time and resources.

Here, we propose and test a strategy for assessing gRNA activity and specificity, using seven gRNAs targeting 5-enolpyruvylshikimate-3-phosphate synthase (*EPSPS*) in hexaploid wheat (*Triticum aestivum*). *EPSPS* is an ideal target for editing via HDR, as several well-characterised amino acid substitutions in this gene are known to confer resistance to the widely used herbicide glyphosate [38]. Furthermore, in wheat there is an opportunity to take advantage of its hexaploid nature by performing homoeoallele-specific editing, thereby potentially avoiding the severe yield penalty associated with homozygous amino acid substitutions and loss-of-function mutations in *EPSPS* [39–41]. Therefore, one of our aims was to identify a highly active and homoeoallele-specific gRNA targeting *ESPSP*. Three of our gRNAs were designed to be homoeoallele-specific based on the presence of single nucleotide polymorphisms (SNPs) between the three homoeologous copies of *EPSPS*. This allowed us to determine the frequency of off-target mutagenesis. The seven gRNAs were rigorously evaluated through transient expression of CRISPR-Cas9 reagents in wheat mesophyll protoplasts, followed by TIDE (Tracking of Indels by DEcomposition) [42] analysis of Sanger sequence reads, and CRISPResso [43] analysis of amplicon reads. On-target activities varied substantially between gRNAs, and there was no obvious correlation between experimentally determined and in silico predicted on-target gRNA activity. Off-target mutations in homoeoalleles of *EPSPS* were detected at low frequencies, whereas large insertions (≥20 bp) of DNA vector-derived sequence were detected at surprisingly high frequencies. One of the gRNAs exhibited several properties that make it potentially suitable for the development of non-transgenic glyphosate resistant wheat.

## Results

### Cloning and sequencing of *EPSPS* in wheat cv. Fielder

**Fig. 1:**
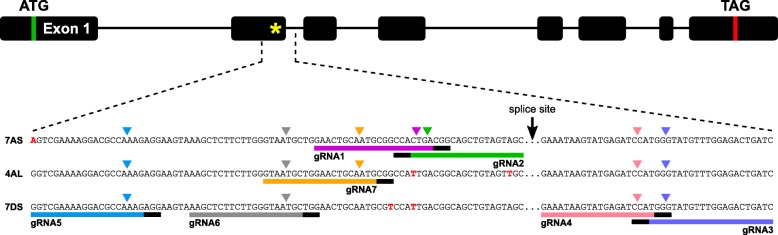
Target sites for seven gRNAs targeting *EPSPS*. The gene structure of *EPSPS* is shown, annotated with the universal mutation hotspot for glyphosate resistance (yellow asterisk in exon 2). The detail underneath shows partial, abbreviated sequences for the three homoeoalleles of *EPSPS* on chromosomes 7AS, 4AL and 7DS. Target sites are indicated by coloured bars. PAM sites (5′-NGG-3′) are indicated by black bars at the ends of the coloured bars. Downward-pointing arrow heads indicate the position of the canonical cut site and predicted specificity based on the number and distribution of homoeologous SNPs at the corresponding target site/PAM

A vast number of intervarietal SNPs are known to exist in hexaploid wheat [44]. Therefore, for the purpose of designing effective gRNAs, we first obtained sequence information for the three homoeoalleles of *EPSPS* in our target wheat cv. Fielder. For each homoeoallele, two independent partial genomic clones of *EPSPS* were Sanger sequenced. In each case, the sequences of the two independent clones were identical. Thus, the following consensus sequences, covering a region from the 5′ end of intron 1 to the middle of exon 5, were obtained: TaEPSPS-FL-7AS [GenBank MG460802], TaEPSPS-FL-4AL [GenBank MG460801], and TaEPSPS-FL-7DS [GenBank MG460803]. These consensus sequences mapped unambiguously to chromosomes 7AS, 4AL (translocated from 7BS) and 7DS, as expected [45]. We identified three synonymous, homoeologous SNPs located at the 3′ end of exon 2, in close proximity to a universal mutation hotspot for resistance to glyphosate [46] (Fig. 1). We exploited these SNPs for the design of homoeoallele-specific gRNAs (see below).

### Protoplast transformation

We designed seven gRNAs targeting a region of *EPSPS* that contains the universal mutation hotspot for resistance to glyphosate (Fig. 1). gRNA1, gRNA2 and gRNA7 were designed to target only one or two of the three *EPSPS* homoeoalleles, whereas the other four gRNAs were designed to target all three homoeoalleles. We transiently co-expressed Cas9 and each gRNA in wheat mesophyll protoplasts. To gauge transient transformation efficiencies, we used a positive control in which YFP was substituted for the gRNA. The proportion of fluorescent (YFP expressing) protoplasts in the positive control ranged from 64 to 72% (mean = 68%) (Additional file 1). We found that the key to achieving high transient transformation efficiencies was to dilute the protoplasts to a concentration of 3.0 × 10^5^ cells/mL (instead of 2.5 × 10^6^ cells/mL as described in another protocol [47]) prior to transformation, and to avoid extended incubation of DNA with protoplasts prior to adding PEG (Additional file 2).

### Assessment of gRNA activity and specificity via TIDE analysis of Sanger sequence traces

**Fig. 2:**
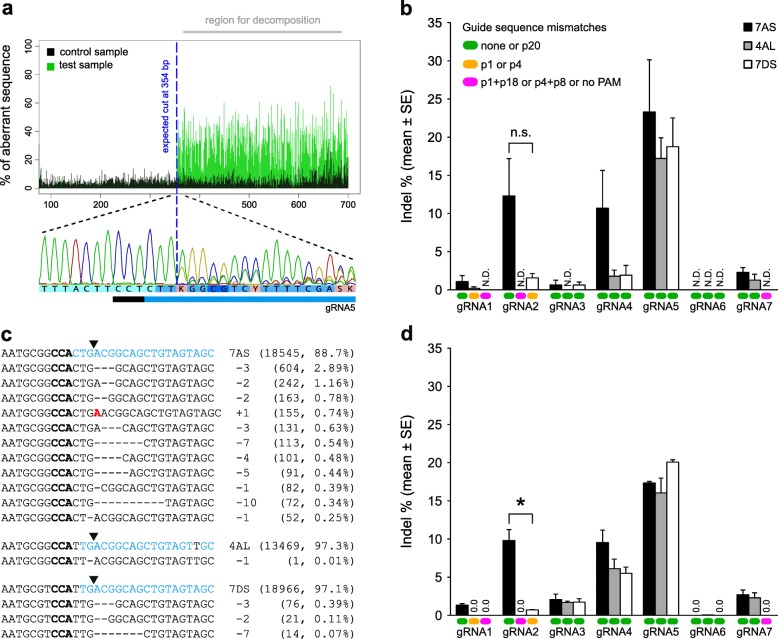
Mutation detection and summary of editing efficiencies for seven gRNAs targeting *EPSPS* on chromosomes 7AS, 4AL and 7DS. **a** TIDE detection of mixed peaks in the reverse Sanger sequence read for gRNA5 on chromosome 7AS (replicate 1). **b** Summary of TIDE results. N.D., not detected. n.s., not statistically significant. Error bars represent the standard error of the mean (*n* = 3). **c** Alignment of representative mutant amplicon reads for gRNA2 on chromosomes 7AS, 4AL and 7DS (replicate 1). Bold black text, PAM; blue text, complementary to gRNA2 guide sequence; red text, inserted nucleotide. Downward-pointing arrow heads indicate the position of the canonical cut site. The number of reads and percent of total reads is shown in brackets. **d** Summary of CRISPResso results. Error bars represent the standard error of the mean (*n* = 3). * statistically significant (*p* < 0.05) based on a two-sample *t*-test assuming unequal variances. The keys in **b** also apply to **d**. In the key for guide sequence mismatches, p20 means position 20 in the guide sequence, etc.

We obtained high-quality forward and reverse Sanger sequence reads (Additional files 3-10) of homoeoallele-specific amplicons (Additional file 11) derived from protoplasts treated with each of the seven *EPSPS*-specific gRNAs and one non-targeting (random guide sequence) negative control gRNA. As expected, some of the sequence traces for samples treated with *EPSPS*-specific gRNAs contained mixed peaks downstream of the predicted cut site, and these mixed peaks were detected by TIDE as an increase in the percent of aberrant sequence relative to the negative control (Fig. 2a). There was a strong correlation between the indel spectra/frequencies calculated by TIDE for forward and reverse sequence traces, with each decomposition result having a high goodness of fit (R^2^ ≥ 0.93) (Additional file 12). Individual indels (significant at *p* < 0.001) were detected at frequencies down to approximately 1% (e.g. gRNA2, Rep 3, 7DS in Additional file 12). The mean frequency of significant indels ranged from 0.0–23.3% depending on the gRNA and homoeoallele (Fig. 2b). gRNA5 was the most highly active gRNA on all three homoeoalleles, and the presence of a single mismatch at the PAM-distal end of the guide sequence (position 20) on 7AS did not reduce the activity of gRNA5 (Fig. 2b). gRNA2 was moderately active on 7AS, and off-target indels were also detected at low frequency on 7DS in the presence of a single mismatch at the PAM-proximal end of the guide sequence (position 1) (Fig. 2b). gRNA4 was also moderately active on 7AS, but the frequency of indels appeared to be lower on 4AL and 7DS, even though no mismatches were present (Fig. 2b). All other gRNAs exhibited low or no activity (Fig. 2b).

### Assessment of gRNA activity and specificity via CRISPResso analysis of amplicon reads

As an alternative method for detecting indels produced through NHEJ, and to crosscheck the TIDE results, we subjected all of the samples to amplicon deep sequencing. Due to high sequence similarity between amplicon reads derived from the three homoeoalleles of *EPSPS*, CRISPResso was unable to accurately map the reads to their respective reference amplicon sequences. Therefore, we used pre-mapped amplicon reads [NCBI BioProject PRJNA420019] as input for the CRISPResso analyses. In CRISPResso, the total number of aligned (analysed) amplicon reads in each subanalysis (i.e. a replicate for a gRNA on a homoeoallele) ranged from 7067 to 35,668 (mean = 18,110). In general, the CRISPResso results (Fig. 2c and d, Additional files 13 and 14) were in agreement with the TIDE results, but there was less variation between replicates in the CRISPResso results, as indicated by smaller standard errors (Fig. 2d). Notably, in the CRISPResso results, the activity of gRNA2 on 7DS (off-target) was only 7% of that on 7AS (on-target), and the difference was statistically significant (*p* < 0.05) (Fig. 2d). Also, the activity of gRNA4 was more consistent across homoeoalleles (Fig. 2d). The frequency of indels in the negative control was ≤0.1% (mean = 0.005%).

Based on both TIDE and CRISPResso derived data, we determined that gRNA2 is likely to be the most effective gRNA for generating stable homoeoallele-specific (chromosome 7AS) edits in *EPSPS*.

### In silico prediction of on-target gRNA activity

The seven gRNAs differed substantially in their in silico predicted on-target activity (Table 1). sgRNA Designer scores [34] ranged from 0.47–0.85 (potential range = 0–1). WU-CRISPR scores [32] ranged from < 50–85 (potential range = 0–100; scores < 50 are not output). There was some disagreement between the sgRNA Designer and WU-CRISPR scores. In particular, the top-scoring gRNA in sgRNA Designer (gRNA7) had a WU-CRISPR score of < 50. There did not appear to be any obvious correlation between experimentally determined and in silico predicted on-target gRNA activity (Table 1).

**Table 1:** Experimentally determined versus in silico predicted on-target gRNA activity

	Target homoeoalleles	Indel frequency (%)^a^	sgRNA Designer score^b^	WU-CRISPR score^c^
gRNA1	7AS	1.3	0.59	58
gRNA2	7AS	9.8	0.58	60
gRNA3	7AS, 4AL, 7DS	1.8	0.64	82
gRNA4	7AS, 4AL, 7DS	7.0	0.52	< 50
gRNA5	7AS, 4AL, 7DS	17.8	0.68	85
gRNA6	7AS, 4AL, 7DS	0.0	0.47	< 50
gRNA7	7AS, 4AL	2.5	0.85	< 50

^a^Experimentally determined on-target gRNA activity, expressed as the mean proportion of edited amplicon reads derived from target homoeoalleles for three replicates. ^b^Potential range = 0–1 (1 is highest predicted gRNA activity); **c** potential range = 0–100 (100 is highest predicted gRNA activity, scores < 50 are not output)

### Analysis of large insertions

**Fig. 3:**
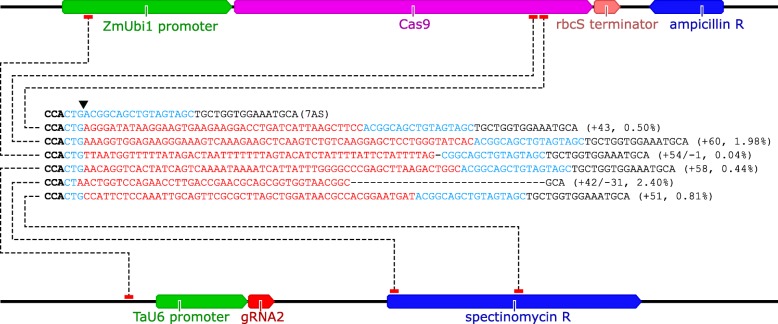
Representative examples of large insertions (≥20 bp) for gRNA2 (replicate 3). Schematics of pUbi-Cas9-rbcS (top) and pCR8-U6-gRNA2 (bottom) are shown, annotated with links (dotted lines) indicating from where the inserted sequences originate. Bold black text, PAM; blue text, complimentary to gRNA2 guide sequence; red text, inserted sequence. The downward-pointing indicates the position of the canonical cut site. The type/size of the mutation is given in brackets, together with the allele frequency as a percent of edited amplicon reads

To detect large insertions (≥20 bp), we used unmapped amplicon reads [NCBI BioProject PRJNA420019] as input for a separate CRISPResso analysis. Large insertions were detected in the majority of samples. The third replicate of gRNA2 had the highest frequency of large insertions (8.5% of edited reads), all of which showed 100% sequence identity to components of the DNA vectors used for protoplast transformation (Fig. 3, Additional file 15). Similar frequencies of large insertions were observed for the third replicate of gRNA5 (5.8% of edited reads), and no large insertions were detected in the negative controls (Additional file 15). CRISPResso failed to correctly predict the size of the insertion when the insertion was accompanied by a deletion. For example, the + 42/− 31 and + 54/− 1 mutations (Fig. 3) were listed as + 21 and + 53 insertions respectively, in the CRISPResso allele frequency table (Additional file 15).

## Discussion

We assessed the activity and specificity of seven gRNAs targeting *EPSPS* in wheat mesophyll protoplasts. Certain amino acid substitutions in EPSPS are known to confer resistance to the herbicide glyphosate [38], and therefore *EPSPS* is an ideal candidate for editing via HDR given that glyphosate resistance is a robust selectable marker in wheat tissue culture and during plant growth [48, 49]. Furthermore, in wheat it may be possible to avoid the severe yield penalty associated with homozygous amino acid substitutions and loss-of-function mutations in *EPSPS* [39–41], by performing homoeoallele-specific editing. However, given the inherent low frequency of HDR, and the potential for simultaneous editing of all three homoeoalleles, this application requires the use of a highly active and (ideally) homoeoallele-specific gRNA. With this in mind, our aim was to identify such a gRNA, and in doing so build on previous work [47] to develop an improved method for validating gRNAs in wheat and other polyploid species.

The wide range of on-target activities observed for the tested gRNAs in this study is consistent with previous reports of CRISPR-based genome editing using hexaploid wheat mesophyll protoplasts. A gRNA targeting *TaMLO-A1* caused indels at a frequency of 29% based on the PCR-restriction enzyme (PCR-RE) assay [9]. In a separate study, the same gRNA caused indels at a frequency of 36% based on a homoeoallele-specific T7E1 assay [50]. Similar editing efficiencies were attained with gRNAs targeting *TaGW2* and *TaGASR7* [51]. gRNAs targeting *TaDEP1*, *TaNAC2*, *TaPIN1* and *TaLOX2* were also evaluated by the PCR-RE assay [20], but the editing efficiencies were not calculated. Nevertheless, it could be seen that editing efficiencies varied substantially between gRNAs and were comparable with those presented here for *EPSPS*. gRNAs targeting *TaDREB2* and *TaERF3* caused indels at a frequency of 6.7 and 10.2% respectively, based on the T7E1 assay [52]. It has been reported that approximately three or four gRNAs out of ten induce indels at a frequency of > 20% in wheat protoplasts (whereas in rice protoplasts the number is approximately seven or eight gRNAs out of ten) [47]. Out of seven gRNAs, we found that one induced indels at a frequency of almost 20%, two induced indels at a frequency of 7–10%, and four induced indels at a frequency of < 3% (based on data presented in Table 1). Taken together, this limited dataset suggests that gRNAs with high activity in wheat are likely the exception rather than the rule. Moreover, we did not find any obvious correlation between experimentally determined and in silico predicted on-target gRNA activity, which suggests that further improvements to bioinformatics tools for gRNA design are needed. For these reasons, we consider it prudent to carry out gRNA validation prior to commencing experiments for the production of stably edited wheat plants.

We observed low levels of gRNA activity at off-target homoeoalleles of *EPSPS*. Our results are consistent with established models of gRNA specificity [27–30] in which: a) the absence of a canonical PAM site (5′-NGG-3′) greatly reduces or abolishes gRNA activity; b) mismatches within the PAM-proximal 8–12 nucleotide seed region of the guide sequence reduce gRNA activity to a greater degree than mismatches outside the seed region; and c) additional mismatches further reduce gRNA activity. Importantly, a single mismatch within the seed region (at position 4 in gRNA1 and position 1 in gRNA2) greatly reduced but did not abolish gRNA activity. However, when the mismatch within the seed region was accompanied by another mismatch (at position 8 in gRNA1 and position 18 in gRNA2), gRNA activity was further reduced to levels that were undetectable with Sanger sequencing and barely detectable with amplicon deep sequencing. These results are consistent with previous reports, which showed that off-target mutations can occur in plants when there is only a single mismatch in the seed region [20, 25]. In these studies, frequencies of off-target mutagenesis were approximately 50–80% lower than frequencies of on-target mutagenesis [20, 25]. By contrast, the gRNA targeting *TaMLO-A1* (mentioned above) did not appear to generate any off-target mutations in homoeoalleles (*TaMLO-B1* and *TaMLO-D1*) in wheat protoplasts or transgenic T_0_ plants, due to the presence of a single mismatch at position 2 [50]. The apparent greater reduction in gRNA activity in the presence of a mismatch at position 1, 2 or 4 (compared with a mismatch at position 7, 8 or 9) may be due to the existence of five nucleotide ‘core’ within the seed region at the PAM-proximal end of the guide sequence [29, 30]. These results suggest that although off-target mutations are substantially reduced in the presence of a single mismatch in the seed region, they are often not eliminated. Therefore, ideally, potential off-target sites should lack a PAM, or else contain multiple mismatches, including at least one in the core of the seed region [24]. Where this is not possible, higher specificity may be achieved through the use of a truncated gRNA [53] and/or high fidelity variant of Cas9 [54]. Surprisingly, the activity of gRNA4 was apparently reduced on 4AL and 7DS, even though no mismatches were present. The reason for this is unknown. However, given that the reductions were less pronounced in the amplicon deep sequencing data (Fig. 2d), it would seem that this unexpected result is at least partly explained by the PCR and/or sequencing method used.

gRNA2 exhibited several properties that make it potentially suitable for the development of non-transgenic glyphosate resistant wheat. First, the canonical cut site for gRNA2 is adjacent to the universal mutation hotspot for resistance to glyphosate. This is important because the frequency of HDR tends to decrease as the distance between the DSB and the site of the desired mutation increases [55]. Second, gRNA2 was active at its target site on 7AS, although the activity was moderate. Third, gRNA2 was relatively specific for *EPSPS* on 7AS, which is the most highly transcribed copy of *EPSPS* in at least some wheat cultivars [45]. This high specificity would facilitate the creation of an *EPSPS* mutant that is edited on 7AS and wild type on 4AL/7DS. Such a mutant would have the desired trait (glyphosate resistance), and the yield penalty that could otherwise result from simultaneous modification or knockout of *EPSPS* on 4AL/7DS would be avoided.

One somewhat surprising finding in this study was the relatively high frequency of insertions (up to 8.5% of edited amplicon reads) that show 100% sequence identity to components of the DNA vectors used for transformation. These insertions are almost certainly vector-derived, and some (e.g. synthetic Cas9 sequences) are undoubtedly vector-derived. Recently, it was reported that DNA vector-derived insertions occur at very low frequencies (0.06–0.14% of edited amplicon reads) in Arabidopsis protoplasts transiently transformed with CRISPR-Cas9 vectors [56], although the authors state that the frequencies were likely underestimated because insertions of > 50 bp were excluded from the analysis. In addition to experimental differences, species-specific differences in NHEJ [57] may help to explain the much higher frequencies of DNA vector-derived insertions in wheat. If so, then sequence knockin via MMEJ may be a particularly effective genome editing strategy in wheat [19, 50]. On a related note, if DNA vector-derived sequences were to be found in an unregulated genome-edited crop (i.e. at an uncharacterised off-target site), it would set back both the regulatory authority and the industry [56]. Fortunately, recent advances in crop genome editing with in vitro transcribed RNA [20] and Cas9 ribonucleoproteins [51, 58, 59] suggest that the potential for unintended insertions of DNA vector-derived sequences can be eliminated in many cases. Where these methods cannot be used, unwanted DNA-vector derived insertions could be detected by whole genome sequencing and, if not linked to the target modification, removed by backcrossing.

Finally, this study demonstrates the utility of two methods for detecting and quantifying on-target and off-target indels: TIDE analysis of Sanger sequence traces, and CRISPResso analysis of amplicon reads. Although restriction enzyme-based assays such as PCR-RE and T7E1 are rapid, cheap and widely used for mutation detection, they suffer from several drawbacks and may not be suitable in some circumstances. For example, if the target site lacks a restriction site at the canonical cut site (as was the case with gRNA6 in this study), then the PCR-RE assay is impractical. On the other hand, the T7E1 enzyme recognises and cleaves mismatched DNA produced through denaturation and re-annealing of wild type and mutant PCR amplicons, so it does not require a restriction site. However, the T7E1 assay may produce results that are difficult to interpret if the wild type amplicons are polymorphic (a potential problem in polyploids) [60]. Moreover, restriction enzyme-based assays provide essentially no information about the indel spectrum or sequences of mutant alleles. Such information can be useful for the purpose of selecting gRNAs for particular applications [61, 62]. The sequencing-based mutation detection methods used in this study overcome the limitations described above, and come with the added benefit of greater sensitivity. Sanger sequencing combined with TIDE analysis takes only a few days and can be cost-effective for low/medium throughput screening, considering that sequencing of the opposite strand is unnecessary if the forward sequence trace is high quality. Amplicon deep sequencing combined with CRISPResso analysis takes longer and is only cost-effective for high throughput screening. In some cases, significant expertise in bioinformatics may be required for the analysis of amplicon reads derived from polyploid species. A number of other mutation detection methods have been established [63, 64]. Ultimately, the choice of mutation detection method should be made on a case-by-case basis, taking into consideration the goals of the experiment and the available resources [60].

## Conclusions

In summary, this study demonstrates that gRNA validation is an essential step in the application of the CRISPR-Cas9 system in wheat. gRNA validation should be carried out prior to commencing transformation and tissue culture experiments for the production of stably edited wheat plants. We have established a rapid and reliable method for assessing gRNA activity and specificity in hexaploid wheat. The method is based on an improved wheat protoplast transformation protocol, as well as the use of sequencing-based mutation detection techniques that overcome many of the limitations of commonly used enzyme-based assays. The method was used to identify a gRNA that could potentially be used for the production of non-transgenic glyphosate resistant wheat lines. Our approach is applicable to any plant species amenable to protoplast transformation, and should facilitate the adoption of CRISPR-Cas9 technology for genome editing in wheat and other polyploid crops.

## Methods

### Cloning and sequencing of *EPSPS* in wheat cv. Fielder

A full-length *Triticum aestivum EPSPS* cDNA consensus sequence (1789 bp) was retrieved from GenBank [EU977181] and used as the query for a BLASTN search against the *T. aestivum* EST database. The returned ESTs were assembled de novo into contigs using the Geneious Assembler in Geneious v9. The cDNA consensus sequence and EST-derived contigs were then aligned with genomic sequences from the TGACv1 wheat genome assembly (scaffold_569503_7AS:9611–10,115, scaffold_290435_4AL:41770–42,544 and scaffold_623048_7DS:39649–41,774), using the MUSCLE Alignment tool in Geneious. Based on this multiple sequence alignment, primers were designed (Additional file 16) to amplify a > 2 kb region of the three homoeologous copies of *EPSPS* in cv. Fielder. Amplicons were TOPO cloned into pCR8 (Invitrogen), and two independent pCR8-TaEPSPS-FL clones derived from each homoeoallele (based on diagnostic restriction enzyme digest) were validated by Sanger sequencing (Australian Genome Research Facility). The Sanger sequence reads were aligned to produce a consensus sequence for each homoeoallele. The consensus sequences were then incorporated into the multiple sequence alignment and used as the basis for gRNA design.

### gRNA design

Seven gRNAs were manually designed to target *EPSPS*. Target sites were 20–22 nucleotides in length, and were located immediately 5′ of a PAM sequence (5′-NGG-3′). An extra G nucleotide was appended to the 5′ end of gRNA6 in order to ensure efficient transcription of the gRNA expressed under the U6 promoter [65].

### In silico prediction of on-target gRNA activity

On-target gRNA activity was predicted using the sgRNA Designer [34, 66] and WU-CRISPR [32, 67] tools, according to the developers’ guidelines.

### Vector design and construction

All vectors were designed using Geneious software. To construct the gRNA vector, the gRNA expression cassette [9] consisting of the TaU6 promoter and a non-targeting gRNA was synthesised (GenScript) and TOPO cloned into pCR8 (Invitrogen). The *BbsI* site in the pCR8 backbone was then removed by digestion with *NheI* and self-ligation, resulting in pCR8-U6-NCgRNA (negative control for editing). To insert guide sequences into pCR8-U6-NCgRNA, the guide sequence oligos (Additional file 16) were first annealed by combining 1 μL of each oligo (100 μM) with 1X T4 DNA ligase buffer (Invitrogen) in a total reaction volume of 10 μL. The reaction was heated to 95 °C for 5 min and then left at room temperature for 30 min. Annealed oligos were inserted into pCR8-U6-NCgRNA by simultaneous digestion/ligation using 1 μL annealed oligos, 50 ng pCR8-U6-NCgRNA, 1X NEBuffer 2.1, 2 units *BbsI* (New England Biolabs), 1X T4 DNA ligase buffer, and 0.5 units T4 DNA ligase (Invitrogen) in a total reaction volume of 10 μL. Cycling conditions were as follows: 37 °C for 1 h, 15 °C for 1 min, 20 °C for 10 min (2 cycles), and finally 37 °C for 1 h. Positive clones of pCR8-U6-gRNA1/2/3/4/5/6/7 were identified by diagnostic double digest with *BbsI* and *EcoRI-HF* (New England Biolabs), and validated by Sanger sequencing (Australian Genome Research Facility).

To construct the Cas9 vector, the rice codon-optimised SpCas9 gene with N- and C-terminal nuclear localisation signals [9] was synthesised (GenScript) and inserted into the generic vector pUbi-rbcS as an *NcoI*–*AscI* fragment between the maize Ubiquitin 1 promoter [68, 69] and the wheat rbcS Class II terminator [70], resulting in pUbi-Cas9-rbcS.

To construct the YFP vector, the EYFP gene was inserted into pUbi-rbcS in the same manner as above, resulting in pUbi-YFP-rbcS.

### Protoplast isolation and transformation

Protoplast isolation and transformation was carried out as described [47], with several modifications. Seedlings of *T. aestivum* cv. Fielder were grown in potted soil within a growth chamber at 24 °C with a photoperiod of 12 h light (~ 100 μmol m^− 2^ s^− 1^) and 12 h dark, for 7–8 days. Only vigorous seedlings (five to eight in total) were used for protoplast isolation. A razor blade was used to make a shallow cut across the adaxial surface of the primary leaf, from which the abaxial epidermis was peeled off. Leaf peels were placed abaxial side down in a petri dish containing 0.6 M mannitol for 15 min. Leaf peels were then placed abaxial side down in a petri dish containing 10 mL of cell wall-dissolving enzyme solution [20 mM MES-KOH (pH 5.7), 1.5% (wt/vol) cellulase Onozuka RS, 0.75% (wt/vol) macerozyme R10, 0.6 M mannitol, 10 mM KCl, 10 mM CaCl_2_, 0.1% (wt/vol) BSA] for 3–4 h with very gentle agitation. After addition of one volume of W5 solution [2 mM MES-KOH (pH 5.7), 154 mM NaCl, 125 mM CaCl_2_, 5 mM KCl] [71], protoplasts were filtered through a 100 μm nylon mesh into a petri dish and then carefully transferred to a 30 mL round-bottom tube (Sarstedt 55.517). Protoplasts were centrifuged for 3 min at 80 x *g*, resuspended in 15 mL of W5 solution, and incubated on ice for 30 min. The W5 solution was removed, and the protoplasts were resuspended in 500 μL MMG solution [4 mM MES-KOH (pH 5.7), 0.4 M mannitol, 15 mM MgCl_2_] [71]. The protoplast concentration was determined by cell counting on a hemocytometer, and subsequently adjusted to 3.0 × 10^5^ cells/mL using MMG solution.

In an empty 2 mL tube, Ubi-Cas9-rbcS (20 μg, 3.5 pmol) was mixed with either pCR8-U6-gRNA1/2/3/4/5/6/7 (gRNAs targeting *EPSPS*) (20 μg, 10.5 pmol), pCR8-U6-NCgRNA (negative control for editing) (20 μg, 10.5 pmol), or pUbi-YFP-rbcS (positive control for transformation) (20 μg, 5.7 pmol). Transformation was carried out by adding (in quick succession) 200 μL of protoplasts and then 200 μL of PEG solution [40% (wt/vol) PEG-4000, 0.2 M mannitol, 100 mM CaCl_2_] to the tube containing pre-mixed DNA. The DNA/protoplast/PEG mixture was homogenised by gently flicking the tube, and then incubated for 15 min at room temperature. The transformation reaction was stopped by adding 840 μL of W5 solution and gently inverting the tube three times. The protoplasts were centrifuged for 2 min at 100 x *g*. The supernatant was removed and the protoplasts were resuspended in 500 μL W5 solution. The protoplasts were then transferred to 12-well plates (Sarstedt 83.3921.500) coated with 5% vol/vol fetal bovine serum (Sigma-Aldrich F4135), and incubated at 23 °C in the dark for 48 h.

The experiment was repeated twice more, from the seed planting step. Thus, there were three biological replicates for each treatment and control.

### Microscopy

After 16–24 h of incubation, protoplasts co-transformed with pUbi-Cas9-rbcS and pUbi-YFP-rbcS (positive control for transformation) were imaged using a Nikon Ni-E microscope equipped with a 490–500 nm excitation filter and a 520–560 nm emission filter (Adelaide Microscopy Waite Facility). Transformation efficiencies were calculated as the proportion of spherical protoplasts (*n* = 100, bright field image) that emitted yellow fluorescence (dark field image).

### Flow cytometry

After 20 h of incubation, protoplasts co-transformed with pUbi-Cas9-rbcS and pUbi-YFP-rbcS were subjected to flow cytometry using a BD Accuri C6. For the negative control, water was used instead of DNA.

### gDNA extraction

At the end of the 48 h incubation period, protoplasts were transferred to 2 mL tubes and centrifuged for 2 min at 100 x *g*. The supernatant was removed and gDNA was extracted from the protoplast pellet using the DNeasy Plant Mini Kit (QIAGEN) according to manufacturer’s instructions. DNA was eluted from the spin column with 28 μL of elution buffer.

### Sanger sequencing and TIDE analysis

To obtain amplicons for Sanger sequencing, a genomic region (1781 bp on 7AS, 1572 bp on 4AL, and 1701 bp on 7DS) containing all seven target sites was amplified by PCR using homoeoallele-specific primers (Additional file 16). PCR was performed using 30–40 ng gDNA template, 0.8 μM primers, 200 μM dNTPs, 1X Phusion HF buffer, and 0.6 units Phusion Hot Start Flex DNA Polymerase (New England Biolabs) in a total reaction volume of 50 μL. gDNA obtained from nulli-tetrasomic lines of *T. aestivum* cv. Chinese Spring was used as template in control PCR reactions to confirm that amplification was homoeoallele-specific. Cycling conditions for touchdown PCR were as follows: initial denaturation at 98 °C for 1 min, denaturation at 98 °C for 5 s, annealing at 68–63 °C (7AS and 7DS) or 66–61 °C (4AL) for 15 s, extension at 72 °C for 55 s, and final extension at 72 °C for 5 min. The starting annealing temperature was decreased by 0.5 °C each cycle for 10 cycles, followed by 30 cycles at the final annealing temperature. The PCR product was run on 1% agarose gel, from which amplicons were extracted using the NucleoSpin Gel and PCR Clean-up kit (Macherey-Nagel) according to the manufacturer’s instructions. DNA was eluted from the spin column with 15 μL of diluted (1 in 40) elution buffer and quantified using a NanoDrop 1000 spectrophotometer.

To detect targeted indels produced via NHEJ, homoeoallele-specific amplicons from each PCR reaction were subjected to Sanger sequencing (Australian Genome Research Facility) in the forward and reverse directions with nested homoeoallele-specific primers (Additional file 16). The 3730xl DNA Analyzer (Applied Biosystems) was used for sequencing, and bases were called with KB Basecaller v1.4.1.8. Output AB1 files for treated and untreated (negative control) samples were uploaded to the online TIDE analysis tool [42]. In TIDE, minor adjustments to the decomposition window were made based on information provided on the online TIDE analysis tool Troubleshooting webpage. All other TIDE settings were the default. The indel frequency for each gRNA/homoeoallele/replicate was calculated as the mean percent of sequences containing significant indels (*p* < 0.001) for the forward and reverse reads.

### Amplicon deep sequencing and CRISPResso analysis

To obtain amplicons for deep sequencing, two rounds of PCR were carried out. In the first round of PCR, a genomic region (269 bp on 7AS, and 270 bp on 4AL/7DS) containing all seven target sites was amplified using conserved primers containing 5′ universal tail sequences (Additional file 16) to which Illumina index primers anneal in the second round of PCR. PCR was performed using 20–40 ng gDNA template, 0.25 μM primers, 200 μM dNTPs, 1X Phusion HF buffer, and 0.2 units Phusion Hot Start Flex DNA Polymerase in a total reaction volume of 20 μL. Cycling conditions for touchdown PCR were as follows: initial denaturation at 98 °C for 1 min, denaturation at 98 °C for 5 s, annealing at 62–57 °C for 15 s, extension at 72 °C for 10 s, and final extension at 72 °C for 2 min. The starting annealing temperature was decreased by 0.5 °C each cycle for 10 cycles, followed by 25 cycles at the final annealing temperature. The PCR product was purified using Agencourt AMPure XP beads (Beckman Coulter) according to the manufacturer’s instructions. The second round of PCR was performed using 10 ng DNA template (purified amplicons from the first round of PCR), 0.3 μM primers (Illumina Nextera XT), 200 μM dNTPs, 1X Phusion HF buffer, and 0.2 units Phusion Hot Start Flex DNA Polymerase in a total reaction volume of 10 μL. Cycling conditions were as follows: initial denaturation at 98 °C for 1 min, denaturation at 98 °C for 5 s, annealing at 60 °C for 15 s, extension at 72 °C for 6 s, and final extension at 72 °C for 2 min (7 cycles in total). The indexed PCR products were purified using Agencourt AMPure XP beads.

Indexed PCR products were quantified by qPCR, diluted to 4 nM, pooled in equal volumes, spiked with 10% PhiX Control v3, and then sequenced on the Illumina MiSeq platform using the MiSeq Reagent Kit v3 600 cycle (Australian Genome Research Facility). The 300 bp unpaired raw reads from each sample were mapped to the three homoeologous amplicon reference sequences in two phases using Bowtie 2 [72]. The aim of the first phase was to map unedited reads using the following parameters: --end-to-end --very-sensitive --np 0 --mp 6,1 --rdg 999,999 --rfg 999,999 --score-min L,-6,0. Unmapped reads from the first phase were used as input for the second phase, where reads with indels (deletions of up to 51 bp or insertions of up to 4 bp) and some low quality mismatches were mapped using the following parameters: --end-to-end --very-sensitive --np 0 --mp 76,1 --rdg 24,1 --rfg 9,14 --score-min L,-75,0. Next, the resulting two BAM files were sliced for the reads that mapped to the respective three amplicons and merged together using SAMtools [73]. An in-house bash script was used to extract the mapped unedited/edited reads from the merged BAM files, and these sequence files in FASTQ format were used as input for CRISPResso [43] analysis using the following parameters: --w 20 --hide_mutations_outside_window_NHEJ --save_also_png --trim_sequences -q 30 --exclude_bp_from_left 5 --exclude_bp_from_right 5 --ignore_substitutions. Allele frequencies shown in Fig. 2c were calculated by summing the values in the %Reads column of the CRISPResso allele frequency table, after applying an Excel text filter (Custom AutoFilter) to only show rows where the aligned sequence contains the allele sequence.. For each of the three biological replicates, indel frequencies were calculated as the %NHEJ for the gRNA minus the %NHEJ for the negative control, based on data from the CRISPResso pie charts.

### Analysis of large insertions

To detect large insertions (≥20 bp), a separate CRISPResso analysis was carried out using unmapped amplicon deep sequencing reads as input, with the same CRISPResso settings as above. Data in CRISPResso allele frequency tables were sorted based on insertion size (largest to smallest), and then filtered to exclude aligned sequences containing insertions of < 20 bp. Reads containing insertions of ≥20 bp were aligned to the cv. Fielder consensus sequences in Geneious using the MUSCLE alignment tool. The sequences of the insertions were then searched for in the sequences of pUbi-Cas9-rbcS and pCR8-U6-gRNA2. Allele frequencies shown in Fig. 3 were calculated by summing the values in the %Reads column of the CRISPResso allele frequency table, after applying an Excel text filter (Custom AutoFilter) to only show rows where the aligned sequence contains the allele sequence.

## Supplementary information

**Additional file 1.** Bright field and dark field microscopy of protoplasts co-transformed with pUbi-Cas9-rbcS and pUbi-YFP-rbcS (A, C, E, G, I, K), and untransformed protoplasts (B, D, F, H, J, L). Three replicates (A-D, E-H, and I-L) for each treatment are shown, along with the transformation efficiency for each replicate (% of protoplasts transformed). Scale bar = 100 μm.

**Additional file 2.** Flow cytometry of protoplasts co-transformed with pUbi-Cas9-rbcS and pUbi-YFP-rbcS. Protoplasts were diluted to different concentrations (50,000–500,000 cells per 200 μL) for transformation, and either incubated with the DNA for 10 min prior to the addition of PEG or not incubated with the DNA (PEG added immediately to DNA/protoplast mixture). The percent of protoplasts expressing YFP is indicated. MFI, mean fluorescence intensity of YFP-expressing protoplasts.

**Additional file 3.** Forward and reverse Sanger sequence reads of homoeoallele-specific amplicons derived from protoplasts treated with gRNA1.

**Additional file 4.** Forward and reverse Sanger sequence reads of homoeoallele-specific amplicons derived from protoplasts treated with gRNA2.

**Additional file 5.** Forward and reverse Sanger sequence reads of homoeoallele-specific amplicons derived from protoplasts treated with gRNA3.

**Additional file 6.** Forward and reverse Sanger sequence reads of homoeoallele-specific amplicons derived from protoplasts treated with gRNA4.

**Additional file 7.** Forward and reverse Sanger sequence reads of homoeoallele-specific amplicons derived from protoplasts treated with gRNA5.

**Additional file 8.** Forward and reverse Sanger sequence reads of homoeoallele-specific amplicons derived from protoplasts treated with gRNA6.

**Additional file 9.** Forward and reverse Sanger sequence reads of homoeoallele-specific amplicons derived from protoplasts treated with gRNA7.

**Additional file 10.** Forward and reverse Sanger sequence reads of homoeoallele-specific amplicons derived from protoplasts treated with a non-targeting (random guide sequence) negative control gRNA.

**Additional file 11 **Homoeoallele-specific amplification of *EPSPS* on chromosomes 7AS, 4AL and 7DS following transient co-expression of Cas9 and gRNA in wheat protoplasts. Nulli-tetra (−) genomic DNA template does not contain the target chromosome. Nulli-tetra (+) genomic DNA template does contain the target chromosome. NT, no template; *gRNA, non-targeting (random guide sequence) gRNA. Three replicates were performed. All bands to the right of the ladder were gel purified and Sanger sequenced in the forward and reverse directions.

**Additional file 12 **TIDE indel spectra/frequencies for gRNAs 1–7 targeting *EPSPS* in wheat protoplasts. Shown are results for forward and reverse Sanger sequence reads of homoeoallele-specific amplicons derived from chromosomes 7AS, 4AL, and 7DS. Three replicates were performed.

**Additional file 13.** CRISPResso allele frequency tables used for analysis of indels induced by gRNA2 in replicate 1.

**Additional file 14.** CRISPResso NHEJ pie charts.

**Additional file 15.** CRISPResso allele frequency tables used for analysis of large insertions (≥20 bp) induced by gRNA2 and gRNA5 in replicate 3.

**Additional file 16.** Primers and oligonucleotides used in this study.

## Data Availability

The datasets supporting the conclusions of this article are included in the article and its additional files. The amplicon deep sequencing data have been deposited in the NCBI SRA repository [BioProject PRJNA420019; http://www.ncbi.nlm.nih.gov/bioproject/420019].

## Abbreviations

Cas9: CRISPR-associated protein 9
CRISPR: clustered regularly-interspaced short palindromic repeats
DSB: Double-strand break
EPSPS: 5-enolpyruvylshikimate-3-phosphate synthase
gRNA: guide RNA
HDR: Homology directed repair
NHEJ: Non-homologous end joining
PAM: Protospacer adjacent motif
PCR-RE: PCR-restriction enzyme
SNP: Single nucleotide polymorphism
TIDE: Tracking of Indels by DEcomposition
