# Supplementary figures and images for: gRNA validation for wheat genome editing with the CRISPR-Cas9 system (part 1 of 2)
Source: BMC Biotechnol. 2019 Oct 30;19:71. doi: 10.1186/s12896-019-0565-z (PMC6829922; doi:10.1186/s12896-019-0565-z)

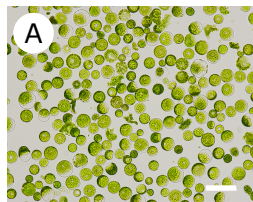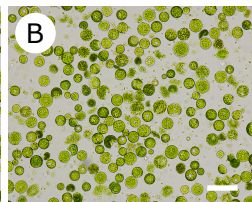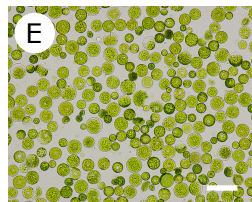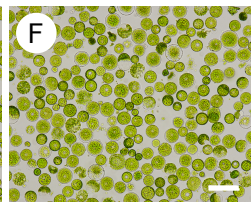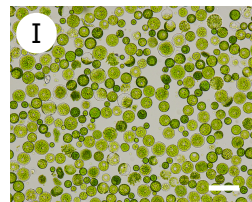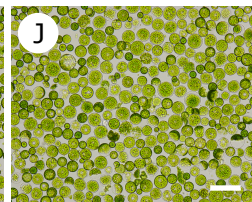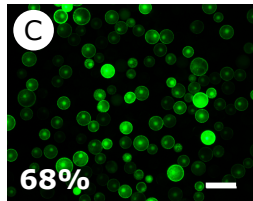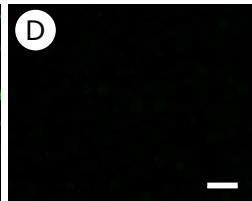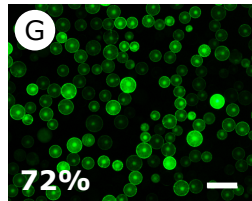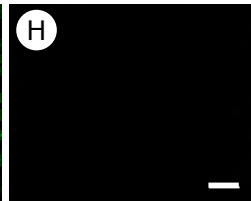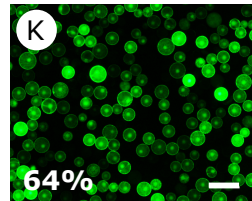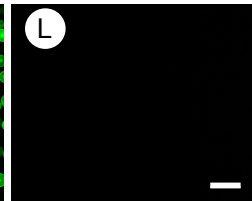

Supplement: Supplementary file 1 — Additional file 1. Bright field and dark field microscopy of protoplasts co-transformed with pUbi-Cas9-rbcS and pUbi-YFP-rbcS (A, C, E, G, I, K), and untransformed protoplasts (B, D, F, H, J, L). Three replicates (A-D, E-H, and I-L) for each treatment are shown, along with the transformation efficiency for each replicate (% of protoplasts transformed). Scale bar = 100 μm. [file 12896_2019_565_MOESM1_ESM.pdf]

10 min incubation

500,000 cells

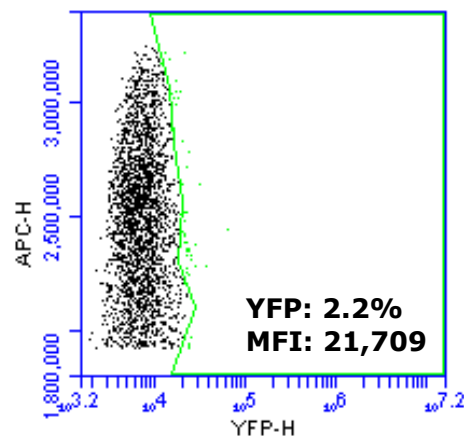

250,000 cells

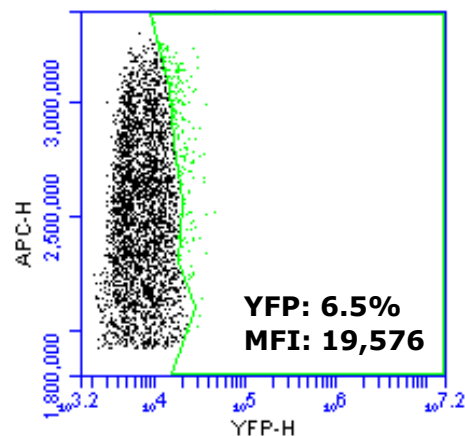

100,000 cells

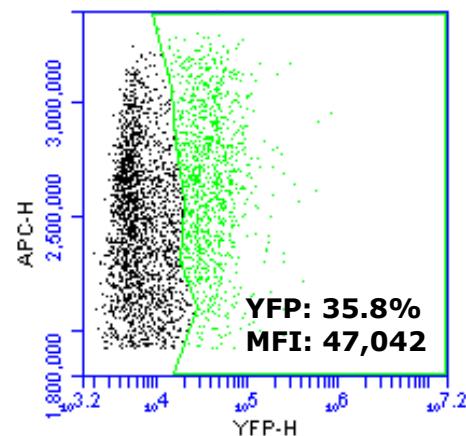

50,000 cells

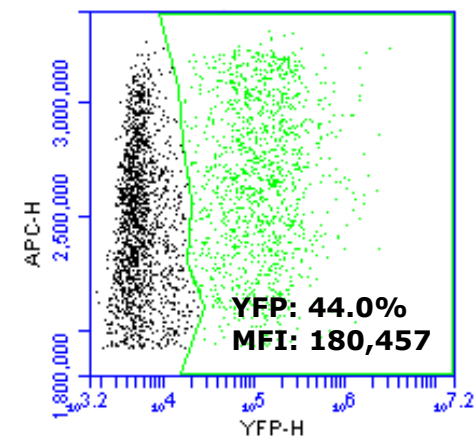

No incubation

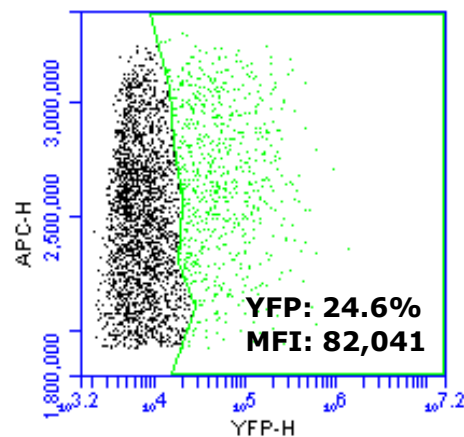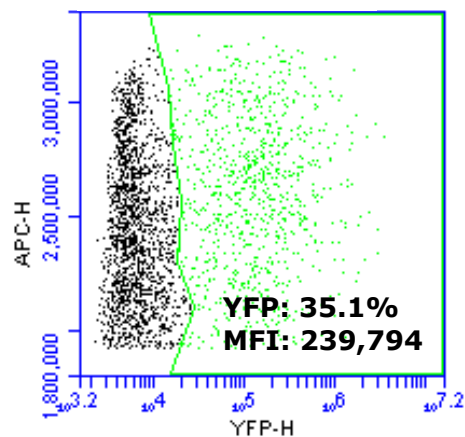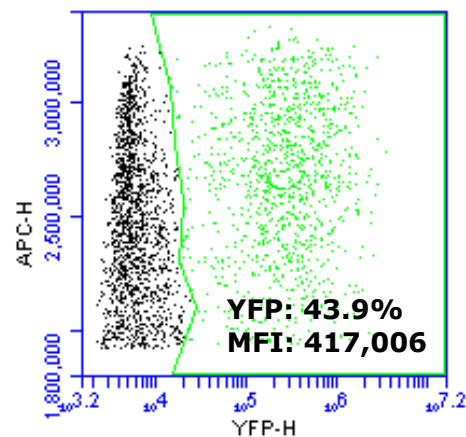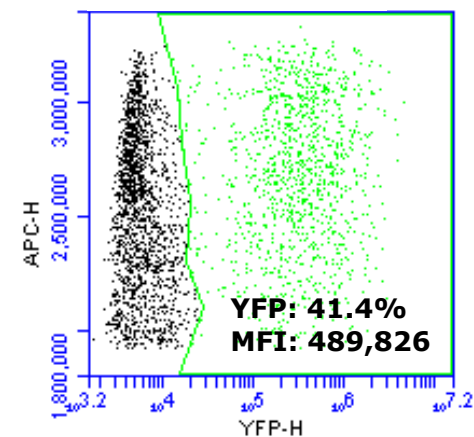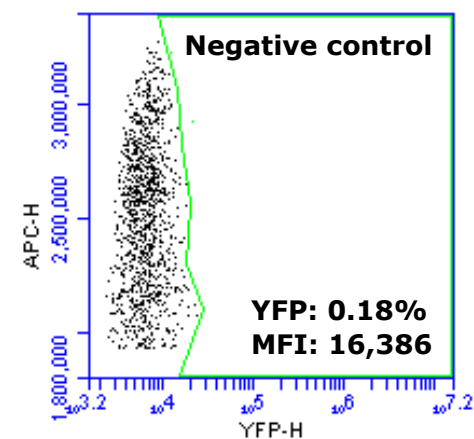

Supplement: Supplementary file 2 — Additional file 2. Flow cytometry of protoplasts co-transformed with pUbi-Cas9-rbcS and pUbi-YFP-rbcS. Protoplasts were diluted to different concentrations (50,000–500,000 cells per 200 μL) for transformation, and either incubated with the DNA for 10 min prior to the addition of PEG or not incubated with the DNA (PEG added immediately to DNA/protoplast mixture). The percent of protoplasts expressing YFP is indicated. MFI, mean fluorescence intensity of YFP-expressing protoplasts. [file 12896_2019_565_MOESM2_ESM.pdf]

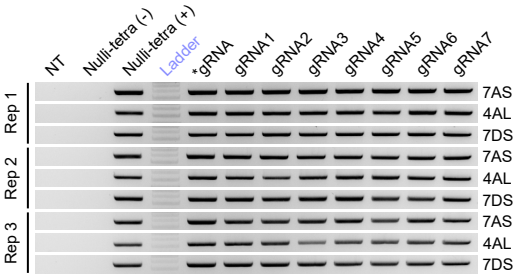

Supplement: Supplementary file 11 — Additional file 11 Homoeoallele-specific amplification of EPSPS on chromosomes 7AS, 4AL and 7DS following transient co-expression of Cas9 and gRNA in wheat protoplasts. Nulli-tetra (−) genomic DNA template does not contain the target chromosome. Nulli-tetra (+) genomic DNA template does contain the target chromosome. NT, no template; *gRNA, non-targeting (random guide sequence) gRNA. Three replicates were performed. All bands to the right of the ladder were gel purified and Sanger sequenced in the forward and reverse directions. [file 12896_2019_565_MOESM11_ESM.pdf]

Unmodified  
(18919 reads)

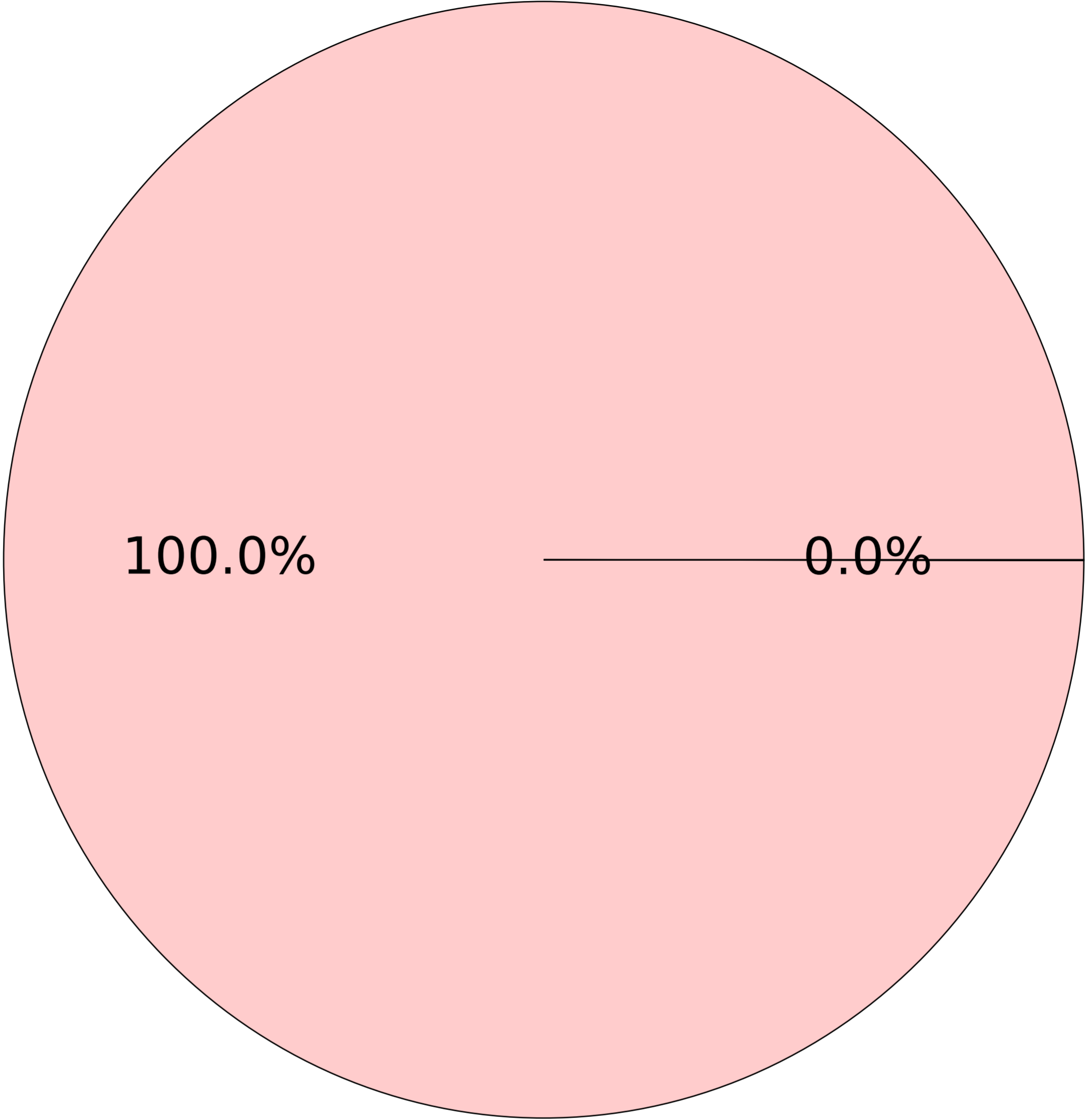

NHEJ  
(4 reads)

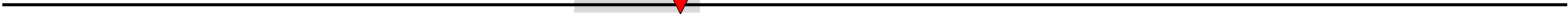

—

Amplicon sequence

—

sgRNA

▼

Predicted Cas9 cleavage site/s

Supplement: Supplementary file 14 — Additional file 14. CRISPResso NHEJ pie charts. [file 12896_2019_565_MOESM14_ESM.zip › CRISPResso_EPSPS-7DS-gRNA7-rep3-negative.pdf]

Unmodified  
(15769 reads)

100.0%

0.0%

NHEJ  
(5 reads)

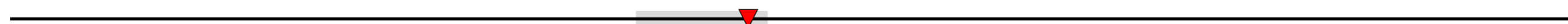

- Amplicon sequence
- sgRNA
- ▼ Predicted Cas9 cleavage site/s

Supplement: Supplementary file 14 — Additional file 14. CRISPResso NHEJ pie charts. [file 12896_2019_565_MOESM14_ESM.zip › CRISPResso_EPSPS-4AL-gRNA1-rep1.pdf]

Unmodified  
(7865 reads)

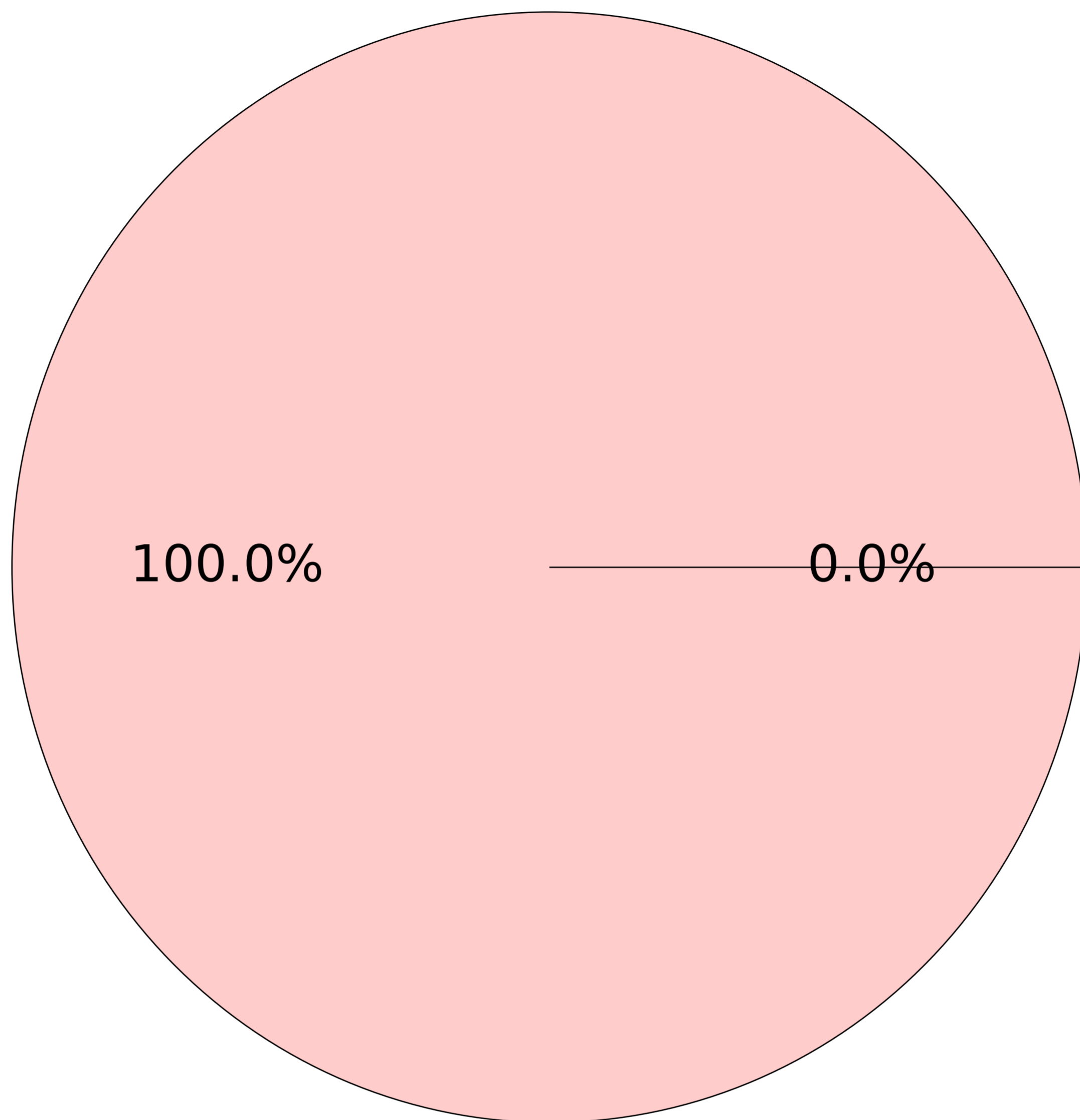

NHEJ  
(0 reads)

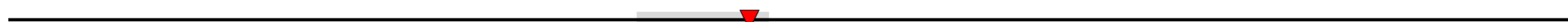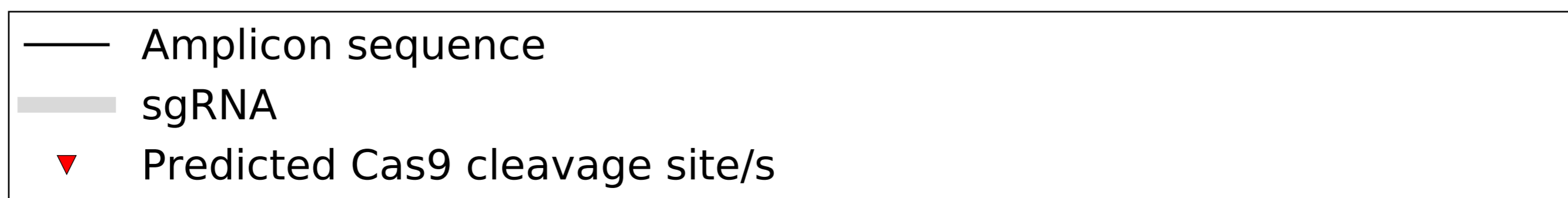

Supplement: Supplementary file 14 — Additional file 14. CRISPResso NHEJ pie charts. [file 12896_2019_565_MOESM14_ESM.zip › CRISPResso_EPSPS-4AL-gRNA1-rep1-negative.pdf]

Unmodified  
(16687 reads)

100.0%

0.0%

NHEJ  
(3 reads)

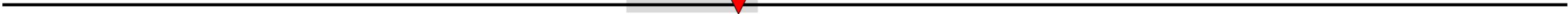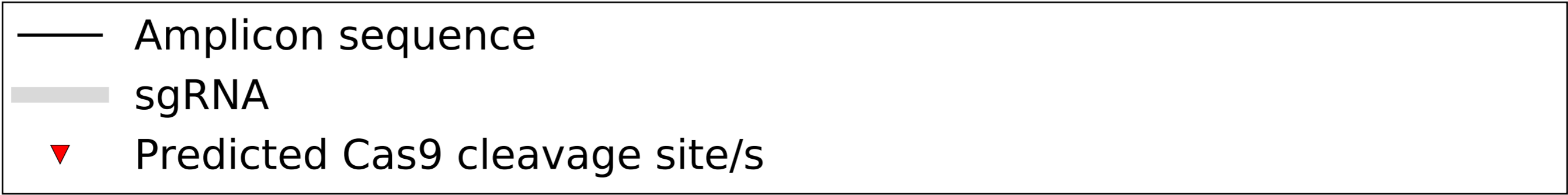

Supplement: Supplementary file 14 — Additional file 14. CRISPResso NHEJ pie charts. [file 12896_2019_565_MOESM14_ESM.zip › CRISPResso_EPSPS-4AL-gRNA1-rep2.pdf]

Unmodified  
(8110 reads)

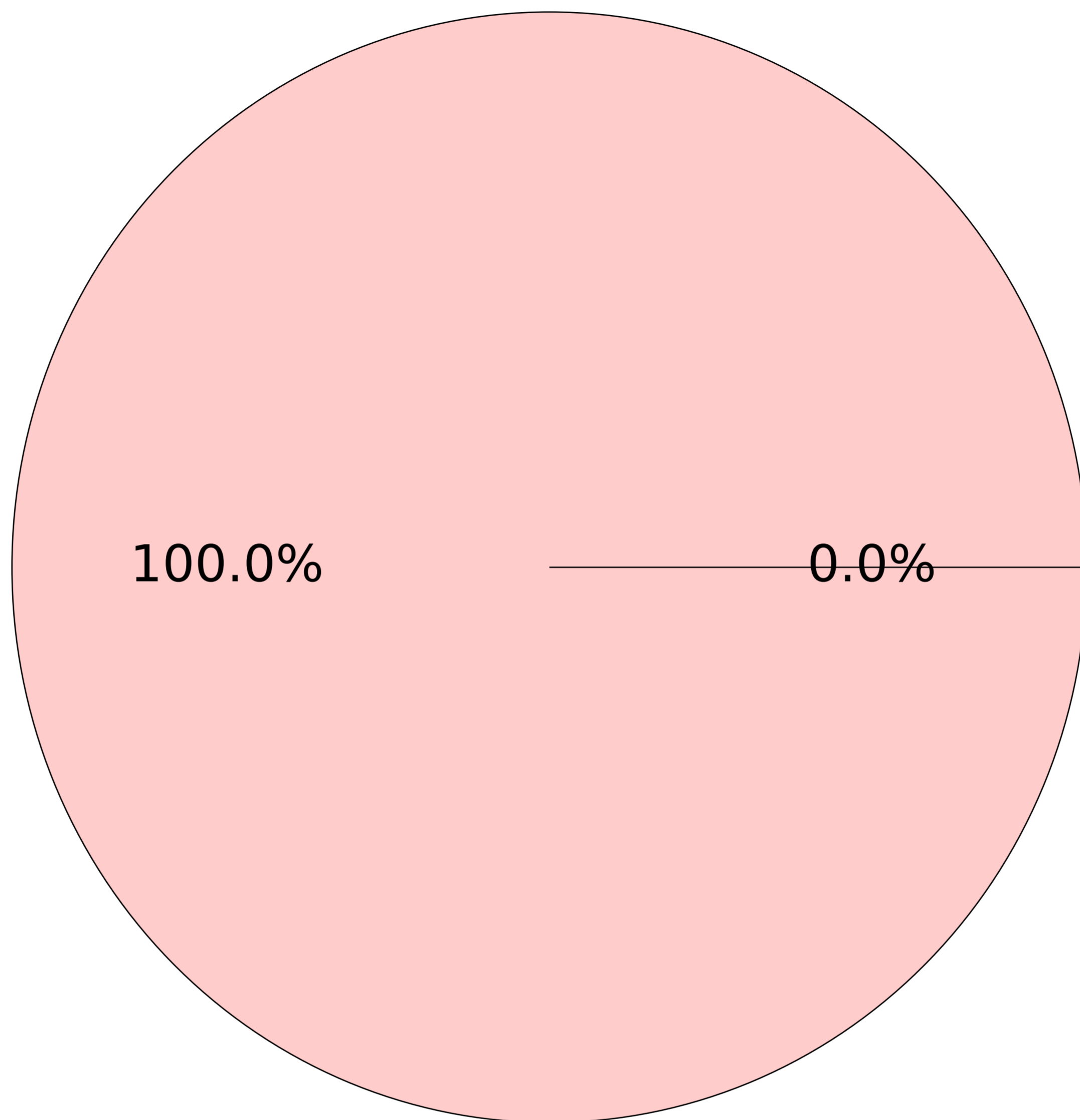

NHEJ  
(0 reads)

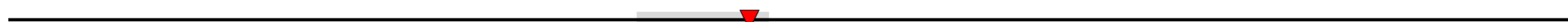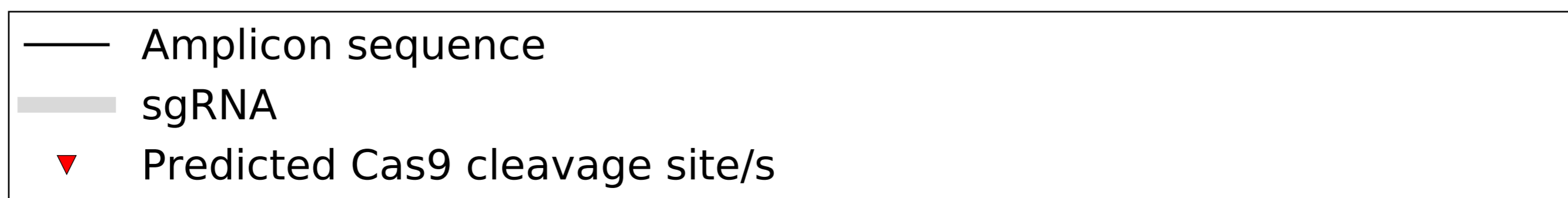

Supplement: Supplementary file 14 — Additional file 14. CRISPResso NHEJ pie charts. [file 12896_2019_565_MOESM14_ESM.zip › CRISPResso_EPSPS-4AL-gRNA1-rep2-negative.pdf]

Unmodified  
(15337 reads)

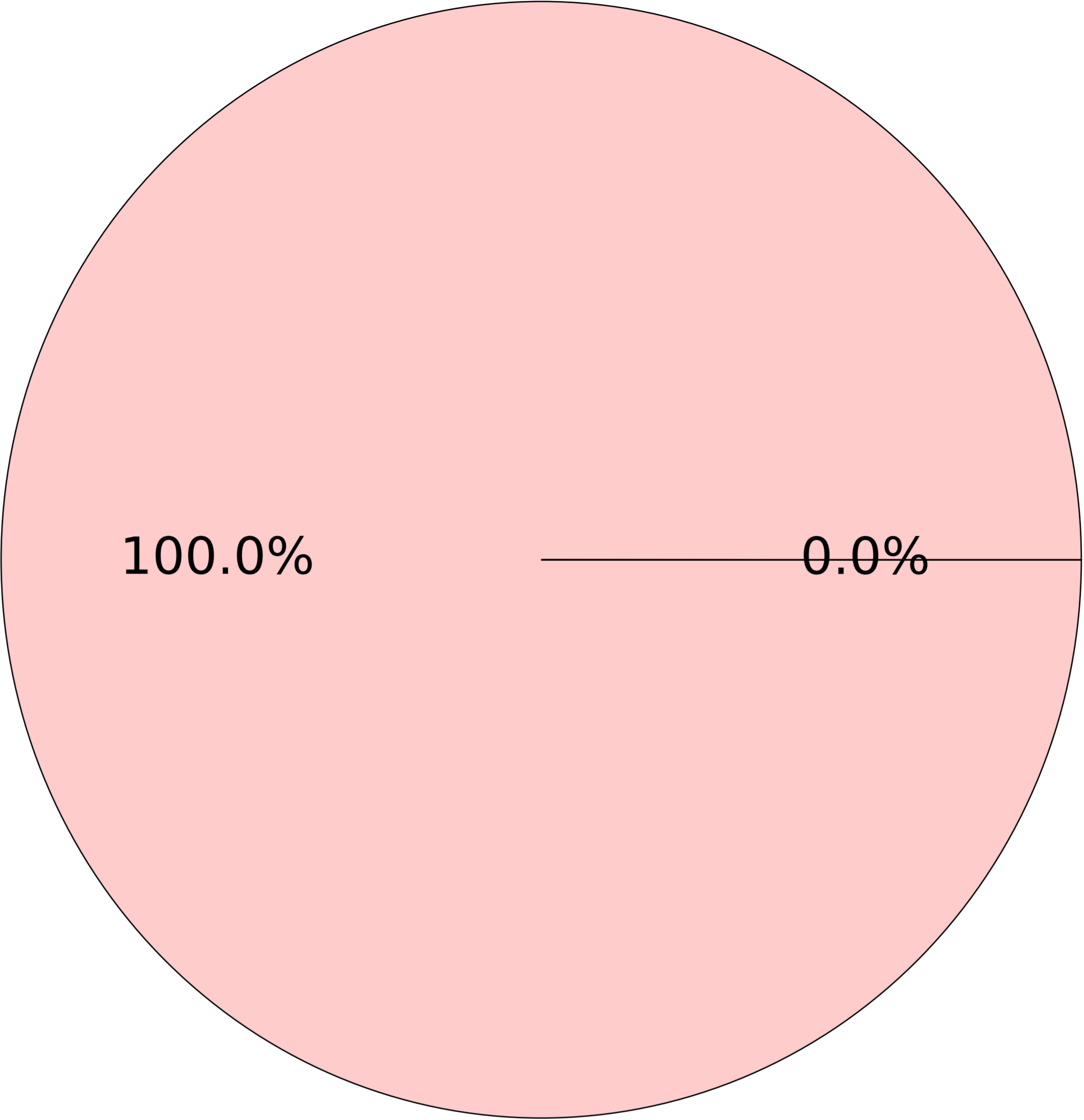

NHEJ  
(1 reads)

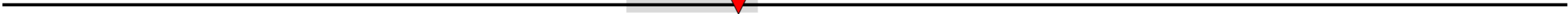

—

Amplicon sequence

—

sgRNA

▼

Predicted Cas9 cleavage site/s

Supplement: Supplementary file 14 — Additional file 14. CRISPResso NHEJ pie charts. [file 12896_2019_565_MOESM14_ESM.zip › CRISPResso_EPSPS-4AL-gRNA1-rep3.pdf]

Unmodified  
(14202 reads)

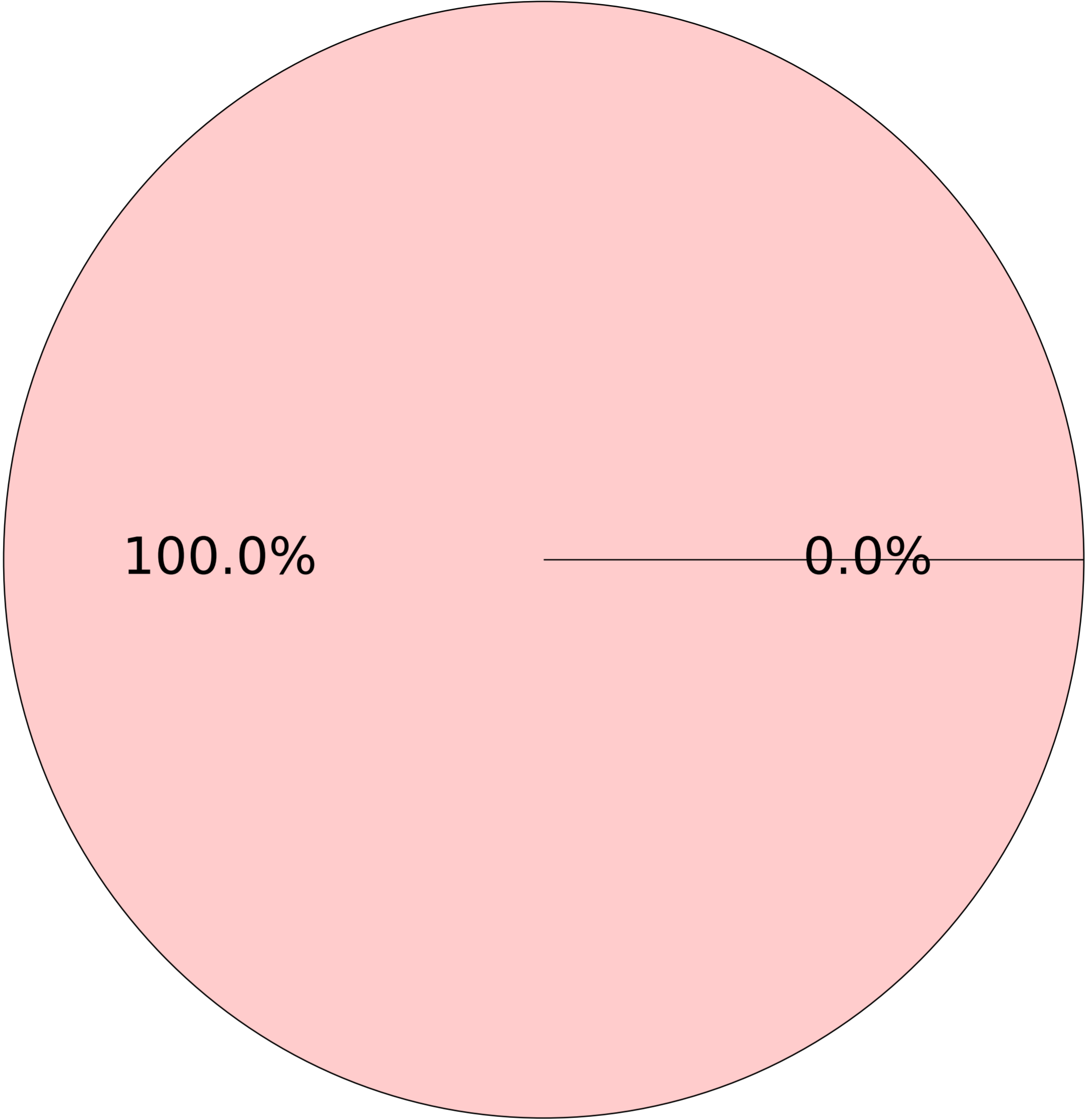

NHEJ  
(0 reads)

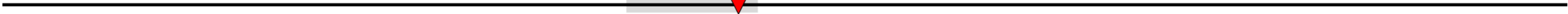

—

Amplicon sequence

—

sgRNA

▼

Predicted Cas9 cleavage site/s

Supplement: Supplementary file 14 — Additional file 14. CRISPResso NHEJ pie charts. [file 12896_2019_565_MOESM14_ESM.zip › CRISPResso_EPSPS-4AL-gRNA1-rep3-negative.pdf]

Unmodified  
(13834 reads)

100.0%

0.0%

NHEJ  
(3 reads)

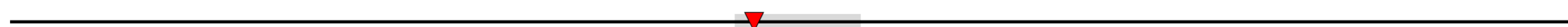

- Amplicon sequence
- sgRNA
- ▼ Predicted Cas9 cleavage site/s

Supplement: Supplementary file 14 — Additional file 14. CRISPResso NHEJ pie charts. [file 12896_2019_565_MOESM14_ESM.zip › CRISPResso_EPSPS-4AL-gRNA2-rep1.pdf]

Unmodified  
(7865 reads)

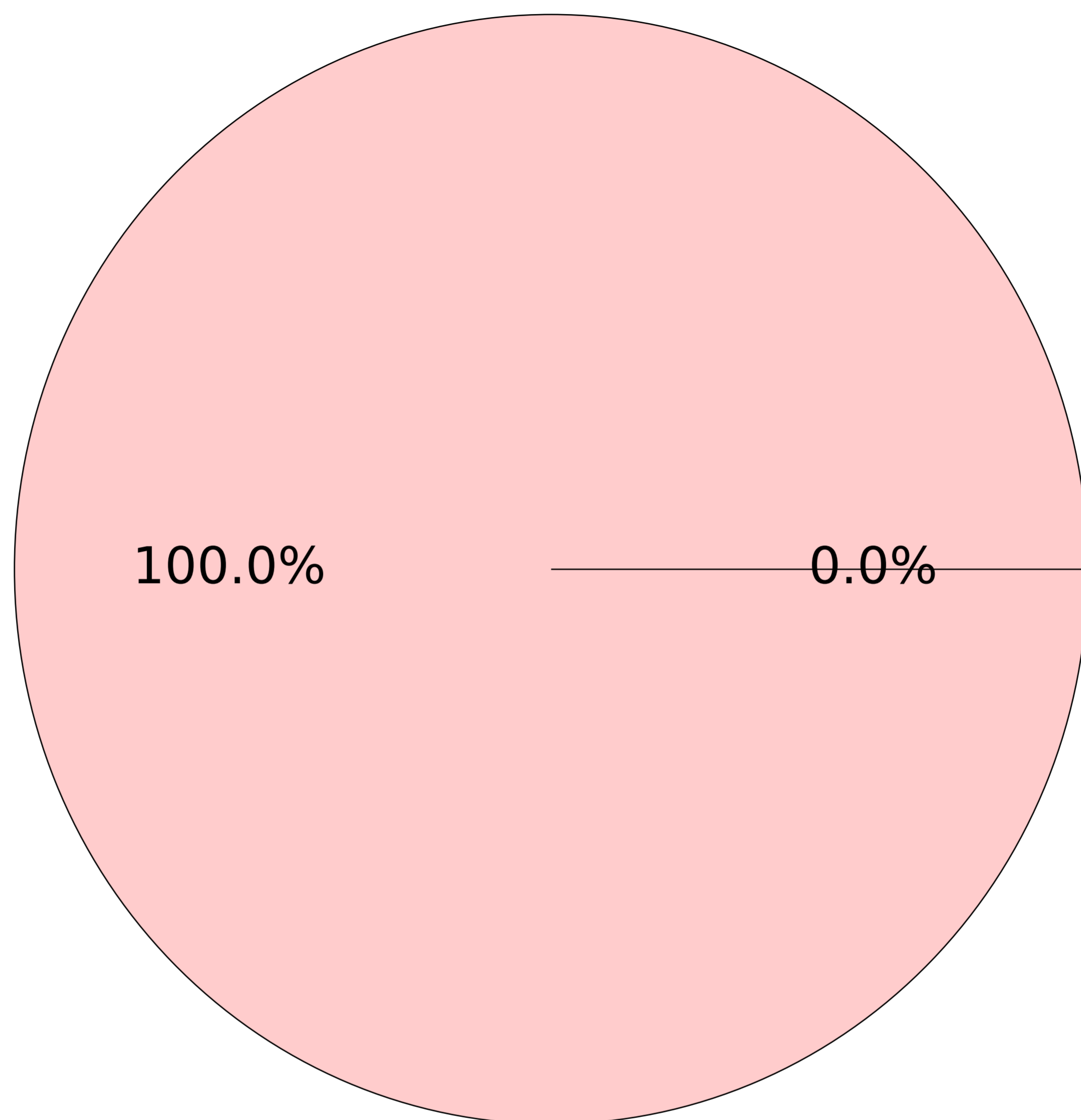

NHEJ  
(0 reads)

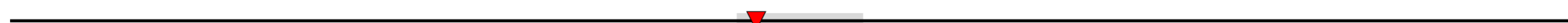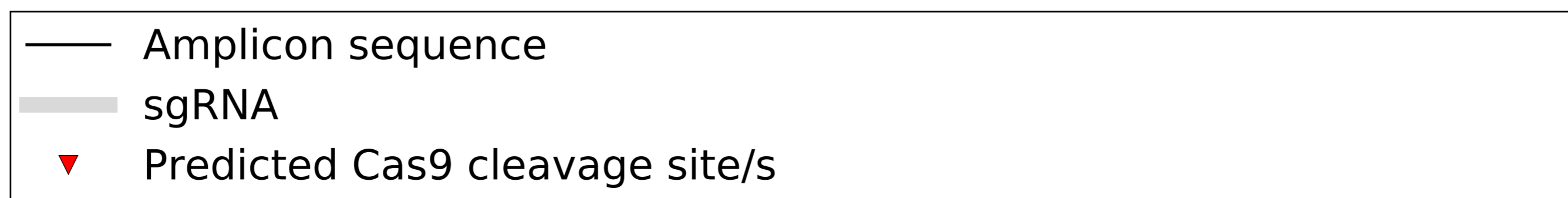

Supplement: Supplementary file 14 — Additional file 14. CRISPResso NHEJ pie charts. [file 12896_2019_565_MOESM14_ESM.zip › CRISPResso_EPSPS-4AL-gRNA2-rep1-negative.pdf]

Unmodified  
(7066 reads)

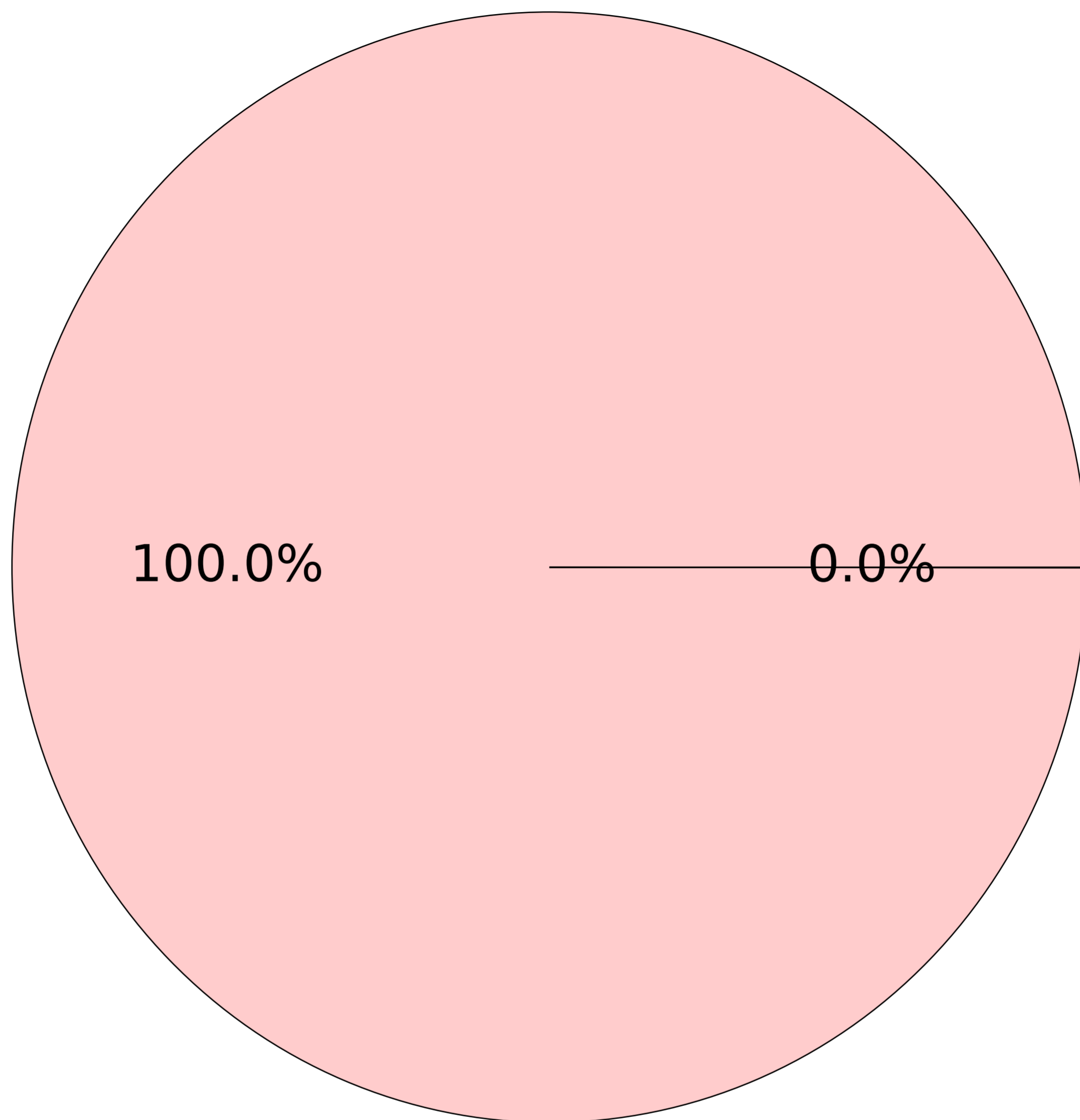

NHEJ  
(1 reads)

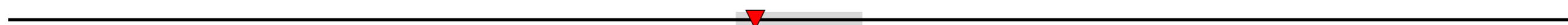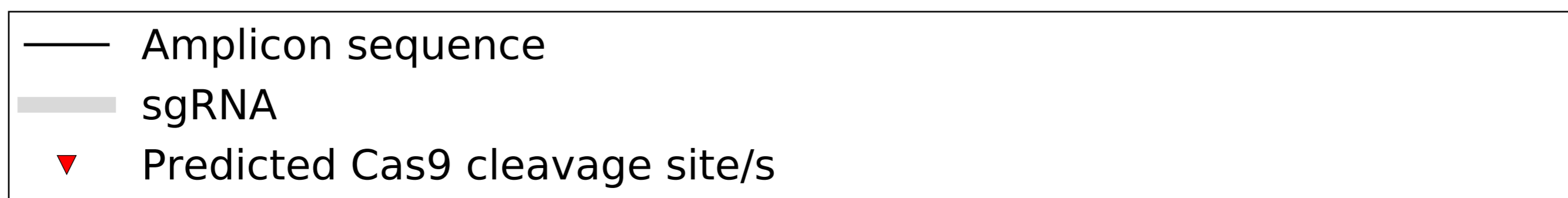

Supplement: Supplementary file 14 — Additional file 14. CRISPResso NHEJ pie charts. [file 12896_2019_565_MOESM14_ESM.zip › CRISPResso_EPSPS-4AL-gRNA2-rep2.pdf]

Unmodified  
(8110 reads)

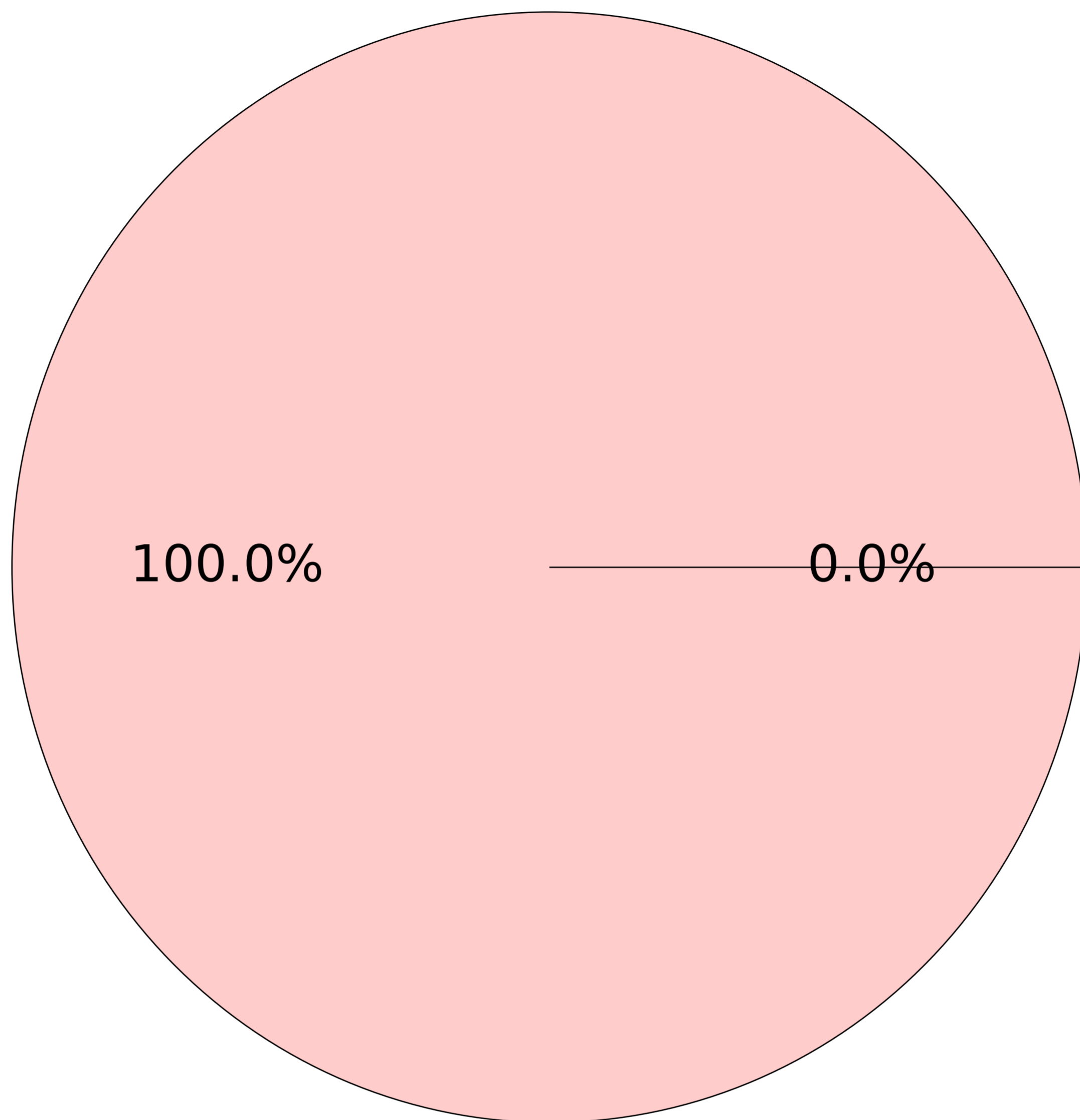

NHEJ  
(0 reads)

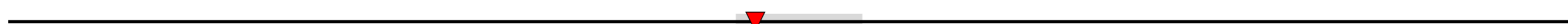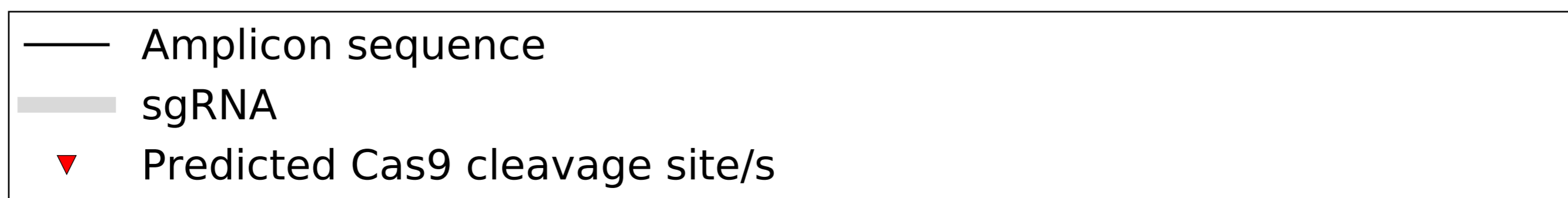

Supplement: Supplementary file 14 — Additional file 14. CRISPResso NHEJ pie charts. [file 12896_2019_565_MOESM14_ESM.zip › CRISPResso_EPSPS-4AL-gRNA2-rep2-negative.pdf]

Unmodified  
(10437 reads)

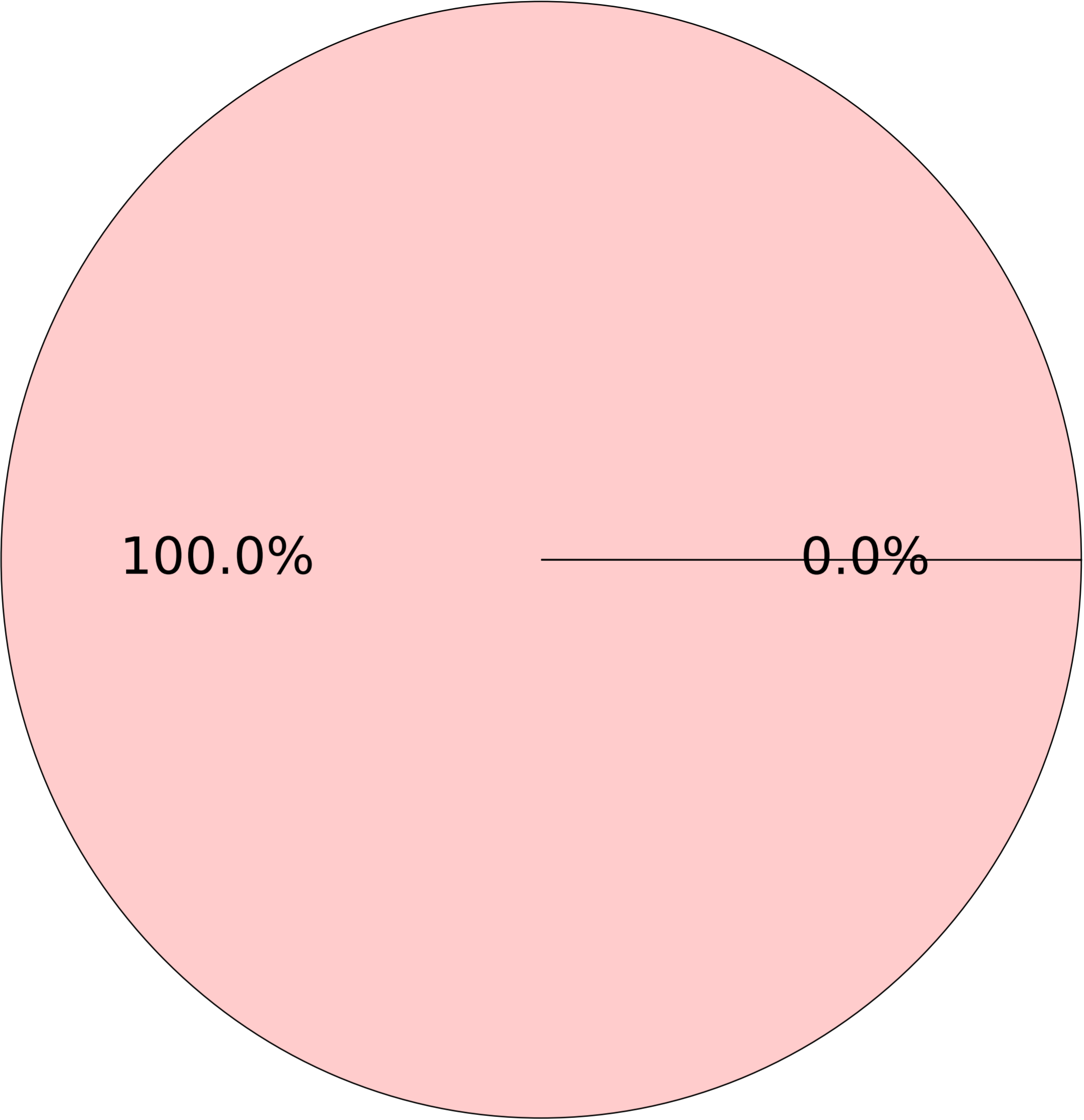

NHEJ  
(1 reads)

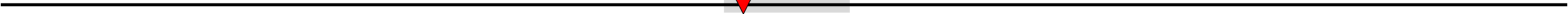

—

Amplicon sequence

—

sgRNA

▼

Predicted Cas9 cleavage site/s

Supplement: Supplementary file 14 — Additional file 14. CRISPResso NHEJ pie charts. [file 12896_2019_565_MOESM14_ESM.zip › CRISPResso_EPSPS-4AL-gRNA2-rep3.pdf]

Unmodified  
(14202 reads)

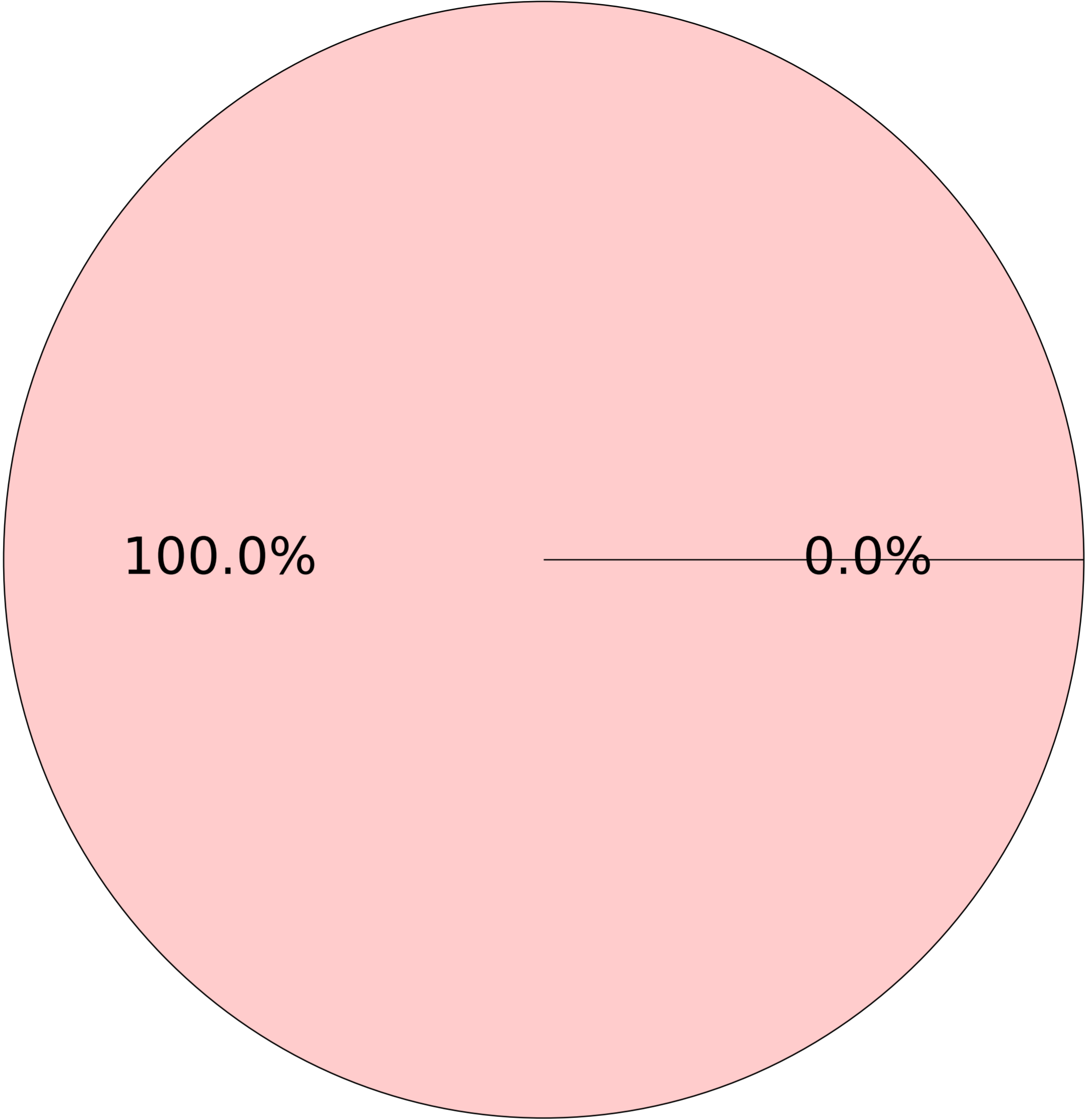

NHEJ  
(0 reads)

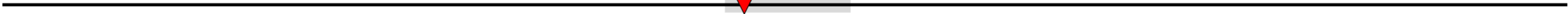

—

Amplicon sequence

—

sgRNA

▼

Predicted Cas9 cleavage site/s

Supplement: Supplementary file 14 — Additional file 14. CRISPResso NHEJ pie charts. [file 12896_2019_565_MOESM14_ESM.zip › CRISPResso_EPSPS-4AL-gRNA2-rep3-negative.pdf]

Unmodified  
(17057 reads)

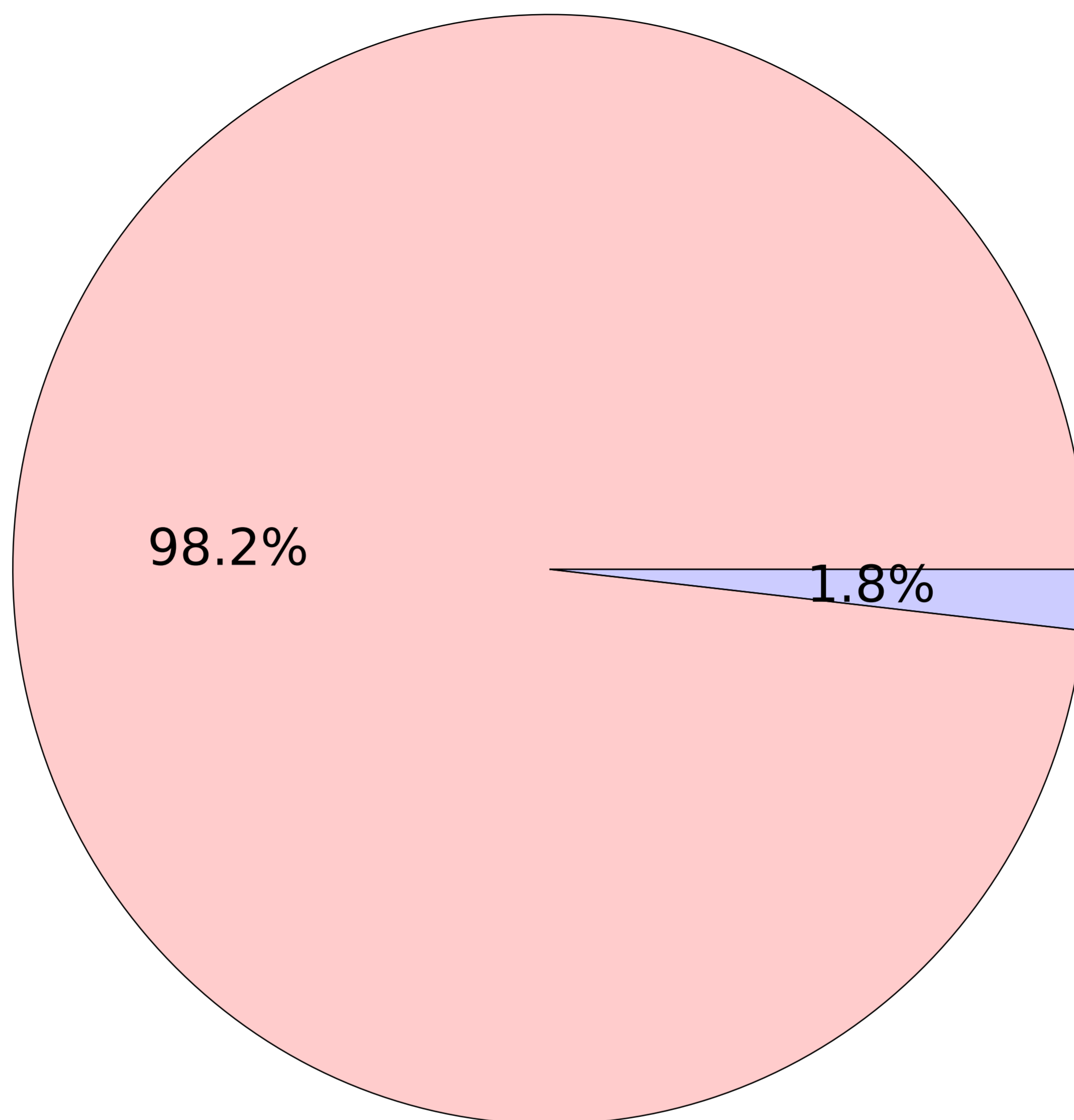

NHEJ  
(306 reads)

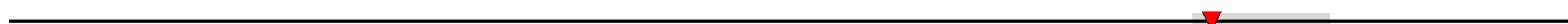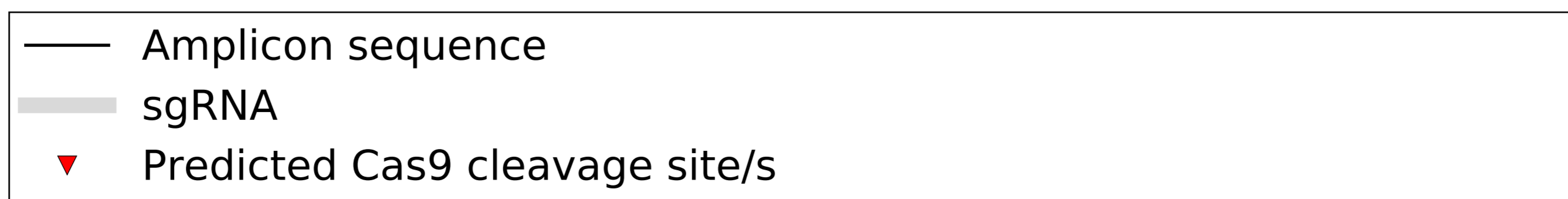

Supplement: Supplementary file 14 — Additional file 14. CRISPResso NHEJ pie charts. [file 12896_2019_565_MOESM14_ESM.zip › CRISPResso_EPSPS-4AL-gRNA3-rep1.pdf]

Unmodified  
(7861 reads)

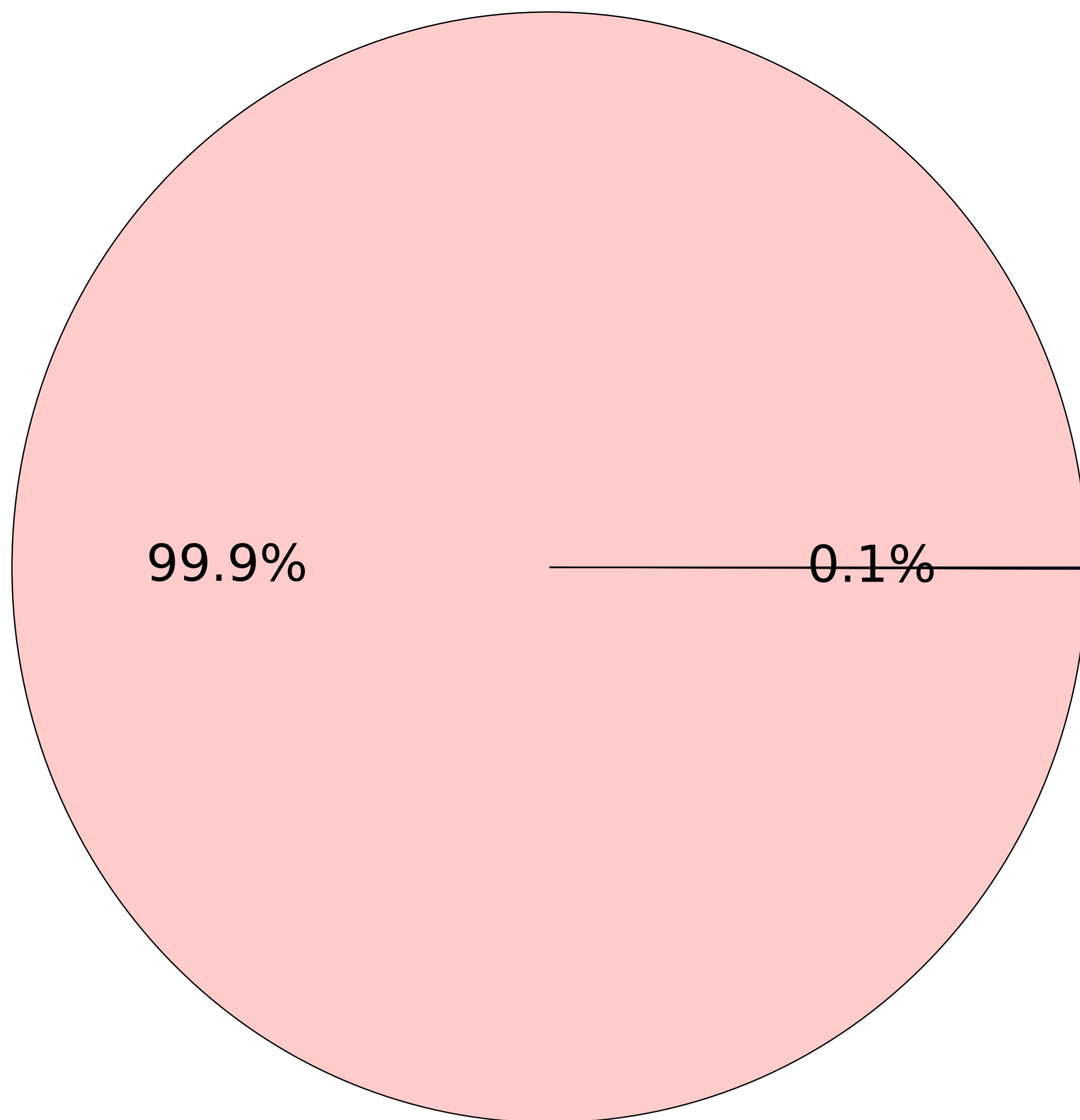

NHEJ  
(4 reads)

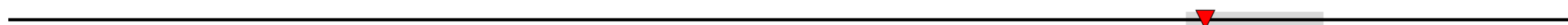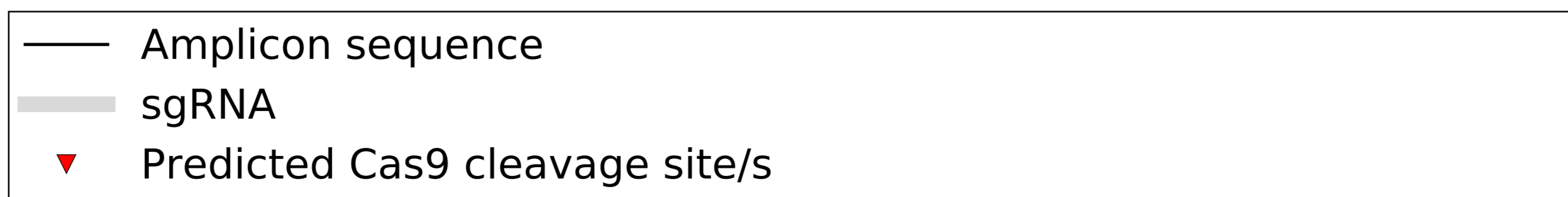

Supplement: Supplementary file 14 — Additional file 14. CRISPResso NHEJ pie charts. [file 12896_2019_565_MOESM14_ESM.zip › CRISPResso_EPSPS-4AL-gRNA3-rep1-negative.pdf]

Unmodified  
(10549 reads)

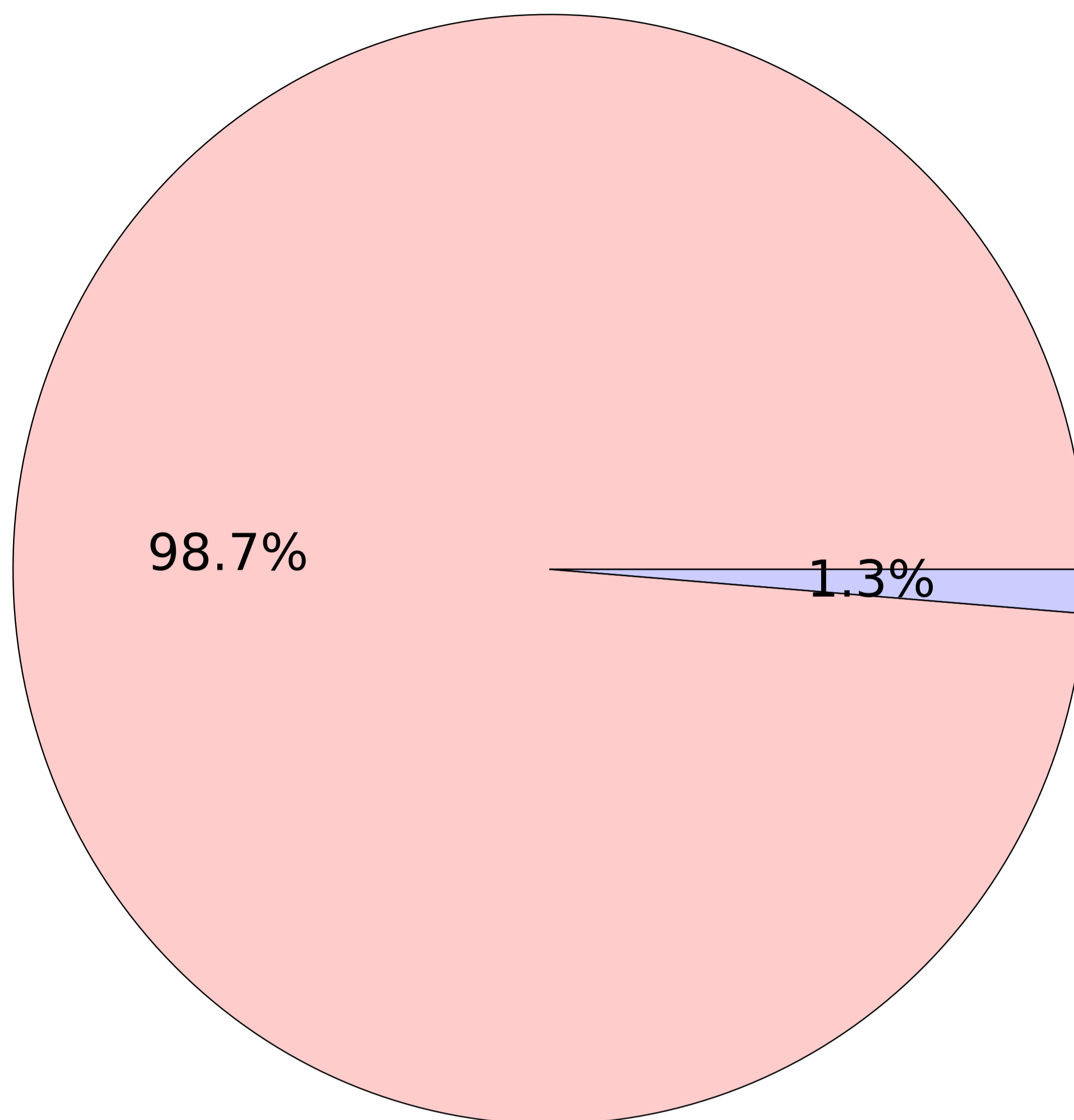

NHEJ  
(136 reads)

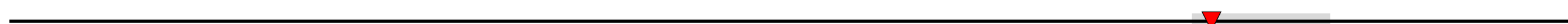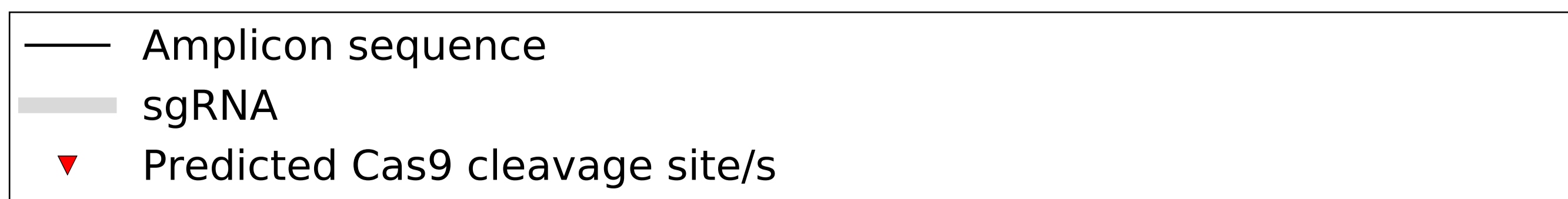

Supplement: Supplementary file 14 — Additional file 14. CRISPResso NHEJ pie charts. [file 12896_2019_565_MOESM14_ESM.zip › CRISPResso_EPSPS-4AL-gRNA3-rep2.pdf]

Unmodified  
(8109 reads)

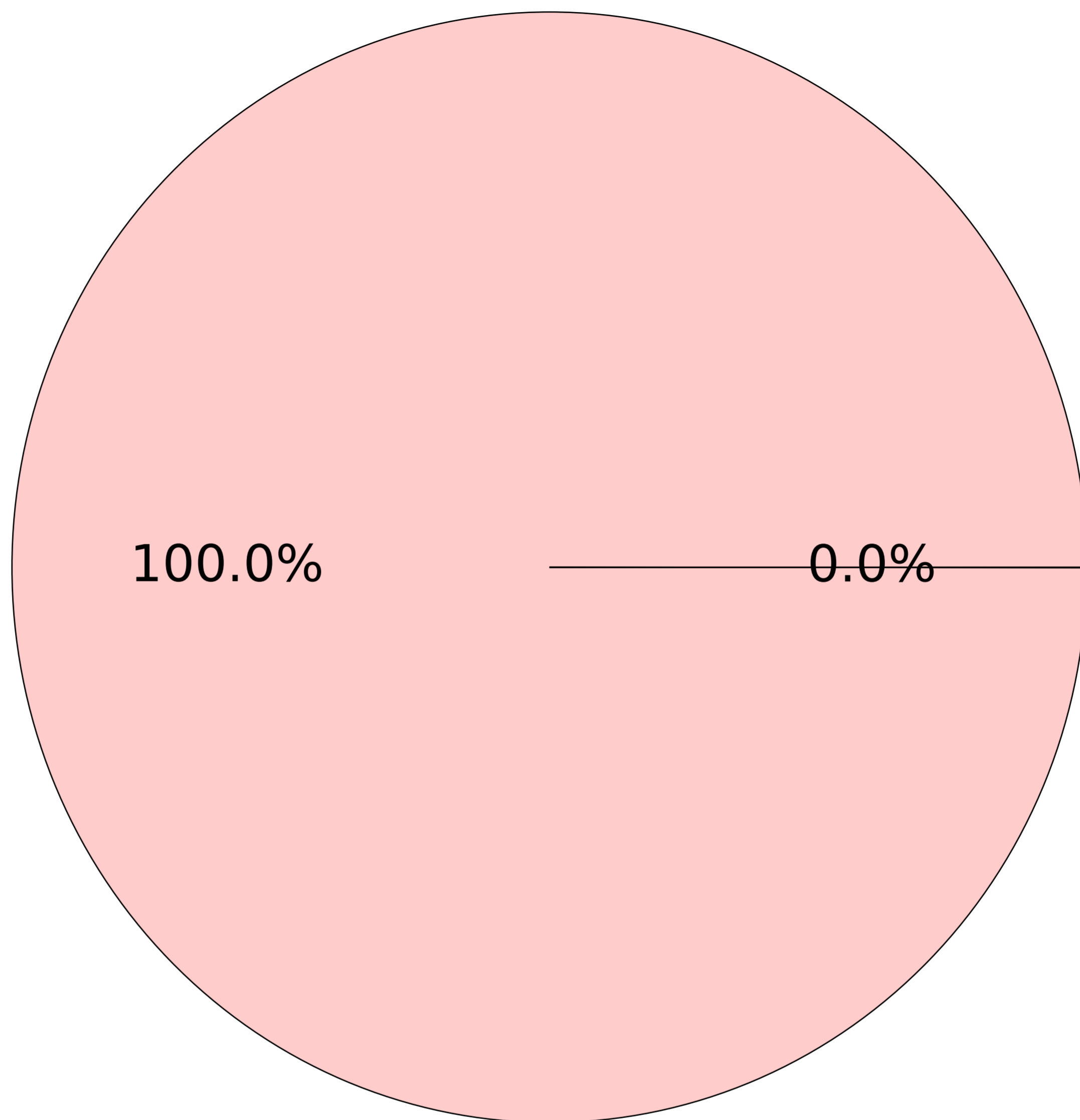

NHEJ  
(1 reads)

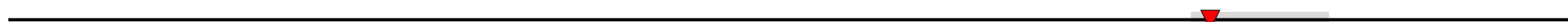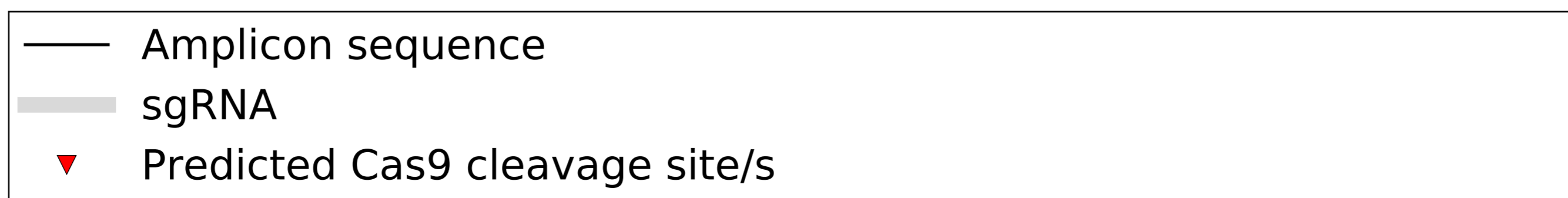

Supplement: Supplementary file 14 — Additional file 14. CRISPResso NHEJ pie charts. [file 12896_2019_565_MOESM14_ESM.zip › CRISPResso_EPSPS-4AL-gRNA3-rep2-negative.pdf]

Unmodified  
(11930 reads)

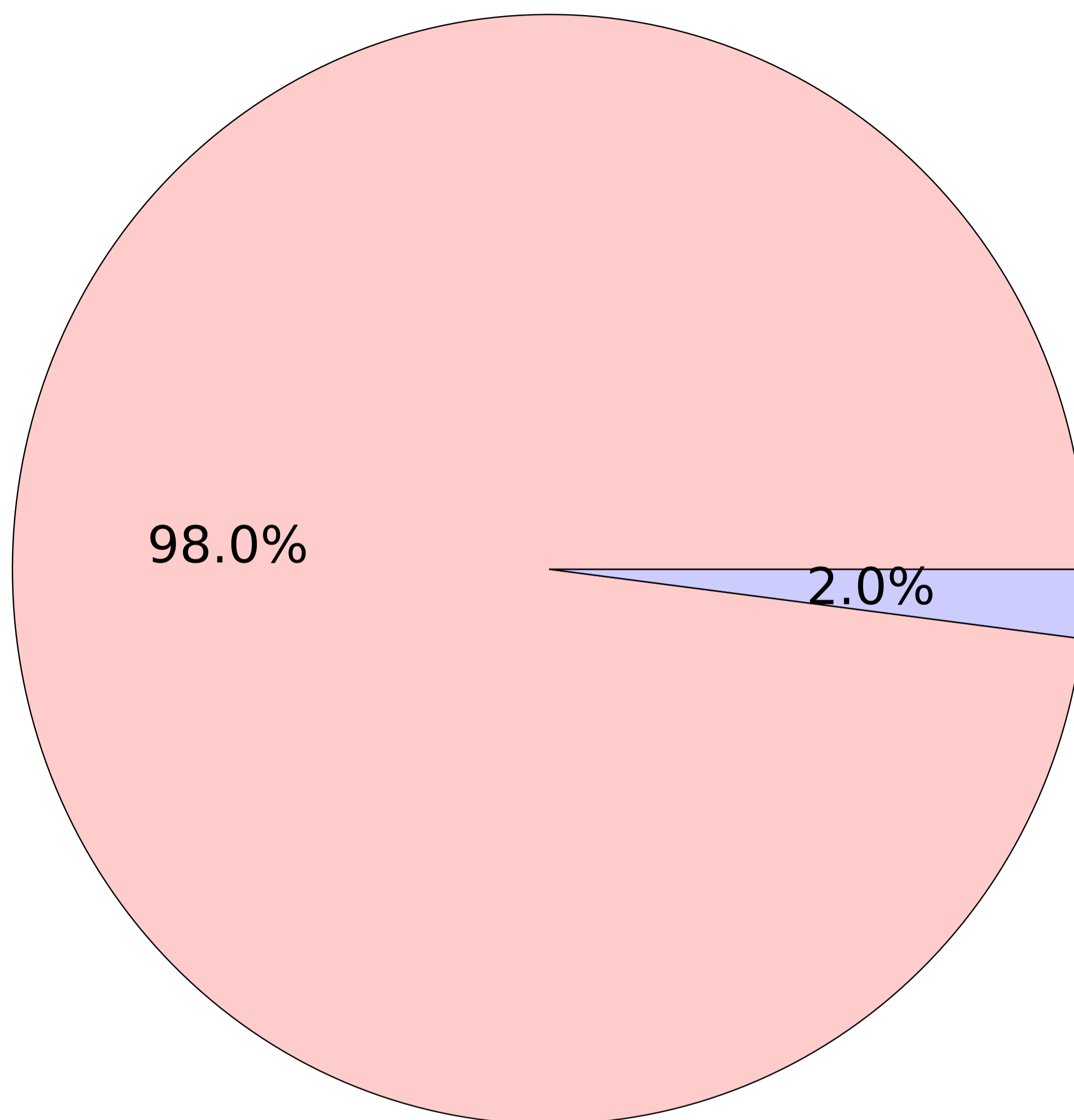

NHEJ  
(243 reads)

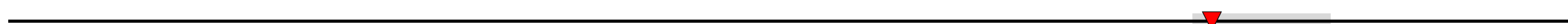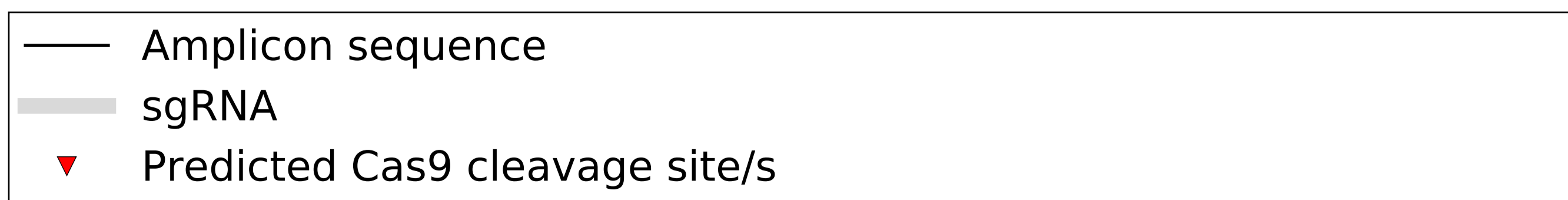

Supplement: Supplementary file 14 — Additional file 14. CRISPResso NHEJ pie charts. [file 12896_2019_565_MOESM14_ESM.zip › CRISPResso_EPSPS-4AL-gRNA3-rep3.pdf]

Unmodified  
(14200 reads)

100.0%

0.0%

NHEJ  
(2 reads)

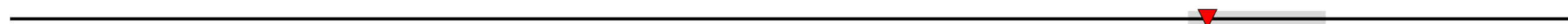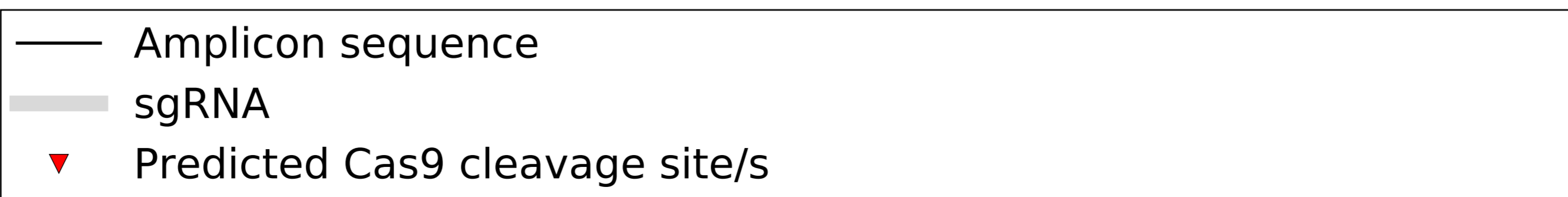

Supplement: Supplementary file 14 — Additional file 14. CRISPResso NHEJ pie charts. [file 12896_2019_565_MOESM14_ESM.zip › CRISPResso_EPSPS-4AL-gRNA3-rep3-negative.pdf]

Unmodified  
(11769 reads)

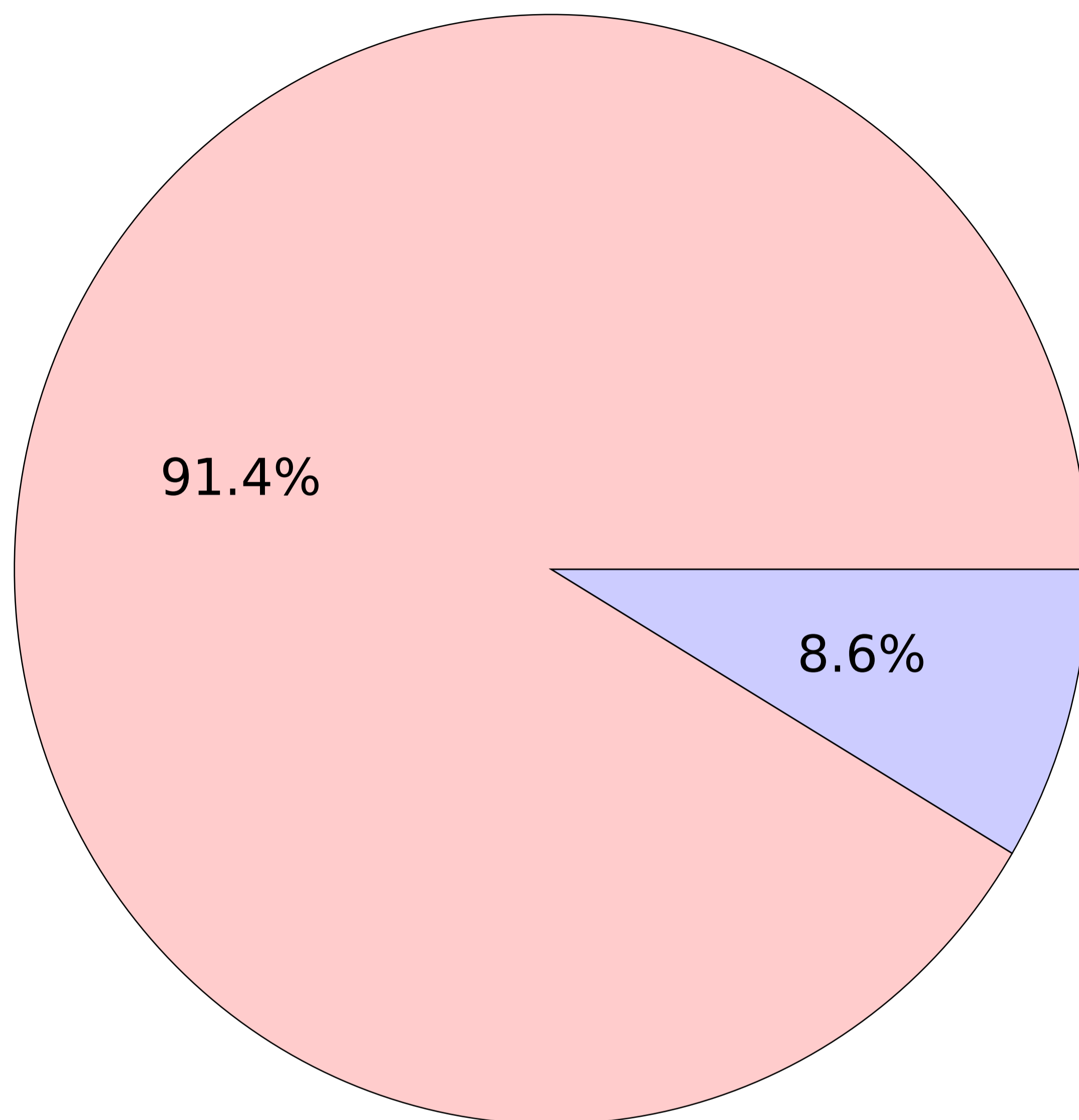

NHEJ  
(1101 reads)

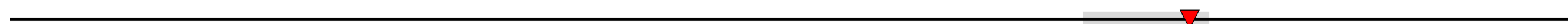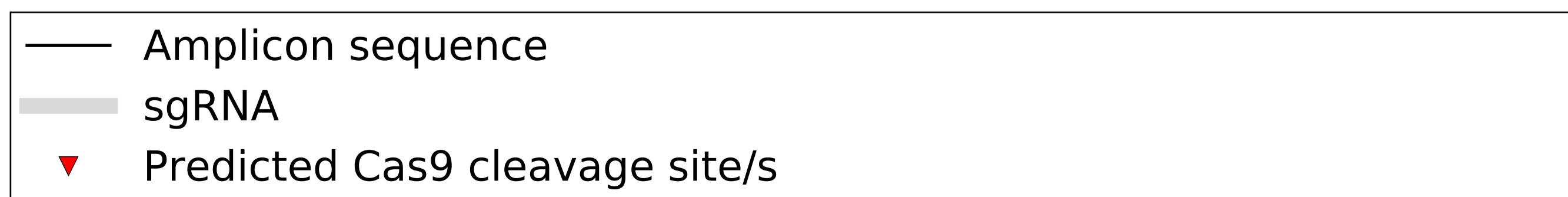

Supplement: Supplementary file 14 — Additional file 14. CRISPResso NHEJ pie charts. [file 12896_2019_565_MOESM14_ESM.zip › CRISPResso_EPSPS-4AL-gRNA4-rep1.pdf]

Unmodified  
(7863 reads)

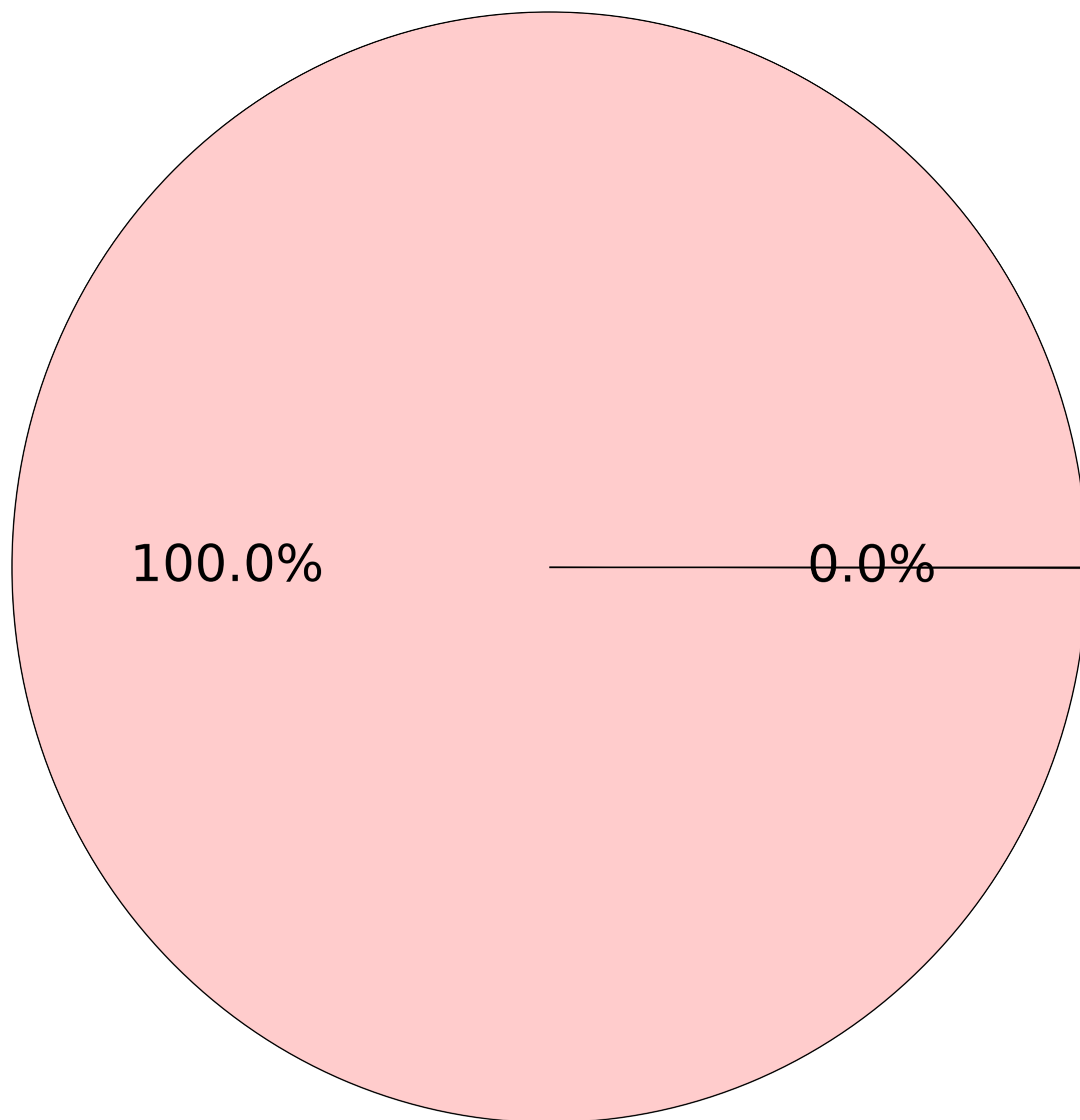

NHEJ  
(2 reads)

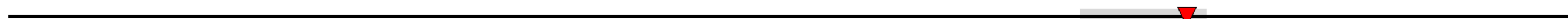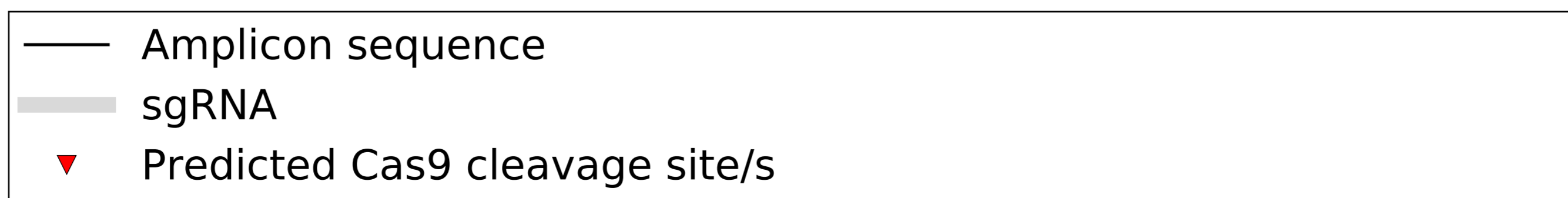

Supplement: Supplementary file 14 — Additional file 14. CRISPResso NHEJ pie charts. [file 12896_2019_565_MOESM14_ESM.zip › CRISPResso_EPSPS-4AL-gRNA4-rep1-negative.pdf]

Unmodified  
(16768 reads)

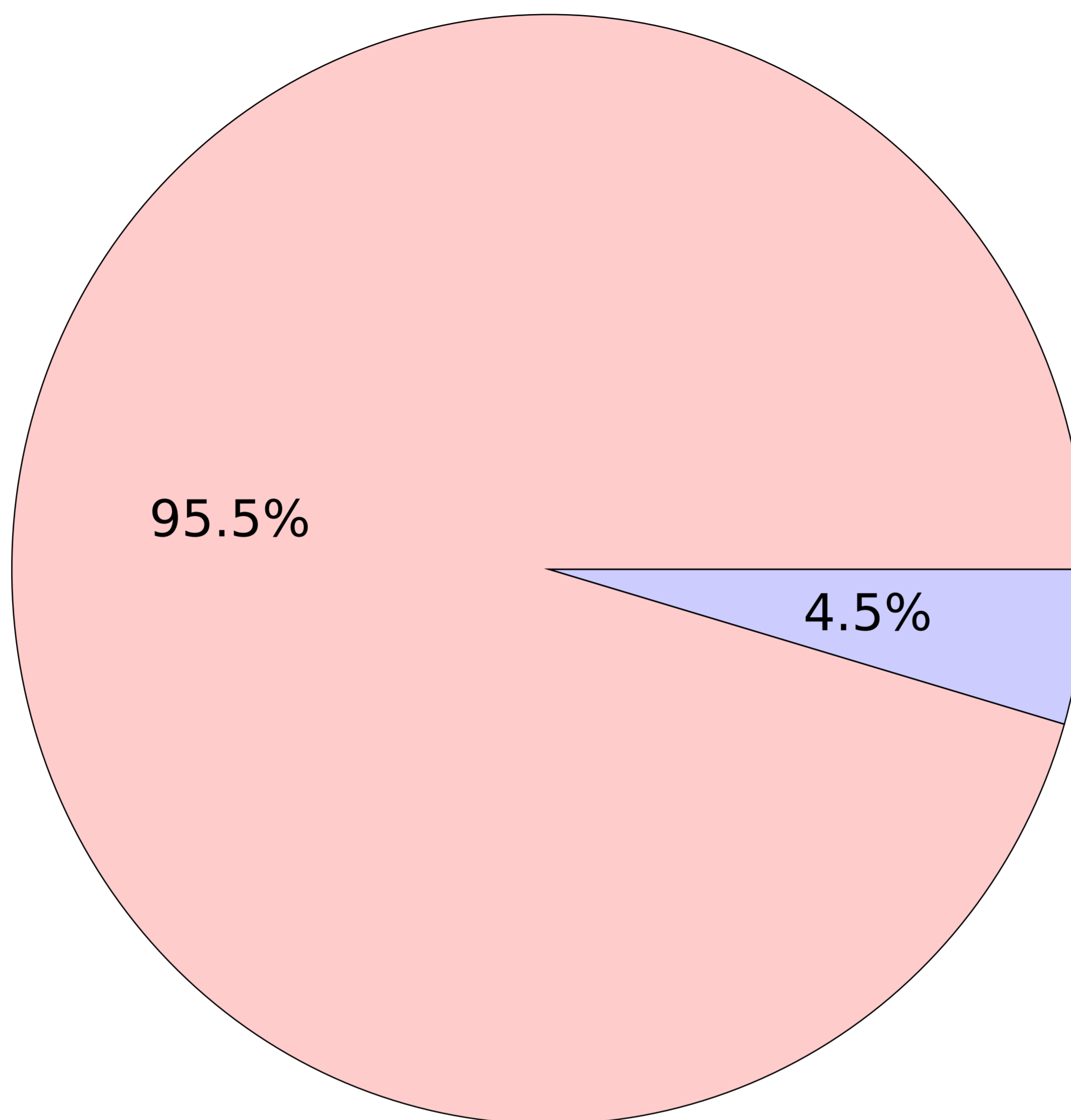

NHEJ  
(791 reads)

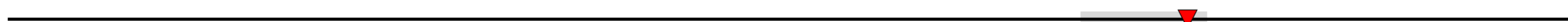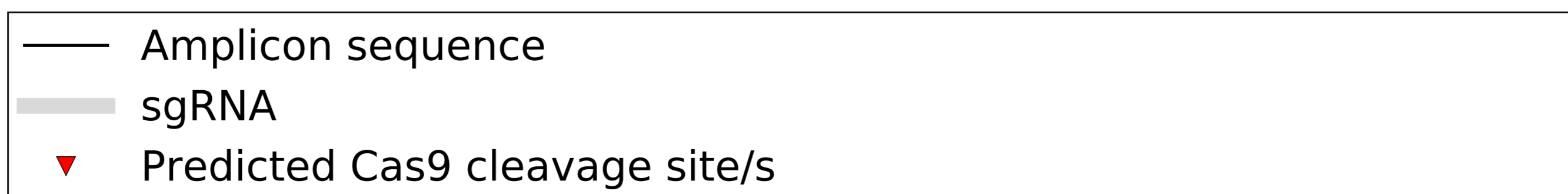

Supplement: Supplementary file 14 — Additional file 14. CRISPResso NHEJ pie charts. [file 12896_2019_565_MOESM14_ESM.zip › CRISPResso_EPSPS-4AL-gRNA4-rep2.pdf]

Unmodified  
(8110 reads)

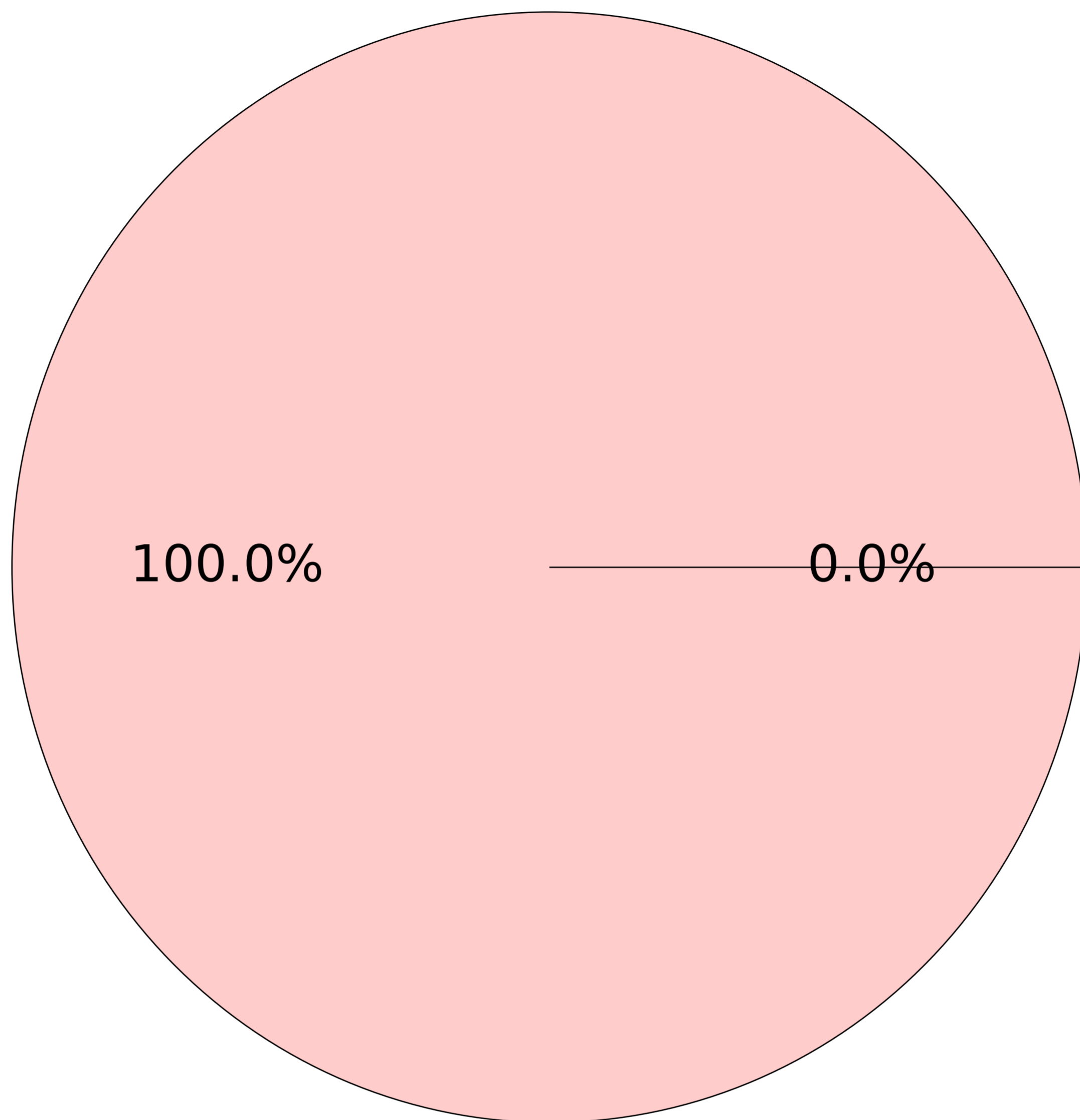

NHEJ  
(0 reads)

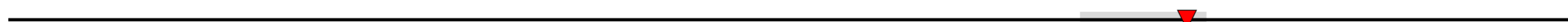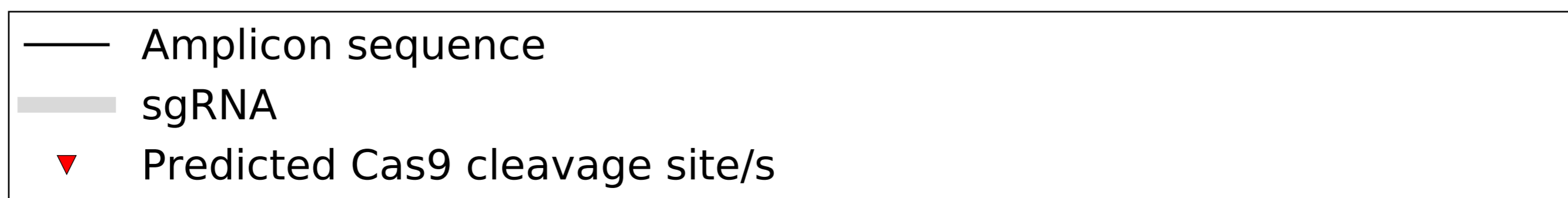

Supplement: Supplementary file 14 — Additional file 14. CRISPResso NHEJ pie charts. [file 12896_2019_565_MOESM14_ESM.zip › CRISPResso_EPSPS-4AL-gRNA4-rep2-negative.pdf]

Unmodified  
(12085 reads)

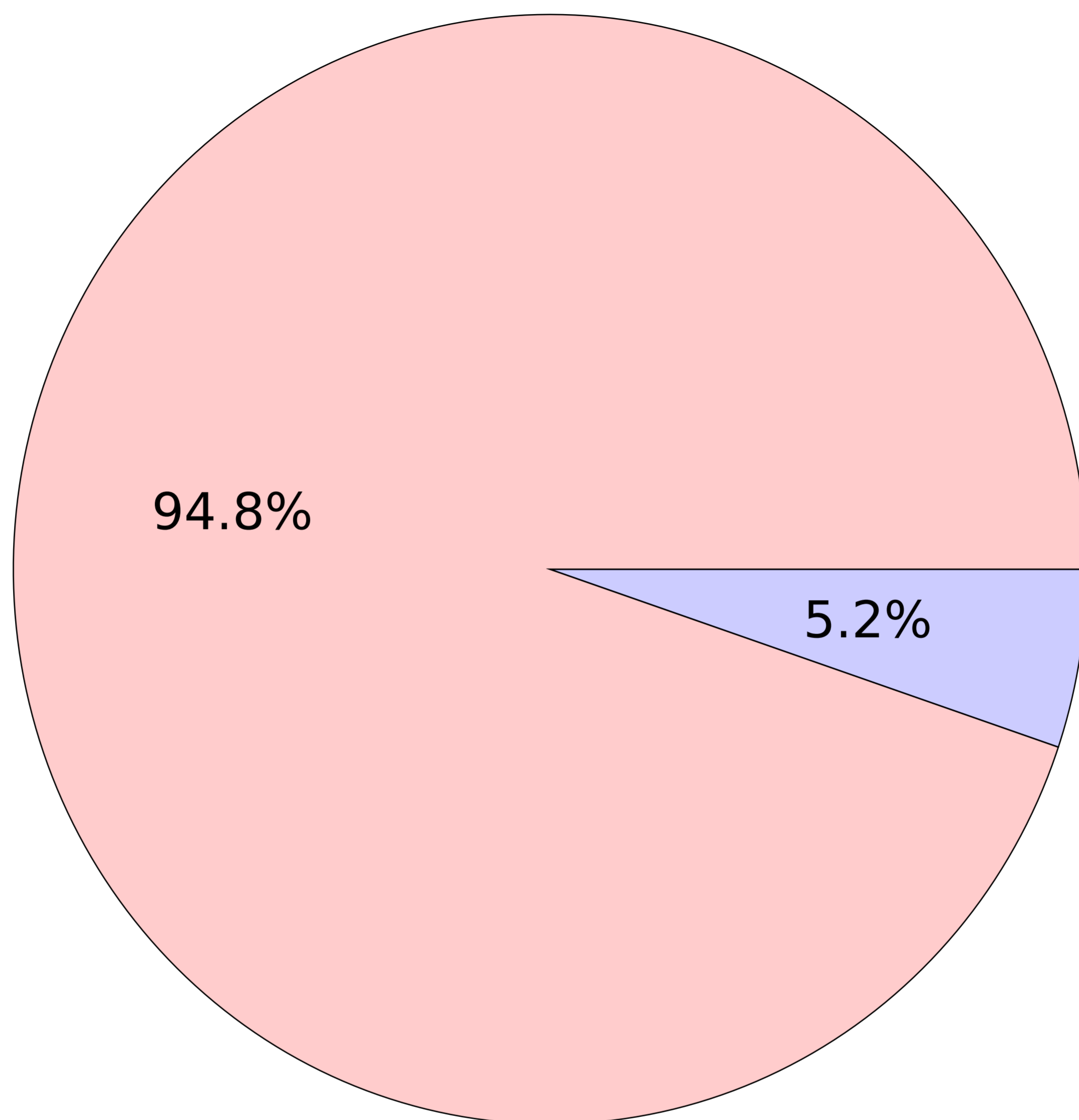

NHEJ  
(662 reads)

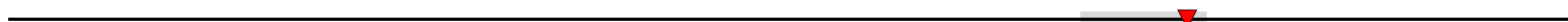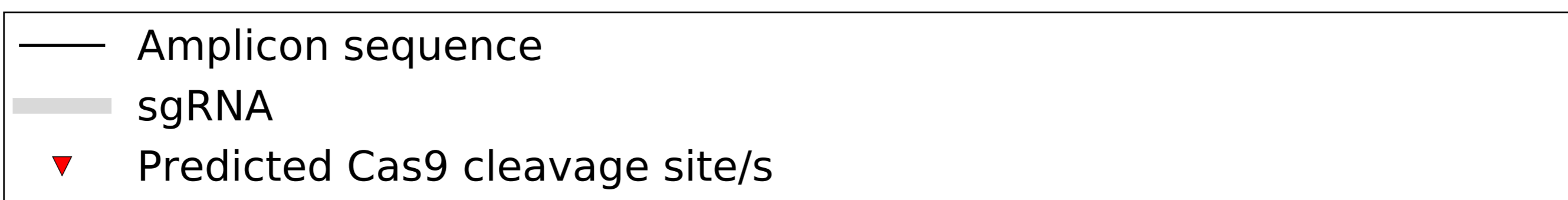

Supplement: Supplementary file 14 — Additional file 14. CRISPResso NHEJ pie charts. [file 12896_2019_565_MOESM14_ESM.zip › CRISPResso_EPSPS-4AL-gRNA4-rep3.pdf]

Unmodified  
(14200 reads)

100.0%

0.0%

NHEJ  
(2 reads)

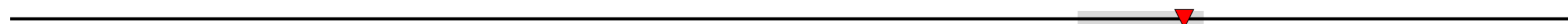

- Amplicon sequence
- sgRNA
- ▼ Predicted Cas9 cleavage site/s

Supplement: Supplementary file 14 — Additional file 14. CRISPResso NHEJ pie charts. [file 12896_2019_565_MOESM14_ESM.zip › CRISPResso_EPSPS-4AL-gRNA4-rep3-negative.pdf]

Unmodified  
(13688 reads)

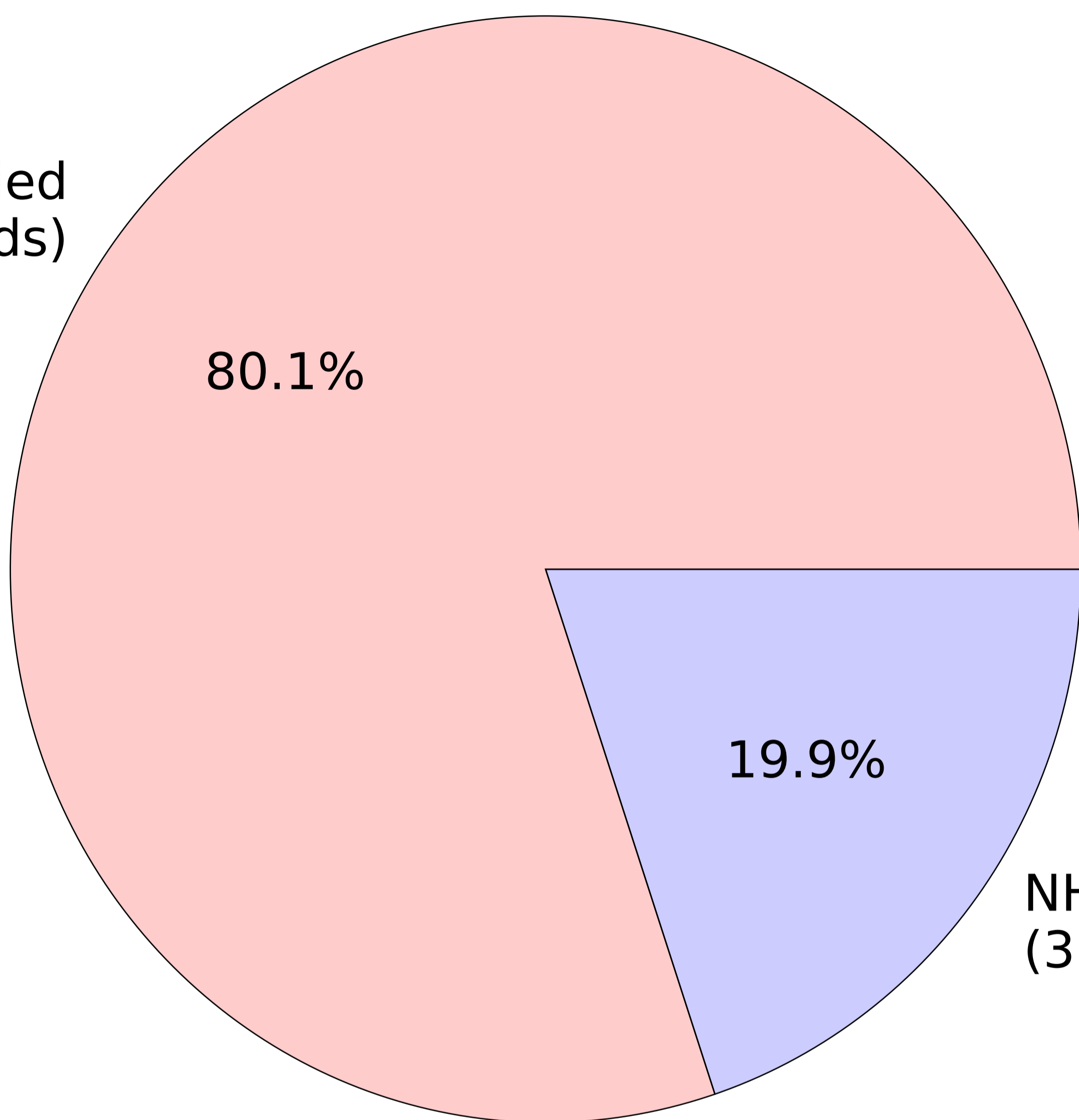

NHEJ  
(3396 reads)

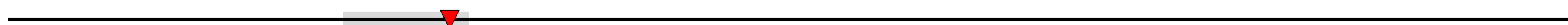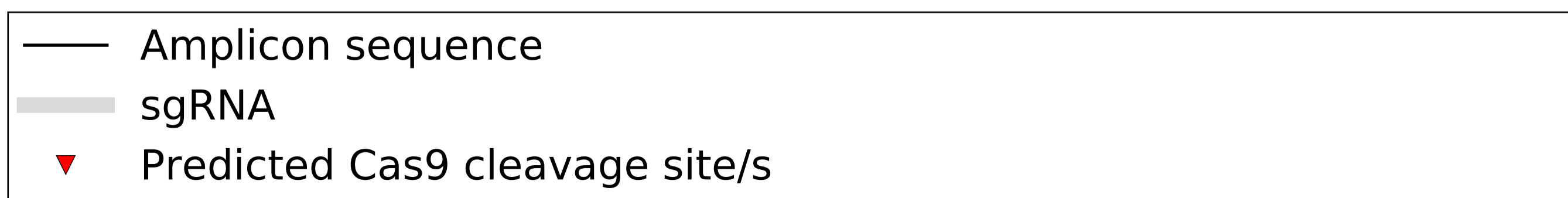

Supplement: Supplementary file 14 — Additional file 14. CRISPResso NHEJ pie charts. [file 12896_2019_565_MOESM14_ESM.zip › CRISPResso_EPSPS-4AL-gRNA5-rep1.pdf]

Unmodified  
(7863 reads)

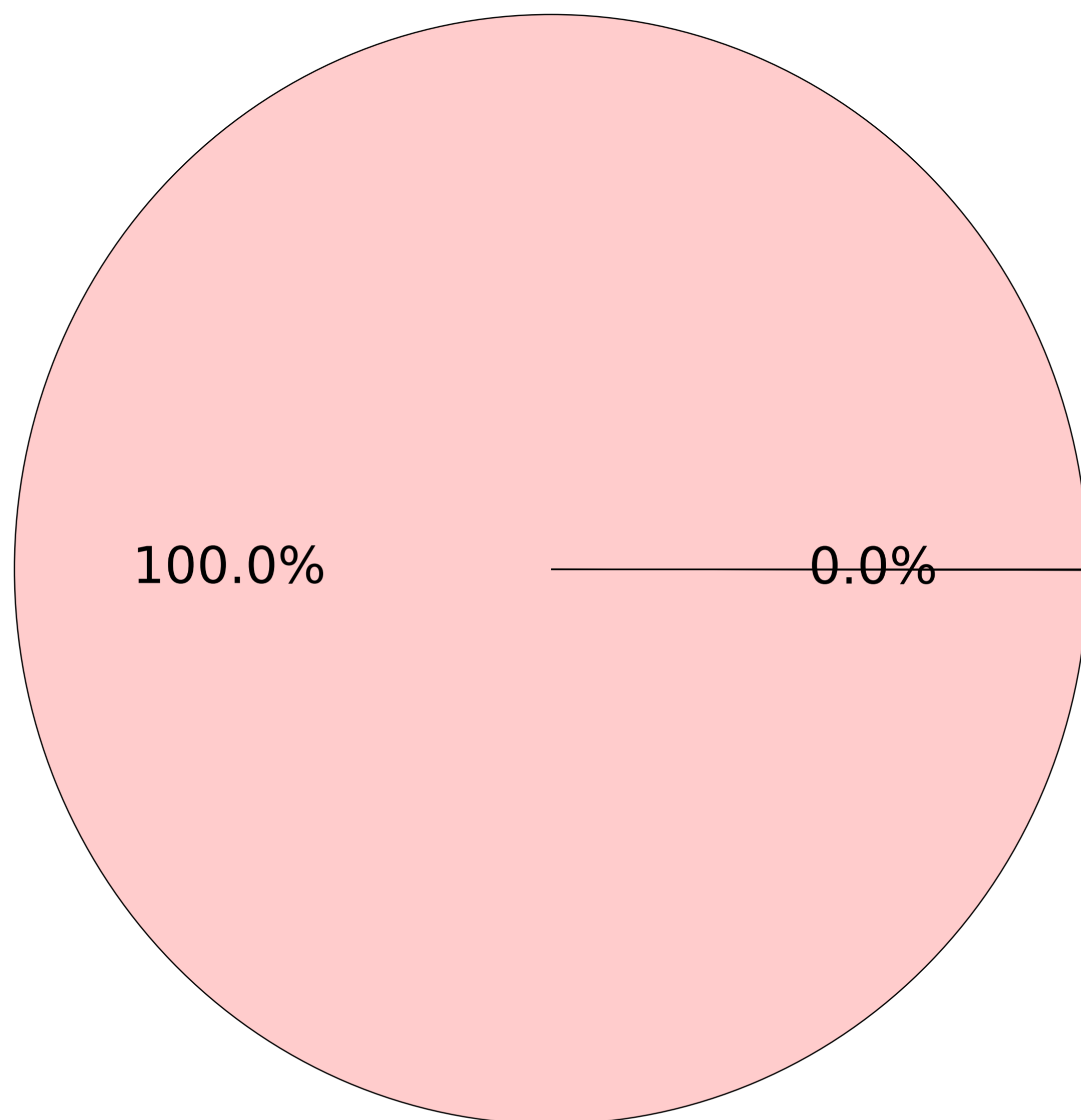

NHEJ  
(2 reads)

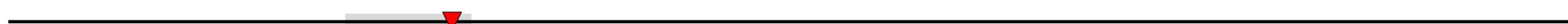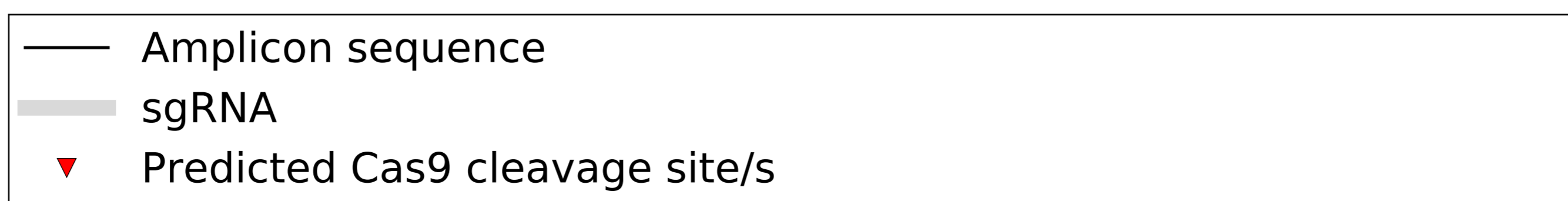

Supplement: Supplementary file 14 — Additional file 14. CRISPResso NHEJ pie charts. [file 12896_2019_565_MOESM14_ESM.zip › CRISPResso_EPSPS-4AL-gRNA5-rep1-negative.pdf]

Unmodified  
(12243 reads)

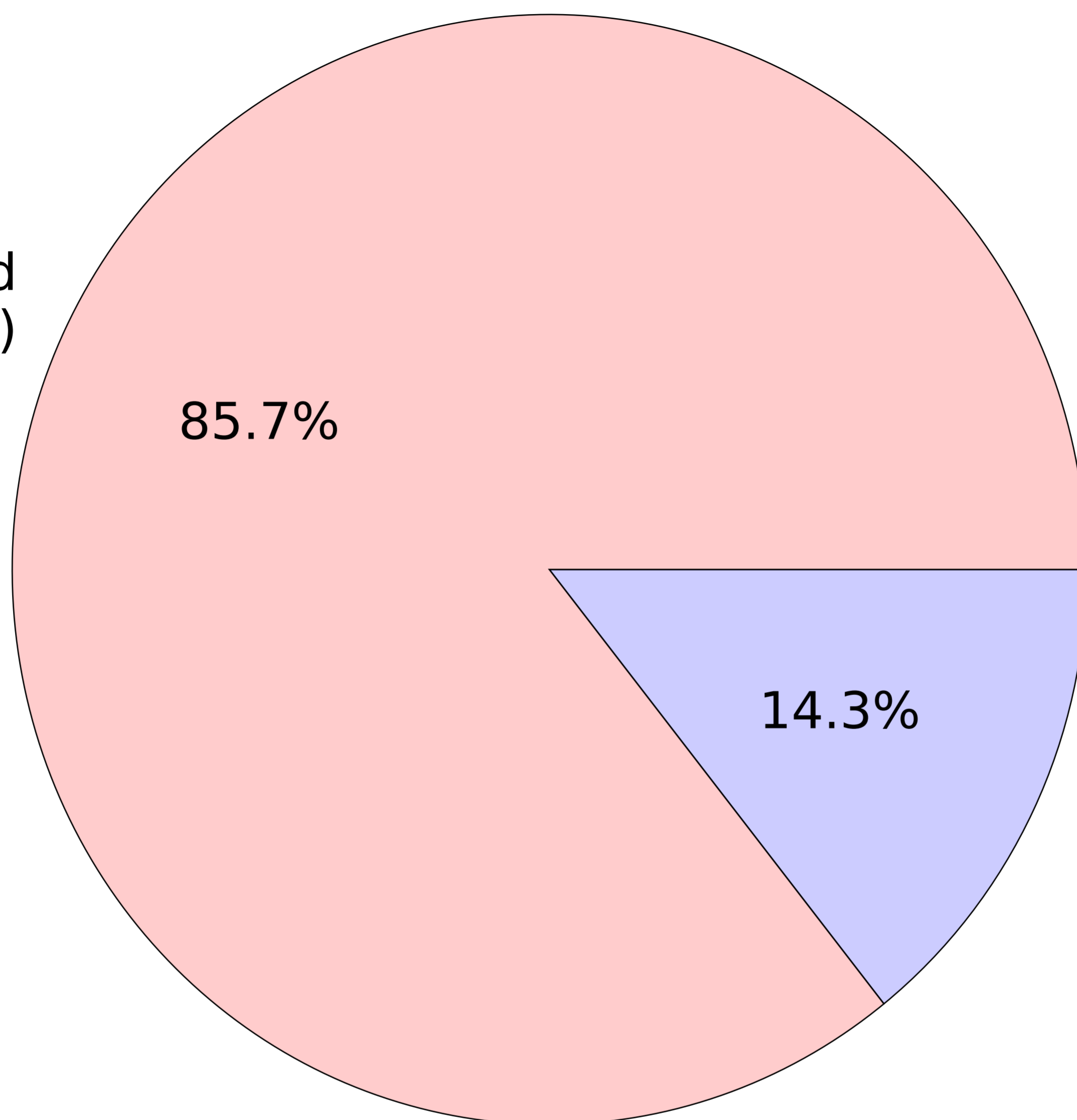

NHEJ  
(2043 reads)

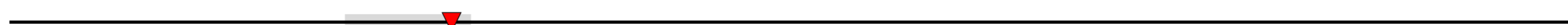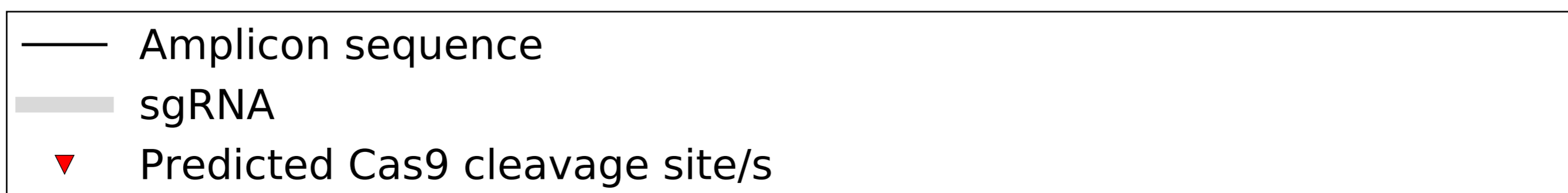

Supplement: Supplementary file 14 — Additional file 14. CRISPResso NHEJ pie charts. [file 12896_2019_565_MOESM14_ESM.zip › CRISPResso_EPSPS-4AL-gRNA5-rep2.pdf]

Unmodified  
(8107 reads)

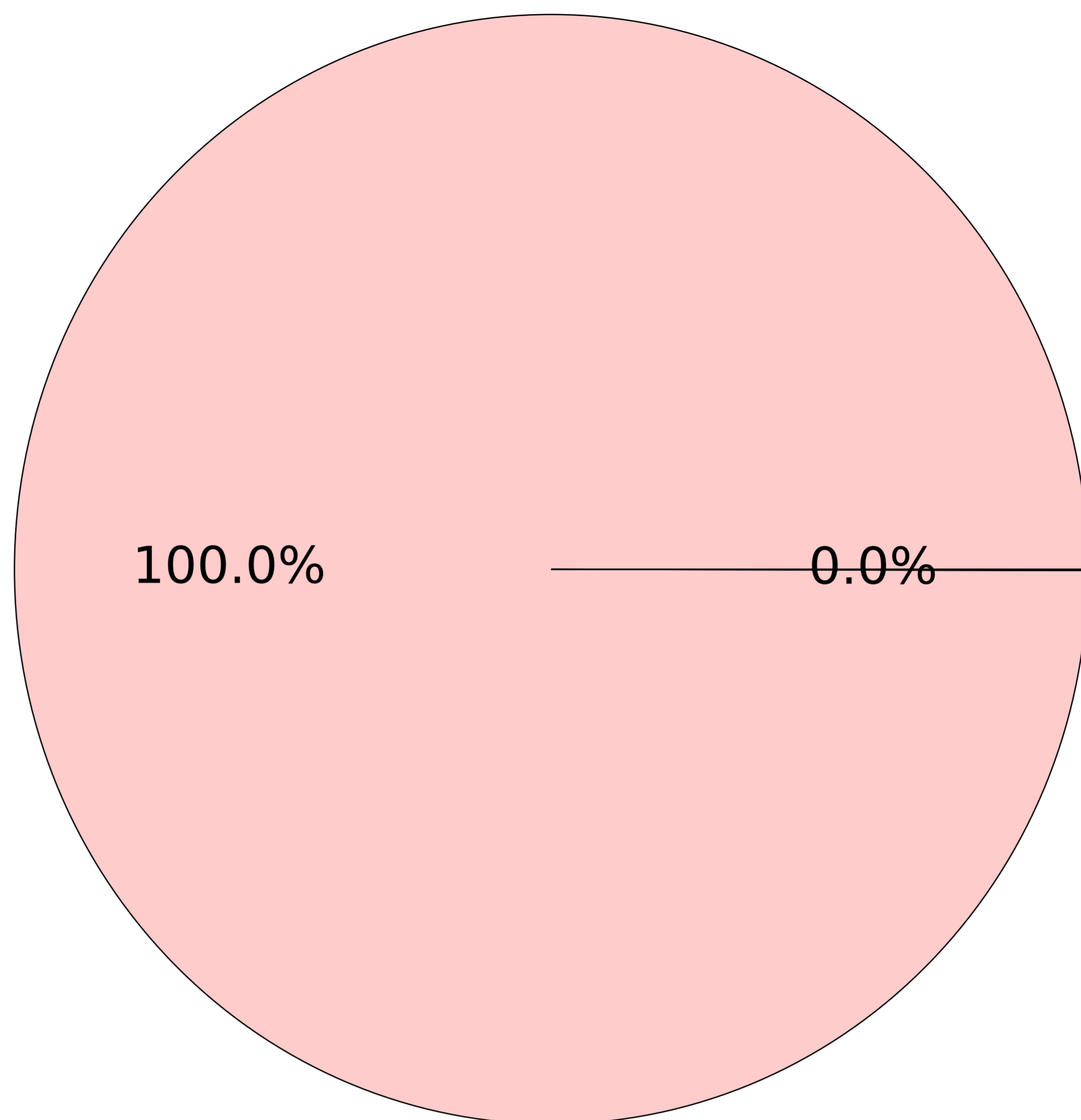

NHEJ  
(3 reads)

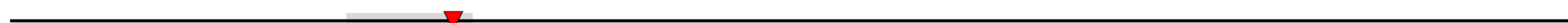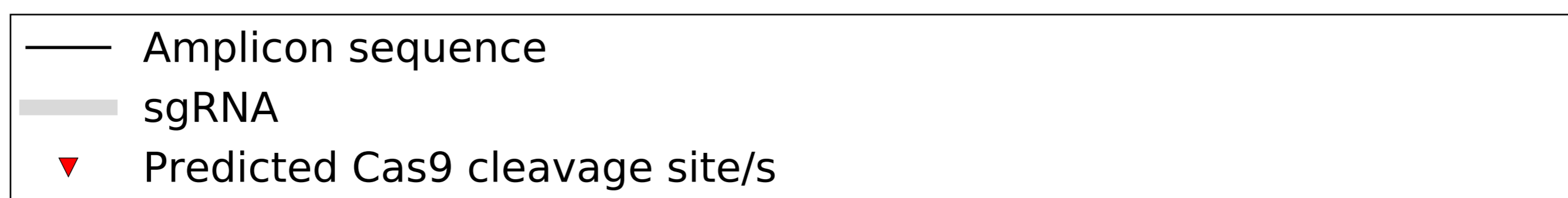

Supplement: Supplementary file 14 — Additional file 14. CRISPResso NHEJ pie charts. [file 12896_2019_565_MOESM14_ESM.zip › CRISPResso_EPSPS-4AL-gRNA5-rep2-negative.pdf]

Unmodified  
(9843 reads)

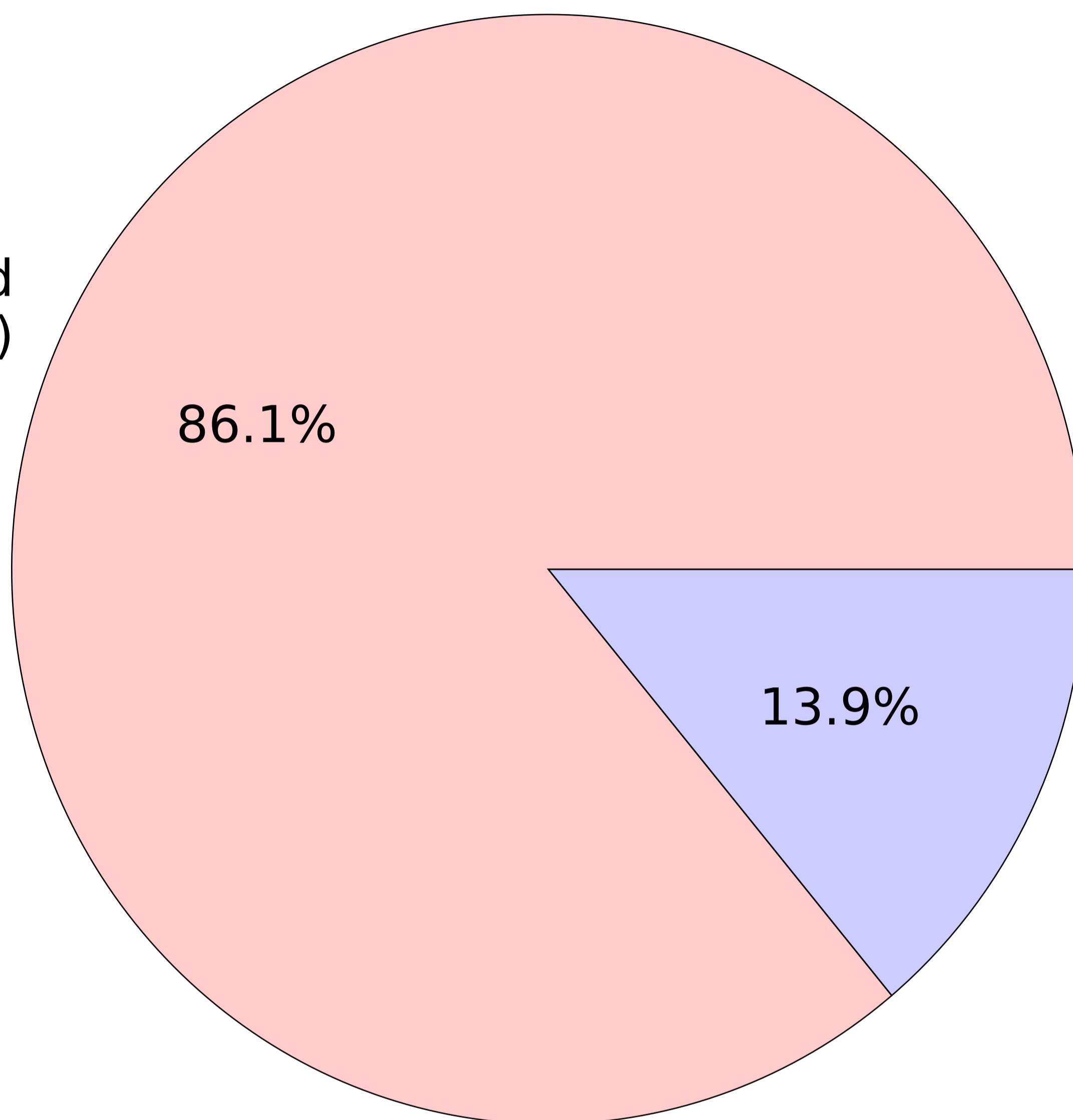

NHEJ  
(1595 reads)

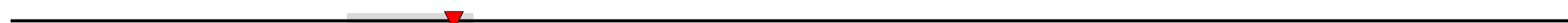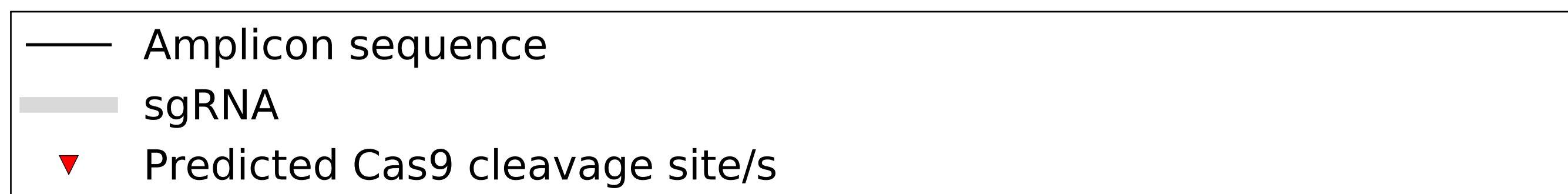

Supplement: Supplementary file 14 — Additional file 14. CRISPResso NHEJ pie charts. [file 12896_2019_565_MOESM14_ESM.zip › CRISPResso_EPSPS-4AL-gRNA5-rep3.pdf]

Unmodified  
(14200 reads)

100.0%

0.0%

NHEJ  
(2 reads)

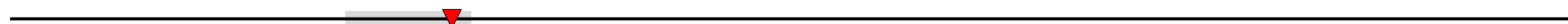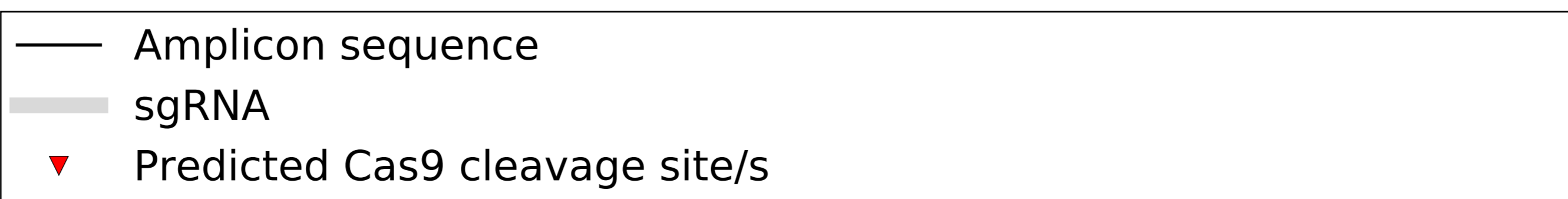

Supplement: Supplementary file 14 — Additional file 14. CRISPResso NHEJ pie charts. [file 12896_2019_565_MOESM14_ESM.zip › CRISPResso_EPSPS-4AL-gRNA5-rep3-negative.pdf]

Unmodified  
(9576 reads)

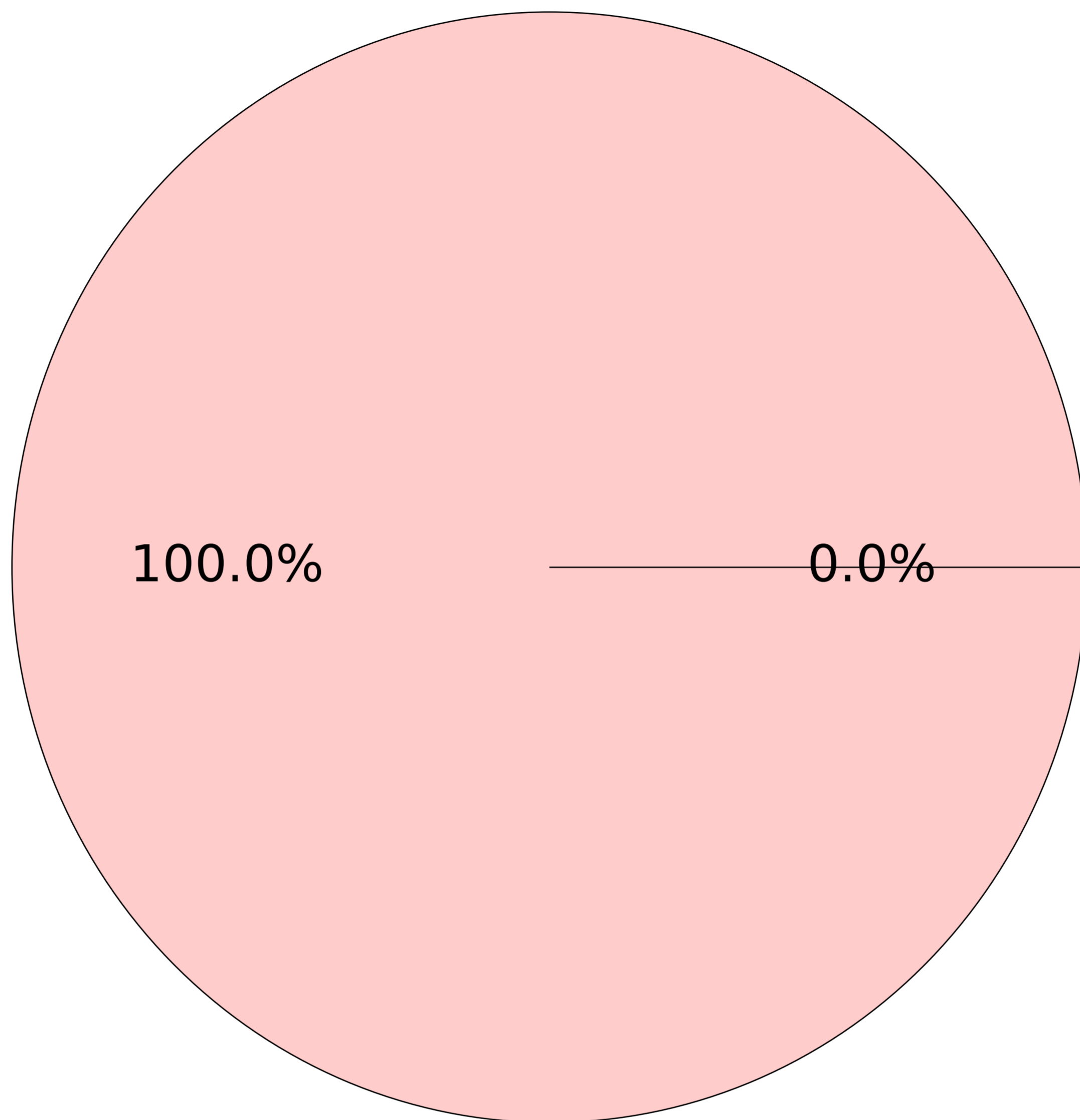

NHEJ  
(0 reads)

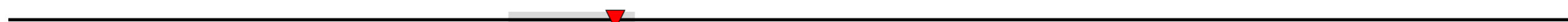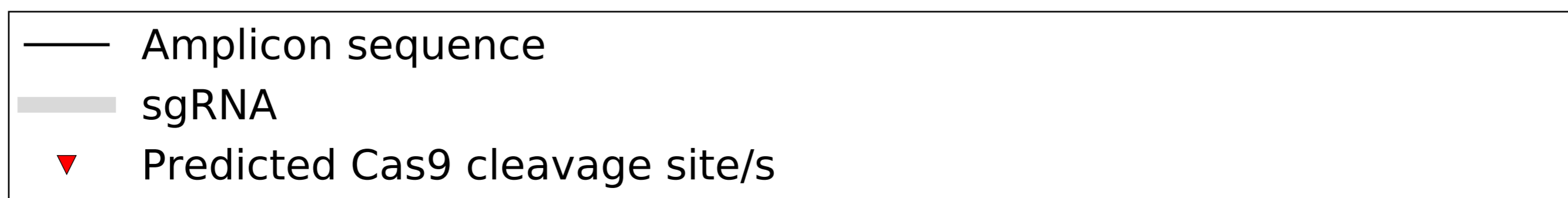

Supplement: Supplementary file 14 — Additional file 14. CRISPResso NHEJ pie charts. [file 12896_2019_565_MOESM14_ESM.zip › CRISPResso_EPSPS-4AL-gRNA6-rep1.pdf]

Unmodified  
(7862 reads)

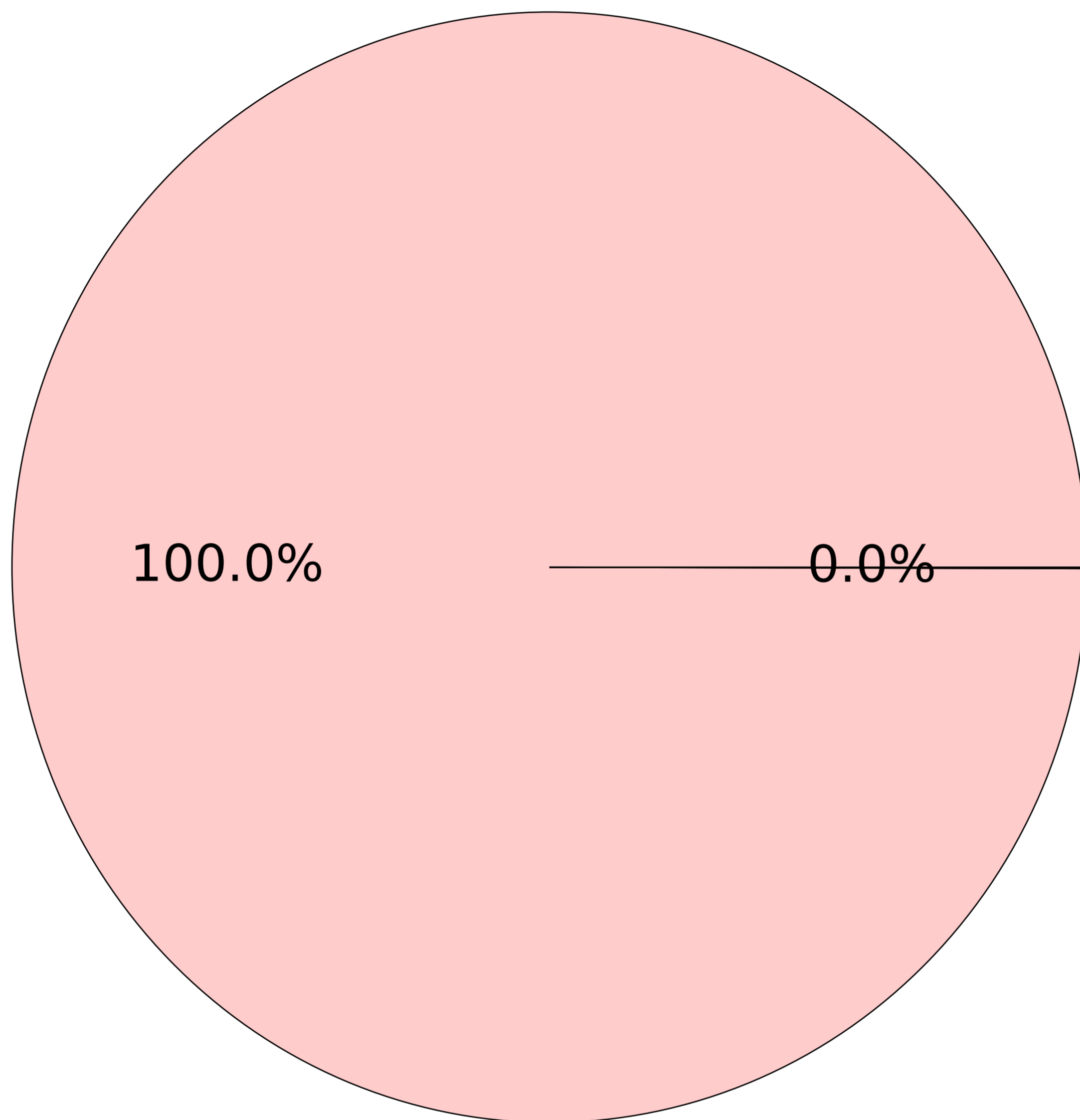

NHEJ  
(3 reads)

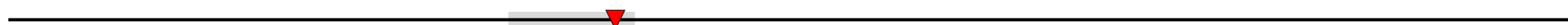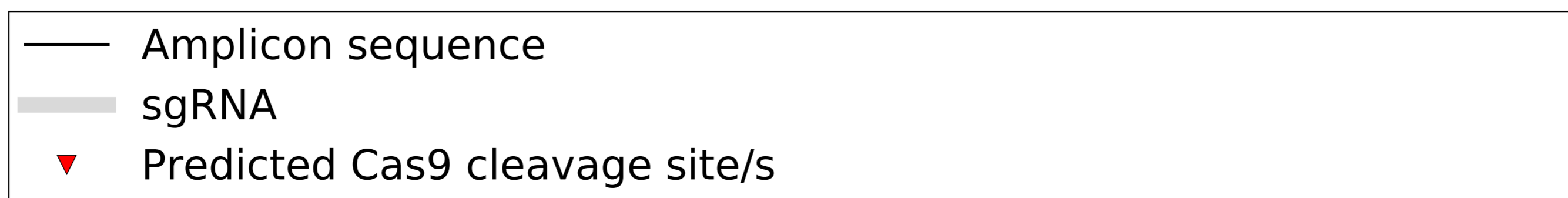

Supplement: Supplementary file 14 — Additional file 14. CRISPResso NHEJ pie charts. [file 12896_2019_565_MOESM14_ESM.zip › CRISPResso_EPSPS-4AL-gRNA6-rep1-negative.pdf]

Unmodified  
(10866 reads)

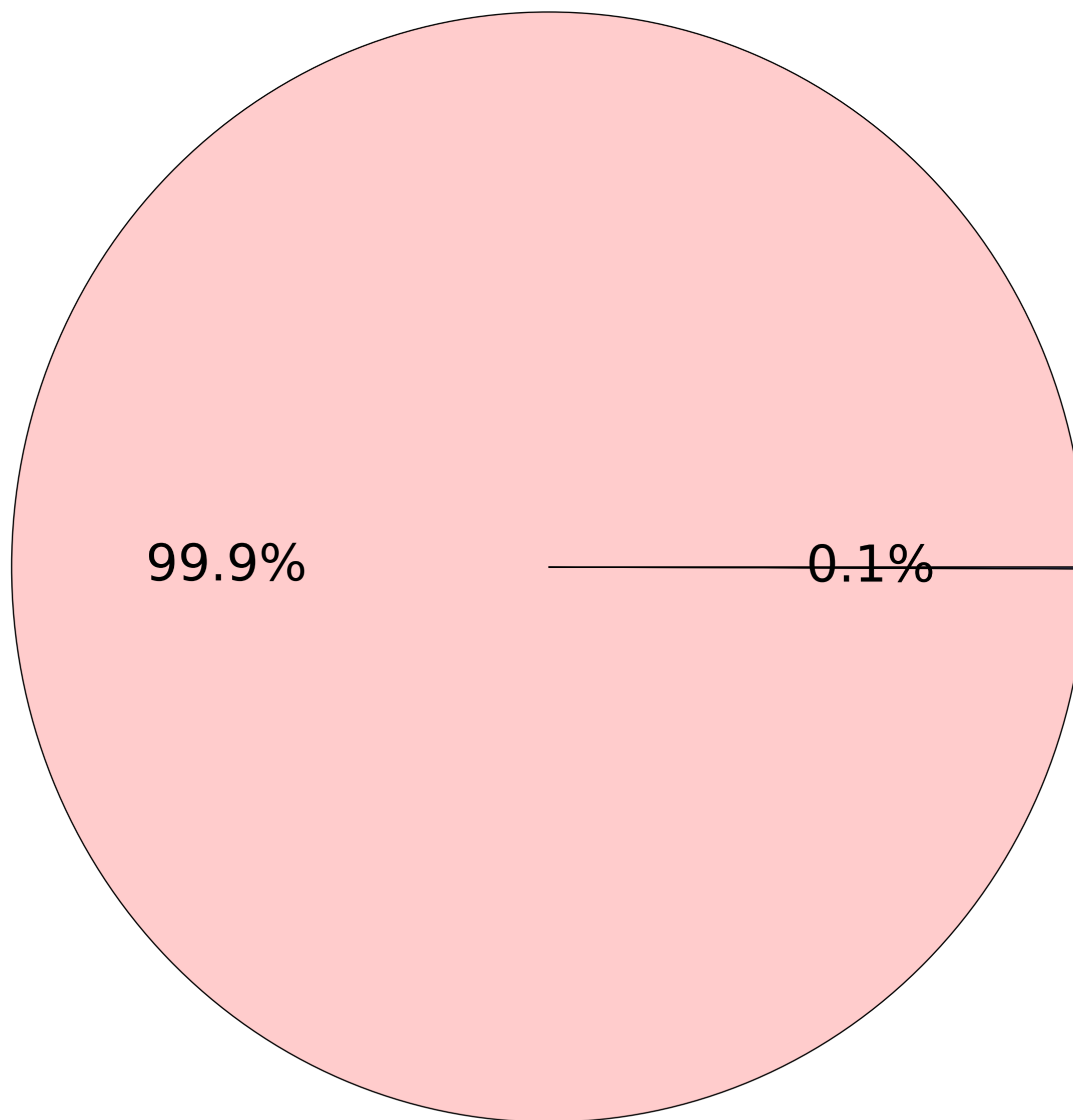

NHEJ  
(6 reads)

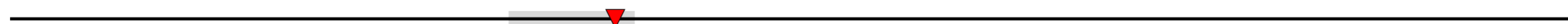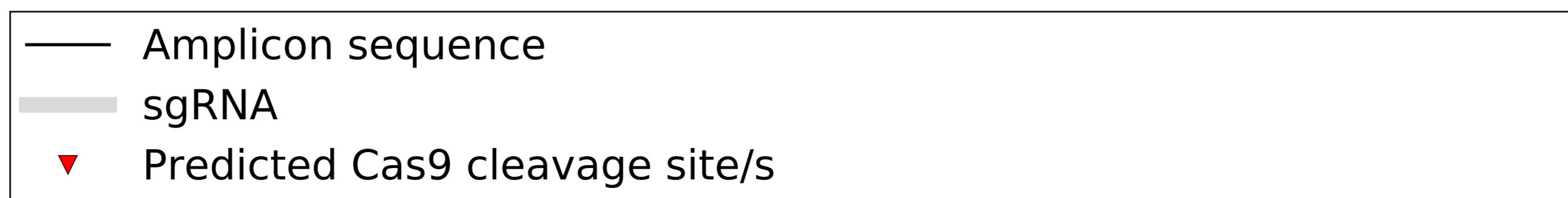

Supplement: Supplementary file 14 — Additional file 14. CRISPResso NHEJ pie charts. [file 12896_2019_565_MOESM14_ESM.zip › CRISPResso_EPSPS-4AL-gRNA6-rep2.pdf]

Unmodified  
(8109 reads)

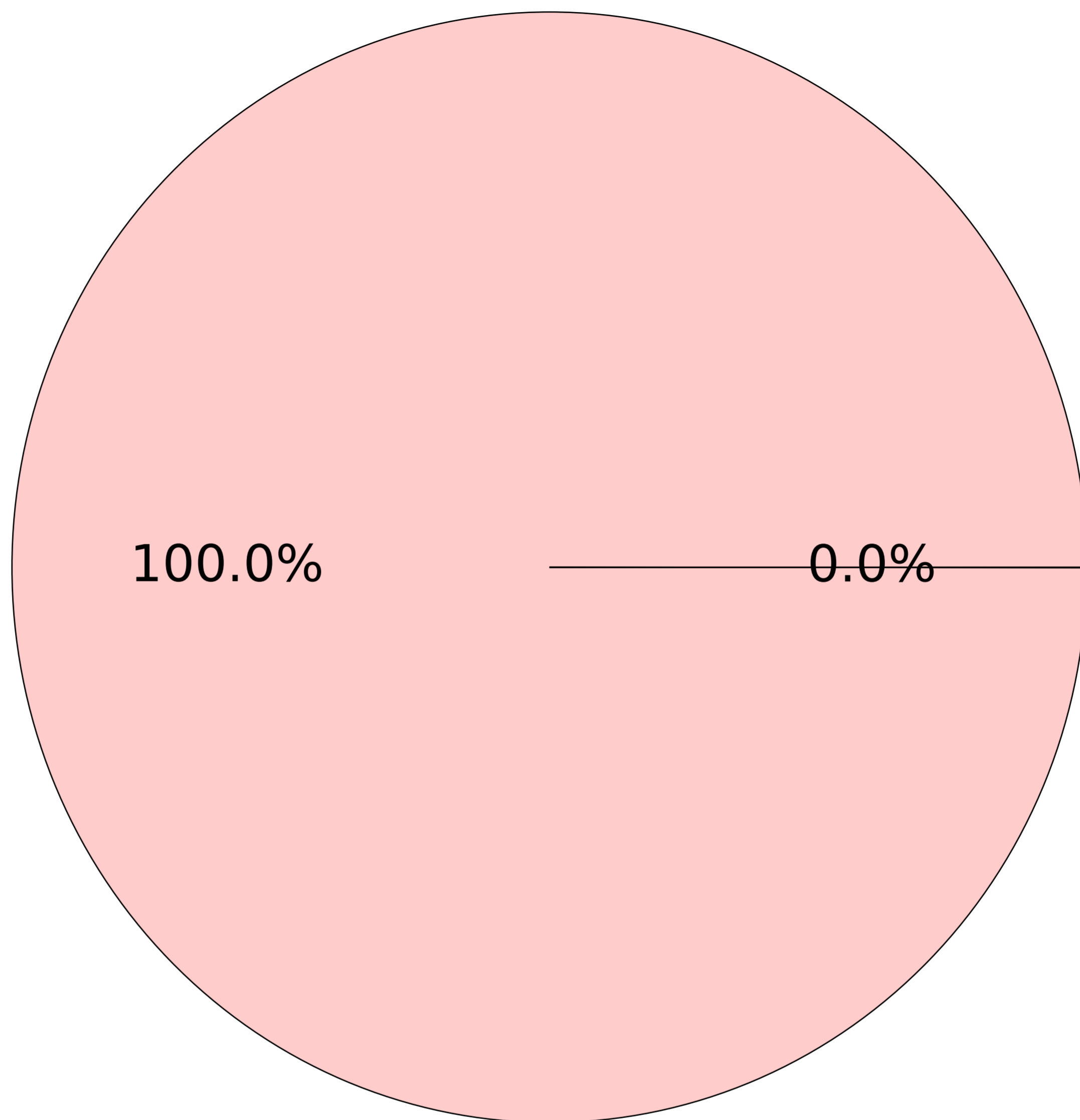

NHEJ  
(1 reads)

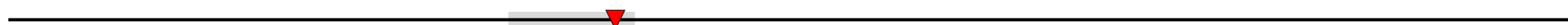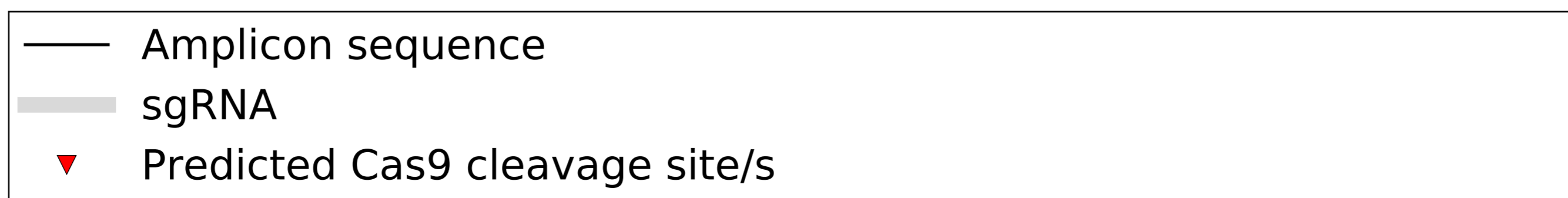

Supplement: Supplementary file 14 — Additional file 14. CRISPResso NHEJ pie charts. [file 12896_2019_565_MOESM14_ESM.zip › CRISPResso_EPSPS-4AL-gRNA6-rep2-negative.pdf]

Unmodified  
(8523 reads)

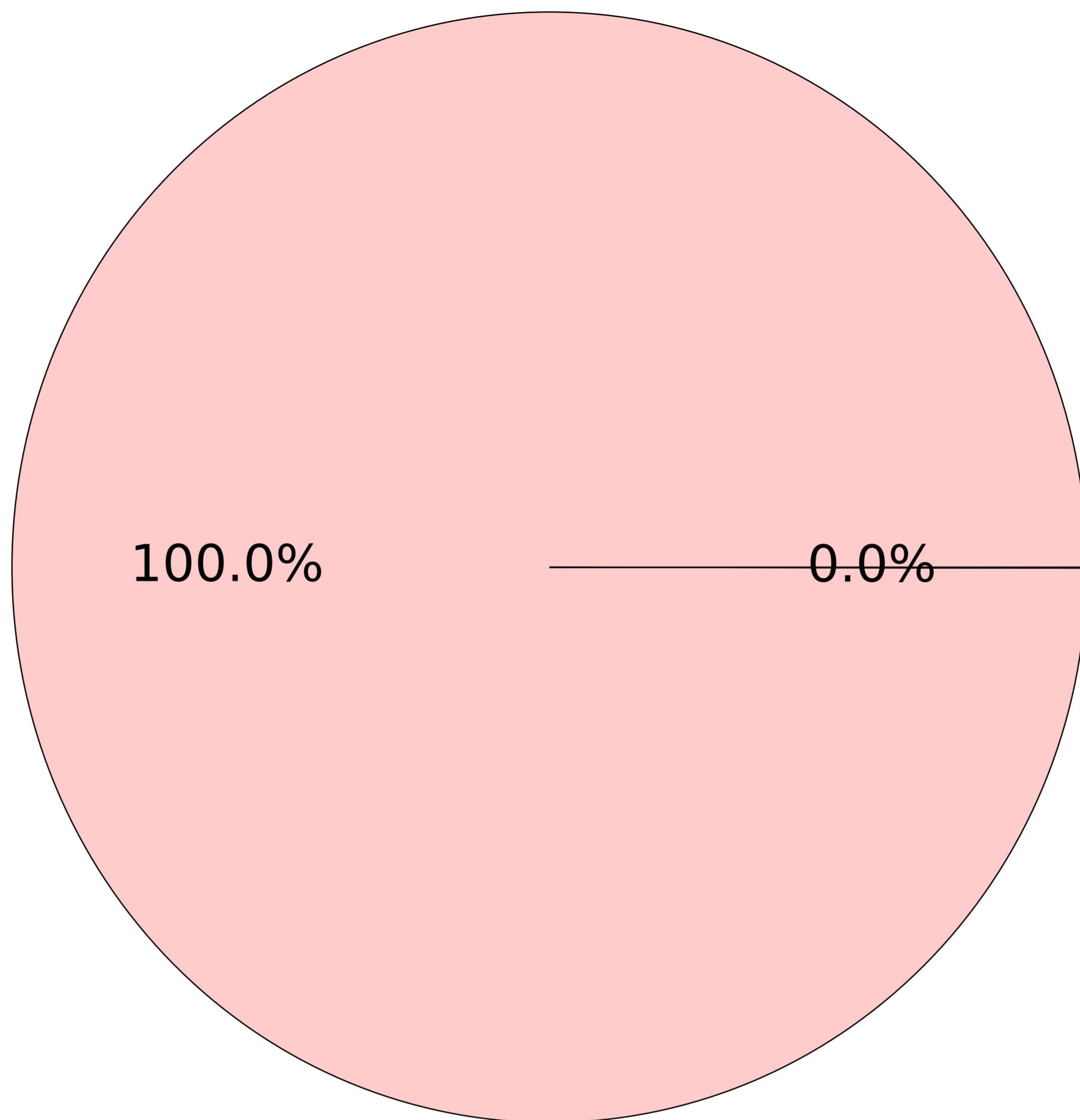

NHEJ  
(2 reads)

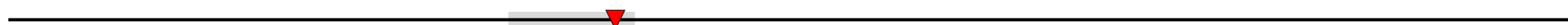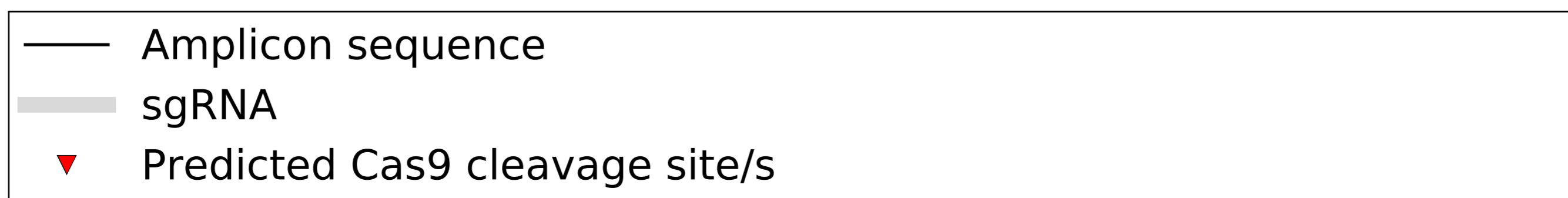

Supplement: Supplementary file 14 — Additional file 14. CRISPResso NHEJ pie charts. [file 12896_2019_565_MOESM14_ESM.zip › CRISPResso_EPSPS-4AL-gRNA6-rep3.pdf]

Unmodified  
(14198 reads)

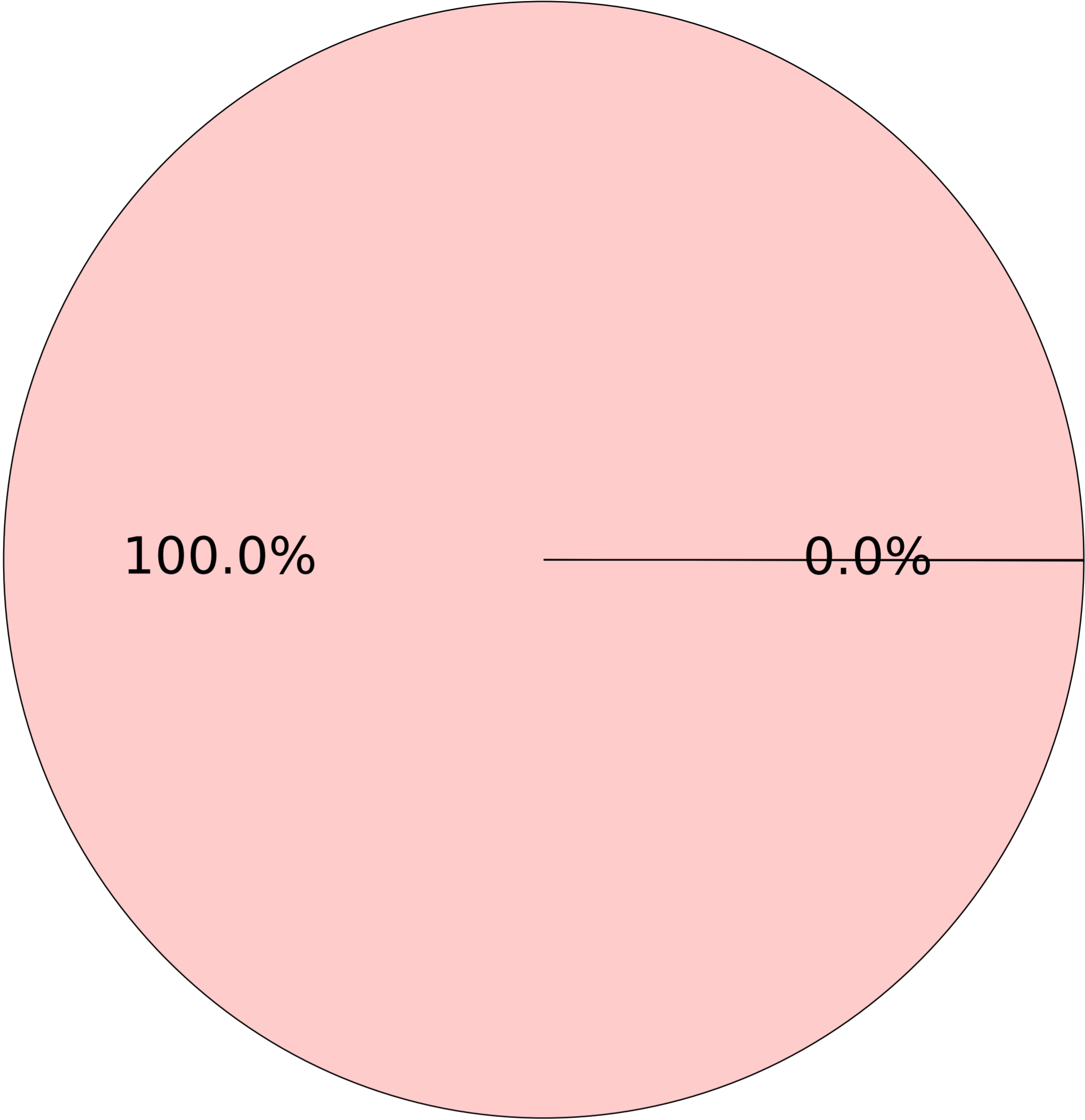

NHEJ  
(4 reads)

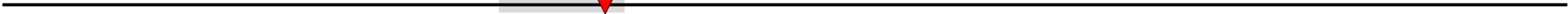

—

Amplicon sequence

—

sgRNA

▼

Predicted Cas9 cleavage site/s

Supplement: Supplementary file 14 — Additional file 14. CRISPResso NHEJ pie charts. [file 12896_2019_565_MOESM14_ESM.zip › CRISPResso_EPSPS-4AL-gRNA6-rep3-negative.pdf]

Unmodified  
(13462 reads)

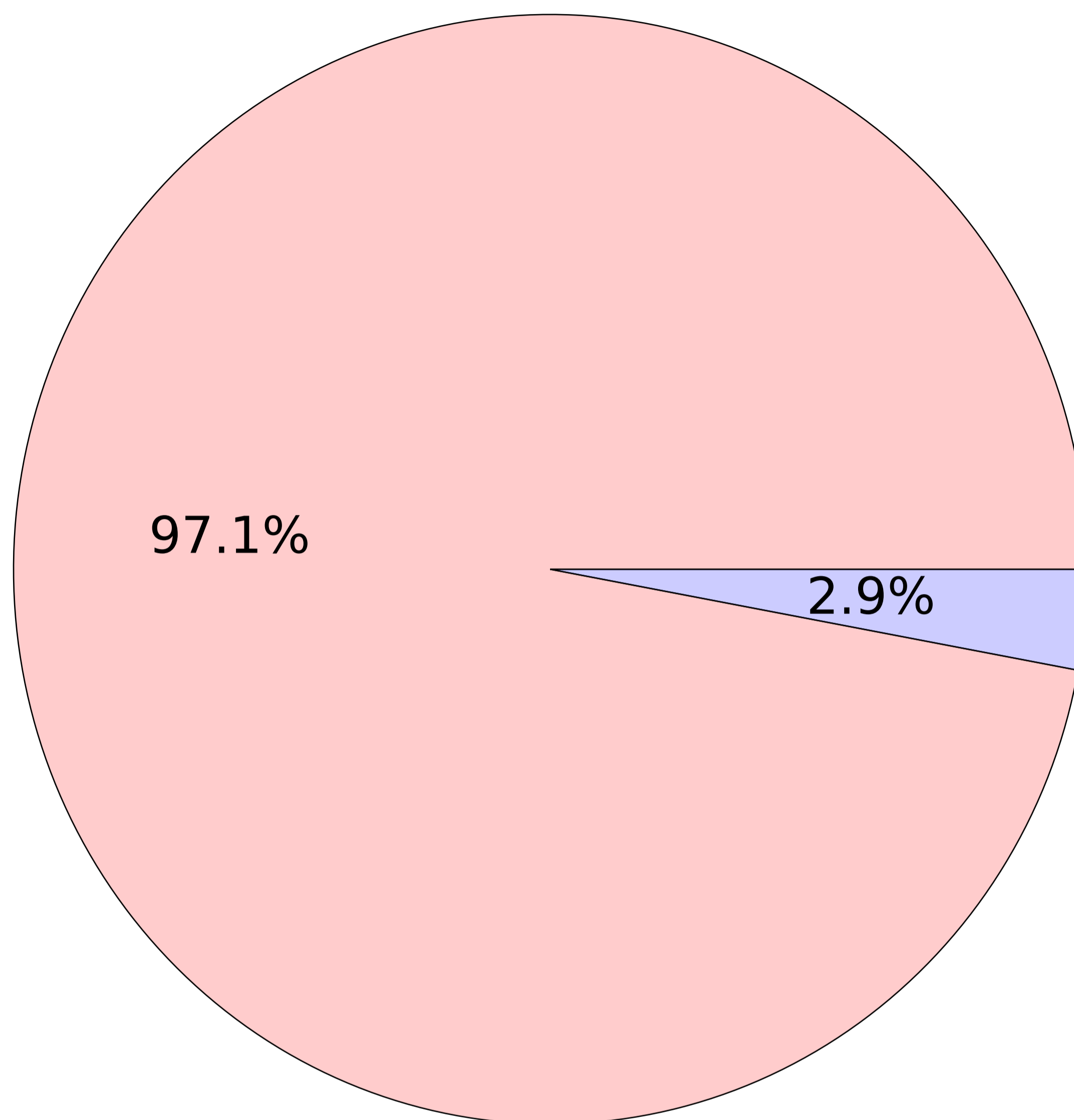

NHEJ  
(404 reads)

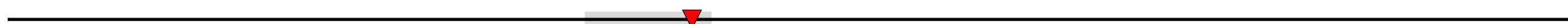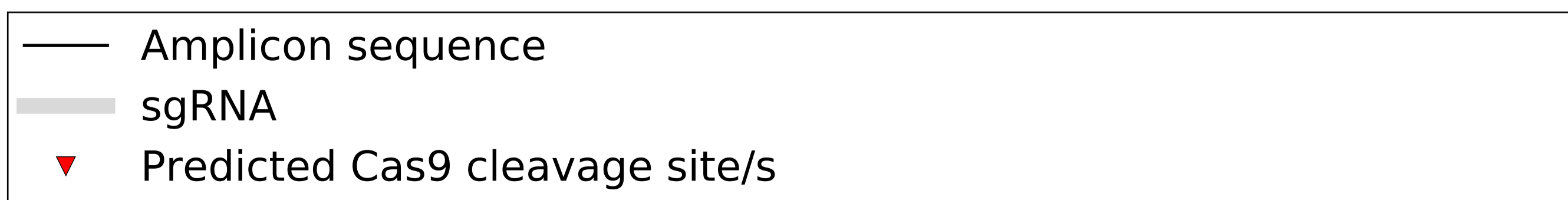

Supplement: Supplementary file 14 — Additional file 14. CRISPResso NHEJ pie charts. [file 12896_2019_565_MOESM14_ESM.zip › CRISPResso_EPSPS-4AL-gRNA7-rep1.pdf]

Unmodified  
(7865 reads)

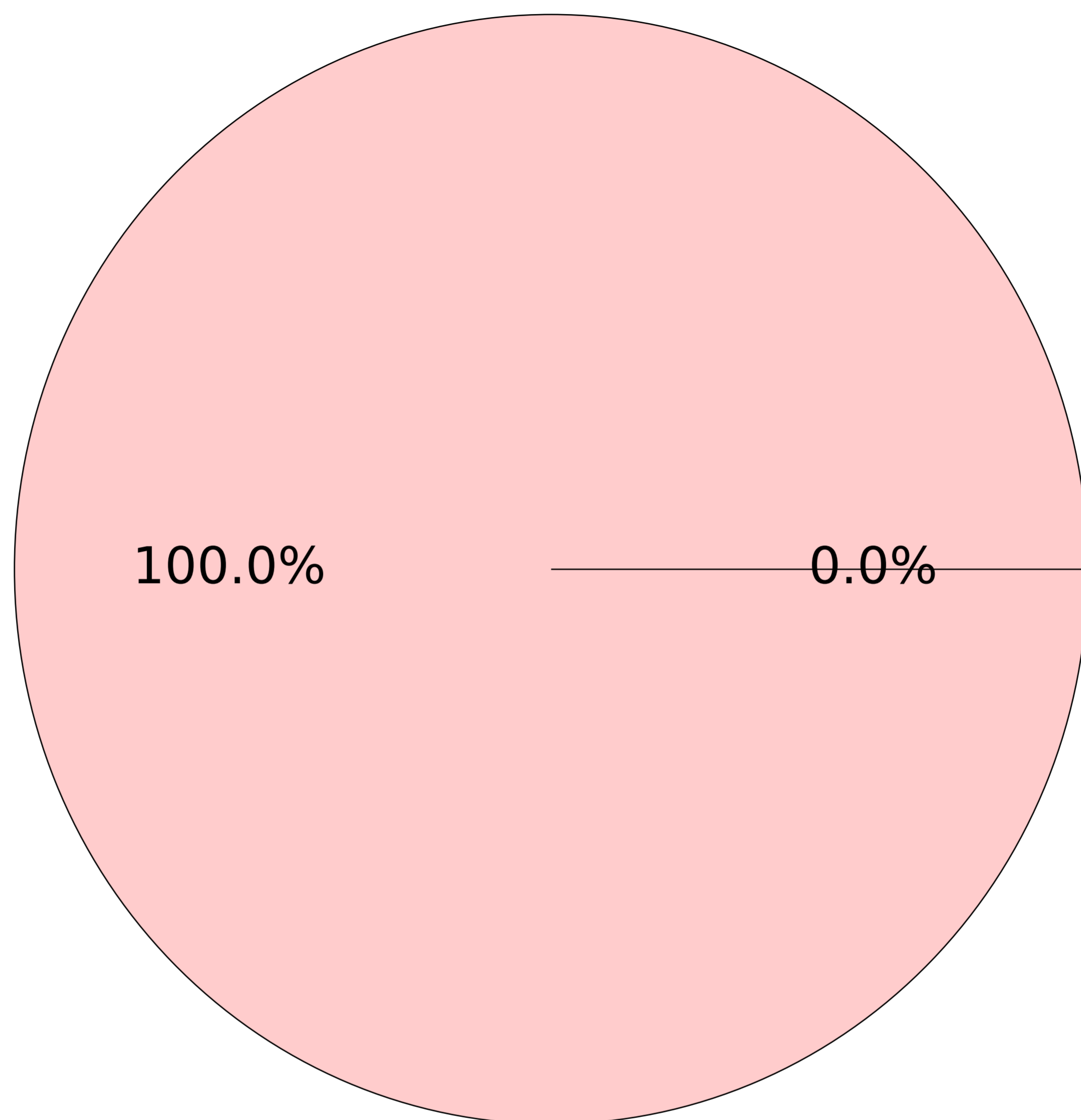

NHEJ  
(0 reads)

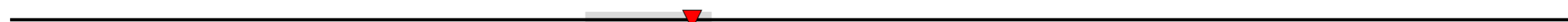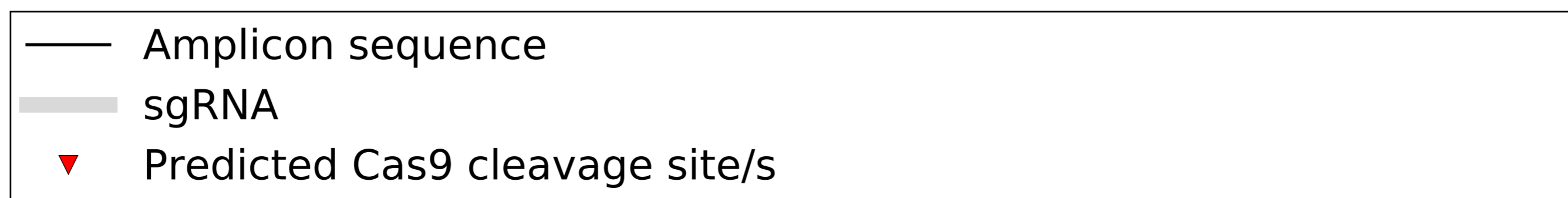

Supplement: Supplementary file 14 — Additional file 14. CRISPResso NHEJ pie charts. [file 12896_2019_565_MOESM14_ESM.zip › CRISPResso_EPSPS-4AL-gRNA7-rep1-negative.pdf]

Unmodified  
(12829 reads)

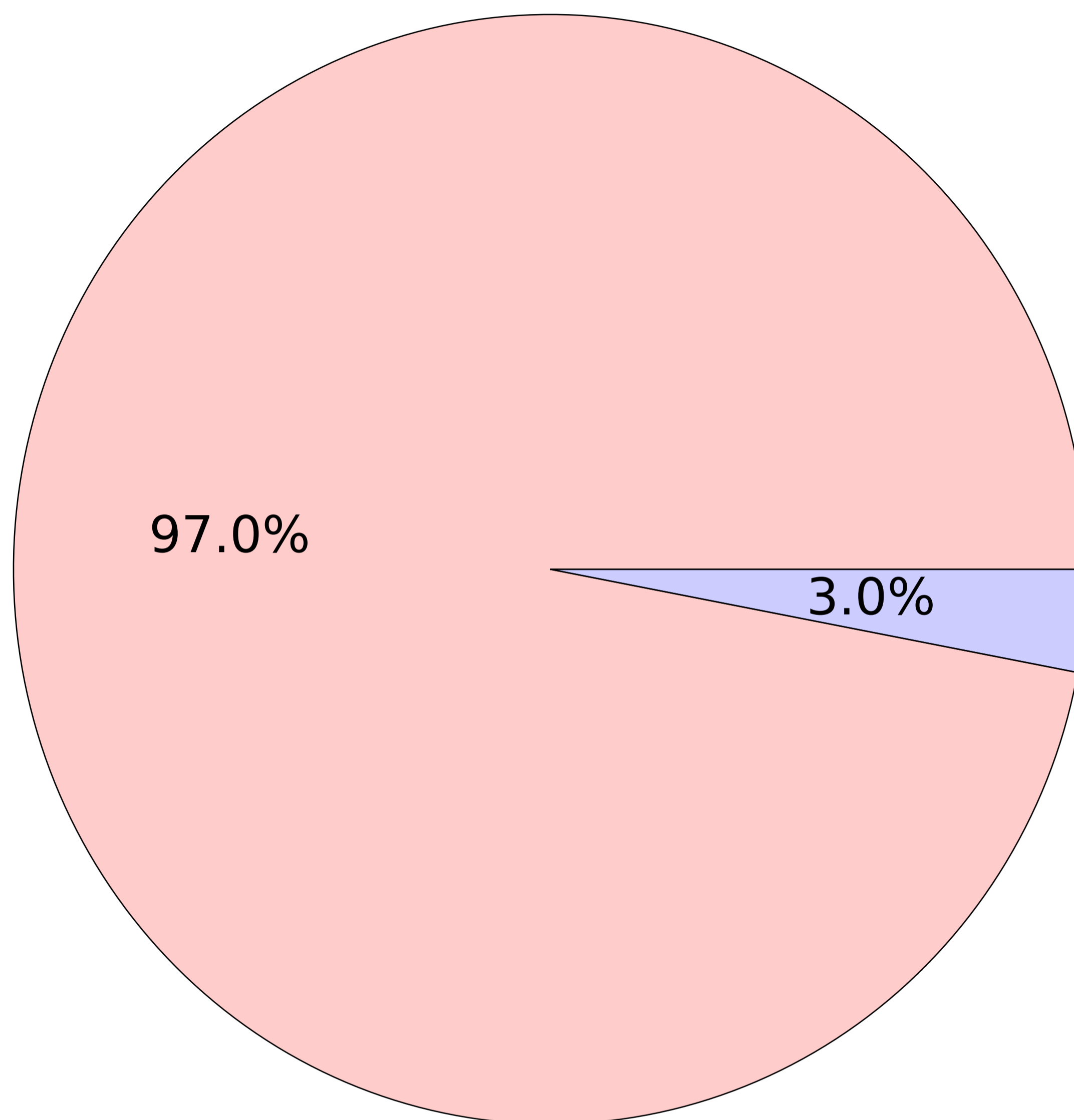

NHEJ  
(394 reads)

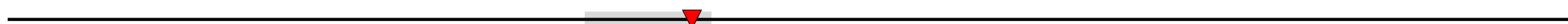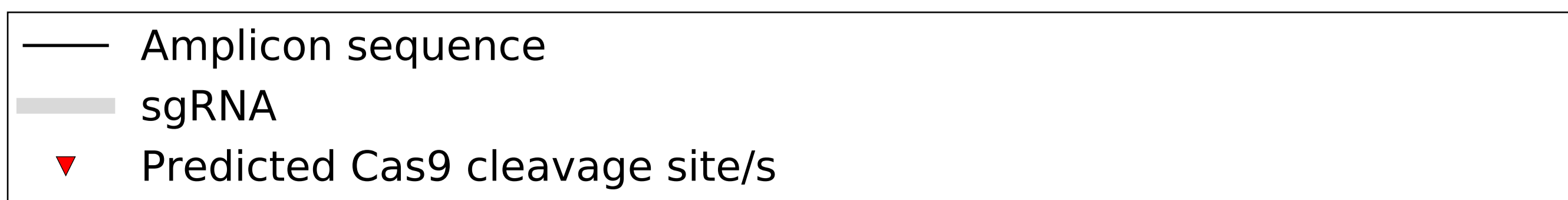

Supplement: Supplementary file 14 — Additional file 14. CRISPResso NHEJ pie charts. [file 12896_2019_565_MOESM14_ESM.zip › CRISPResso_EPSPS-4AL-gRNA7-rep2.pdf]

Unmodified  
(8110 reads)

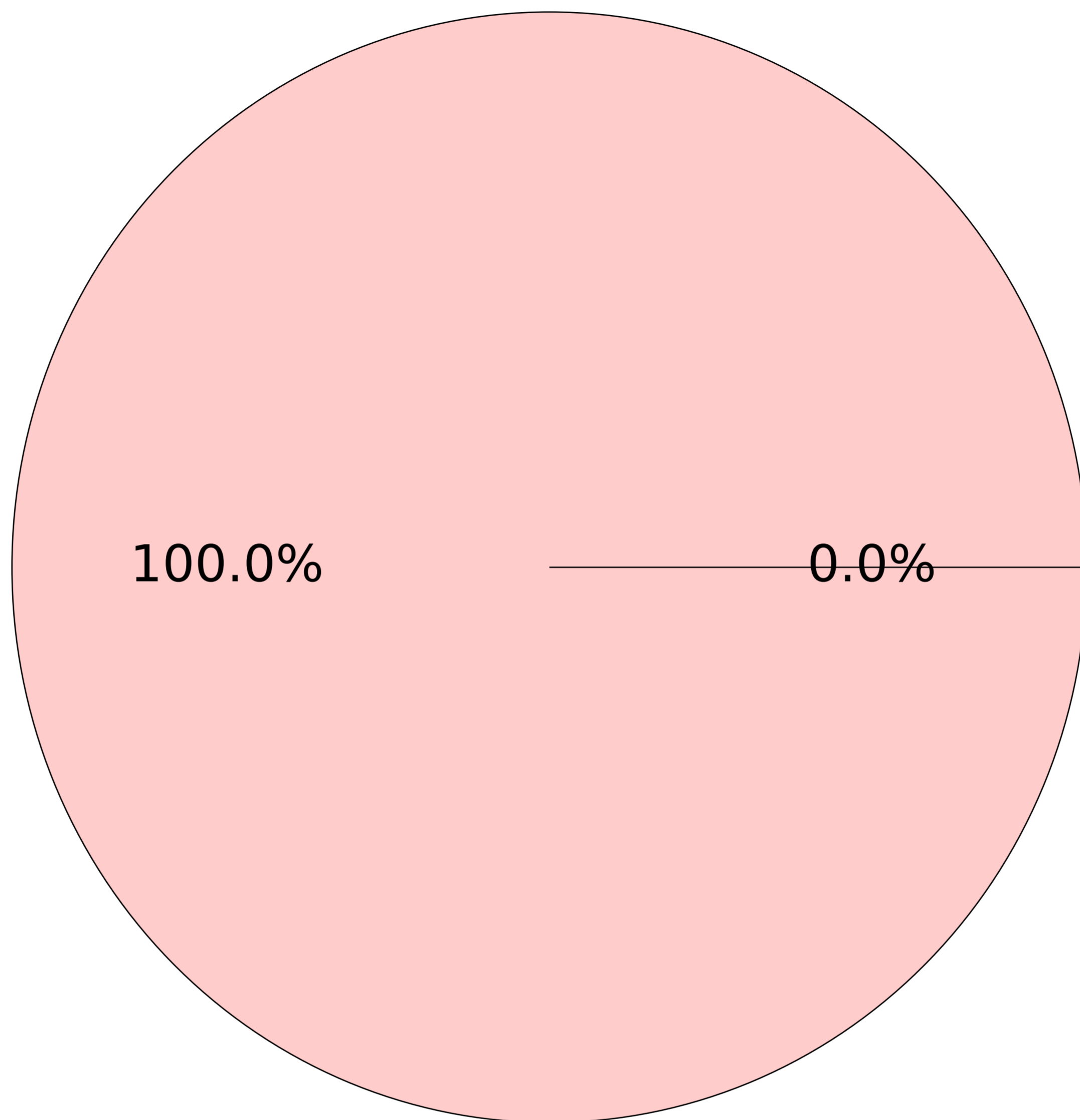

NHEJ  
(0 reads)

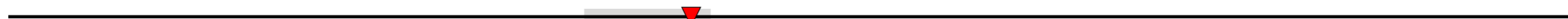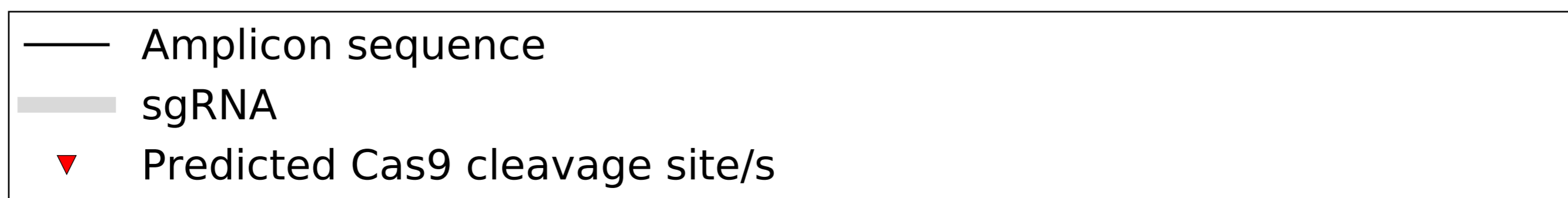

Supplement: Supplementary file 14 — Additional file 14. CRISPResso NHEJ pie charts. [file 12896_2019_565_MOESM14_ESM.zip › CRISPResso_EPSPS-4AL-gRNA7-rep2-negative.pdf]

Unmodified  
(14403 reads)

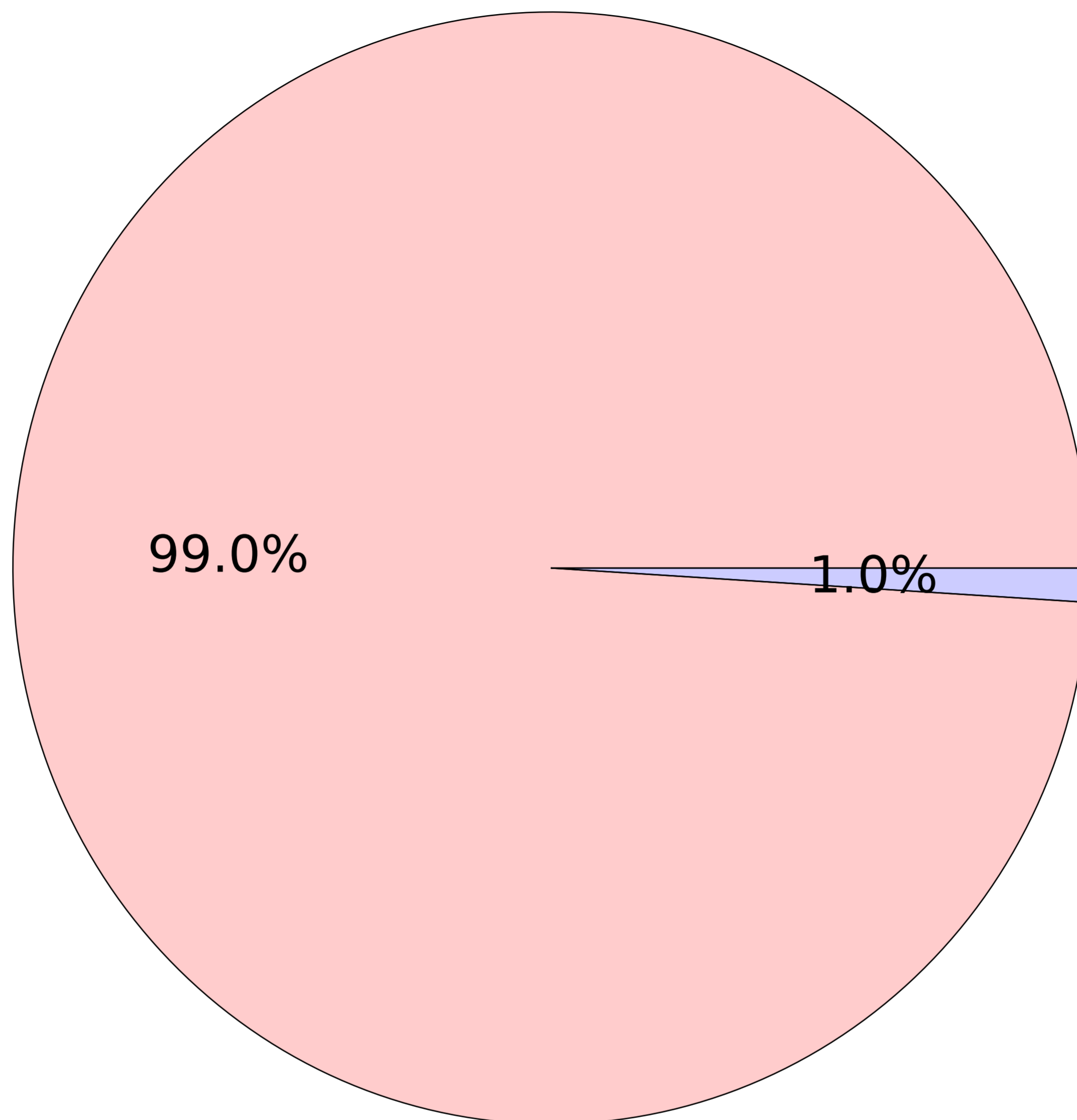

NHEJ  
(143 reads)

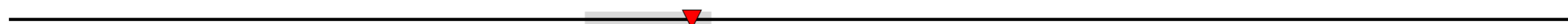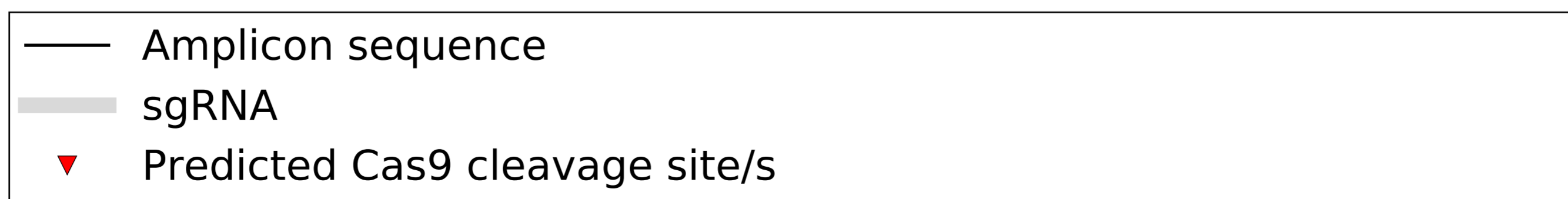

Supplement: Supplementary file 14 — Additional file 14. CRISPResso NHEJ pie charts. [file 12896_2019_565_MOESM14_ESM.zip › CRISPResso_EPSPS-4AL-gRNA7-rep3.pdf]

Unmodified  
(14200 reads)

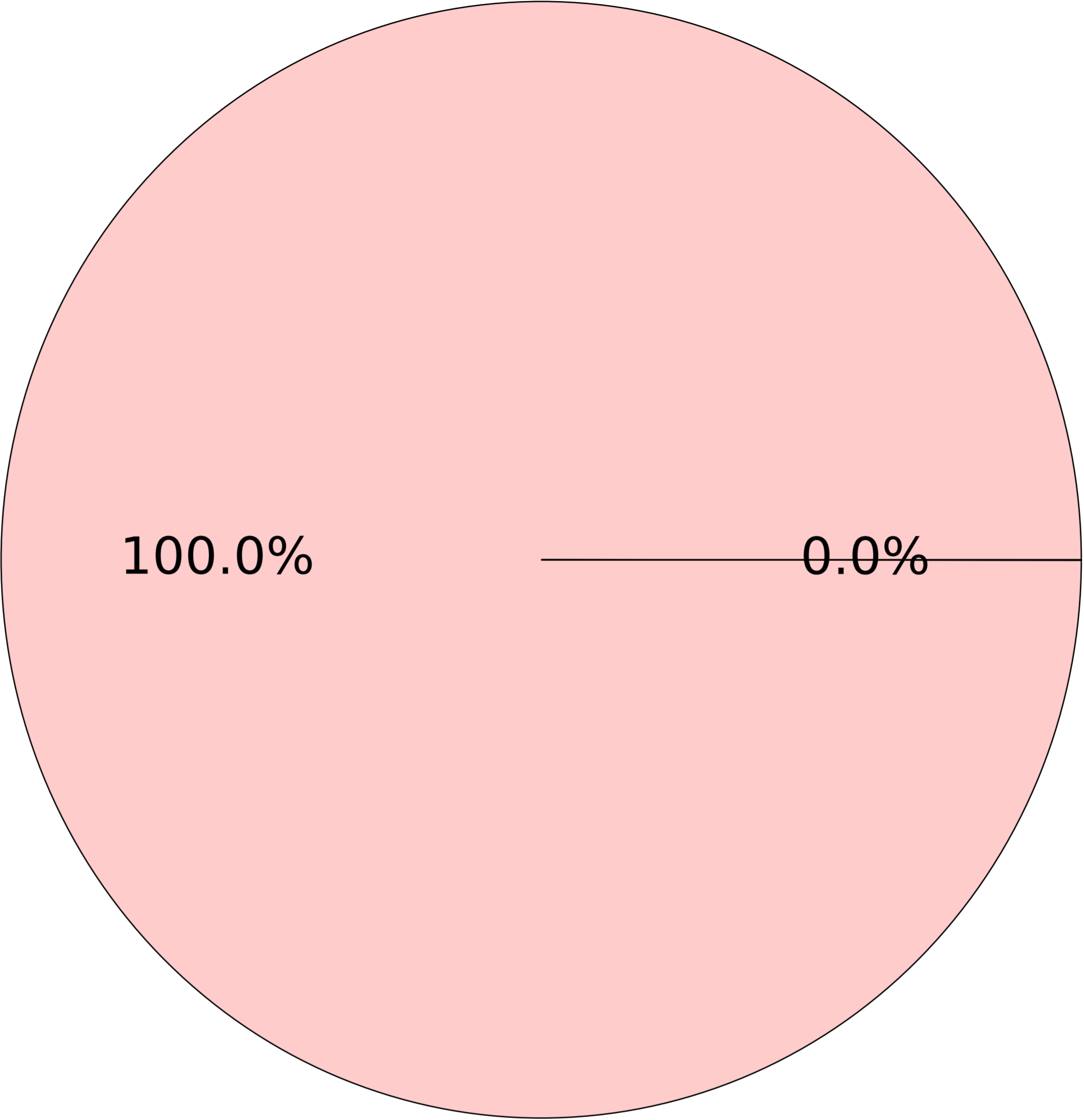

NHEJ  
(2 reads)

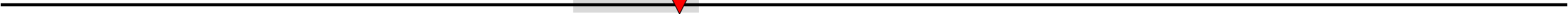

—

Amplicon sequence

—

sgRNA

▼

Predicted Cas9 cleavage site/s

Supplement: Supplementary file 14 — Additional file 14. CRISPResso NHEJ pie charts. [file 12896_2019_565_MOESM14_ESM.zip › CRISPResso_EPSPS-4AL-gRNA7-rep3-negative.pdf]

Unmodified  
(32927 reads)

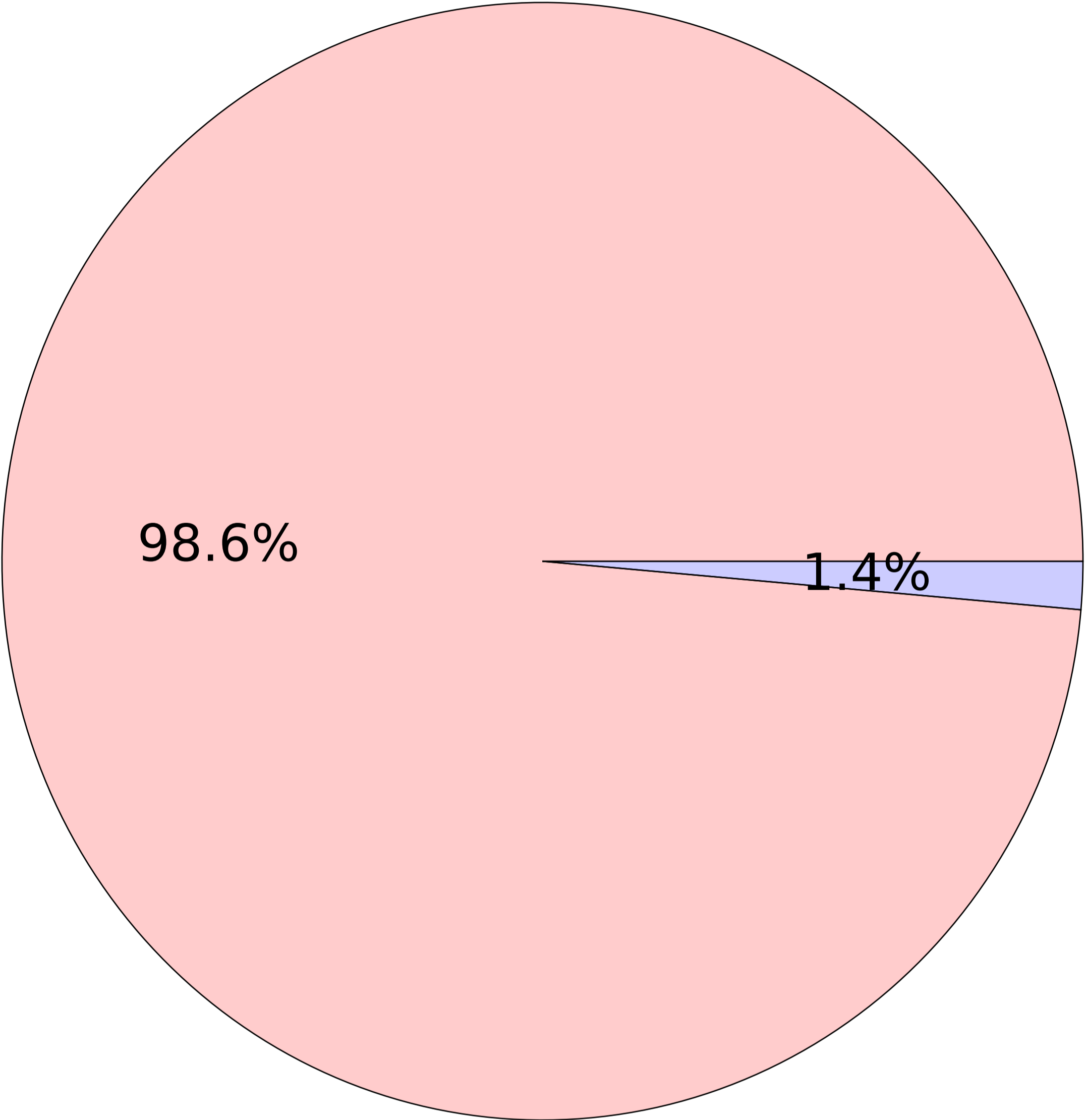

NHEJ  
(462 reads)

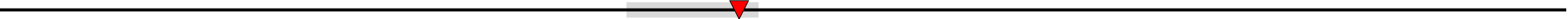

—

Amplicon sequence

—

sgRNA

▼

Predicted Cas9 cleavage site/s

Supplement: Supplementary file 14 — Additional file 14. CRISPResso NHEJ pie charts. [file 12896_2019_565_MOESM14_ESM.zip › CRISPResso_EPSPS-7AS-gRNA1-rep1.pdf]

Unmodified  
(9969 reads)

99.9%

0.1%

NHEJ  
(5 reads)

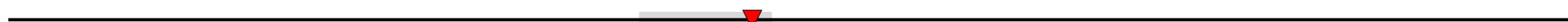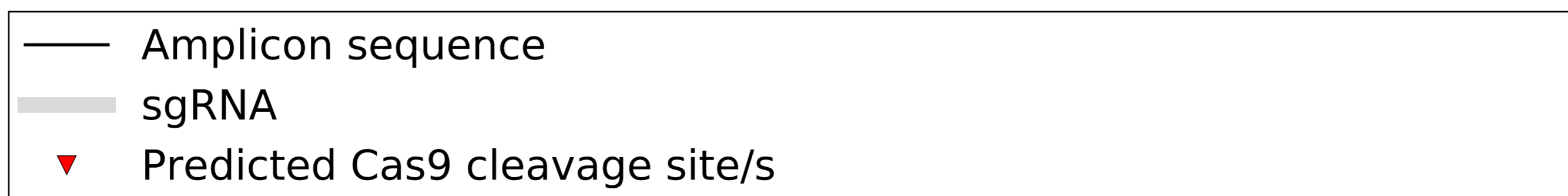

Supplement: Supplementary file 14 — Additional file 14. CRISPResso NHEJ pie charts. [file 12896_2019_565_MOESM14_ESM.zip › CRISPResso_EPSPS-7AS-gRNA1-rep1-negative.pdf]

Unmodified  
(32469 reads)

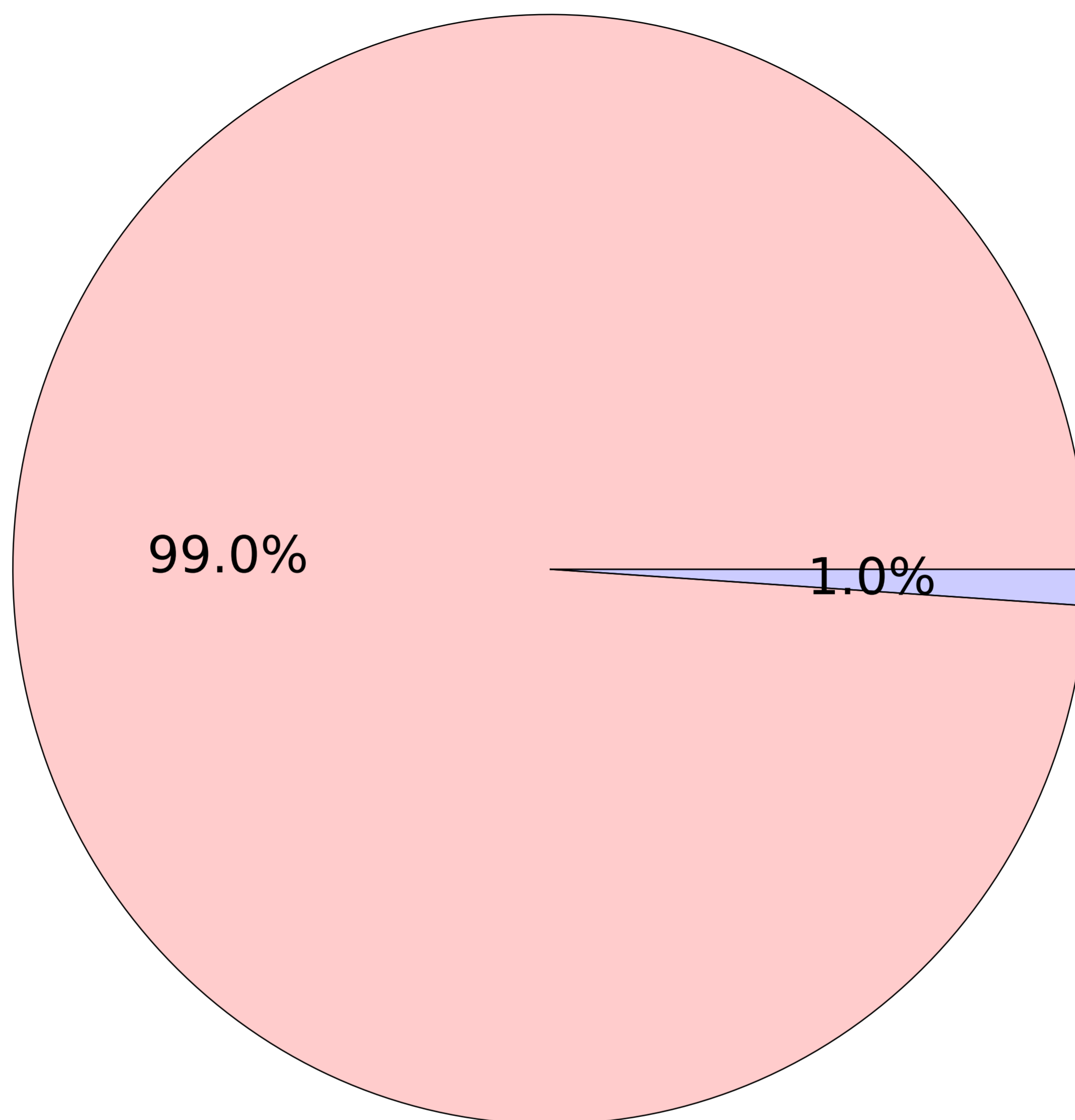

NHEJ  
(343 reads)

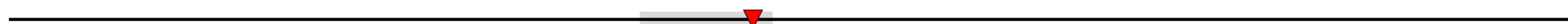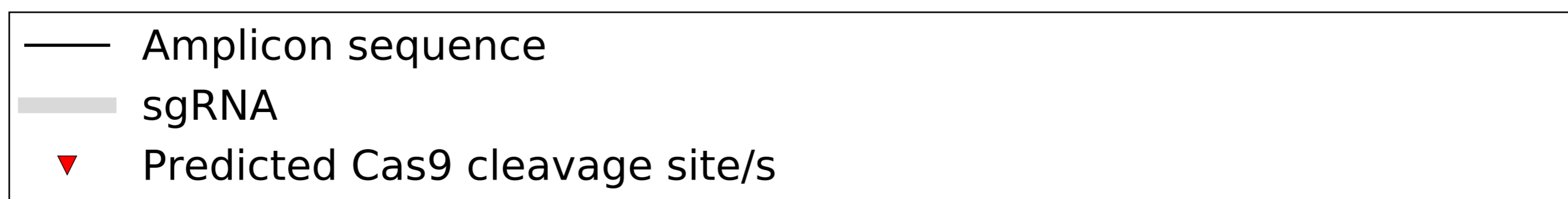

Supplement: Supplementary file 14 — Additional file 14. CRISPResso NHEJ pie charts. [file 12896_2019_565_MOESM14_ESM.zip › CRISPResso_EPSPS-7AS-gRNA1-rep2.pdf]

Unmodified  
(21172 reads)

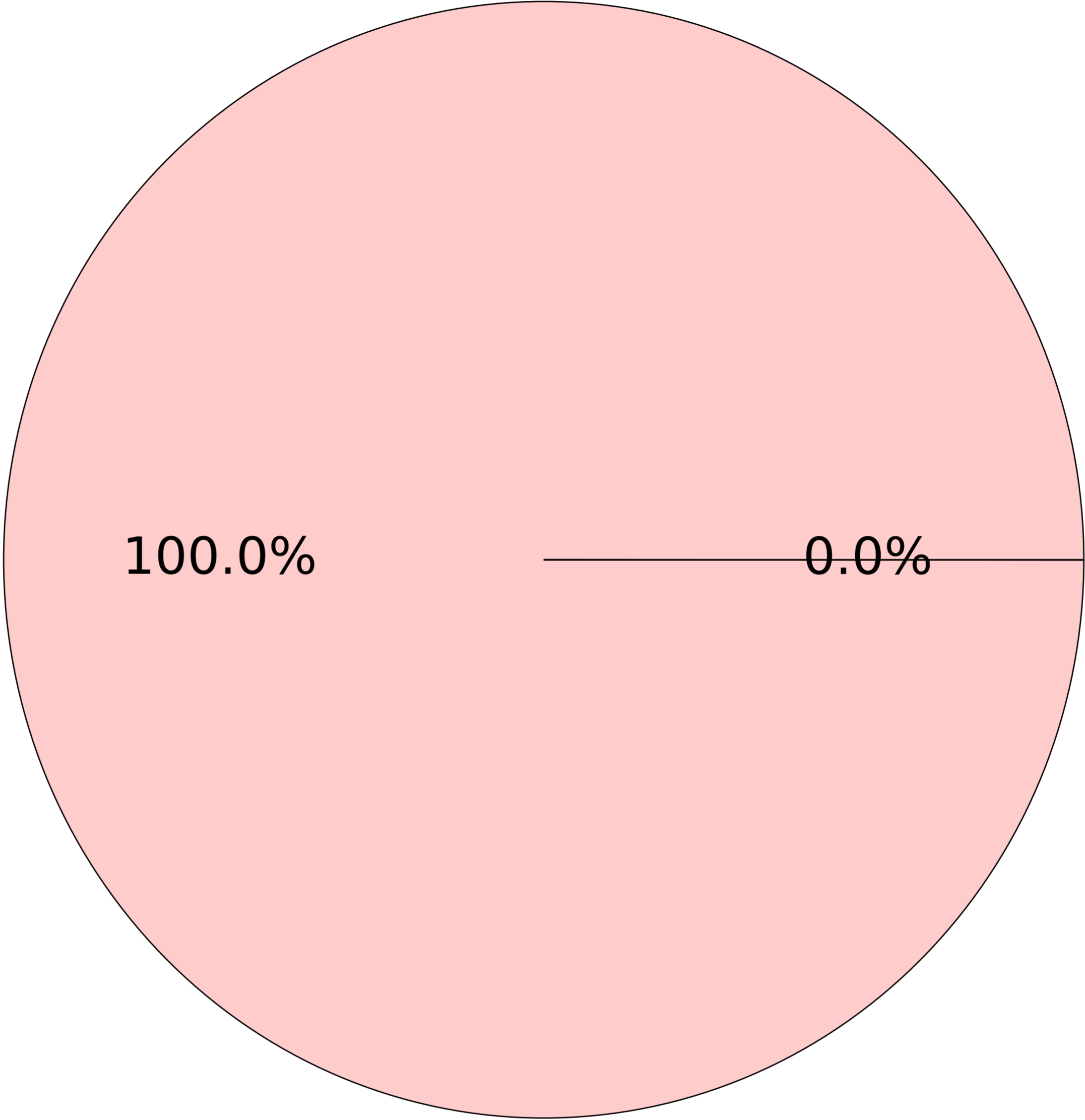

NHEJ  
(2 reads)

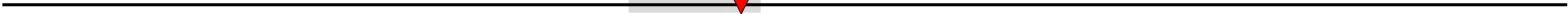

—

Amplicon sequence

—

sgRNA

▼

Predicted Cas9 cleavage site/s

Supplement: Supplementary file 14 — Additional file 14. CRISPResso NHEJ pie charts. [file 12896_2019_565_MOESM14_ESM.zip › CRISPResso_EPSPS-7AS-gRNA1-rep2-negative.pdf]

Unmodified  
(14901 reads)

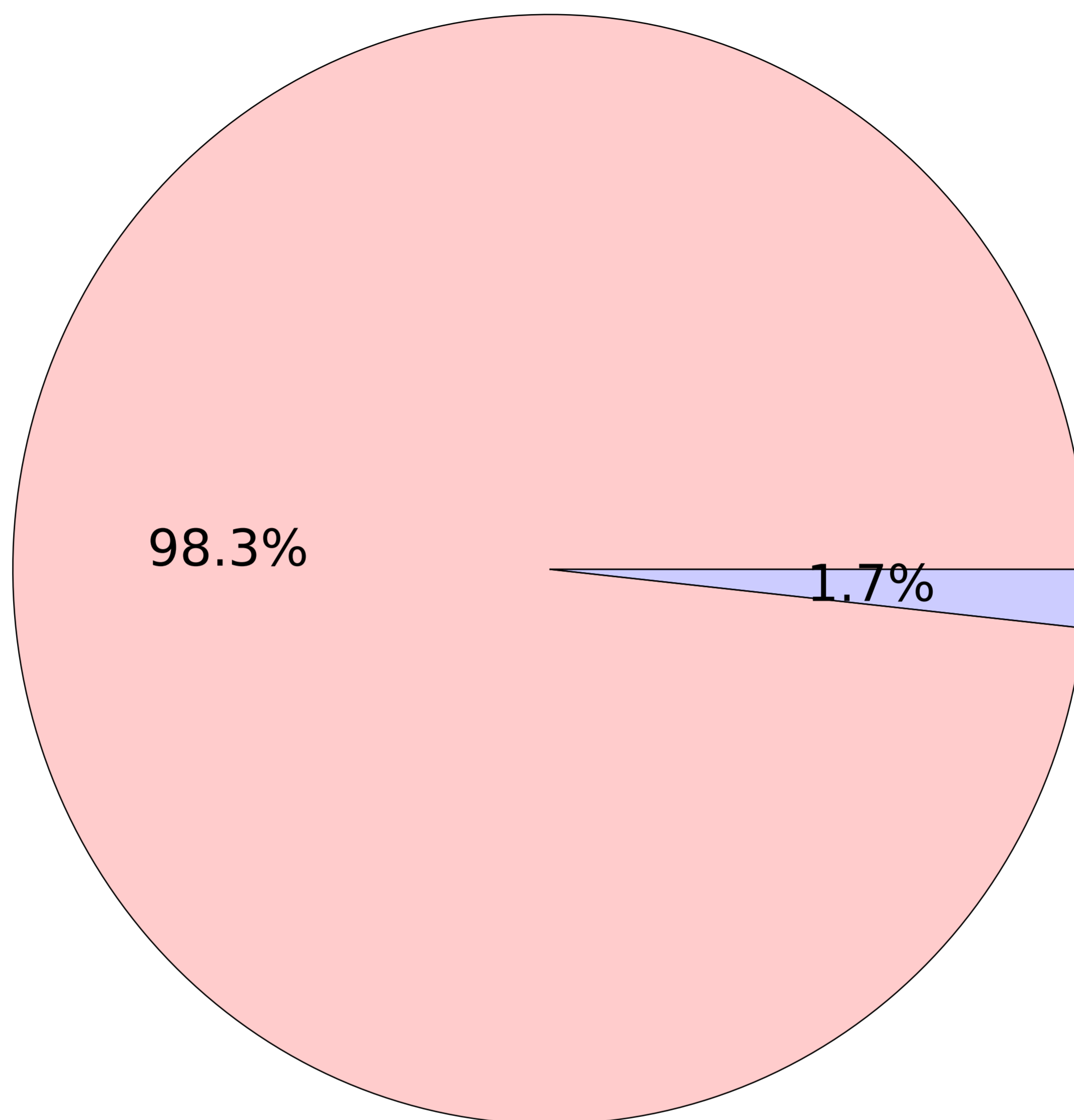

NHEJ  
(256 reads)

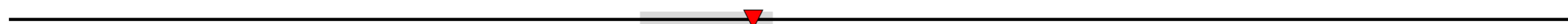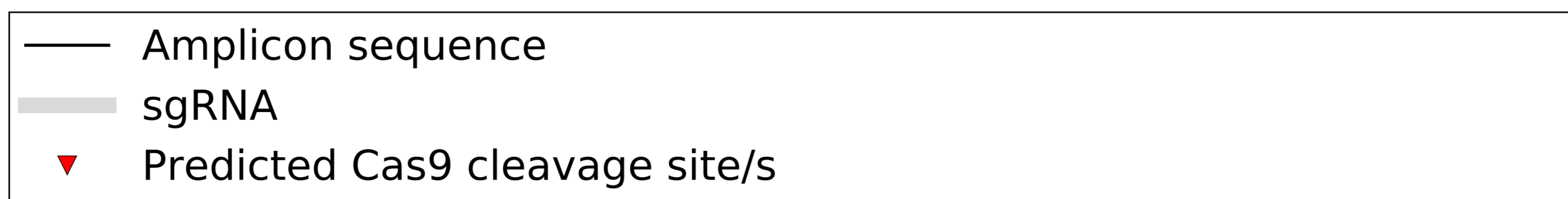

Supplement: Supplementary file 14 — Additional file 14. CRISPResso NHEJ pie charts. [file 12896_2019_565_MOESM14_ESM.zip › CRISPResso_EPSPS-7AS-gRNA1-rep3.pdf]

Unmodified  
(28701 reads)

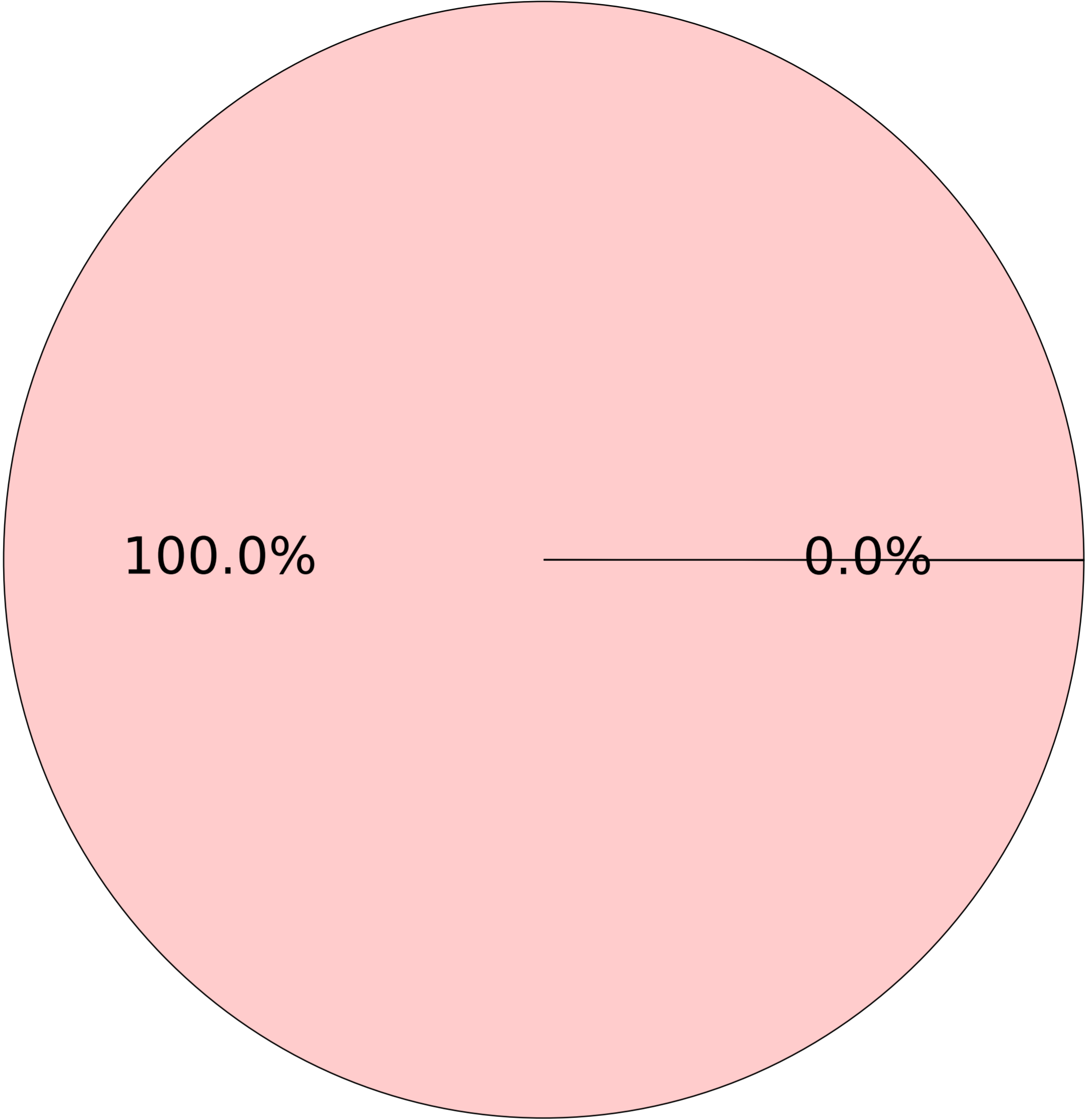

NHEJ  
(6 reads)

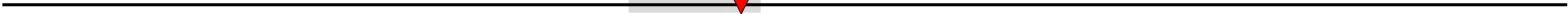

—

Amplicon sequence

—

sgRNA

▼

Predicted Cas9 cleavage site/s

Supplement: Supplementary file 14 — Additional file 14. CRISPResso NHEJ pie charts. [file 12896_2019_565_MOESM14_ESM.zip › CRISPResso_EPSPS-7AS-gRNA1-rep3-negative.pdf]

Unmodified  
(18900 reads)

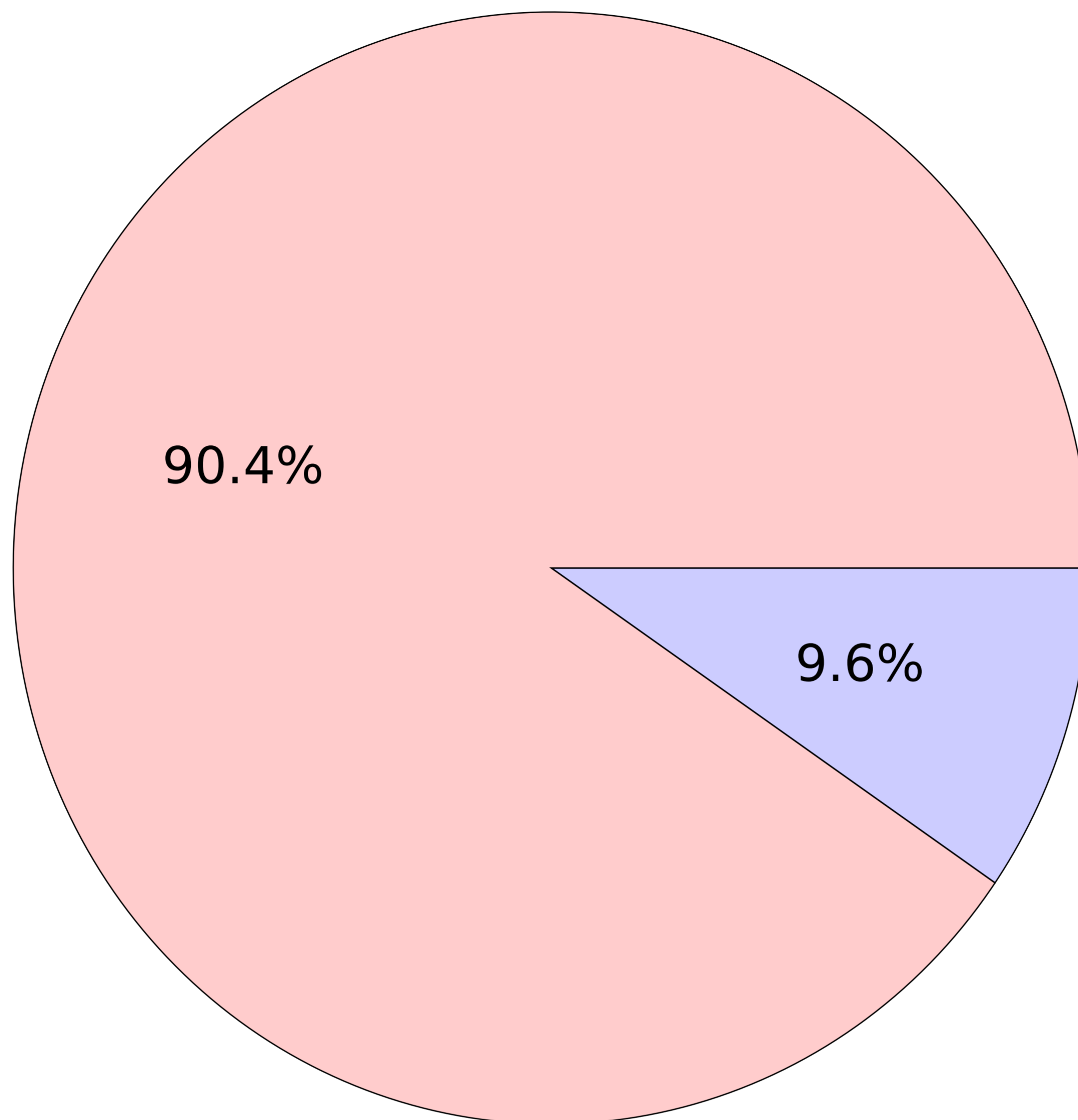

NHEJ  
(2002 reads)

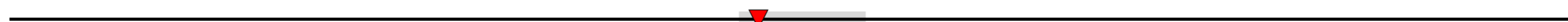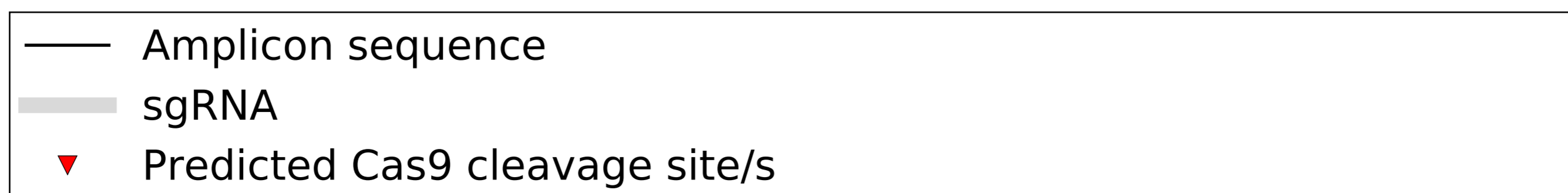

Supplement: Supplementary file 14 — Additional file 14. CRISPResso NHEJ pie charts. [file 12896_2019_565_MOESM14_ESM.zip › CRISPResso_EPSPS-7AS-gRNA2-rep1.pdf]

Unmodified  
(9969 reads)

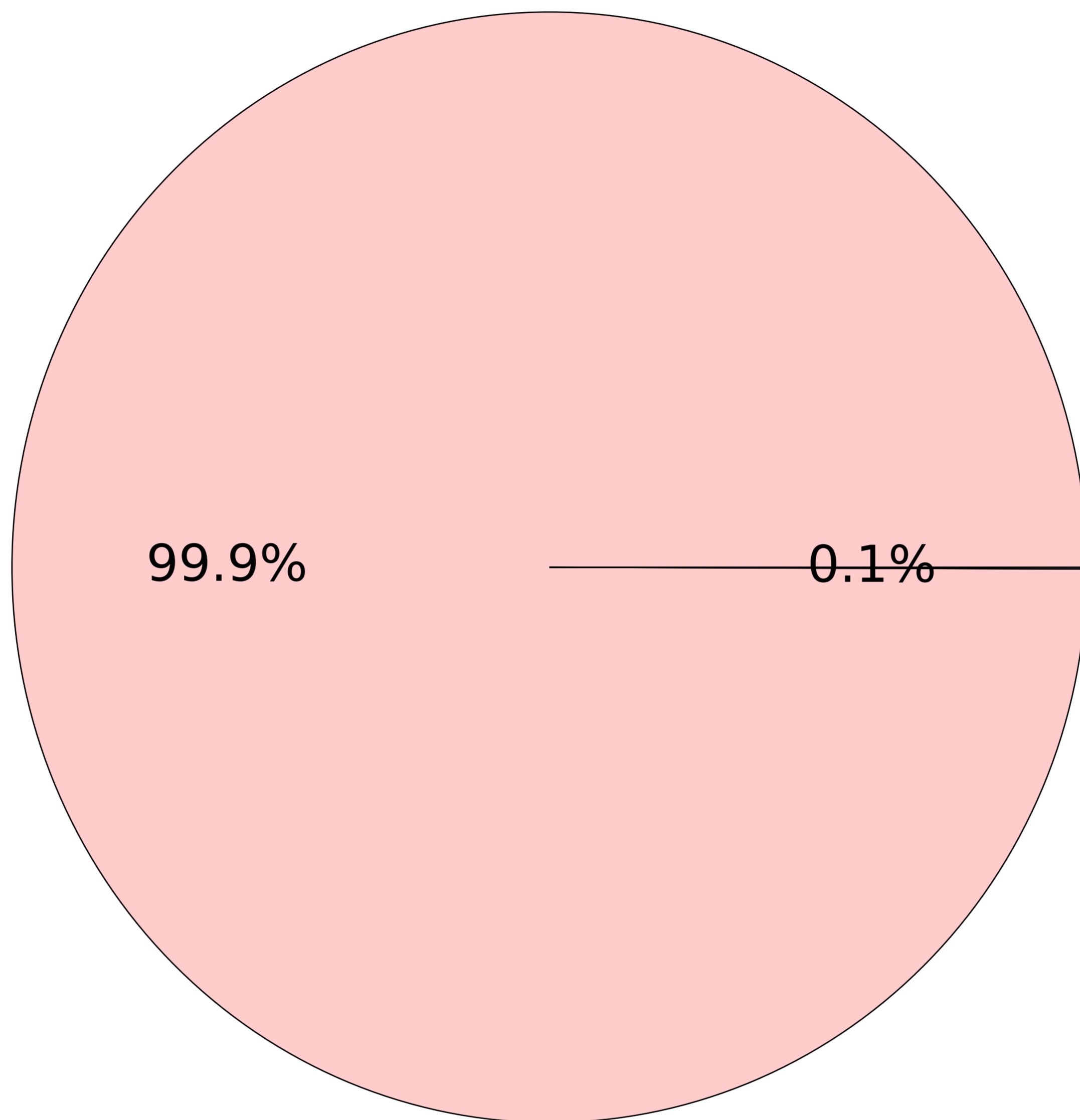

NHEJ  
(5 reads)

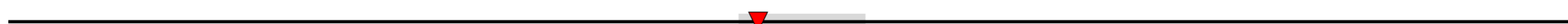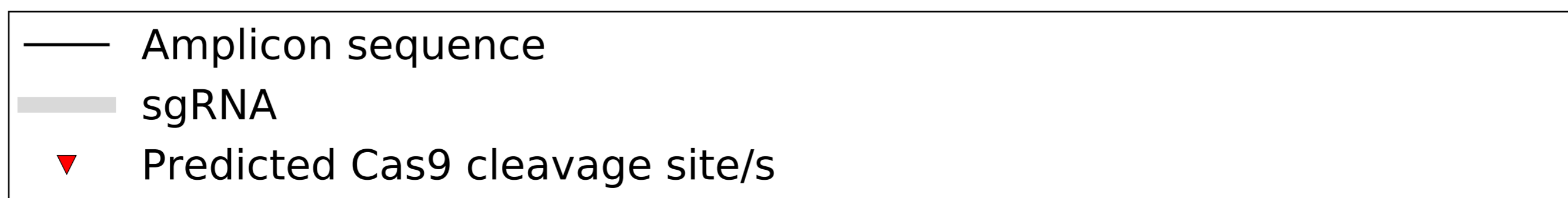

Supplement: Supplementary file 14 — Additional file 14. CRISPResso NHEJ pie charts. [file 12896_2019_565_MOESM14_ESM.zip › CRISPResso_EPSPS-7AS-gRNA2-rep1-negative.pdf]

Unmodified  
(15190 reads)

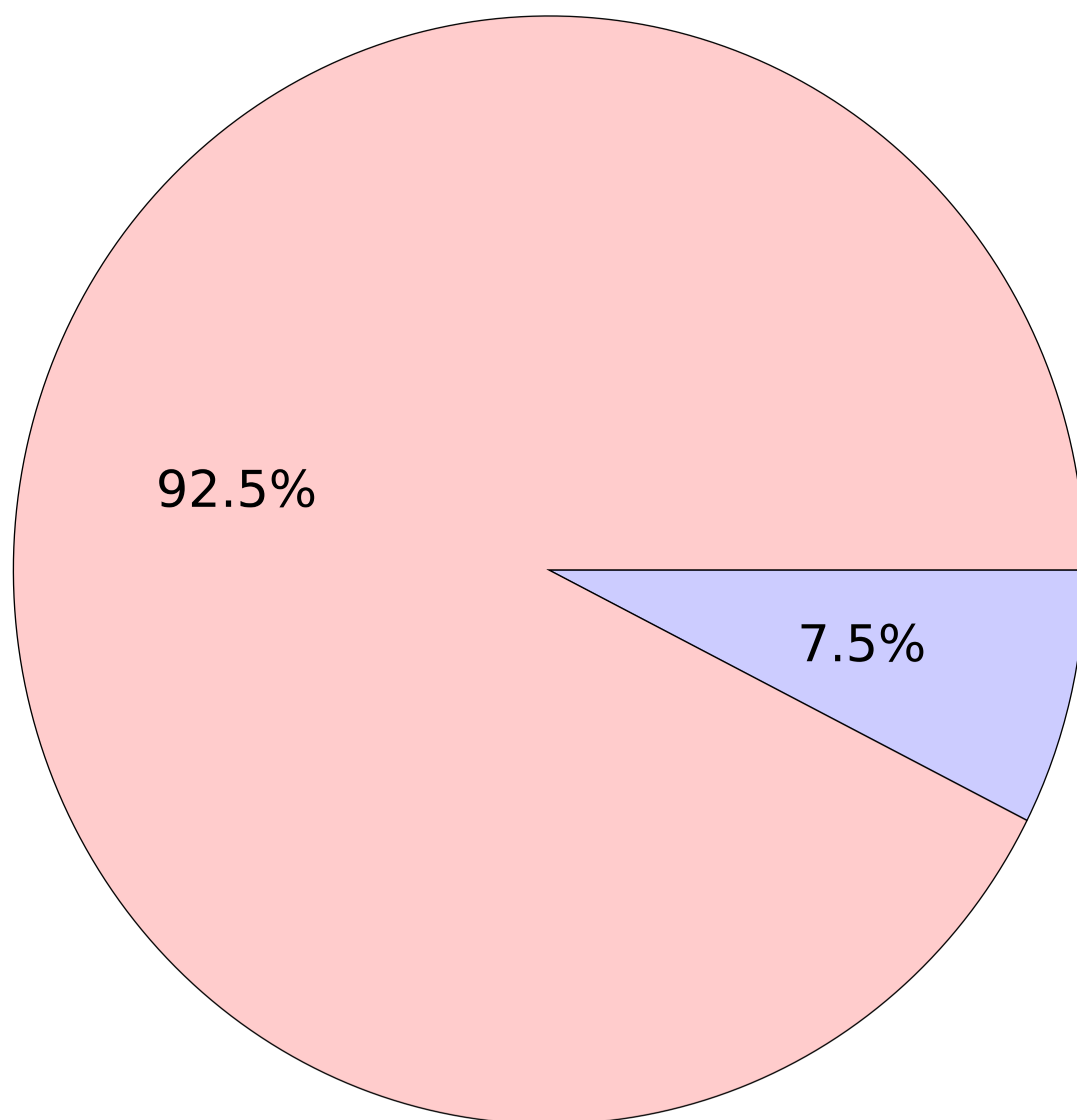

NHEJ  
(1225 reads)

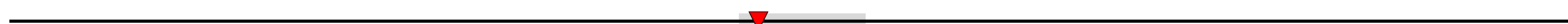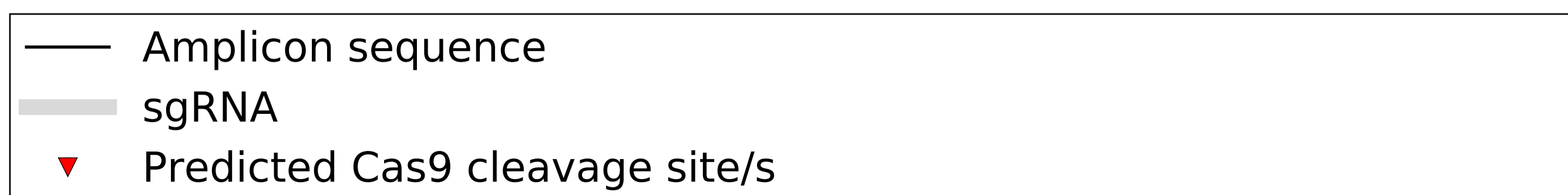

Supplement: Supplementary file 14 — Additional file 14. CRISPResso NHEJ pie charts. [file 12896_2019_565_MOESM14_ESM.zip › CRISPResso_EPSPS-7AS-gRNA2-rep2.pdf]

Unmodified  
(21172 reads)

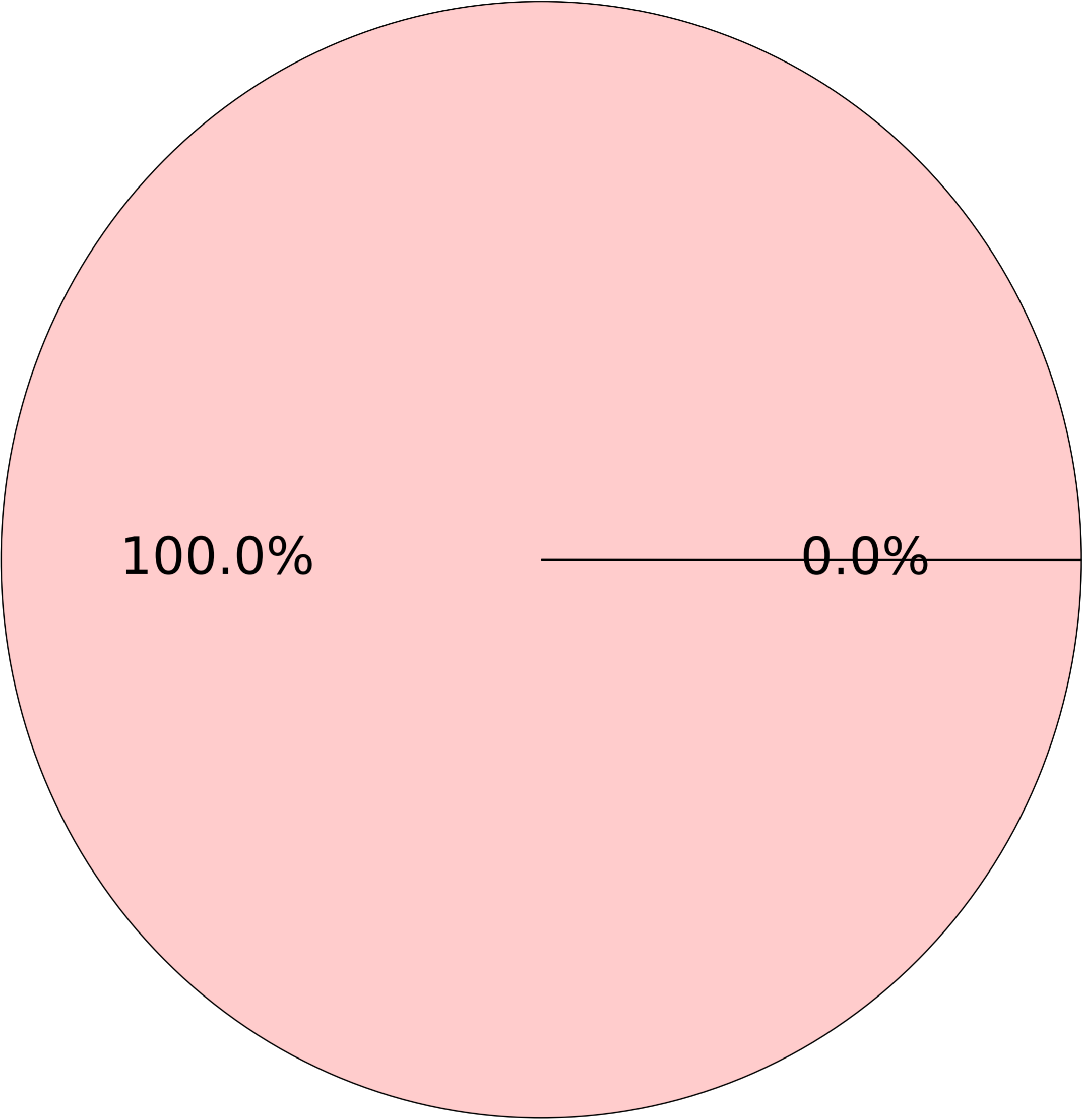

NHEJ  
(2 reads)

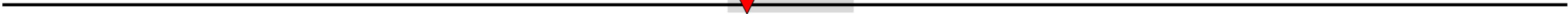

—

Amplicon sequence

—

sgRNA

▼

Predicted Cas9 cleavage site/s

Supplement: Supplementary file 14 — Additional file 14. CRISPResso NHEJ pie charts. [file 12896_2019_565_MOESM14_ESM.zip › CRISPResso_EPSPS-7AS-gRNA2-rep2-negative.pdf]

Unmodified  
(14118 reads)

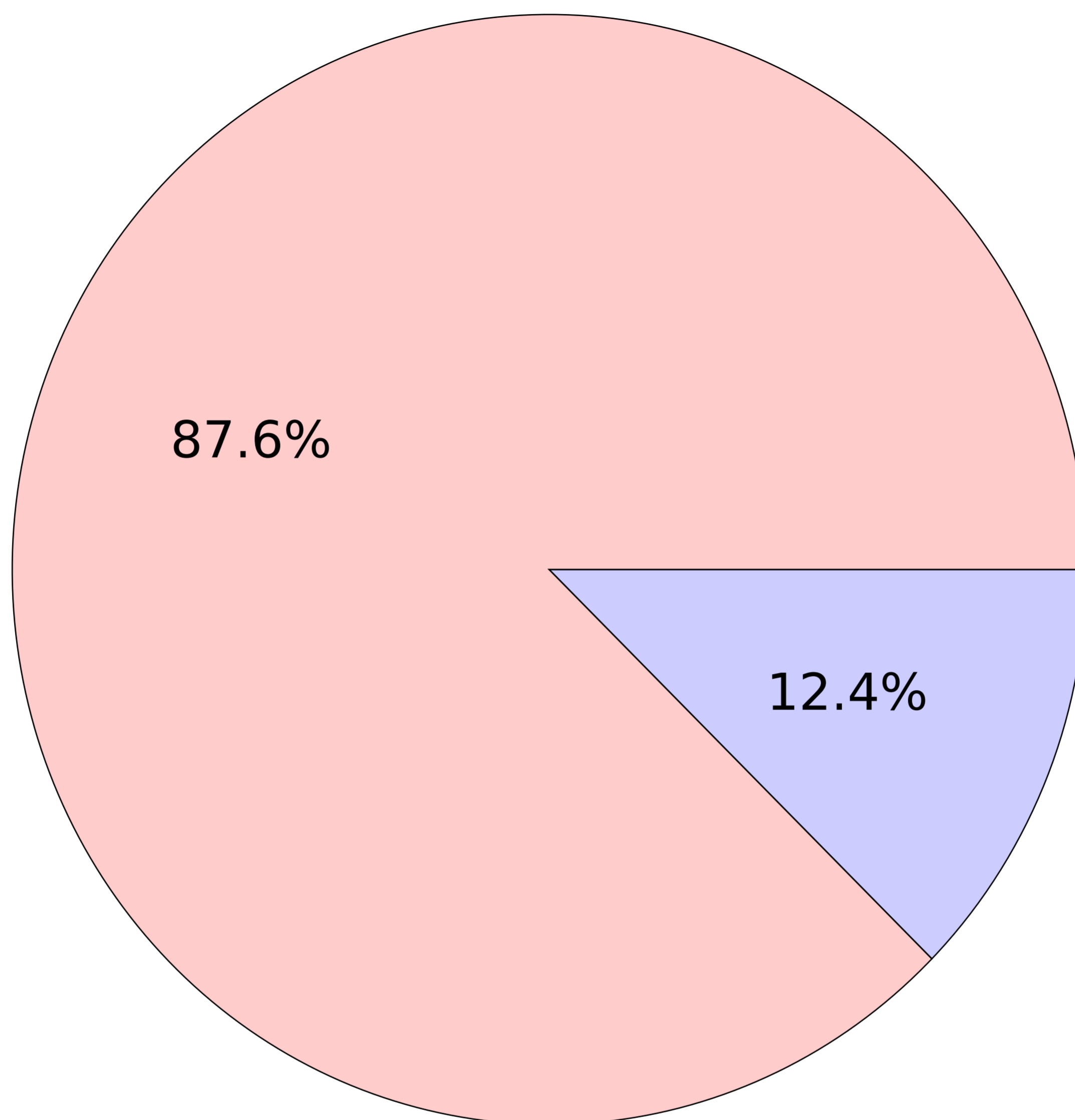

NHEJ  
(1993 reads)

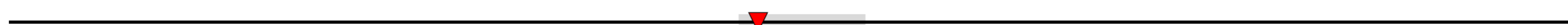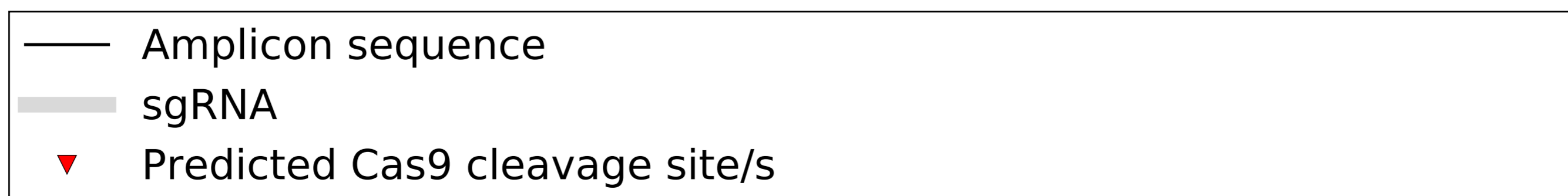

Supplement: Supplementary file 14 — Additional file 14. CRISPResso NHEJ pie charts. [file 12896_2019_565_MOESM14_ESM.zip › CRISPResso_EPSPS-7AS-gRNA2-rep3.pdf]

Unmodified  
(28701 reads)

100.0%

0.0%

NHEJ  
(6 reads)

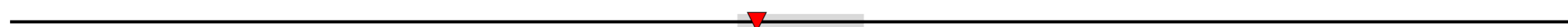

- Amplicon sequence
- sgRNA
- ▼ Predicted Cas9 cleavage site/s

Supplement: Supplementary file 14 — Additional file 14. CRISPResso NHEJ pie charts. [file 12896_2019_565_MOESM14_ESM.zip › CRISPResso_EPSPS-7AS-gRNA2-rep3-negative.pdf]

Unmodified  
(31756 reads)

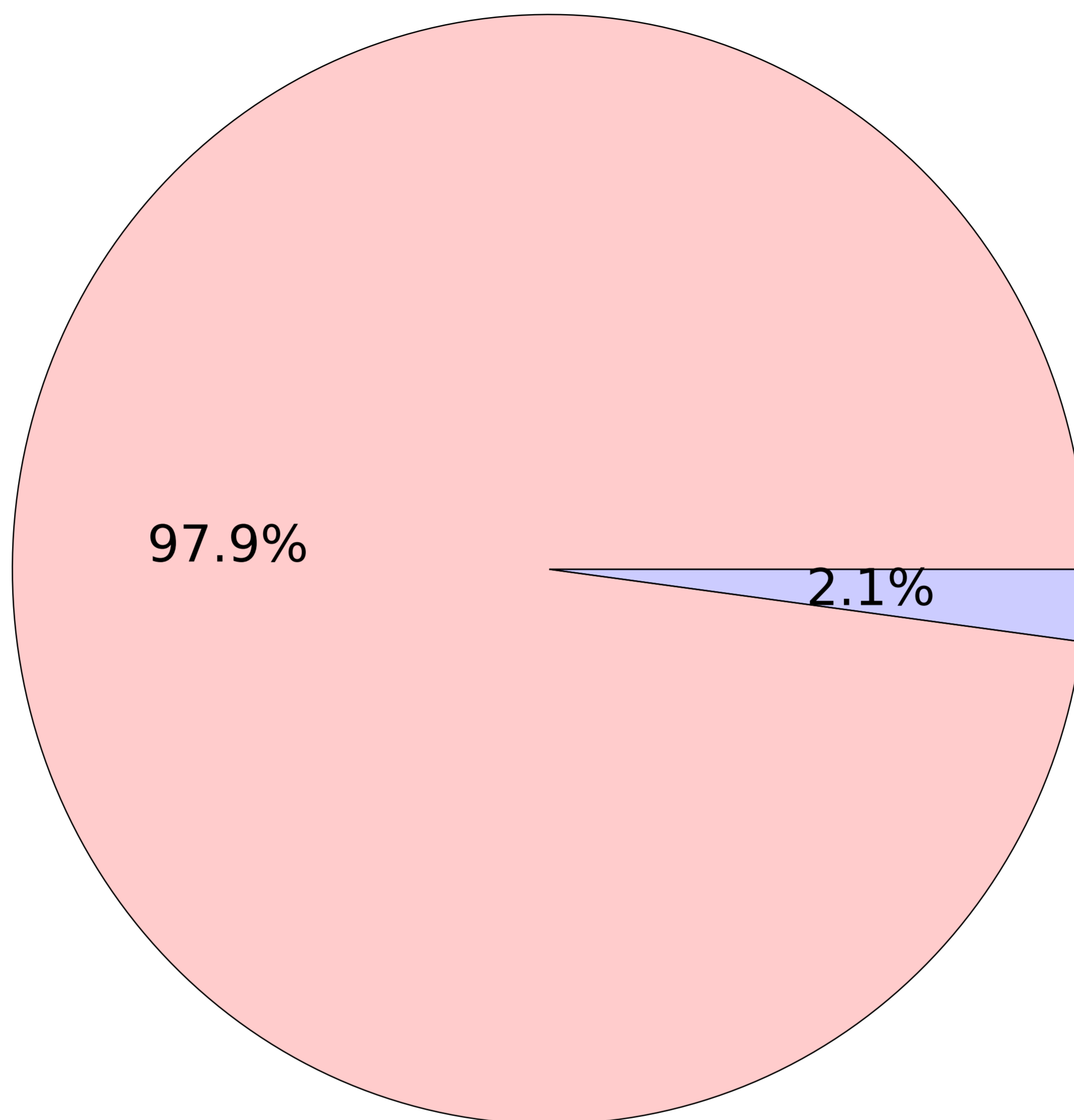

NHEJ  
(676 reads)

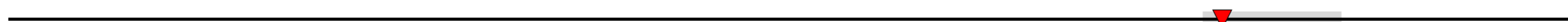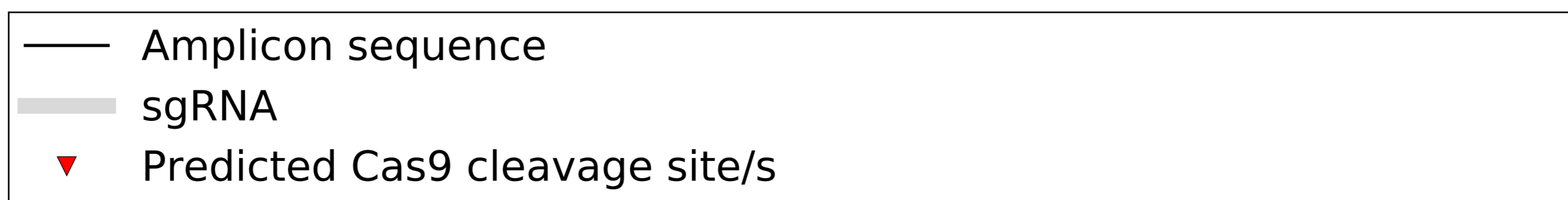

Supplement: Supplementary file 14 — Additional file 14. CRISPResso NHEJ pie charts. [file 12896_2019_565_MOESM14_ESM.zip › CRISPResso_EPSPS-7AS-gRNA3-rep1.pdf]

Unmodified  
(9974 reads)

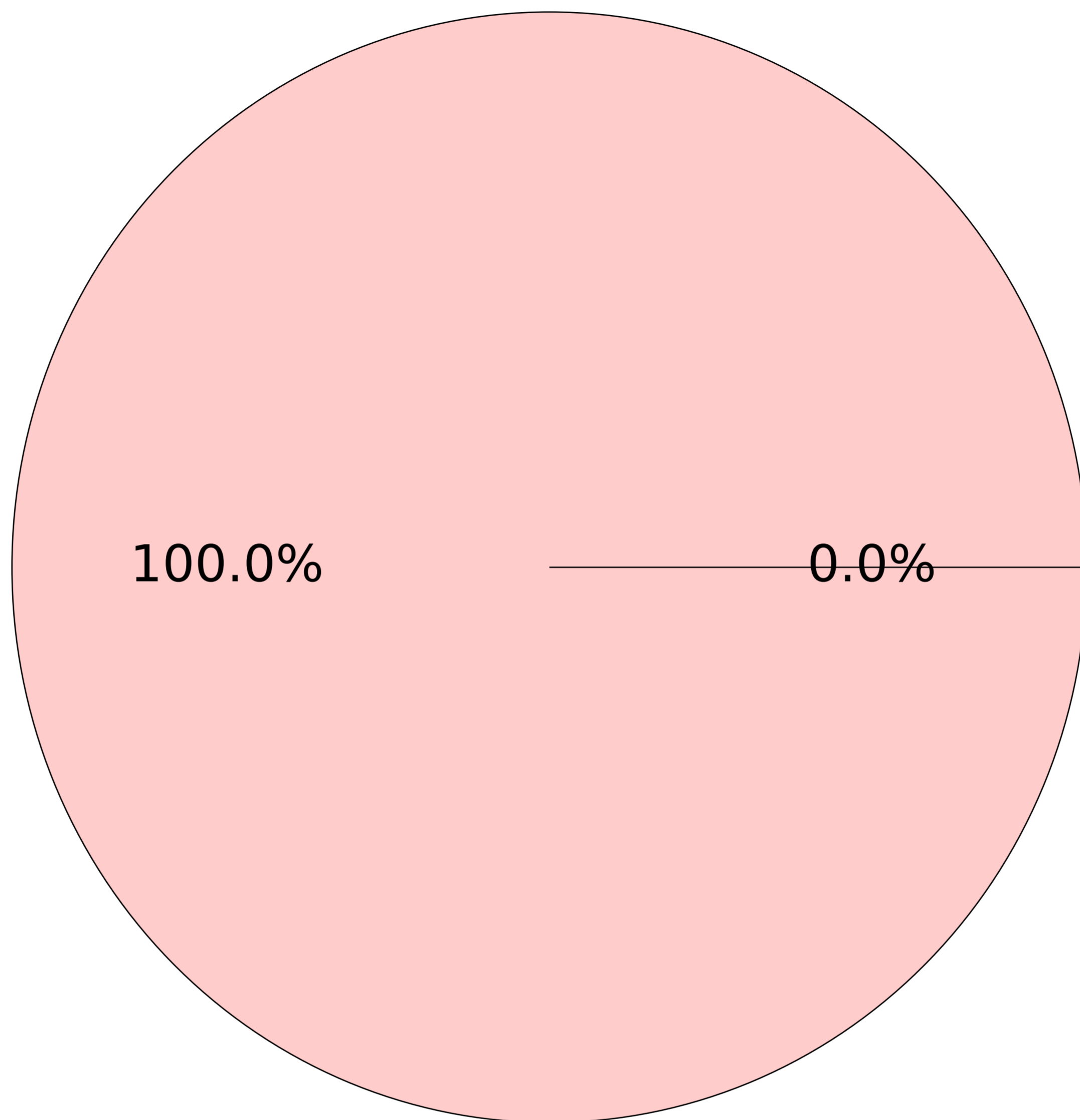

NHEJ  
(0 reads)

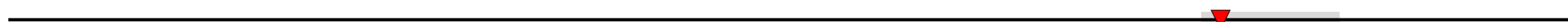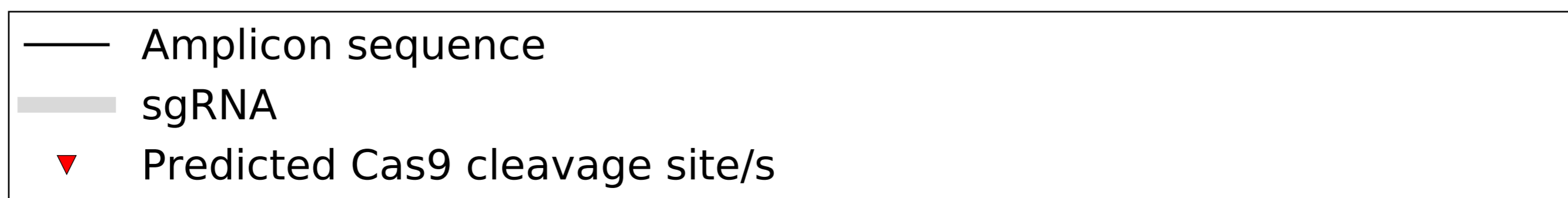

Supplement: Supplementary file 14 — Additional file 14. CRISPResso NHEJ pie charts. [file 12896_2019_565_MOESM14_ESM.zip › CRISPResso_EPSPS-7AS-gRNA3-rep1-negative.pdf]

Unmodified  
(27158 reads)

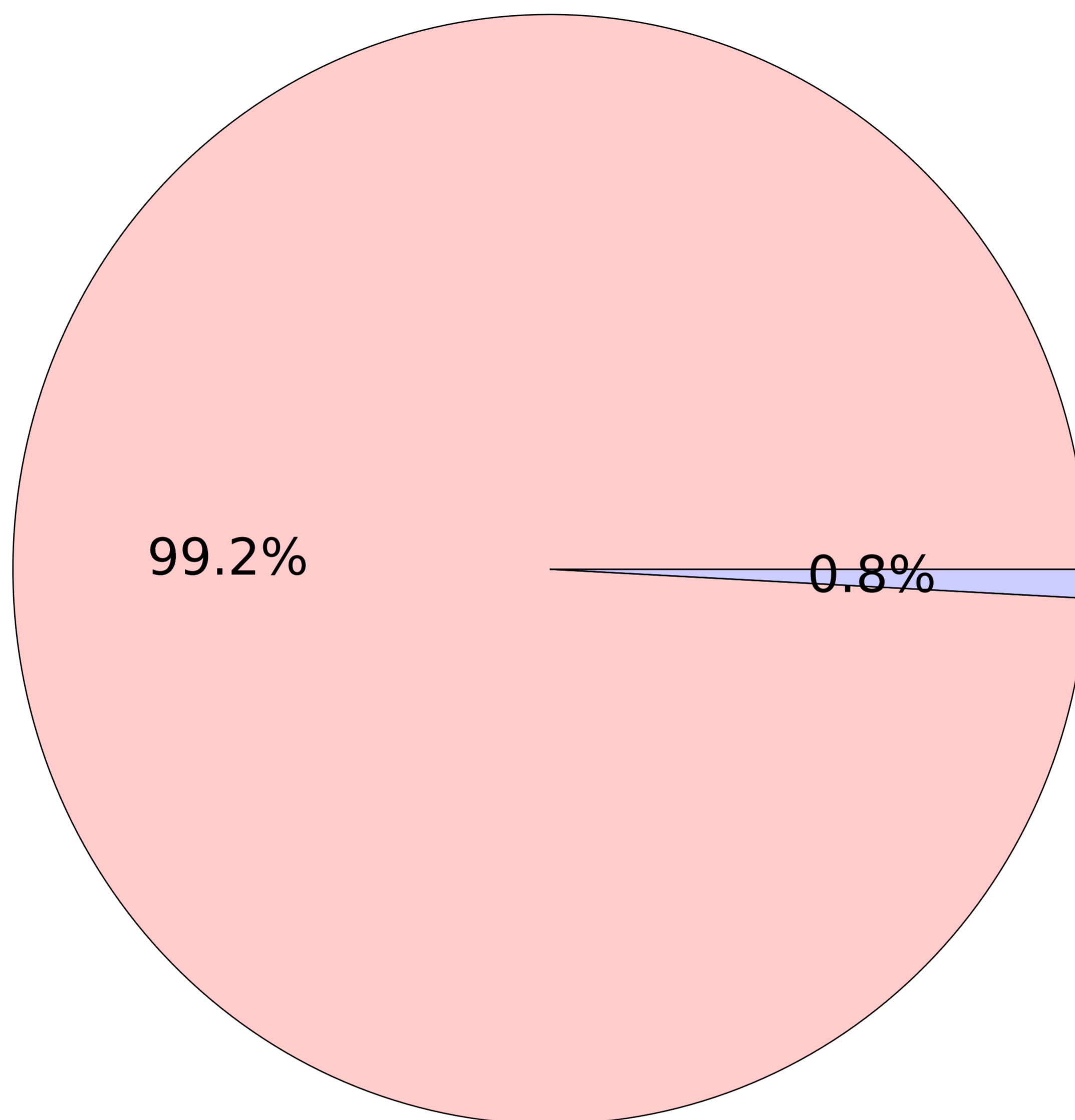

NHEJ  
(228 reads)

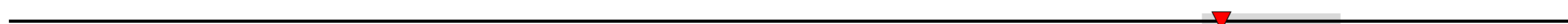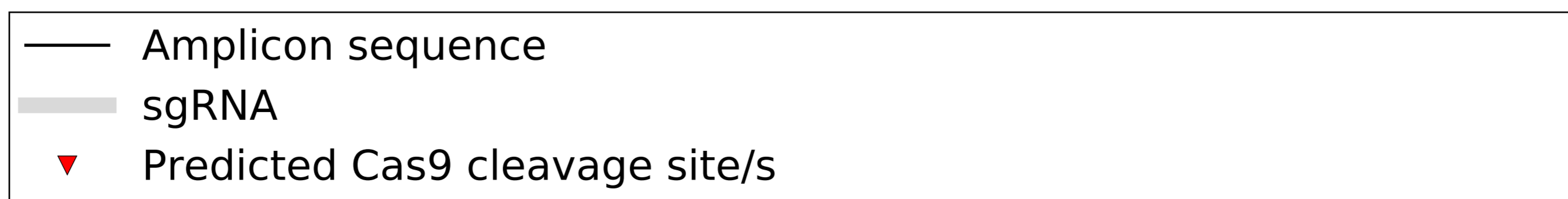

Supplement: Supplementary file 14 — Additional file 14. CRISPResso NHEJ pie charts. [file 12896_2019_565_MOESM14_ESM.zip › CRISPResso_EPSPS-7AS-gRNA3-rep2.pdf]

Unmodified  
(21171 reads)

100.0%

0.0%

NHEJ  
(3 reads)

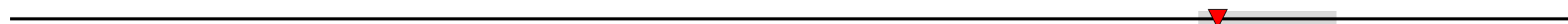

- Amplicon sequence
- sgRNA
- ▼ Predicted Cas9 cleavage site/s

Supplement: Supplementary file 14 — Additional file 14. CRISPResso NHEJ pie charts. [file 12896_2019_565_MOESM14_ESM.zip › CRISPResso_EPSPS-7AS-gRNA3-rep2-negative.pdf]

Unmodified  
(22413 reads)

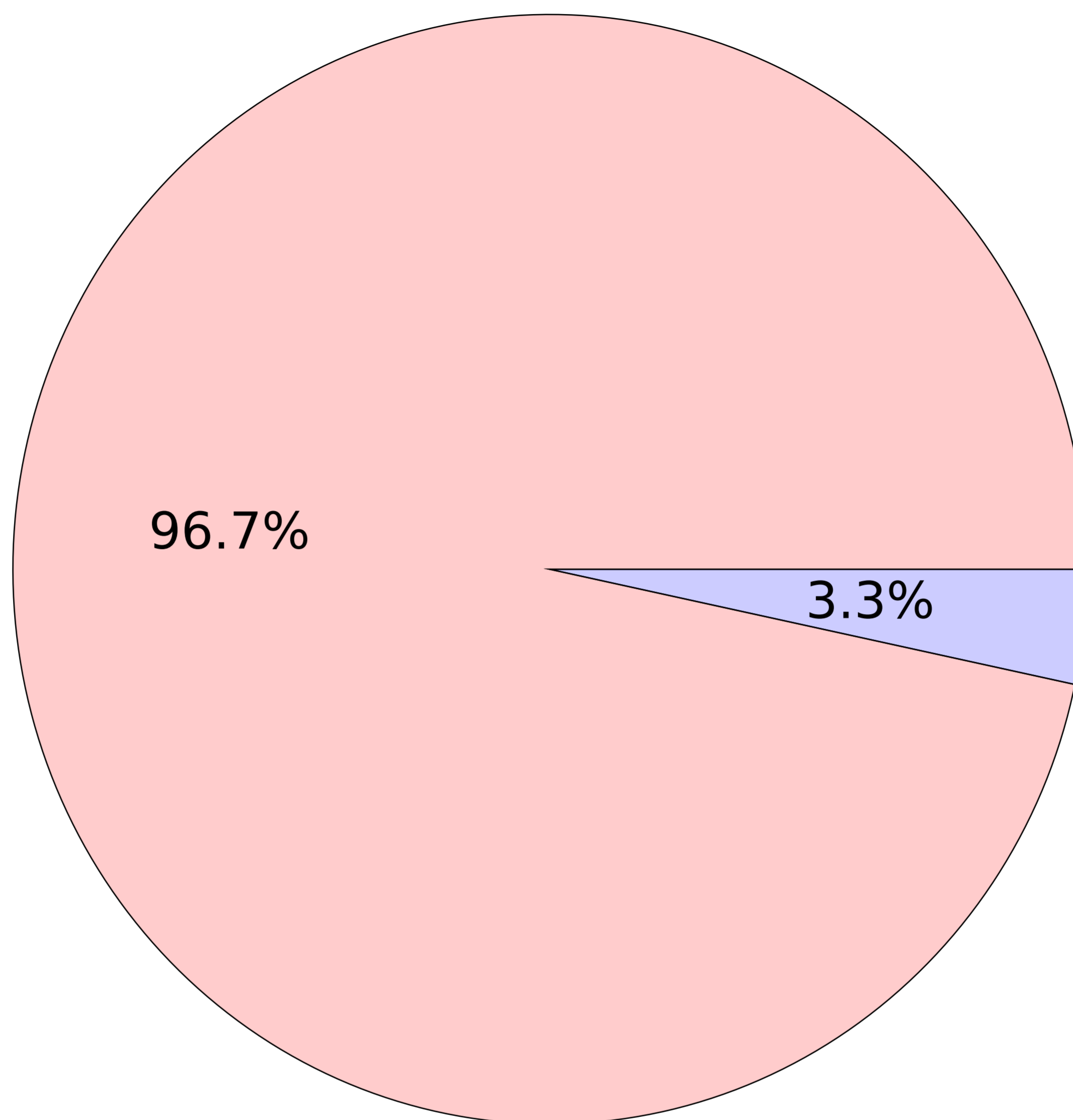

NHEJ  
(772 reads)

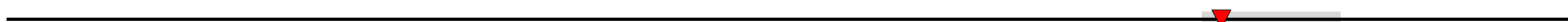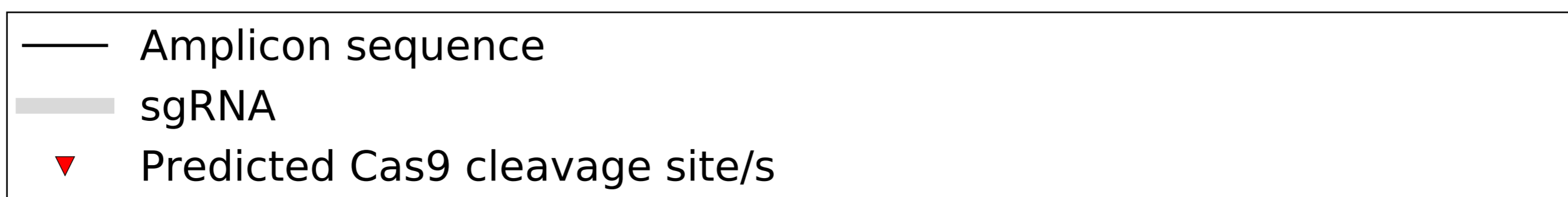

Supplement: Supplementary file 14 — Additional file 14. CRISPResso NHEJ pie charts. [file 12896_2019_565_MOESM14_ESM.zip › CRISPResso_EPSPS-7AS-gRNA3-rep3.pdf]

Unmodified  
(28700 reads)

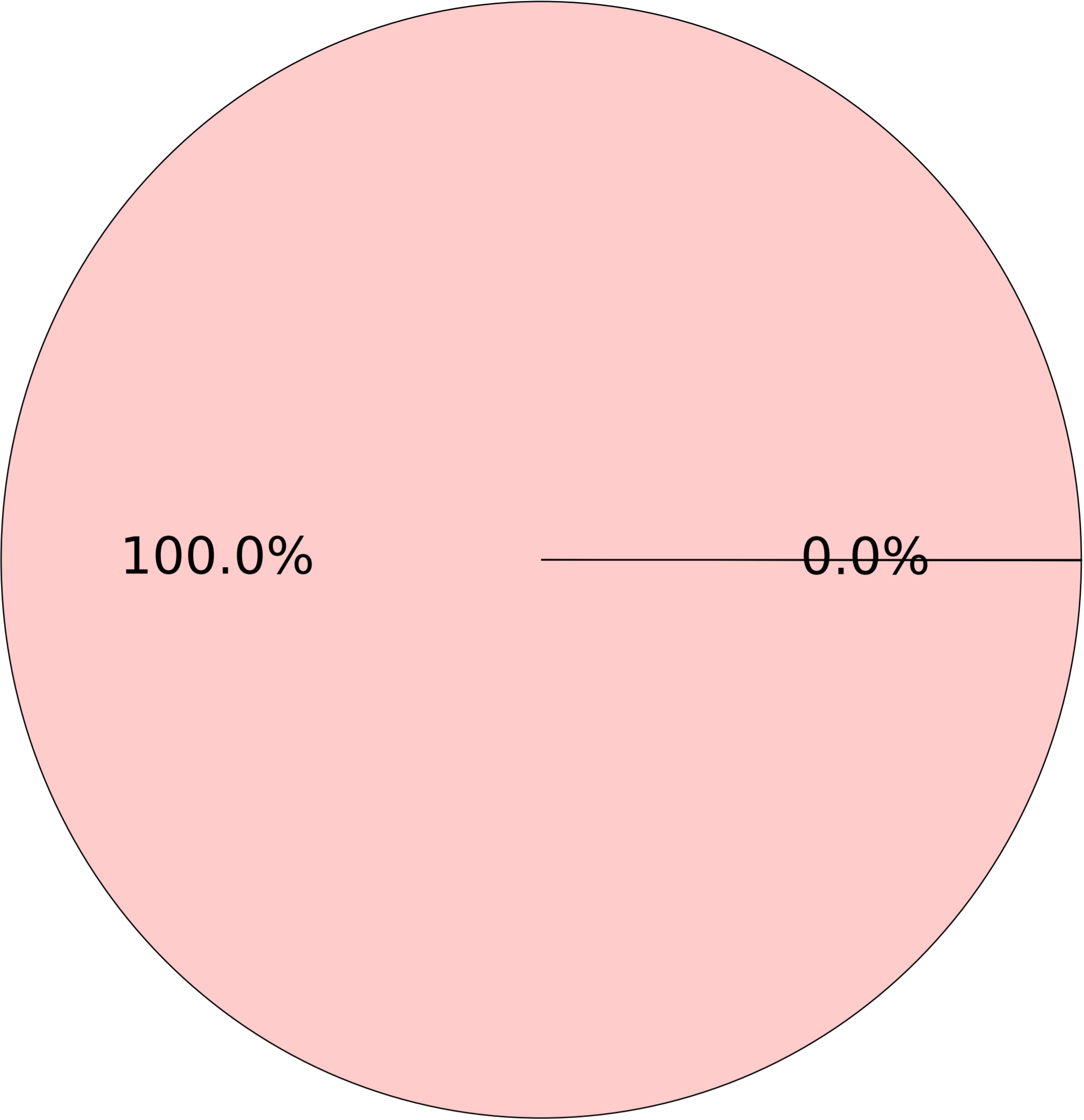

NHEJ  
(7 reads)

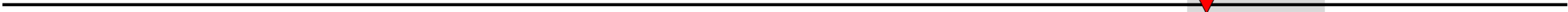

—

Amplicon sequence

—

sgRNA

▼

Predicted Cas9 cleavage site/s

Supplement: Supplementary file 14 — Additional file 14. CRISPResso NHEJ pie charts. [file 12896_2019_565_MOESM14_ESM.zip › CRISPResso_EPSPS-7AS-gRNA3-rep3-negative.pdf]

Unmodified  
(25660 reads)

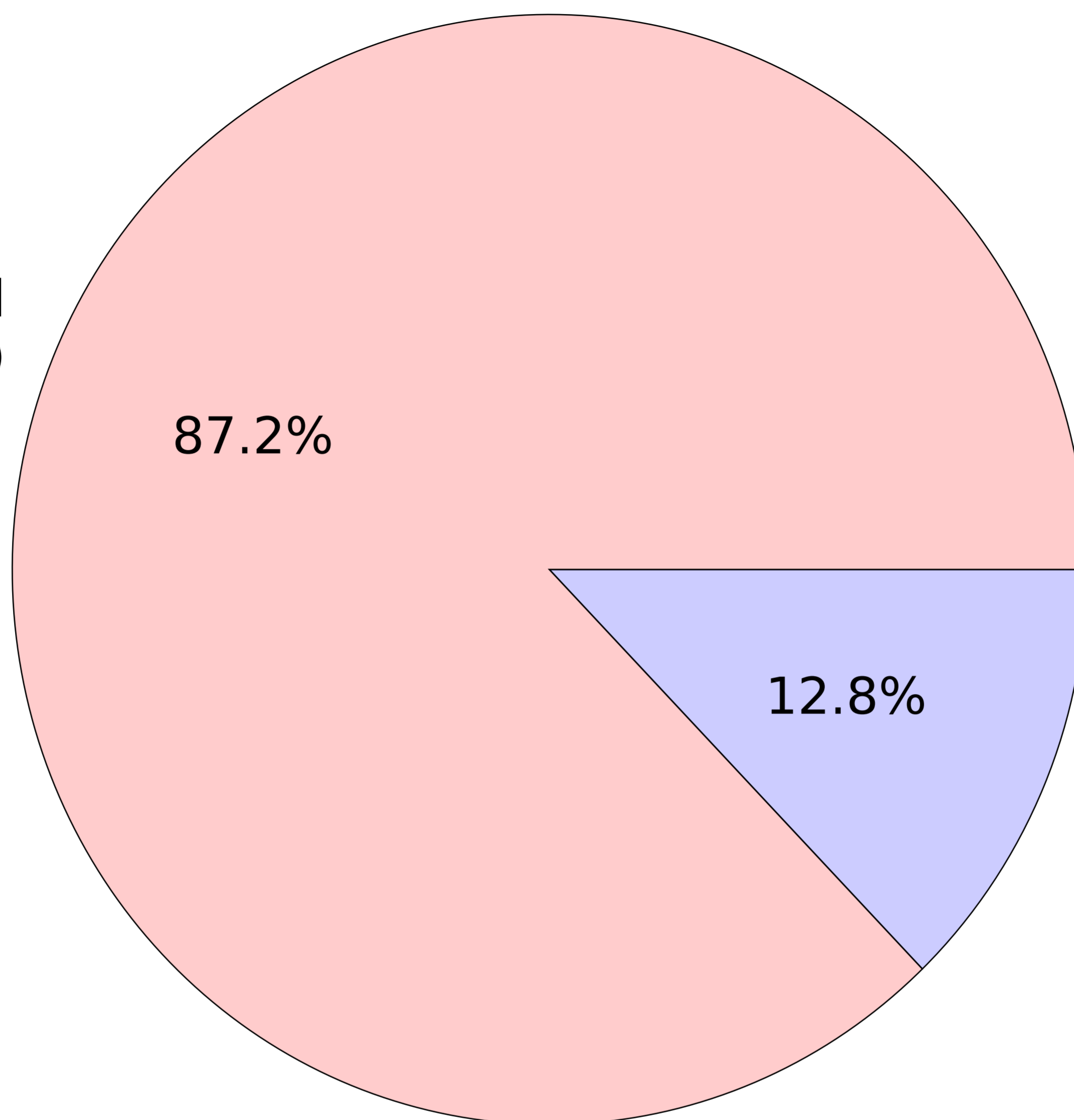

NHEJ  
(3761 reads)

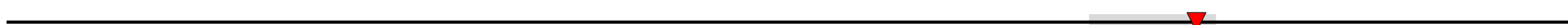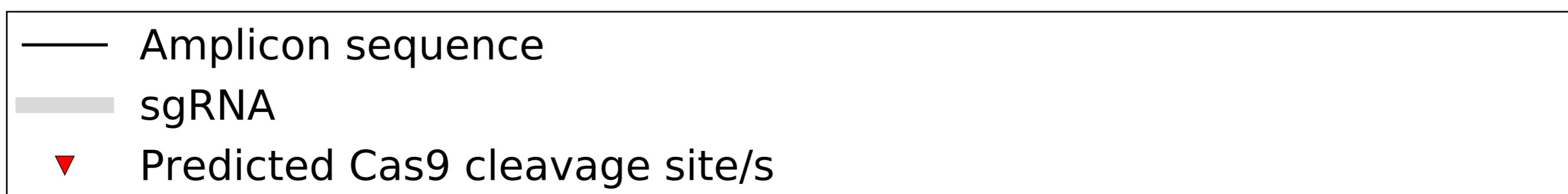

Supplement: Supplementary file 14 — Additional file 14. CRISPResso NHEJ pie charts. [file 12896_2019_565_MOESM14_ESM.zip › CRISPResso_EPSPS-7AS-gRNA4-rep1.pdf]

Unmodified  
(9974 reads)

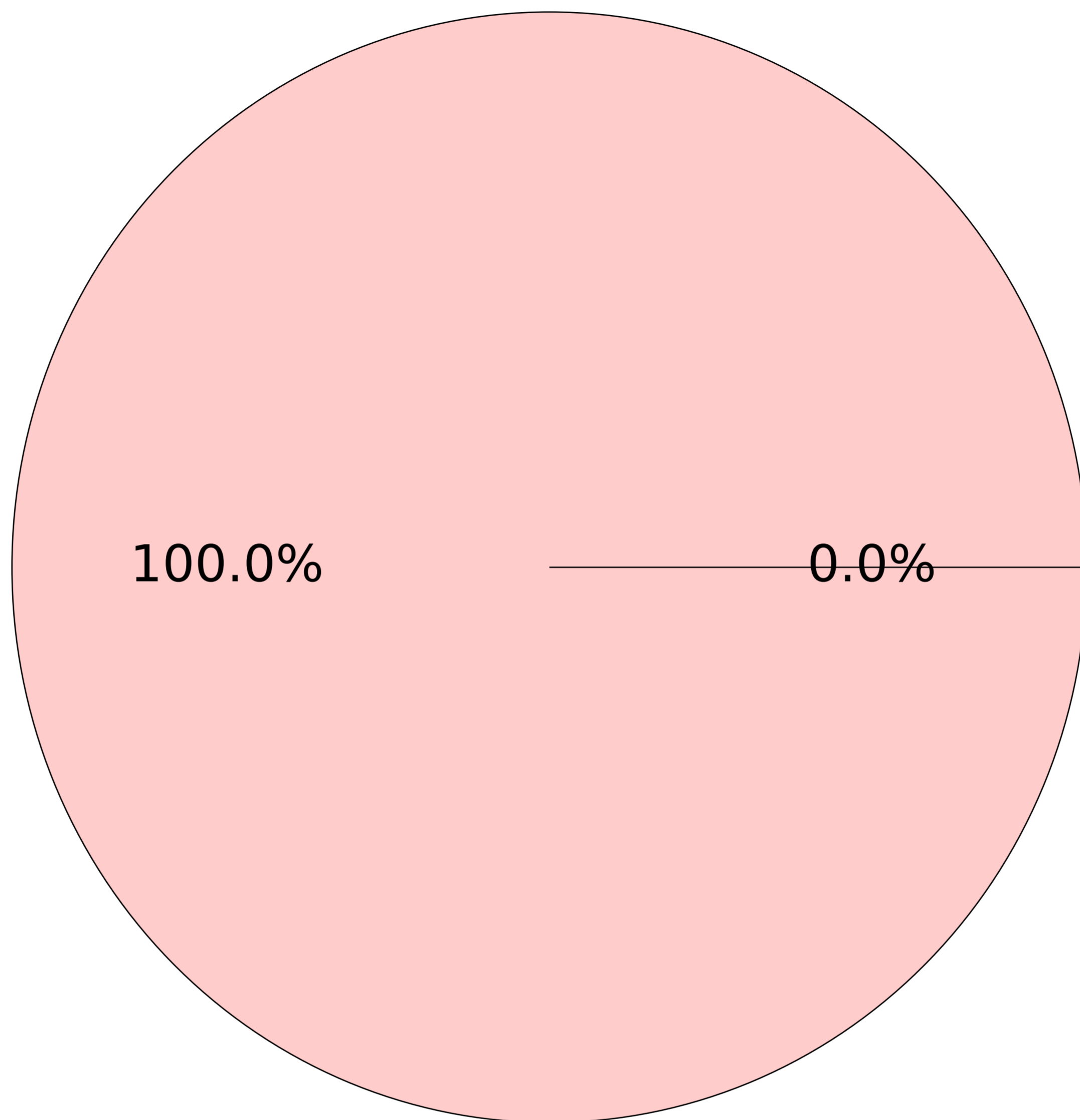

NHEJ  
(0 reads)

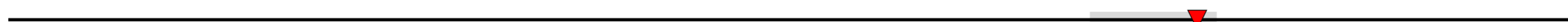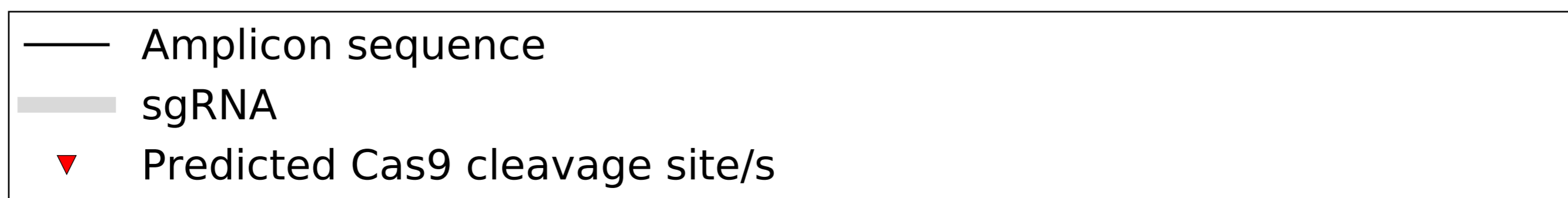

Supplement: Supplementary file 14 — Additional file 14. CRISPResso NHEJ pie charts. [file 12896_2019_565_MOESM14_ESM.zip › CRISPResso_EPSPS-7AS-gRNA4-rep1-negative.pdf]

Unmodified  
(29504 reads)

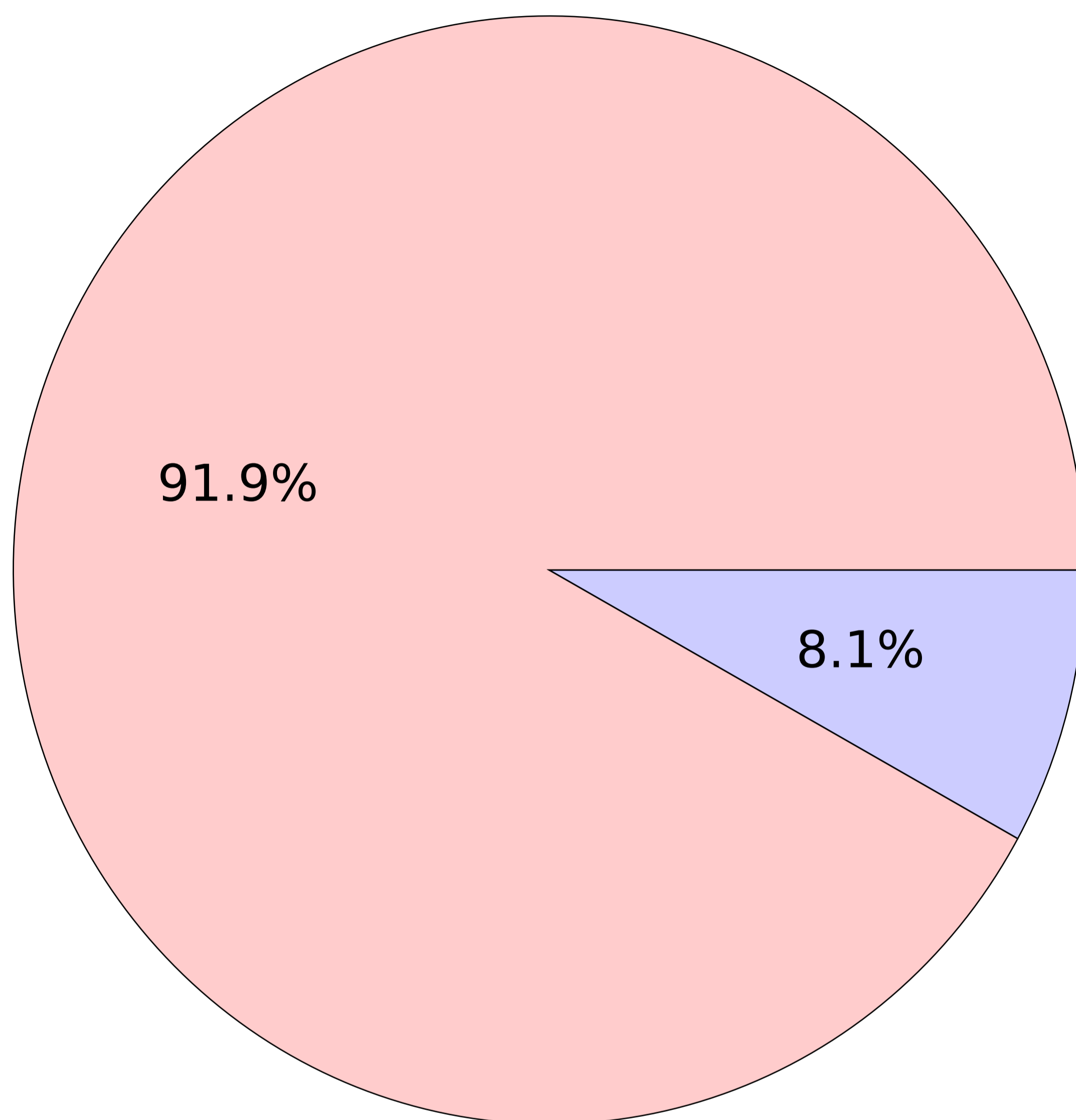

NHEJ  
(2585 reads)

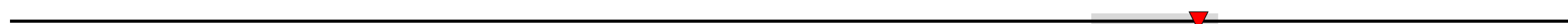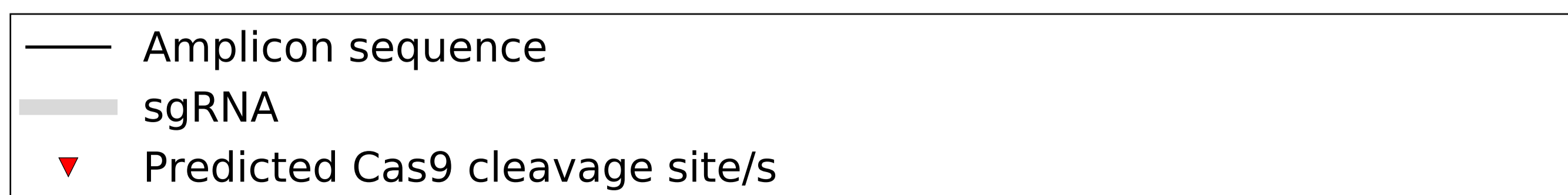

Supplement: Supplementary file 14 — Additional file 14. CRISPResso NHEJ pie charts. [file 12896_2019_565_MOESM14_ESM.zip › CRISPResso_EPSPS-7AS-gRNA4-rep2.pdf]

Unmodified  
(21172 reads)

100.0%

0.0%

NHEJ  
(2 reads)

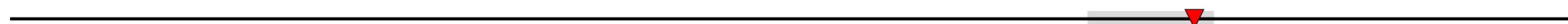

- Amplicon sequence
- sgRNA
- ▼ Predicted Cas9 cleavage site/s

Supplement: Supplementary file 14 — Additional file 14. CRISPResso NHEJ pie charts. [file 12896_2019_565_MOESM14_ESM.zip › CRISPResso_EPSPS-7AS-gRNA4-rep2-negative.pdf]

Unmodified  
(20609 reads)

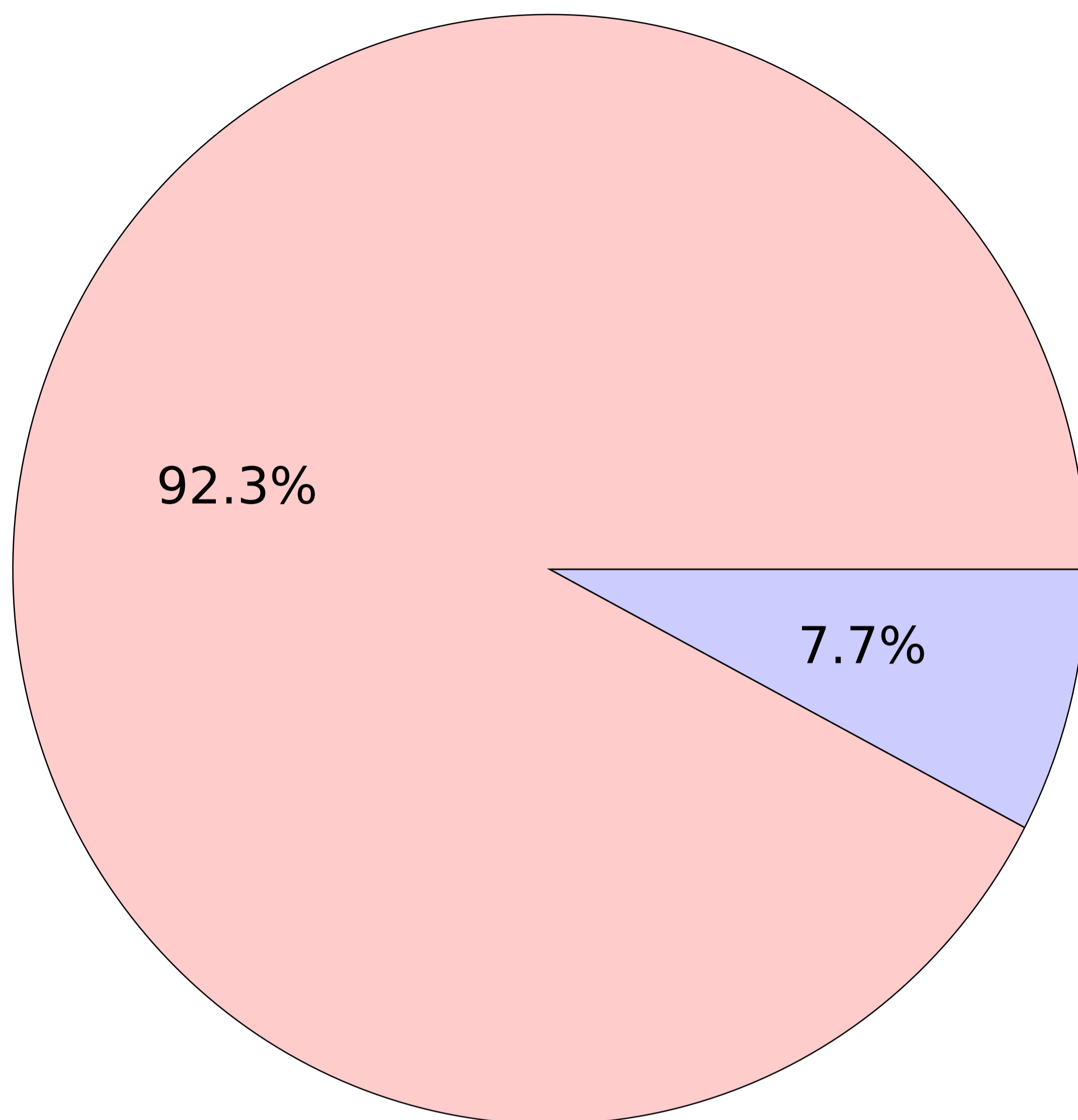

NHEJ  
(1721 reads)

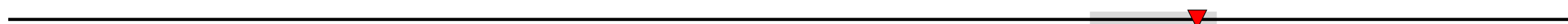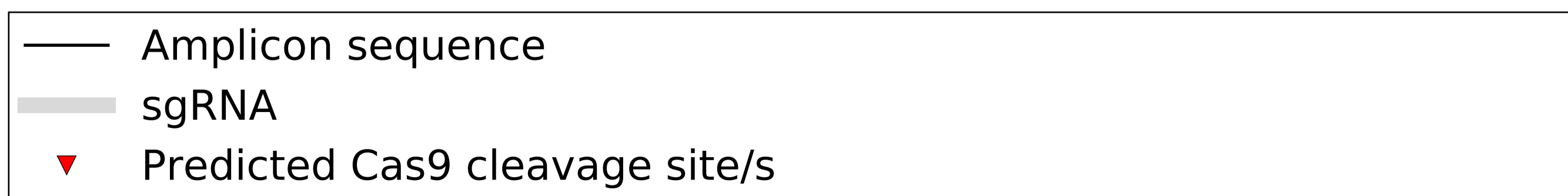

Supplement: Supplementary file 14 — Additional file 14. CRISPResso NHEJ pie charts. [file 12896_2019_565_MOESM14_ESM.zip › CRISPResso_EPSPS-7AS-gRNA4-rep3.pdf]

Unmodified  
(28704 reads)

100.0%

0.0%

NHEJ  
(3 reads)

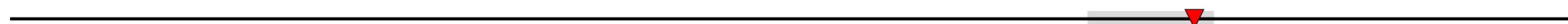

- Amplicon sequence
- sgRNA
- ▼ Predicted Cas9 cleavage site/s

Supplement: Supplementary file 14 — Additional file 14. CRISPResso NHEJ pie charts. [file 12896_2019_565_MOESM14_ESM.zip › CRISPResso_EPSPS-7AS-gRNA4-rep3-negative.pdf]

Unmodified  
(29374 reads)

82.4%

17.6%

NHEJ  
(6294 reads)

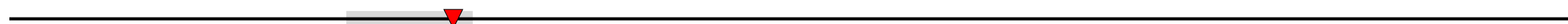

- Amplicon sequence
- sgRNA
- ▼ Predicted Cas9 cleavage site/s

Supplement: Supplementary file 14 — Additional file 14. CRISPResso NHEJ pie charts. [file 12896_2019_565_MOESM14_ESM.zip › CRISPResso_EPSPS-7AS-gRNA5-rep1.pdf]

Unmodified  
(9972 reads)

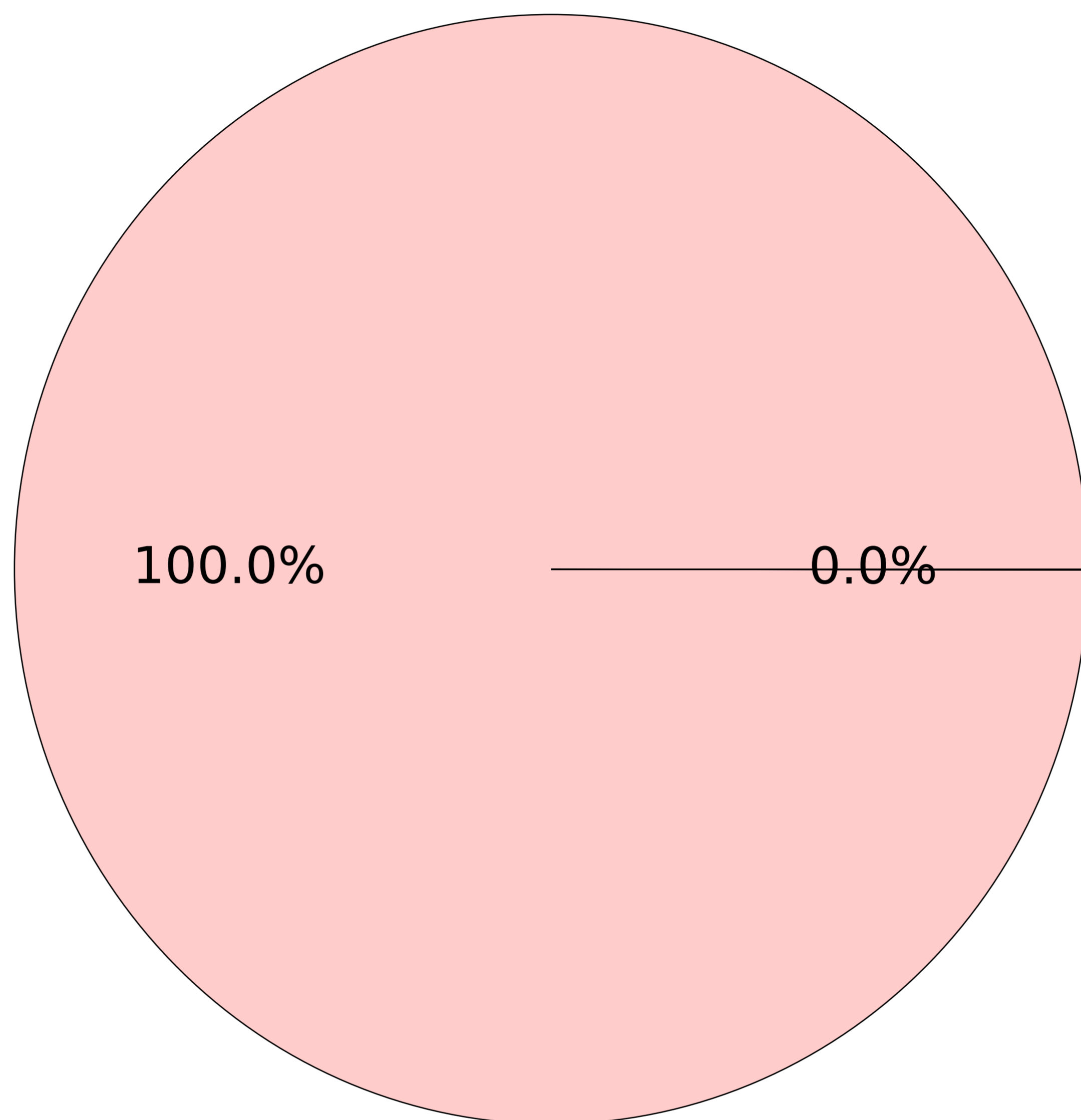

NHEJ  
(2 reads)

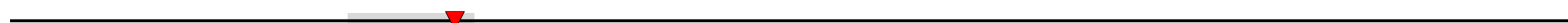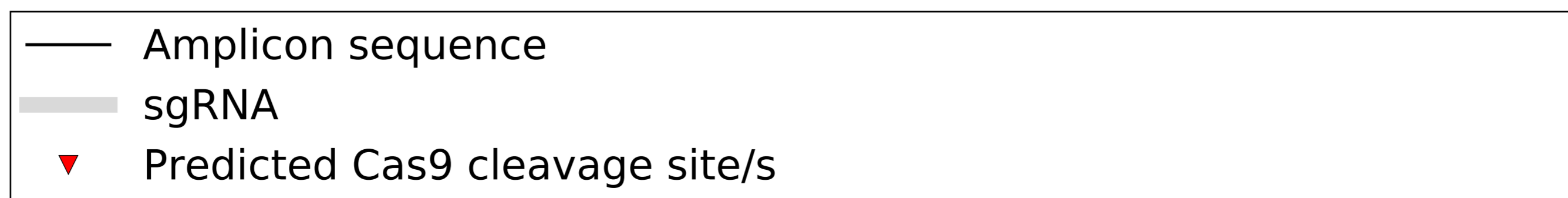

Supplement: Supplementary file 14 — Additional file 14. CRISPResso NHEJ pie charts. [file 12896_2019_565_MOESM14_ESM.zip › CRISPResso_EPSPS-7AS-gRNA5-rep1-negative.pdf]

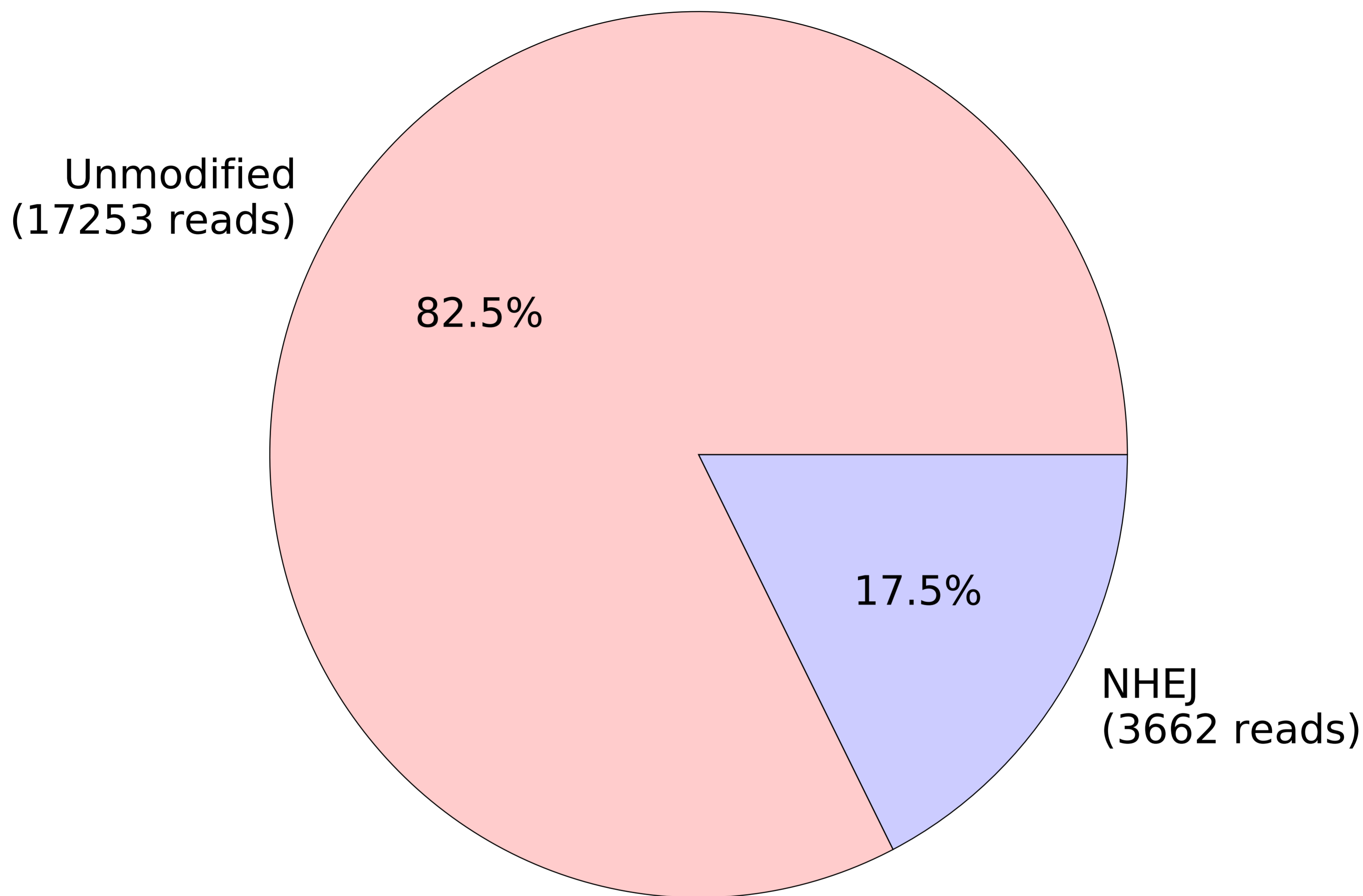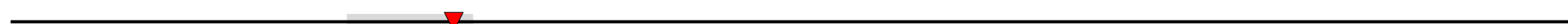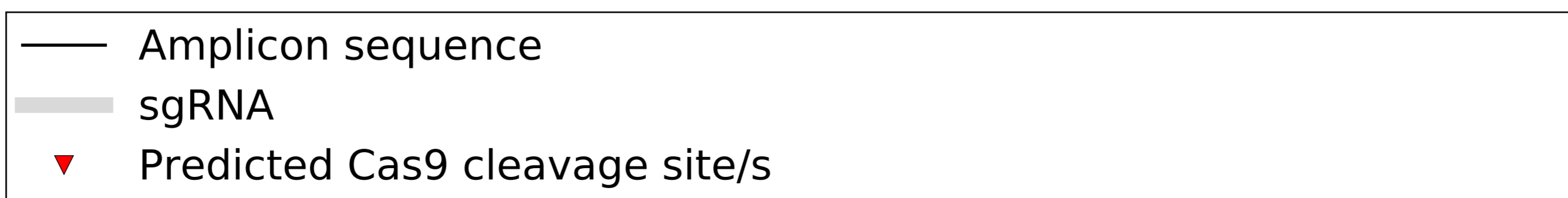

Supplement: Supplementary file 14 — Additional file 14. CRISPResso NHEJ pie charts. [file 12896_2019_565_MOESM14_ESM.zip › CRISPResso_EPSPS-7AS-gRNA5-rep2.pdf]

Unmodified  
(21168 reads)

100.0%

0.0%

NHEJ  
(6 reads)

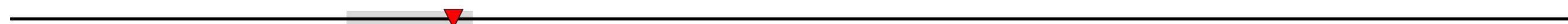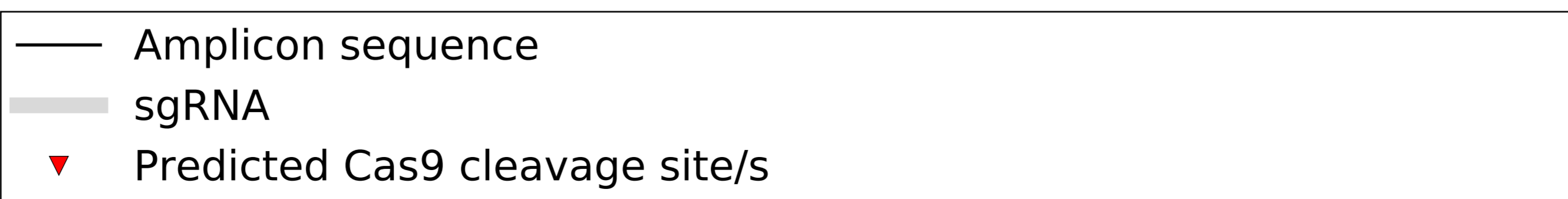

Supplement: Supplementary file 14 — Additional file 14. CRISPResso NHEJ pie charts. [file 12896_2019_565_MOESM14_ESM.zip › CRISPResso_EPSPS-7AS-gRNA5-rep2-negative.pdf]

Unmodified  
(17256 reads)

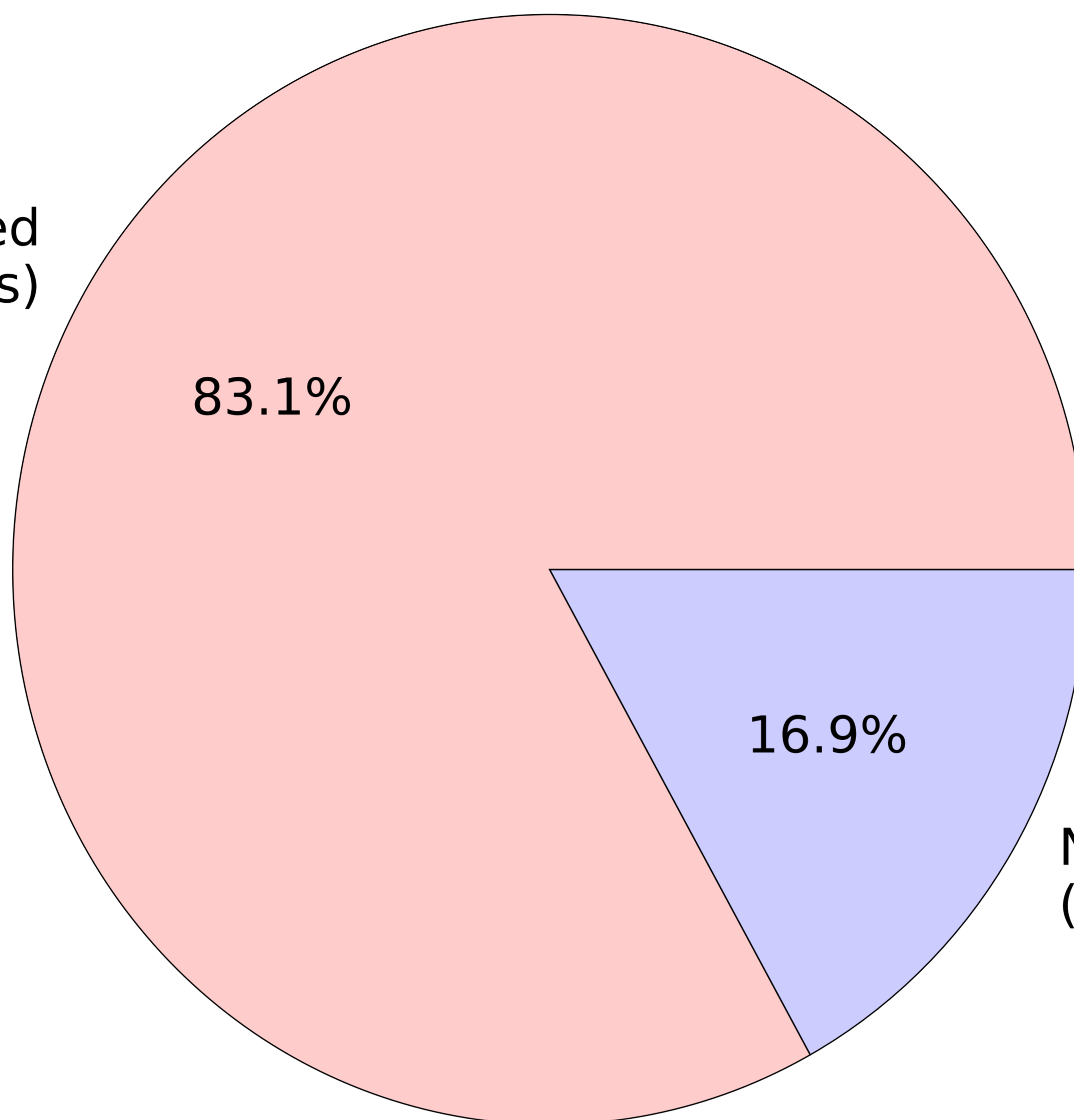

NHEJ  
(3518 reads)

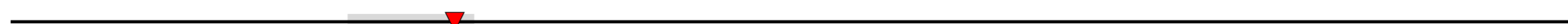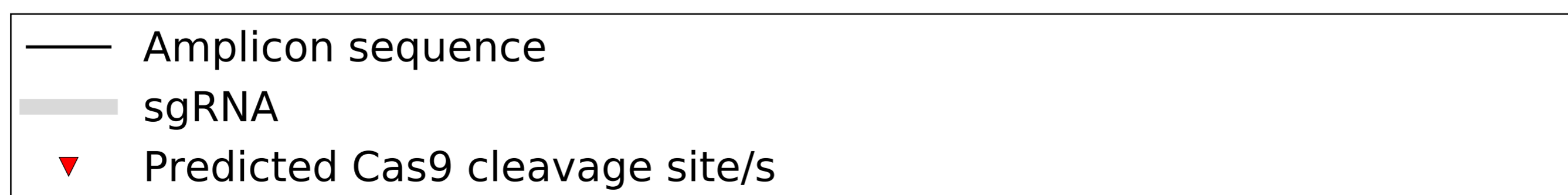

Supplement: Supplementary file 14 — Additional file 14. CRISPResso NHEJ pie charts. [file 12896_2019_565_MOESM14_ESM.zip › CRISPResso_EPSPS-7AS-gRNA5-rep3.pdf]

Unmodified  
(28702 reads)

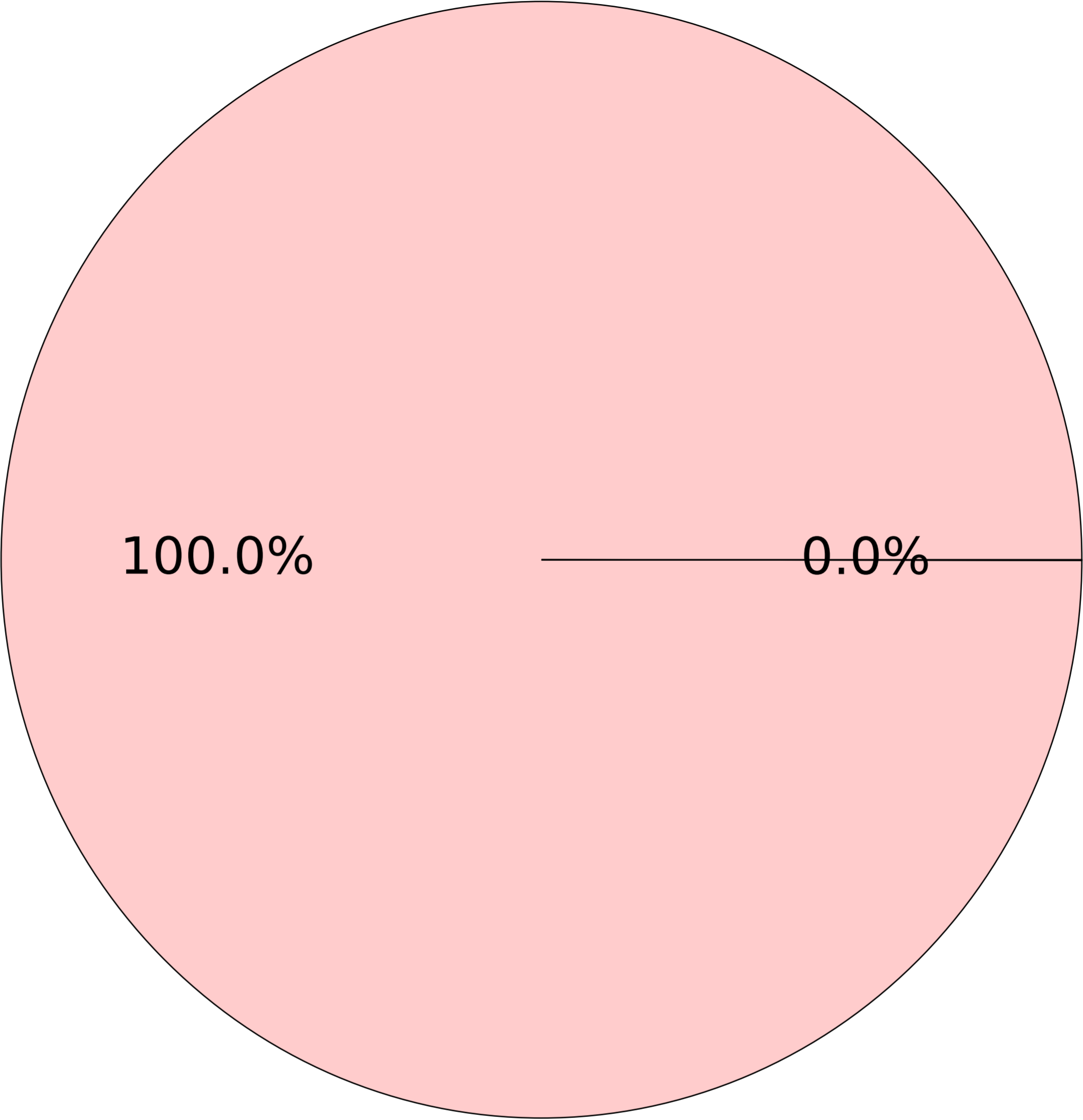

NHEJ  
(5 reads)

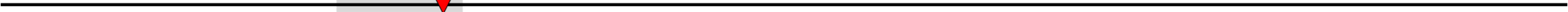

—

Amplicon sequence

—

sgRNA

▼

Predicted Cas9 cleavage site/s

Supplement: Supplementary file 14 — Additional file 14. CRISPResso NHEJ pie charts. [file 12896_2019_565_MOESM14_ESM.zip › CRISPResso_EPSPS-7AS-gRNA5-rep3-negative.pdf]

Unmodified  
(17489 reads)

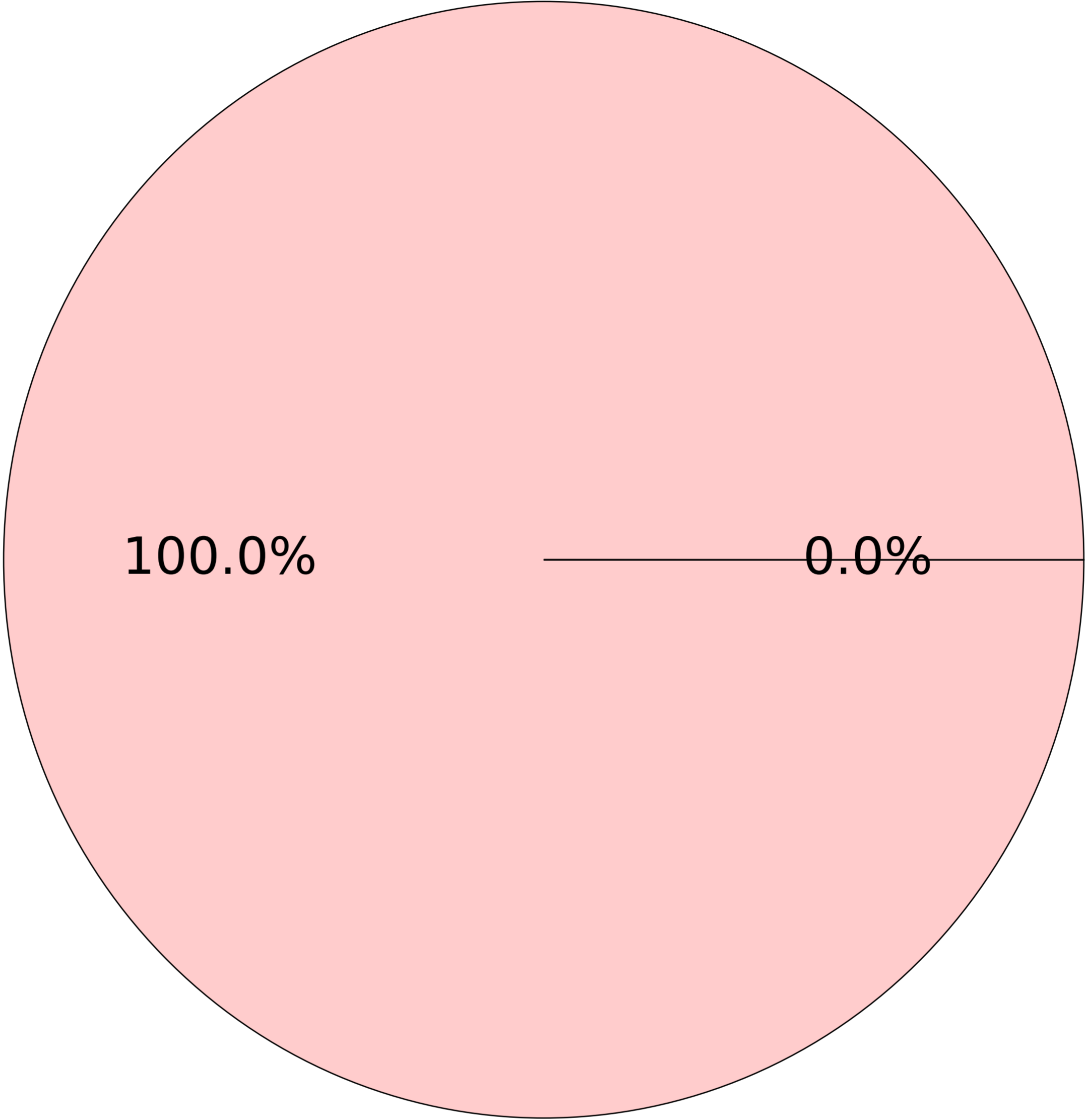

NHEJ  
(1 reads)

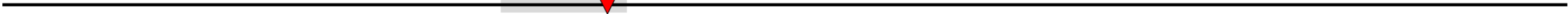

—

Amplicon sequence

—

sgRNA

▼

Predicted Cas9 cleavage site/s

Supplement: Supplementary file 14 — Additional file 14. CRISPResso NHEJ pie charts. [file 12896_2019_565_MOESM14_ESM.zip › CRISPResso_EPSPS-7AS-gRNA6-rep1.pdf]

Unmodified  
(9973 reads)

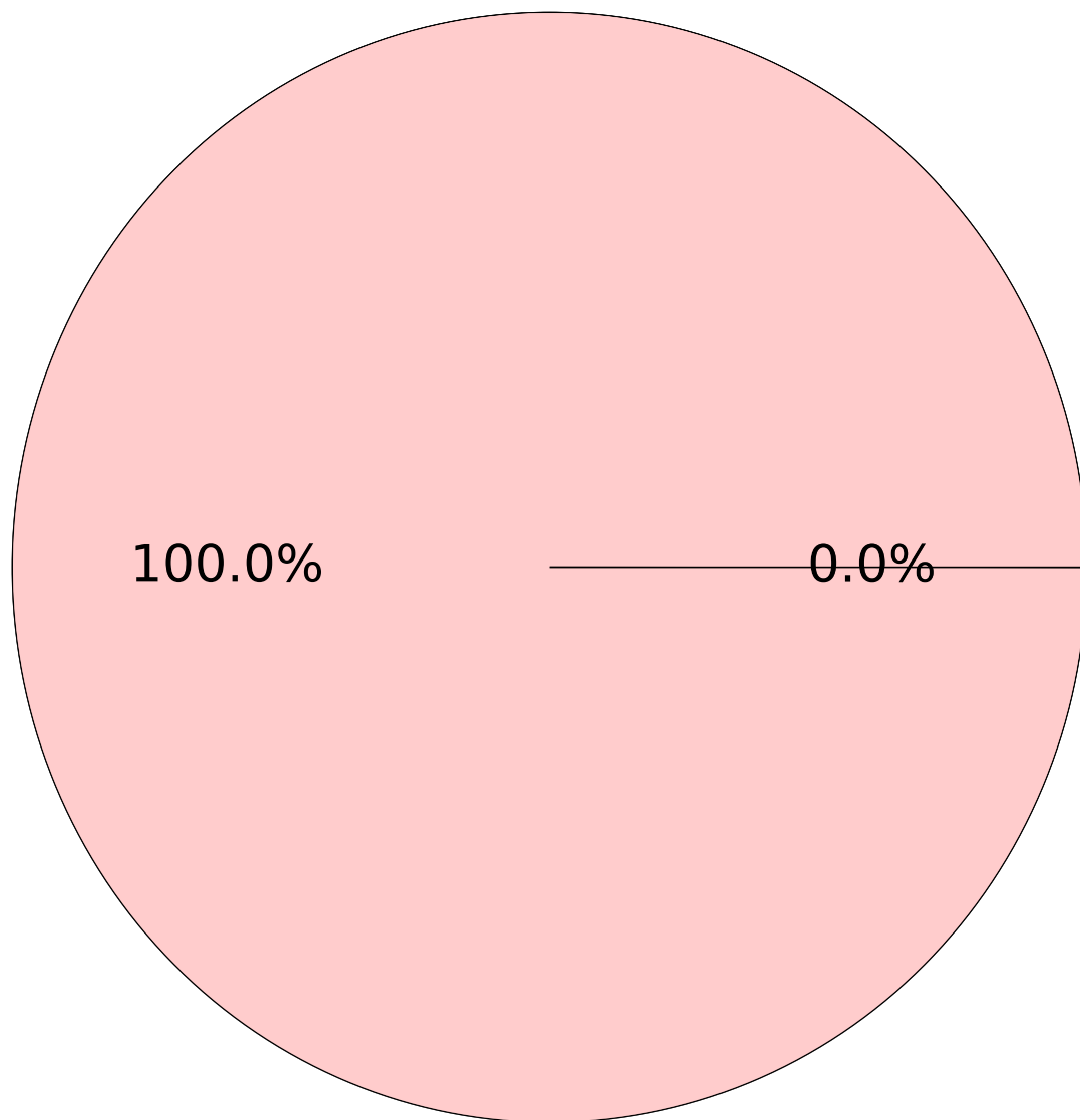

NHEJ  
(1 reads)

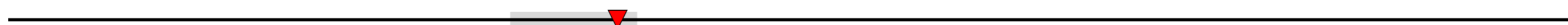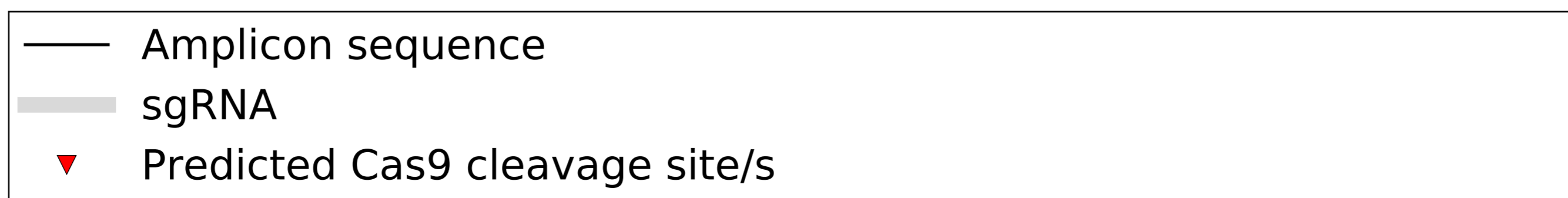

Supplement: Supplementary file 14 — Additional file 14. CRISPResso NHEJ pie charts. [file 12896_2019_565_MOESM14_ESM.zip › CRISPResso_EPSPS-7AS-gRNA6-rep1-negative.pdf]

Unmodified  
(17954 reads)

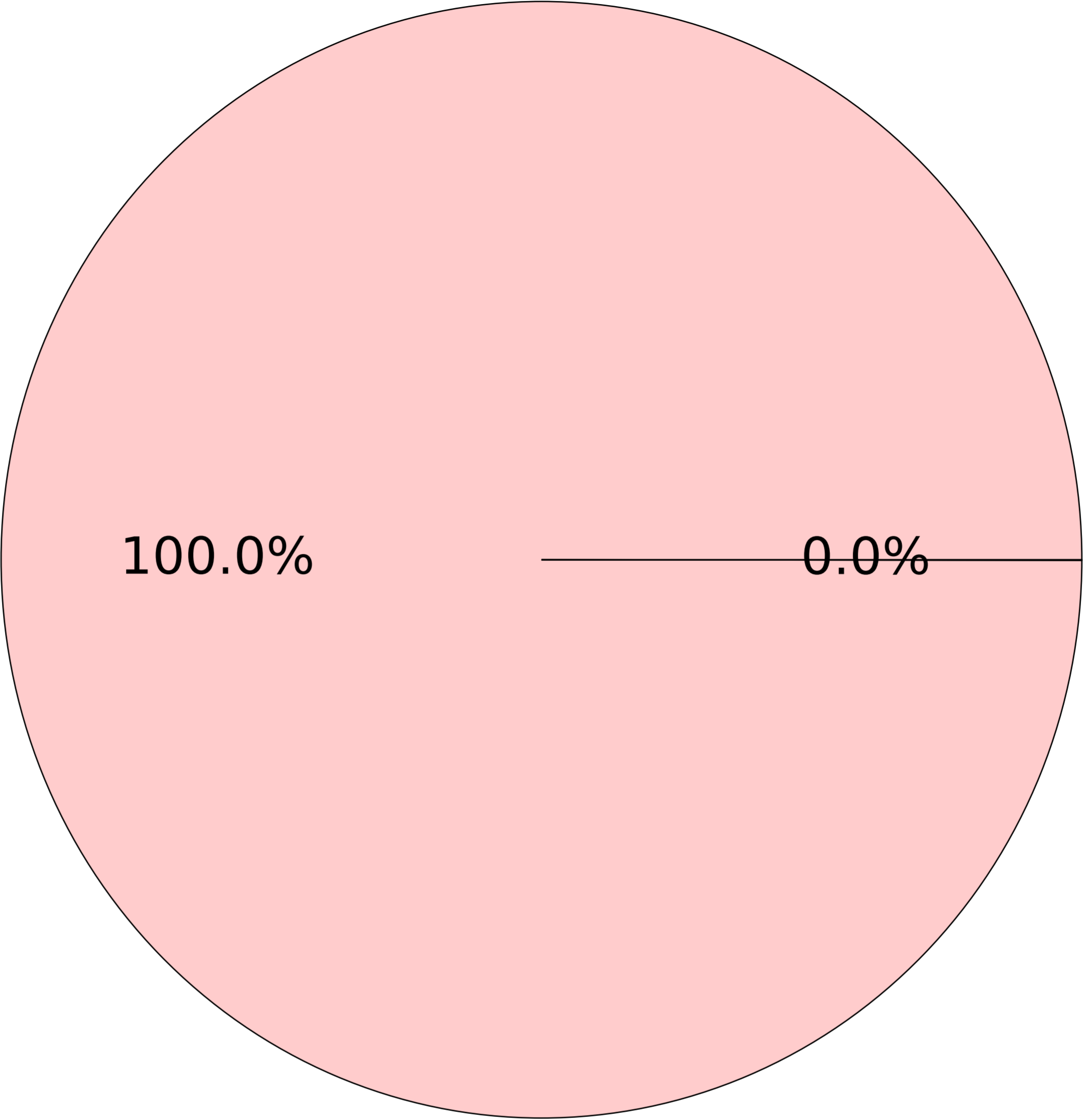

NHEJ  
(3 reads)

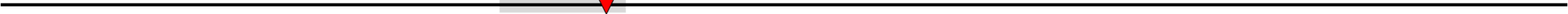

—

Amplicon sequence

—

sgRNA

▼

Predicted Cas9 cleavage site/s

Supplement: Supplementary file 14 — Additional file 14. CRISPResso NHEJ pie charts. [file 12896_2019_565_MOESM14_ESM.zip › CRISPResso_EPSPS-7AS-gRNA6-rep2.pdf]

Unmodified  
(21170 reads)

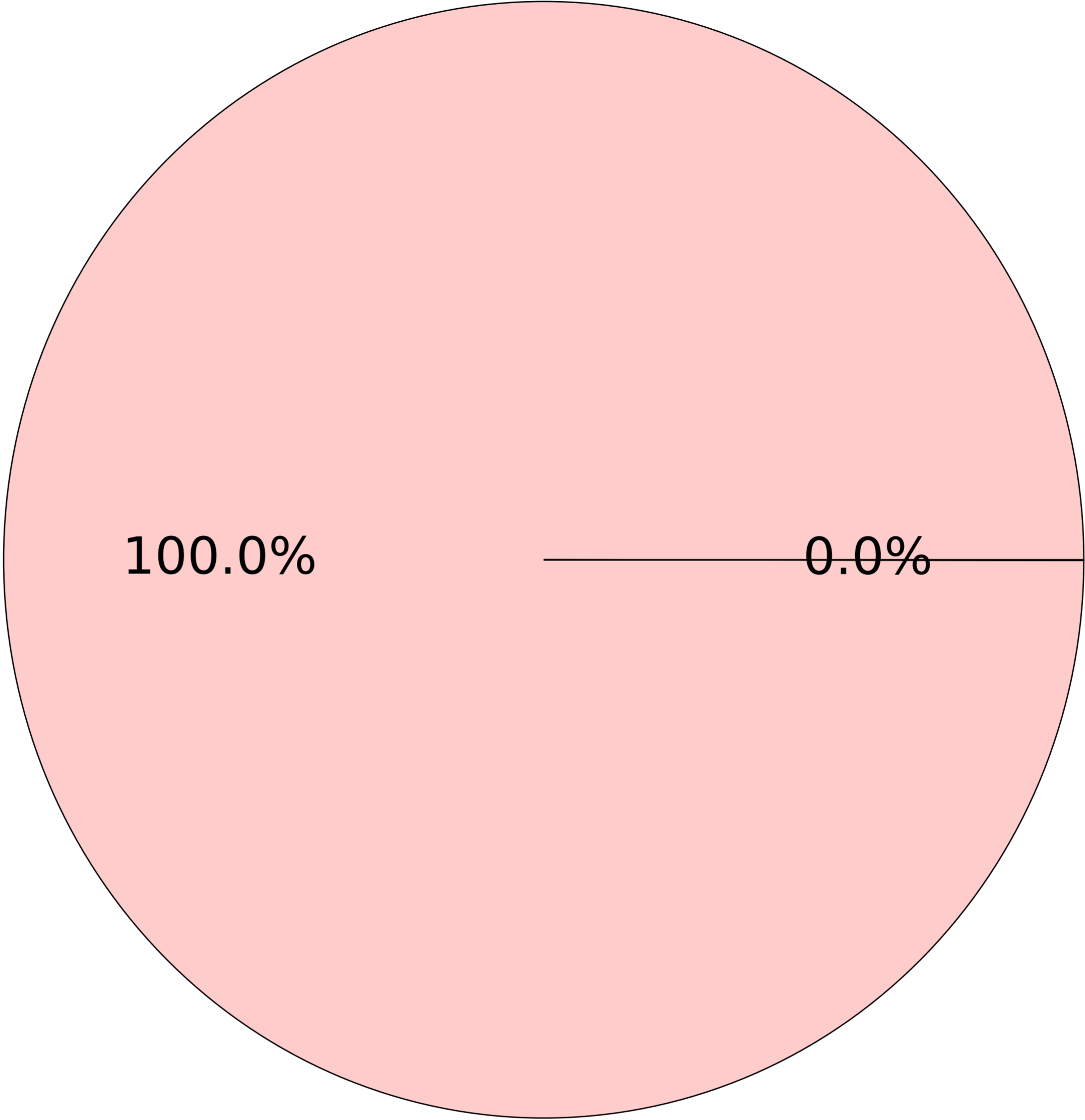

NHEJ  
(4 reads)

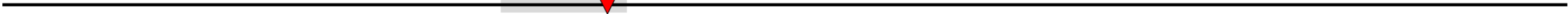

—

Amplicon sequence

—

sgRNA

▼

Predicted Cas9 cleavage site/s

Supplement: Supplementary file 14 — Additional file 14. CRISPResso NHEJ pie charts. [file 12896_2019_565_MOESM14_ESM.zip › CRISPResso_EPSPS-7AS-gRNA6-rep2-negative.pdf]

Unmodified  
(19515 reads)

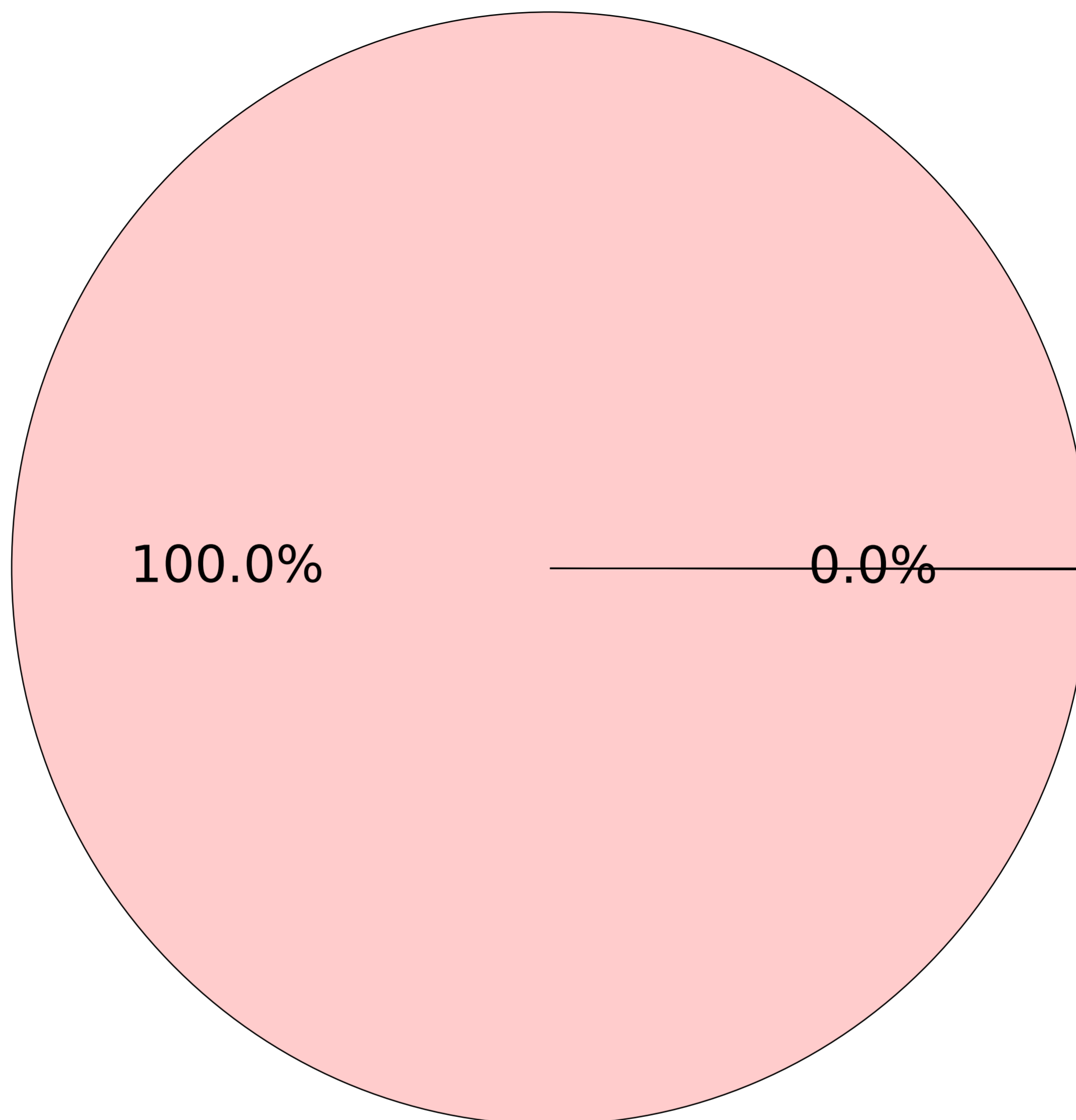

NHEJ  
(7 reads)

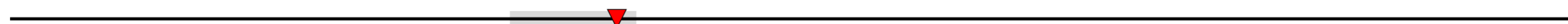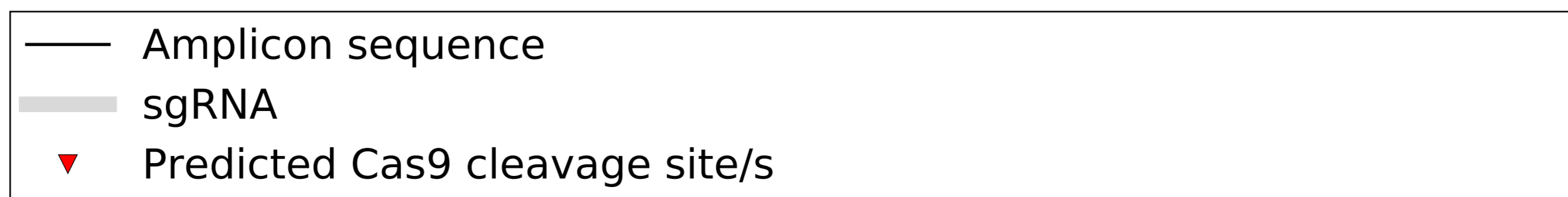

Supplement: Supplementary file 14 — Additional file 14. CRISPResso NHEJ pie charts. [file 12896_2019_565_MOESM14_ESM.zip › CRISPResso_EPSPS-7AS-gRNA6-rep3.pdf]

Unmodified  
(28698 reads)

100.0%

0.0%

NHEJ  
(9 reads)

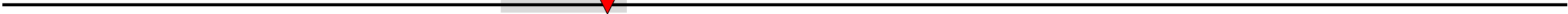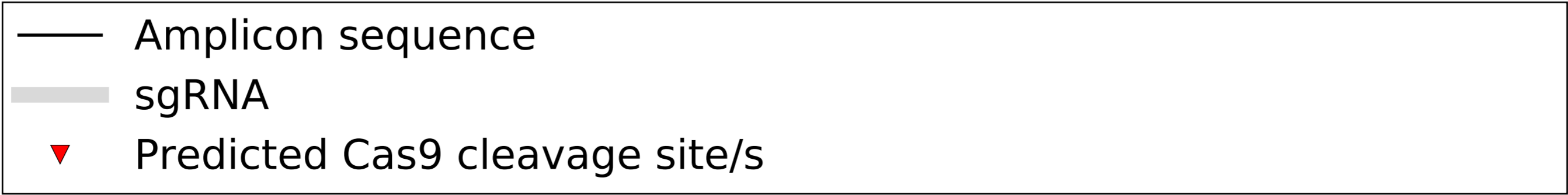

Supplement: Supplementary file 14 — Additional file 14. CRISPResso NHEJ pie charts. [file 12896_2019_565_MOESM14_ESM.zip › CRISPResso_EPSPS-7AS-gRNA6-rep3-negative.pdf]

Unmodified  
(27835 reads)

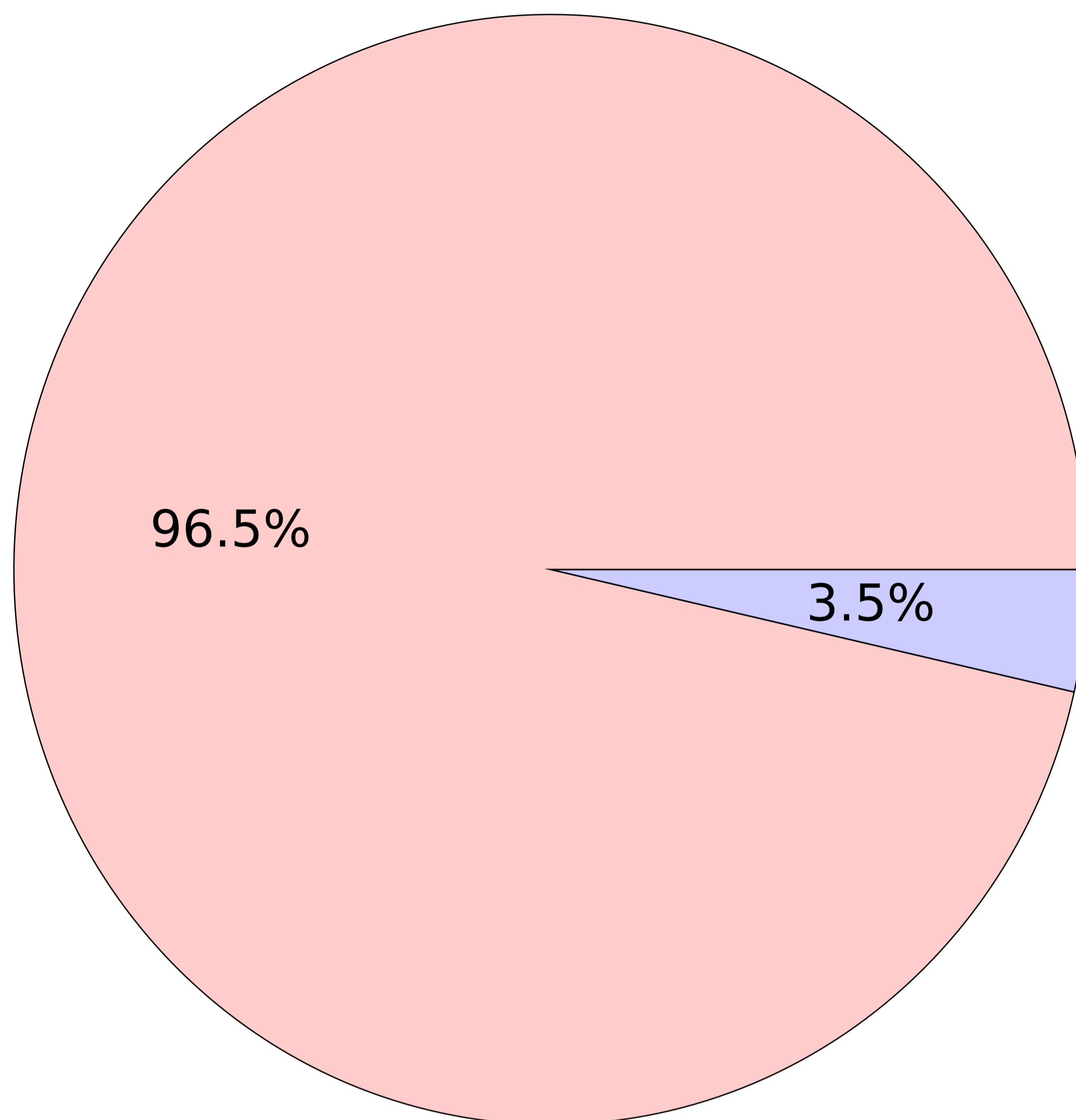

NHEJ  
(1022 reads)

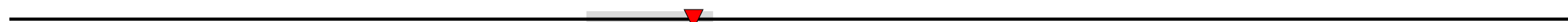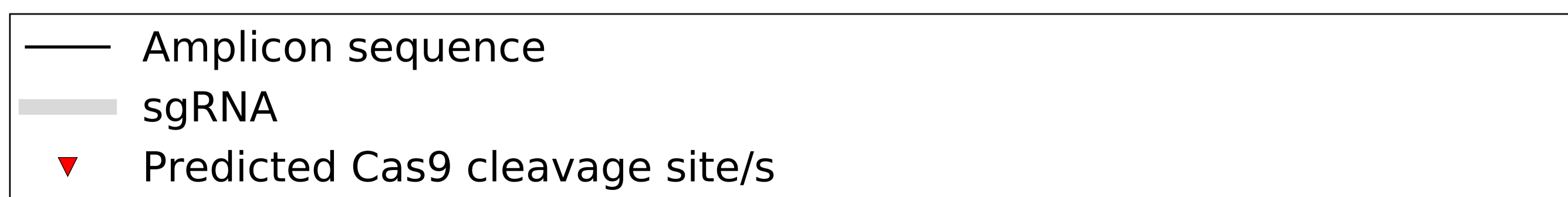

Supplement: Supplementary file 14 — Additional file 14. CRISPResso NHEJ pie charts. [file 12896_2019_565_MOESM14_ESM.zip › CRISPResso_EPSPS-7AS-gRNA7-rep1.pdf]

Unmodified  
(9972 reads)

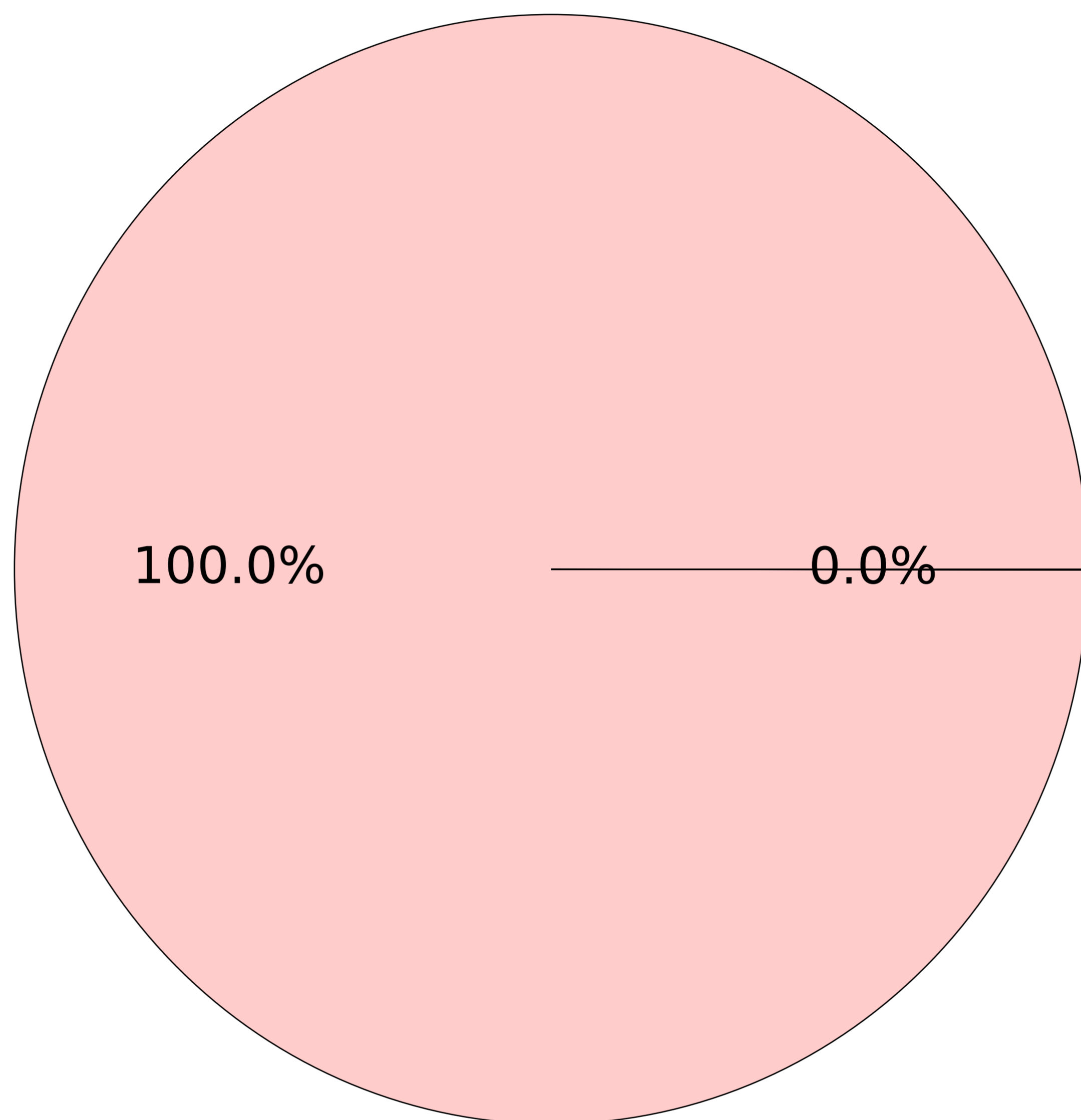

NHEJ  
(2 reads)

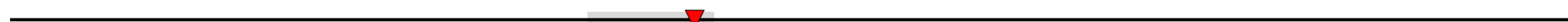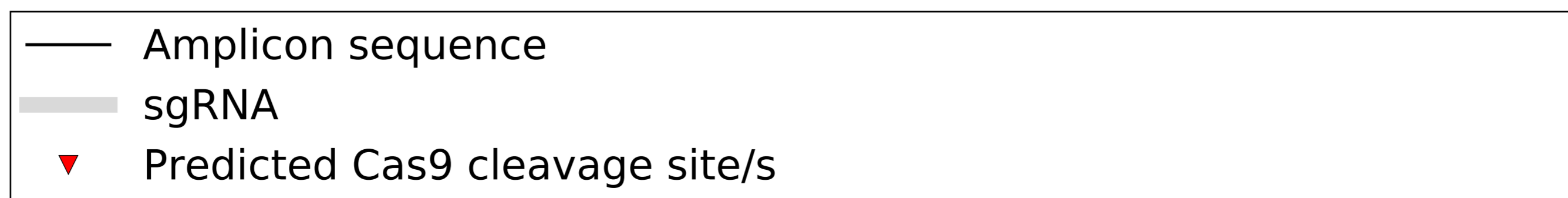

Supplement: Supplementary file 14 — Additional file 14. CRISPResso NHEJ pie charts. [file 12896_2019_565_MOESM14_ESM.zip › CRISPResso_EPSPS-7AS-gRNA7-rep1-negative.pdf]

Unmodified  
(27058 reads)

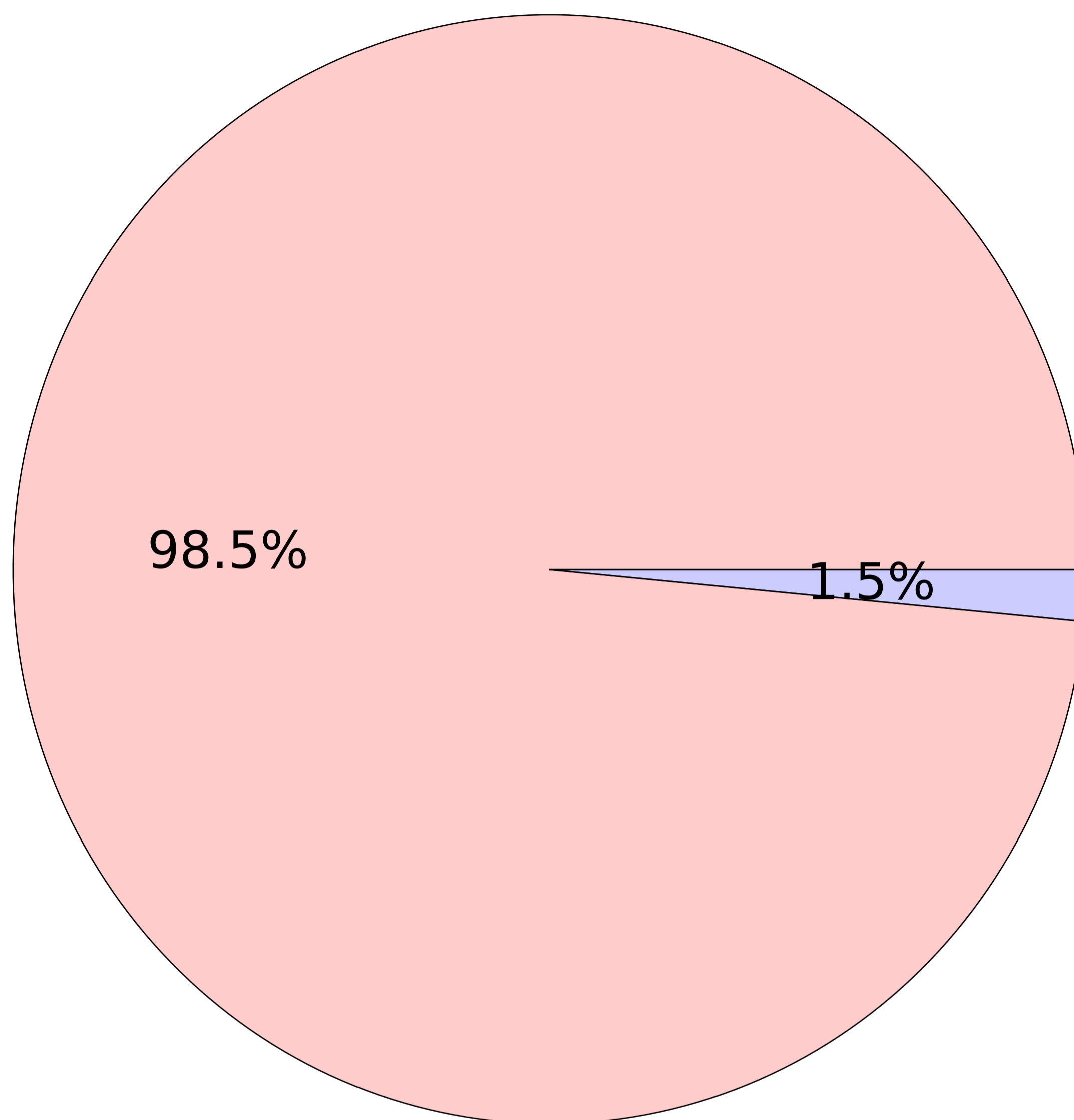

NHEJ  
(410 reads)

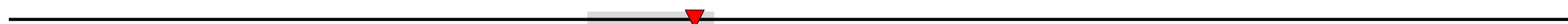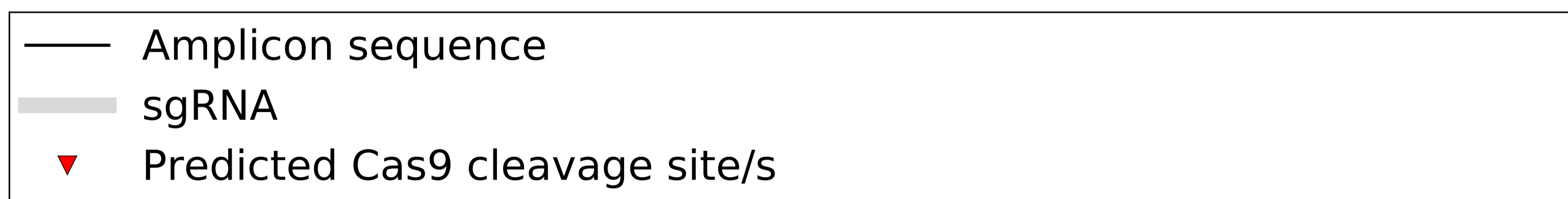

Supplement: Supplementary file 14 — Additional file 14. CRISPResso NHEJ pie charts. [file 12896_2019_565_MOESM14_ESM.zip › CRISPResso_EPSPS-7AS-gRNA7-rep2.pdf]

Unmodified  
(21171 reads)

100.0%

0.0%

NHEJ  
(3 reads)

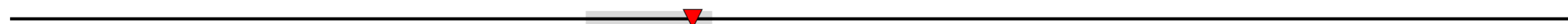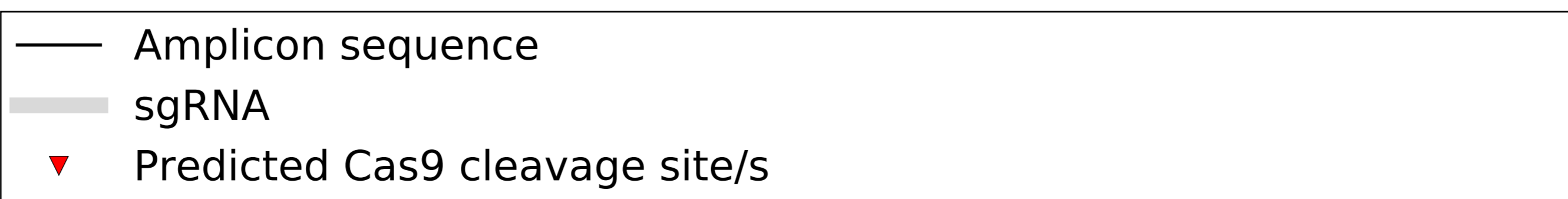

Supplement: Supplementary file 14 — Additional file 14. CRISPResso NHEJ pie charts. [file 12896_2019_565_MOESM14_ESM.zip › CRISPResso_EPSPS-7AS-gRNA7-rep2-negative.pdf]

Unmodified  
(24010 reads)

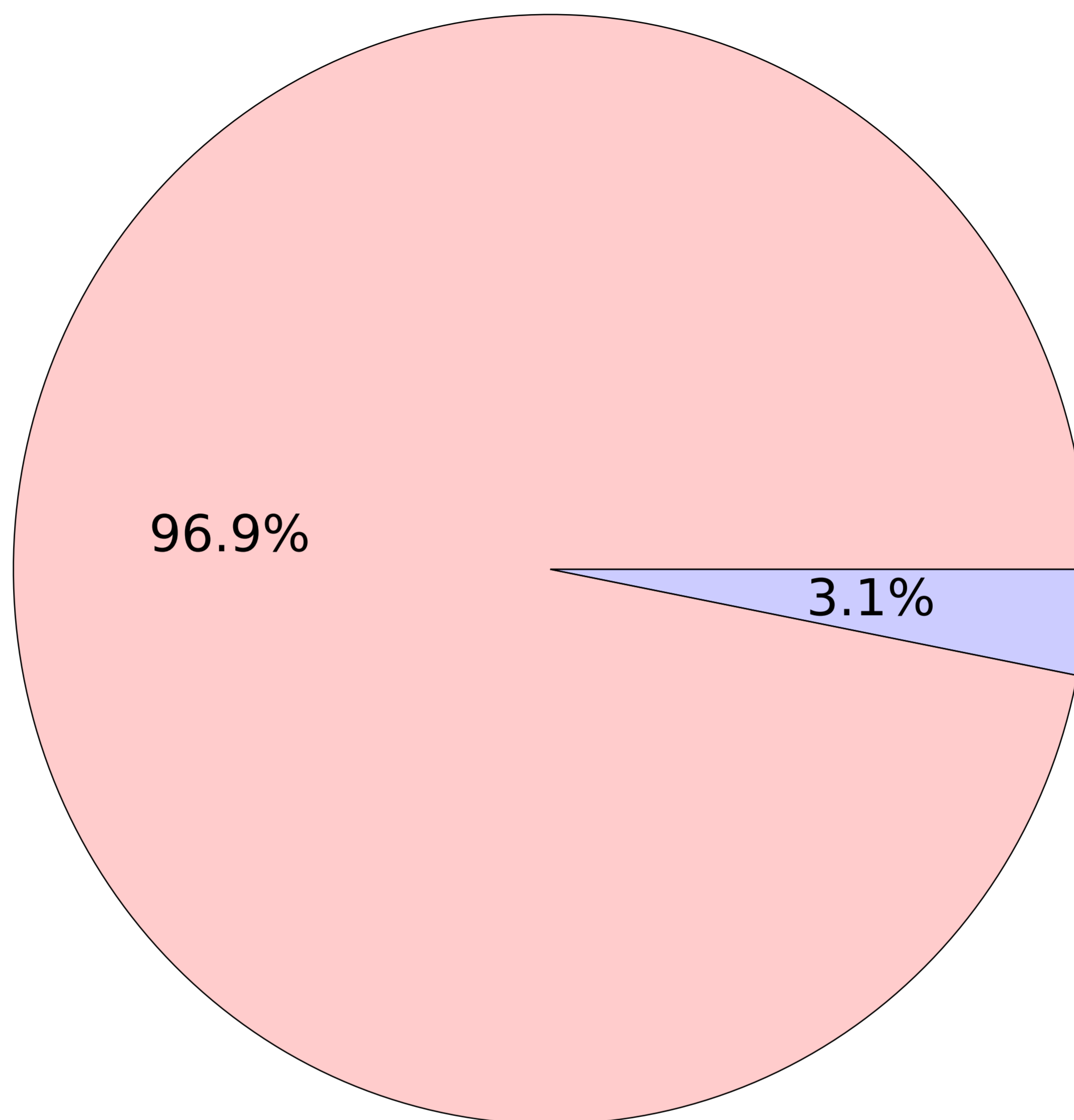

NHEJ  
(759 reads)

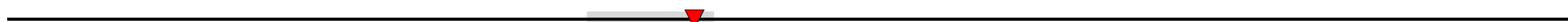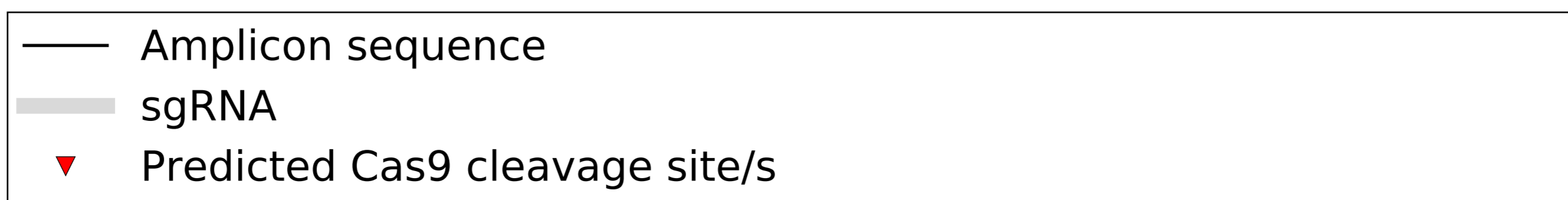

Supplement: Supplementary file 14 — Additional file 14. CRISPResso NHEJ pie charts. [file 12896_2019_565_MOESM14_ESM.zip › CRISPResso_EPSPS-7AS-gRNA7-rep3.pdf]

Unmodified  
(28700 reads)

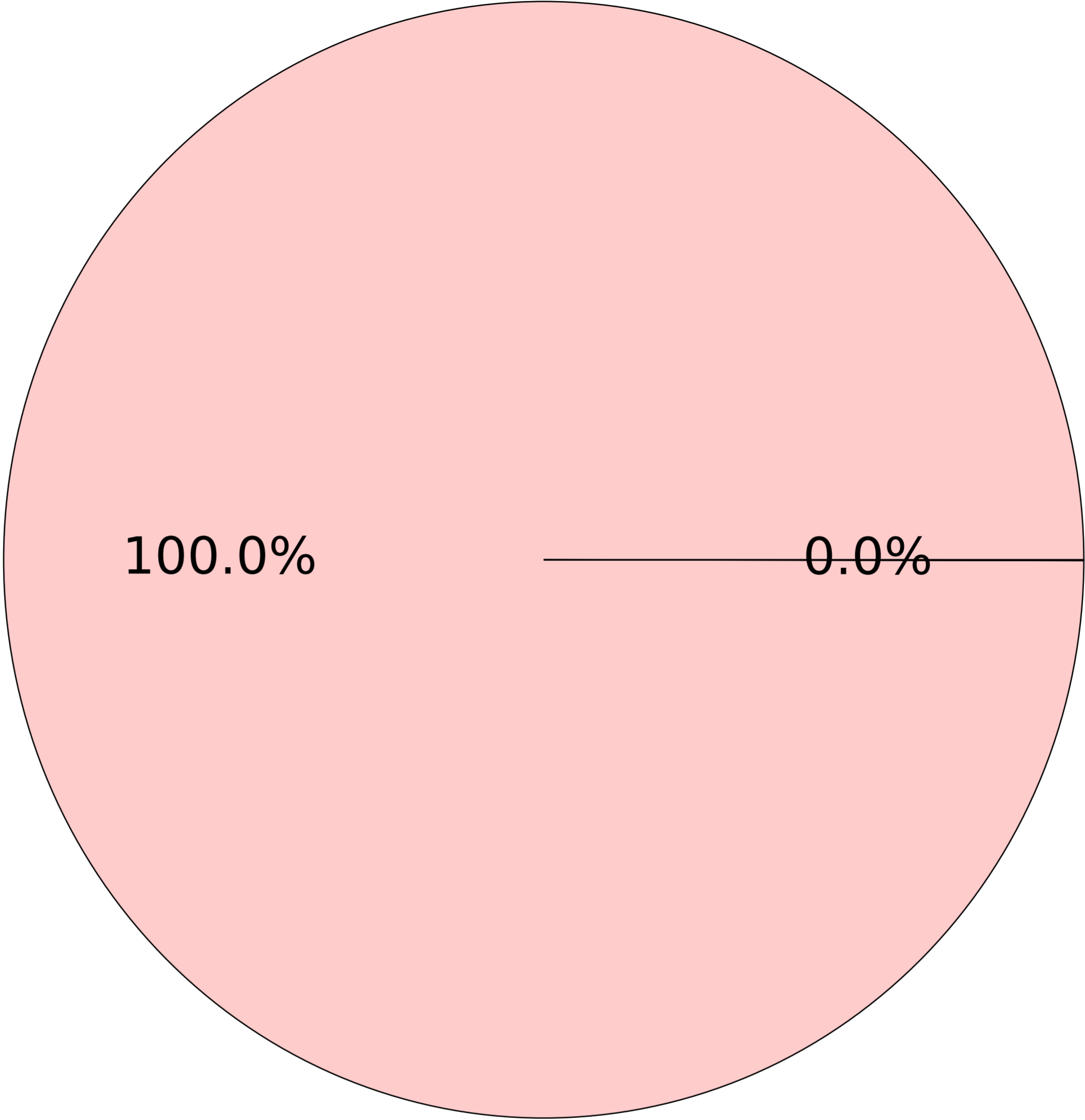

NHEJ  
(7 reads)

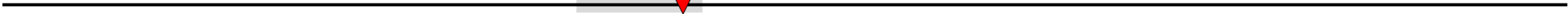

—

Amplicon sequence

—

sgRNA

▼

Predicted Cas9 cleavage site/s

Supplement: Supplementary file 14 — Additional file 14. CRISPResso NHEJ pie charts. [file 12896_2019_565_MOESM14_ESM.zip › CRISPResso_EPSPS-7AS-gRNA7-rep3-negative.pdf]

Unmodified  
(22958 reads)

100.0%

0.0%

NHEJ  
(6 reads)

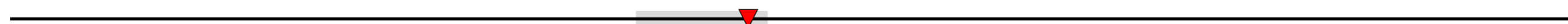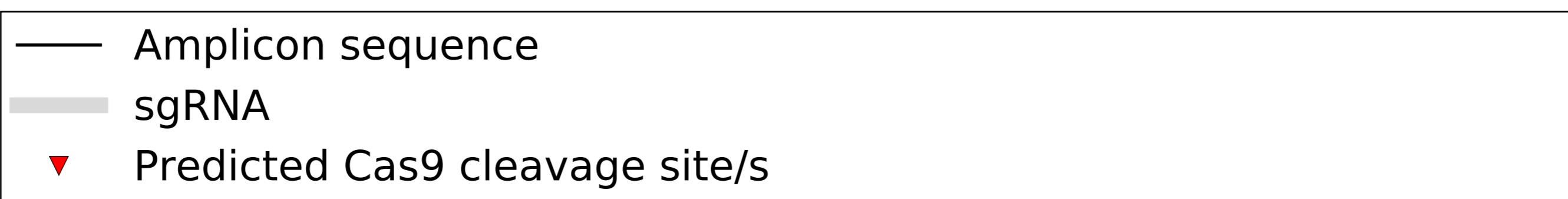

Supplement: Supplementary file 14 — Additional file 14. CRISPResso NHEJ pie charts. [file 12896_2019_565_MOESM14_ESM.zip › CRISPResso_EPSPS-7DS-gRNA1-rep1.pdf]

Unmodified  
(14983 reads)

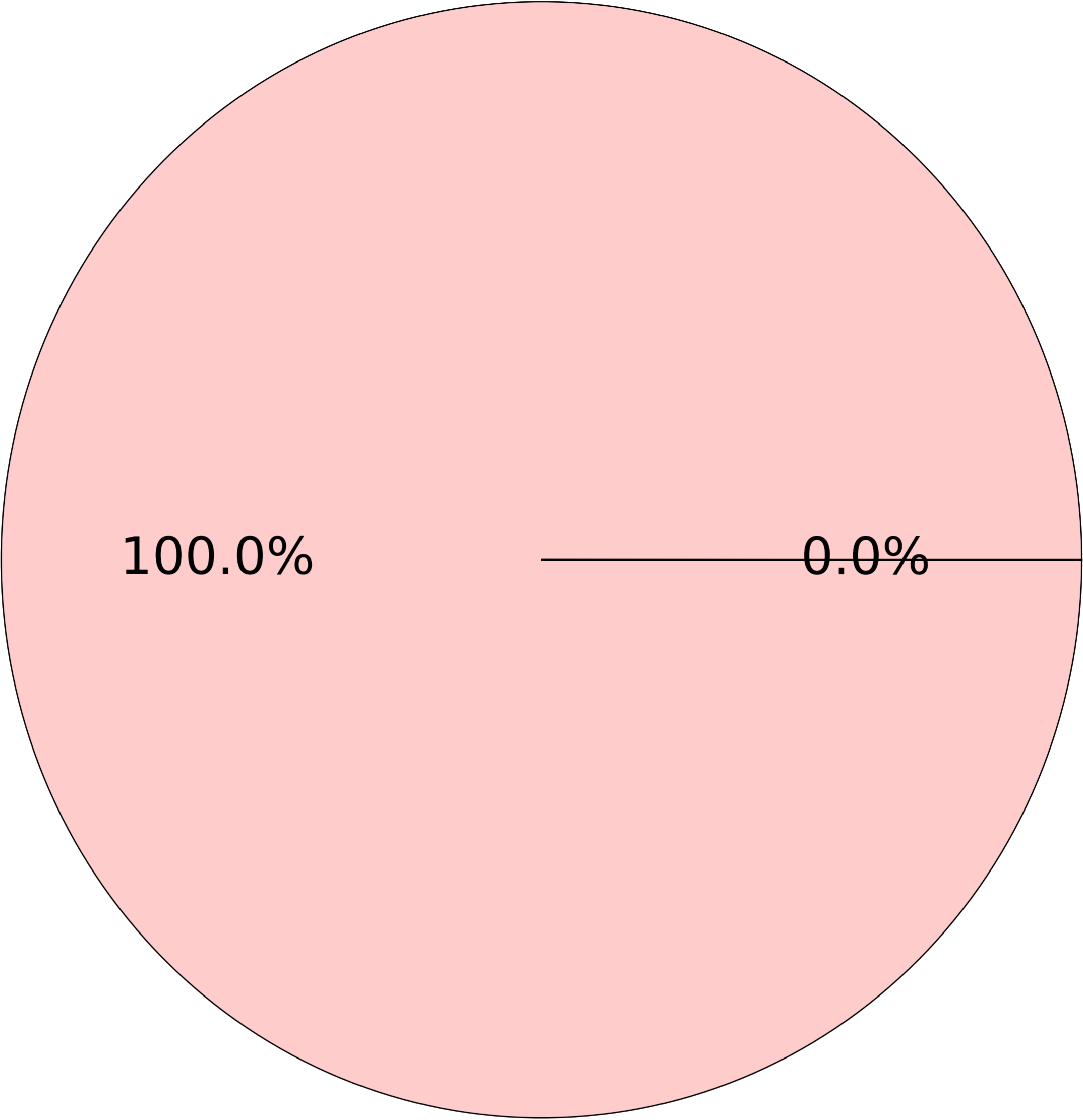

NHEJ  
(1 reads)

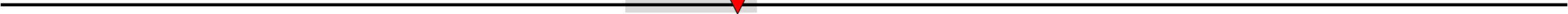

—

Amplicon sequence

—

sgRNA

▼

Predicted Cas9 cleavage site/s

Supplement: Supplementary file 14 — Additional file 14. CRISPResso NHEJ pie charts. [file 12896_2019_565_MOESM14_ESM.zip › CRISPResso_EPSPS-7DS-gRNA1-rep1-negative.pdf]

Unmodified  
(22961 reads)

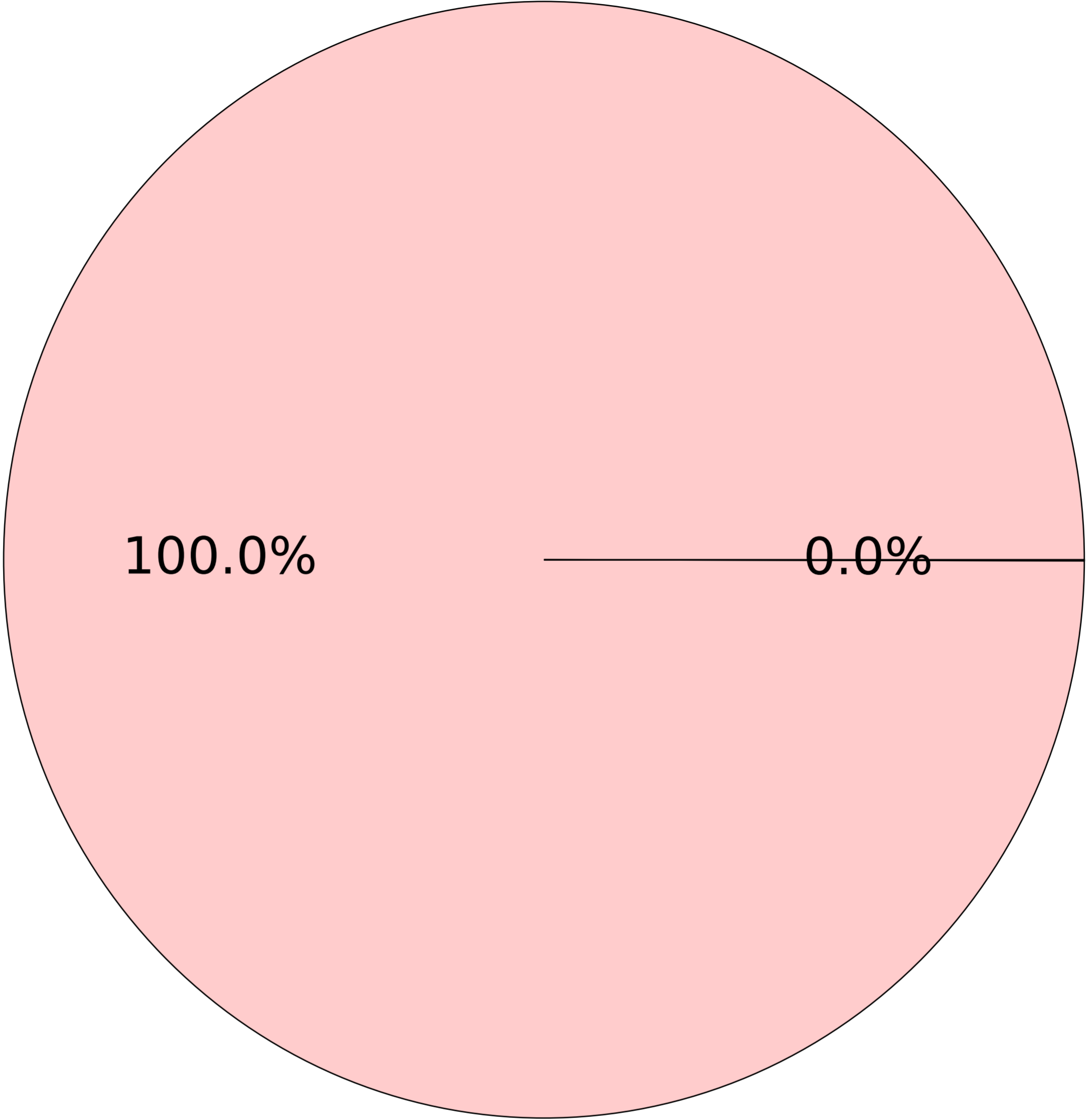

NHEJ  
(7 reads)

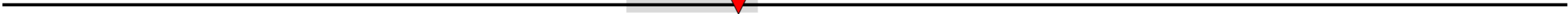

—

Amplicon sequence

—

sgRNA

▼

Predicted Cas9 cleavage site/s

Supplement: Supplementary file 14 — Additional file 14. CRISPResso NHEJ pie charts. [file 12896_2019_565_MOESM14_ESM.zip › CRISPResso_EPSPS-7DS-gRNA1-rep2.pdf]

Unmodified  
(12966 reads)

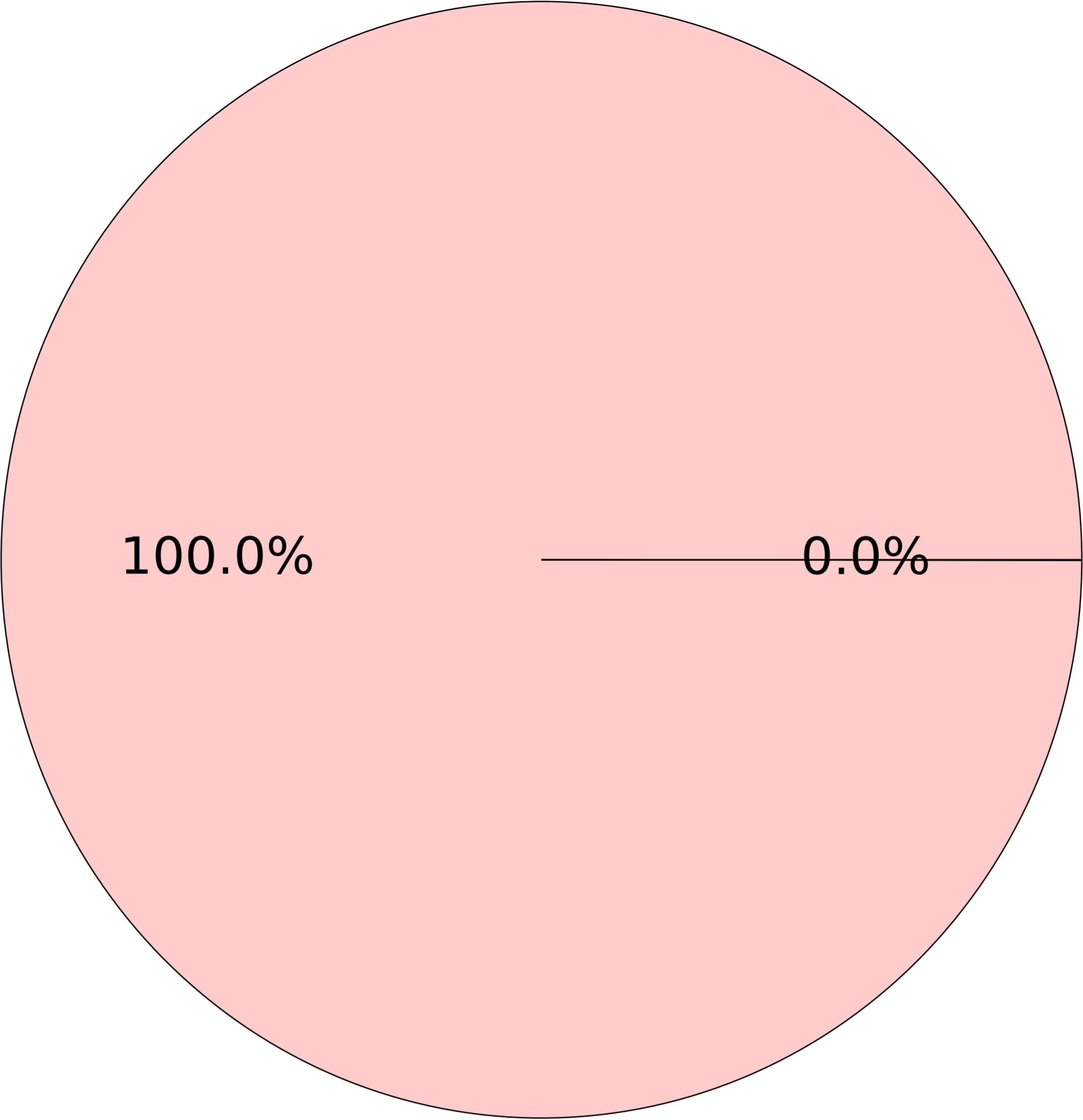

NHEJ  
(2 reads)

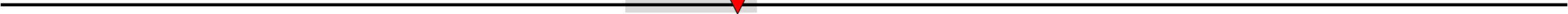

—

Amplicon sequence

—

sgRNA

▼

Predicted Cas9 cleavage site/s

Supplement: Supplementary file 14 — Additional file 14. CRISPResso NHEJ pie charts. [file 12896_2019_565_MOESM14_ESM.zip › CRISPResso_EPSPS-7DS-gRNA1-rep2-negative.pdf]

Unmodified  
(16434 reads)

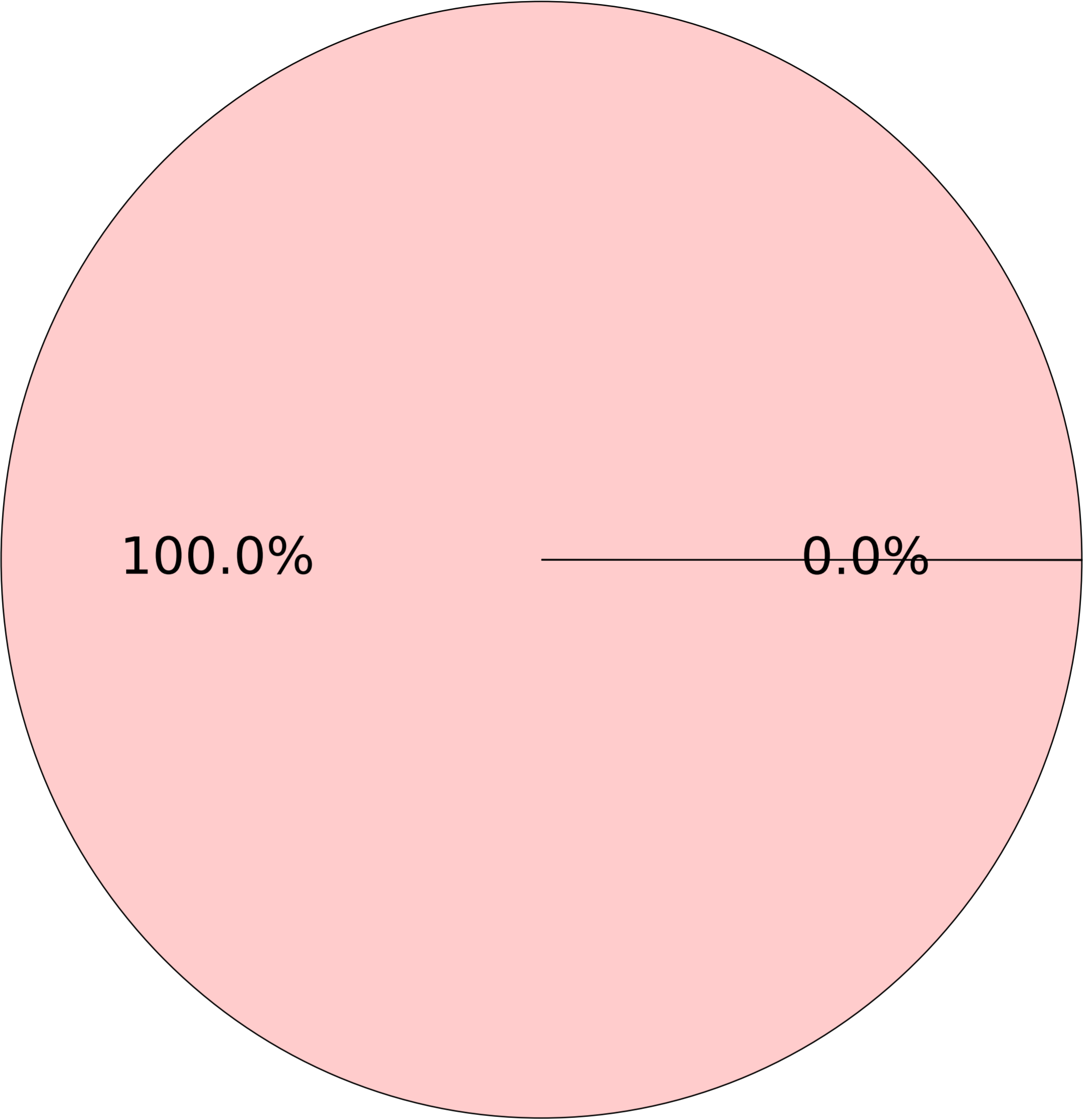

NHEJ  
(2 reads)

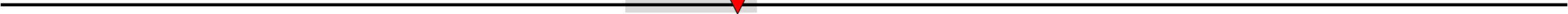

—

Amplicon sequence

—

sgRNA

▼

Predicted Cas9 cleavage site/s

Supplement: Supplementary file 14 — Additional file 14. CRISPResso NHEJ pie charts. [file 12896_2019_565_MOESM14_ESM.zip › CRISPResso_EPSPS-7DS-gRNA1-rep3.pdf]

Unmodified  
(18922 reads)

100.0%

0.0%

NHEJ  
(1 reads)

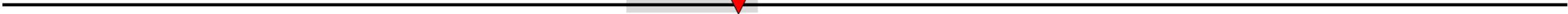

—

Amplicon sequence

—

sgRNA

▼

Predicted Cas9 cleavage site/s

Supplement: Supplementary file 14 — Additional file 14. CRISPResso NHEJ pie charts. [file 12896_2019_565_MOESM14_ESM.zip › CRISPResso_EPSPS-7DS-gRNA1-rep3-negative.pdf]

Unmodified  
(19420 reads)

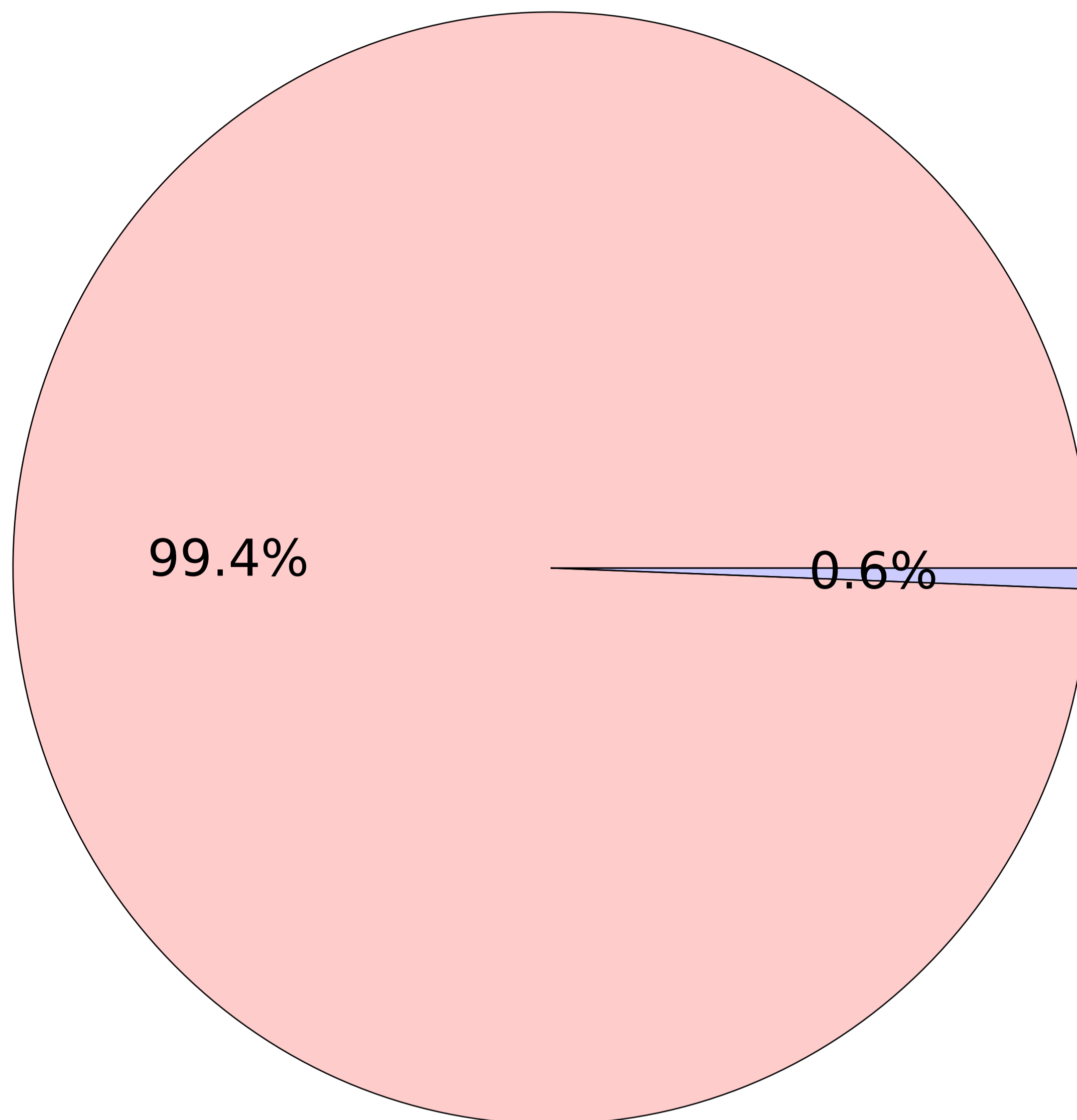

NHEJ  
(118 reads)

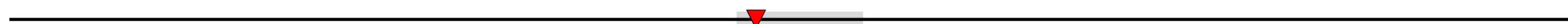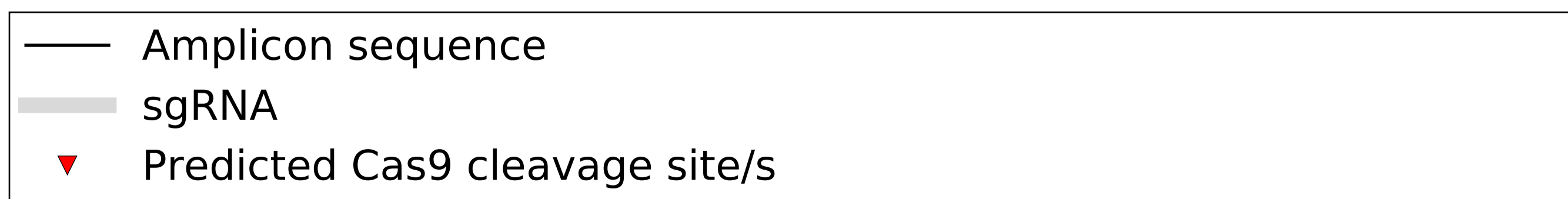

Supplement: Supplementary file 14 — Additional file 14. CRISPResso NHEJ pie charts. [file 12896_2019_565_MOESM14_ESM.zip › CRISPResso_EPSPS-7DS-gRNA2-rep1.pdf]

Unmodified  
(14983 reads)

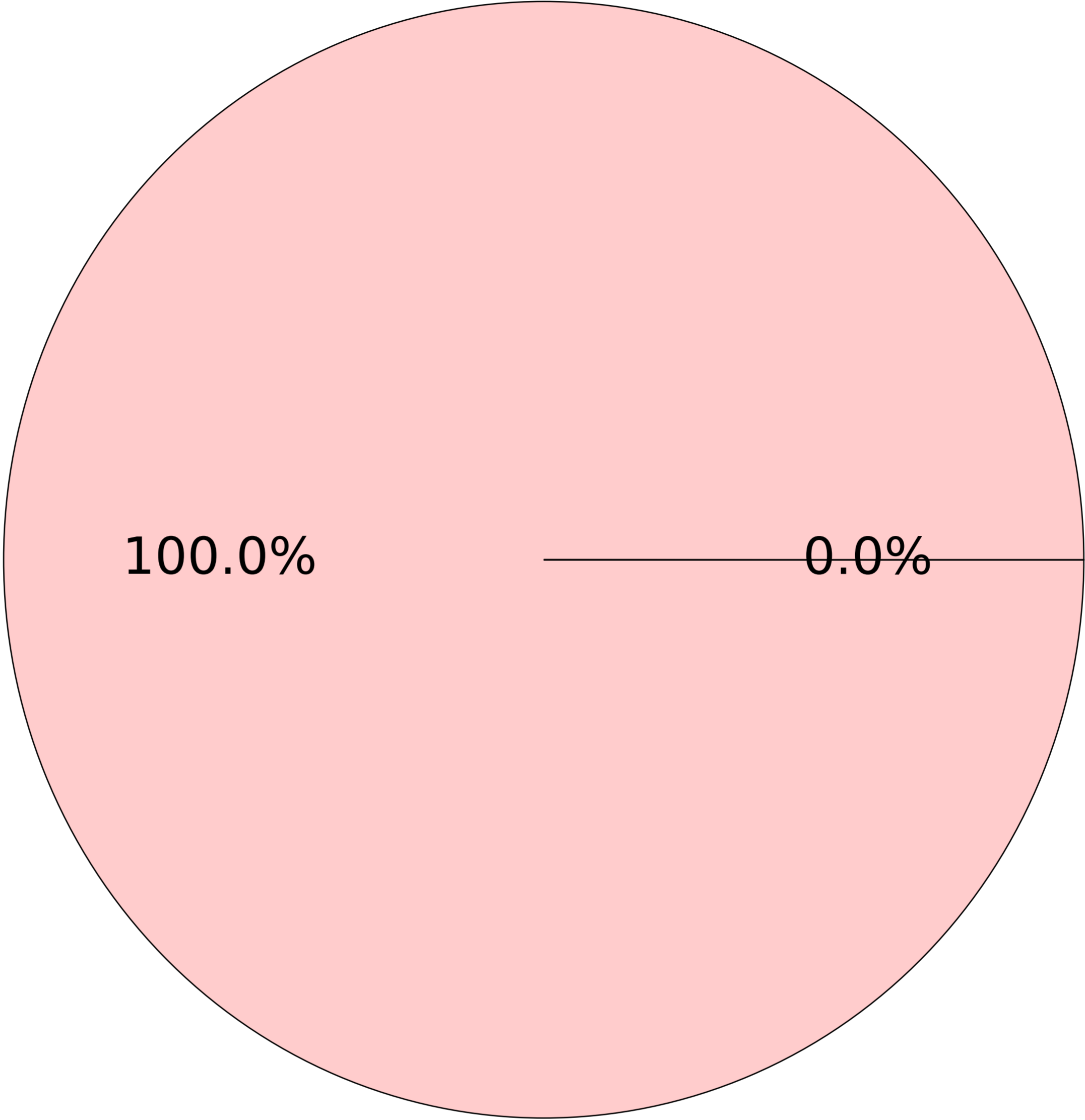

NHEJ  
(1 reads)

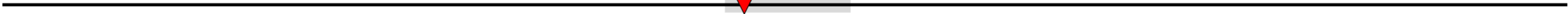

—

Amplicon sequence

—

sgRNA

▼

Predicted Cas9 cleavage site/s

Supplement: Supplementary file 14 — Additional file 14. CRISPResso NHEJ pie charts. [file 12896_2019_565_MOESM14_ESM.zip › CRISPResso_EPSPS-7DS-gRNA2-rep1-negative.pdf]

Unmodified  
(9974 reads)

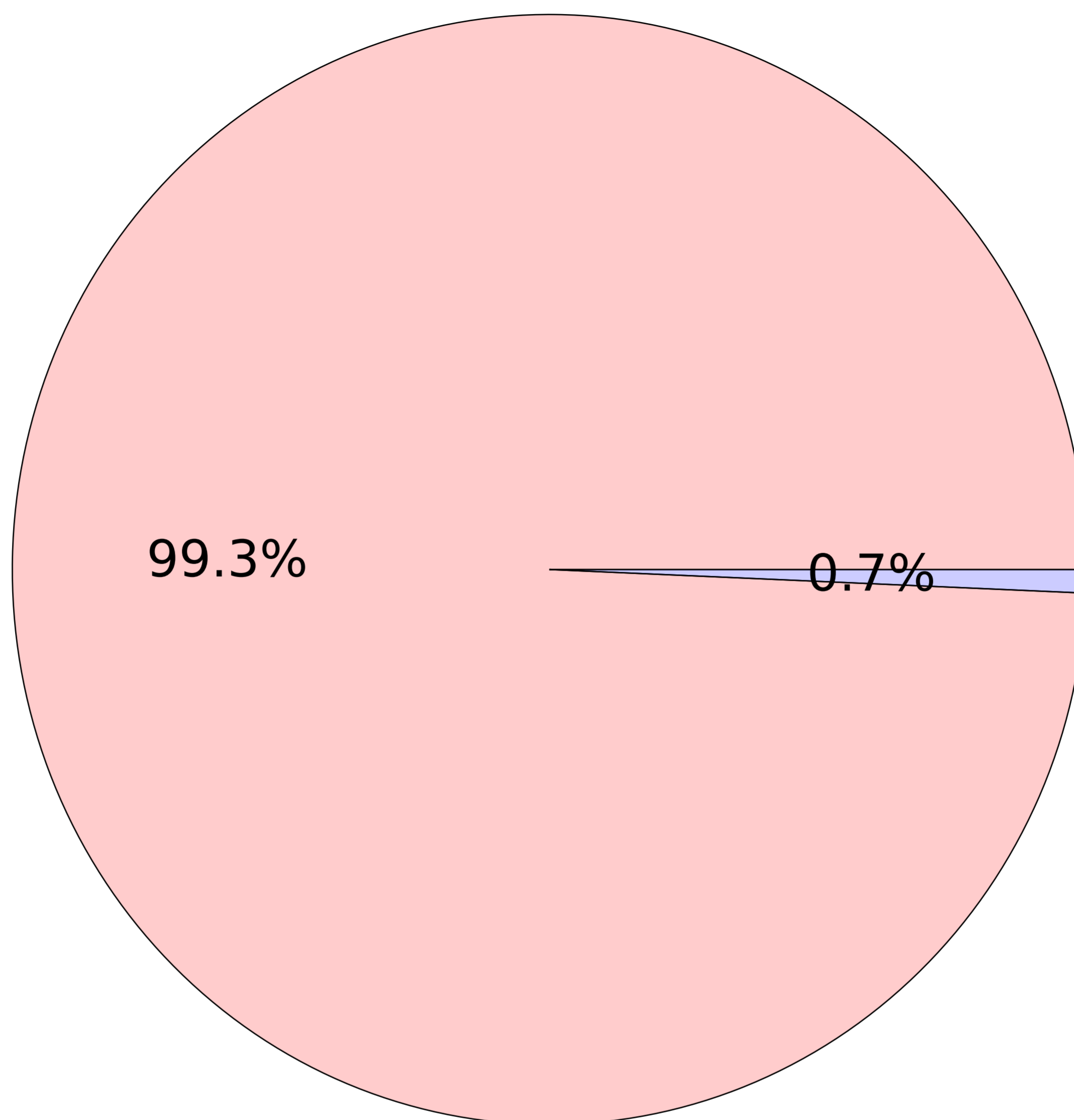

NHEJ  
(68 reads)

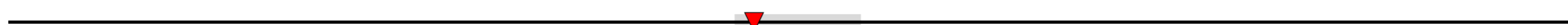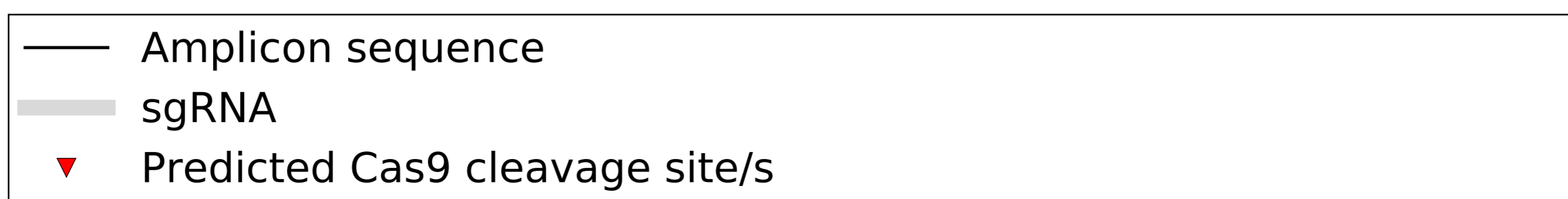

Supplement: Supplementary file 14 — Additional file 14. CRISPResso NHEJ pie charts. [file 12896_2019_565_MOESM14_ESM.zip › CRISPResso_EPSPS-7DS-gRNA2-rep2.pdf]

Unmodified  
(12966 reads)

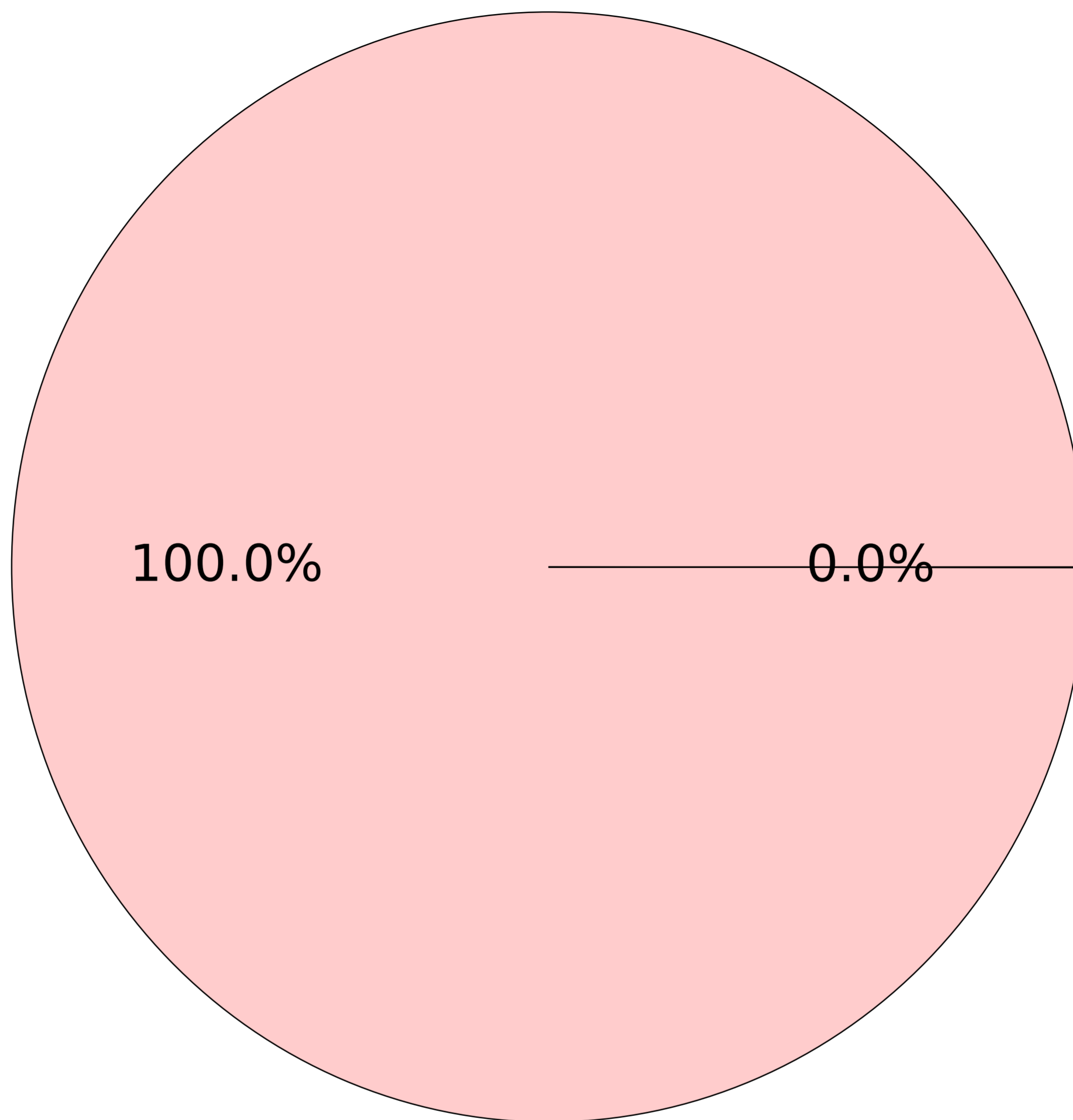

NHEJ  
(2 reads)

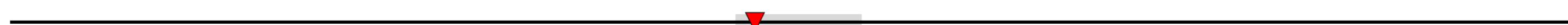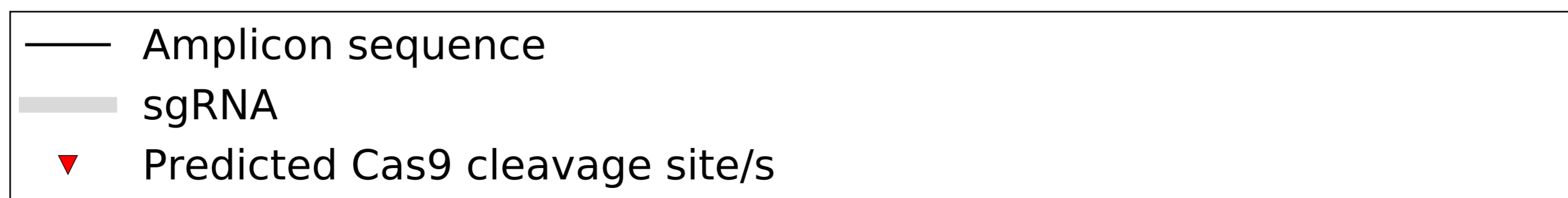

Supplement: Supplementary file 14 — Additional file 14. CRISPResso NHEJ pie charts. [file 12896_2019_565_MOESM14_ESM.zip › CRISPResso_EPSPS-7DS-gRNA2-rep2-negative.pdf]

Unmodified  
(13775 reads)

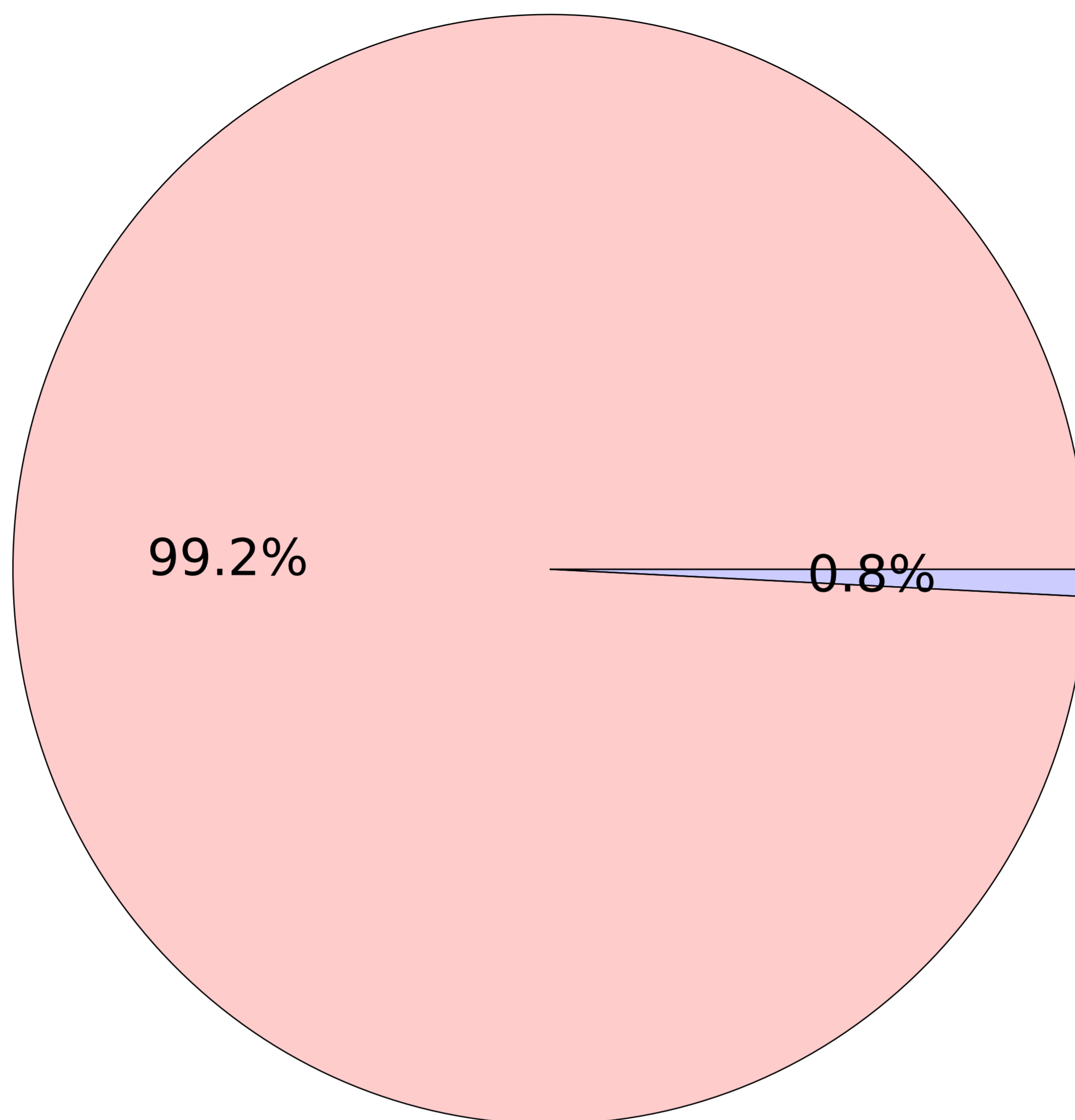

NHEJ  
(109 reads)

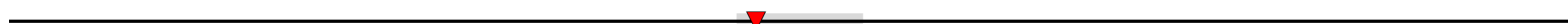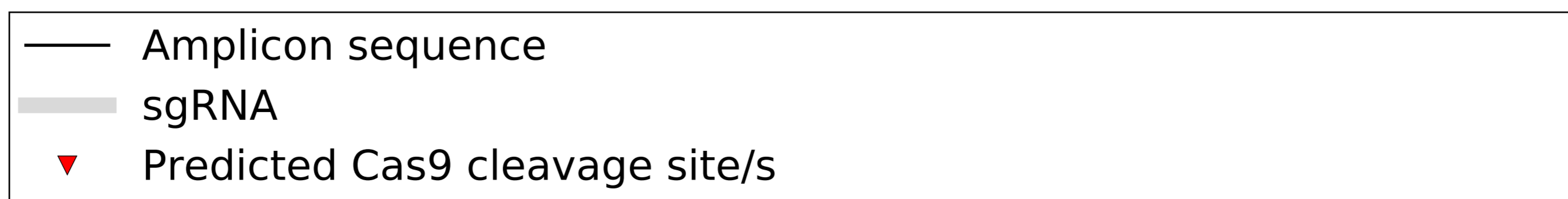

Supplement: Supplementary file 14 — Additional file 14. CRISPResso NHEJ pie charts. [file 12896_2019_565_MOESM14_ESM.zip › CRISPResso_EPSPS-7DS-gRNA2-rep3.pdf]

Unmodified  
(18922 reads)

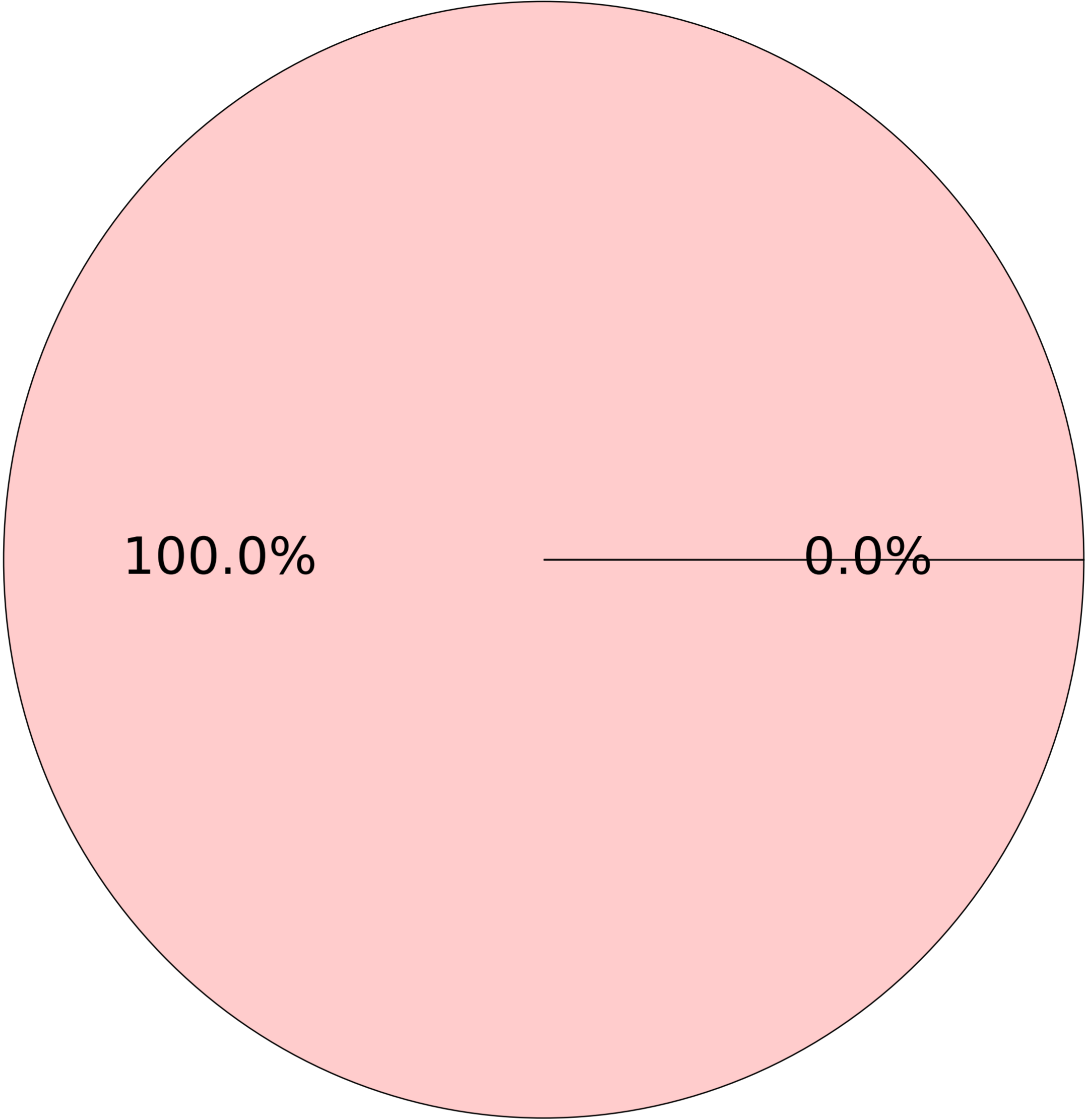

NHEJ  
(1 reads)

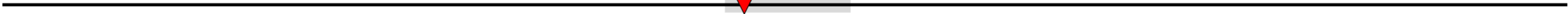

—

Amplicon sequence

—

sgRNA

▼

Predicted Cas9 cleavage site/s

Supplement: Supplementary file 14 — Additional file 14. CRISPResso NHEJ pie charts. [file 12896_2019_565_MOESM14_ESM.zip › CRISPResso_EPSPS-7DS-gRNA2-rep3-negative.pdf]
