# Supplementary material for: gRNA validation for wheat genome editing with the CRISPR-Cas9 system
Source: BMC Biotechnol. 2019 Oct 30;19:71. doi: 10.1186/s12896-019-0565-z (PMC6829922; doi:10.1186/s12896-019-0565-z)
Supplement: Supplementary file 12 — Additional file 12 TIDE indel spectra/frequencies for gRNAs 1–7 targeting EPSPS in wheat protoplasts. Shown are results for forward and reverse Sanger sequence reads of homoeoallele-specific amplicons derived from chromosomes 7AS, 4AL, and 7DS. Three replicates were performed. [file 12896_2019_565_MOESM12_ESM.pdf]

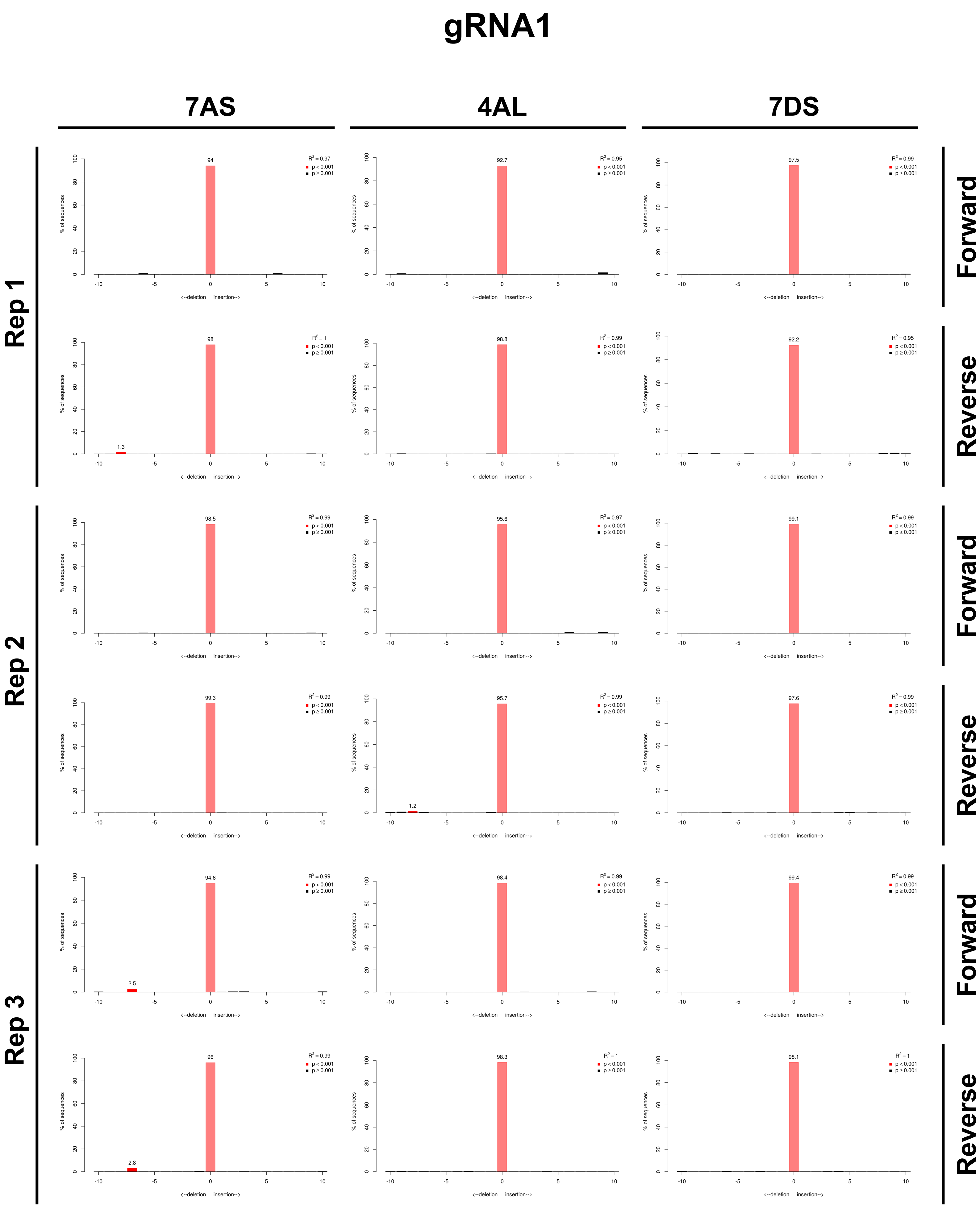
gRNA1
7AS
4AL
7DS
Rep 1
Rep 2
Rep 3
Forward
Reverse
Forward
Reverse
Forward
Reverse
% of sequences
<--deletion insertion-->
p < 0.001
p ≥ 0.001
94
R2 = 0.97
92.7
R2 = 0.95
97.5
R2 = 0.99
98
1.3
R2 = 1
98.8
R2 = 0.99
92.2
R2 = 0.95
98.5
R2 = 0.99
95.6
R2 = 0.97
99.1
R2 = 0.99
99.3
R2 = 0.99
95.7
1.2
R2 = 0.99
97.6
R2 = 0.99
94.6
2.5
R2 = 0.99
98.4
R2 = 0.99
99.4
R2 = 0.99
96
2.8
R2 = 0.99
98.3
R2 = 1
98.1
R2 = 1

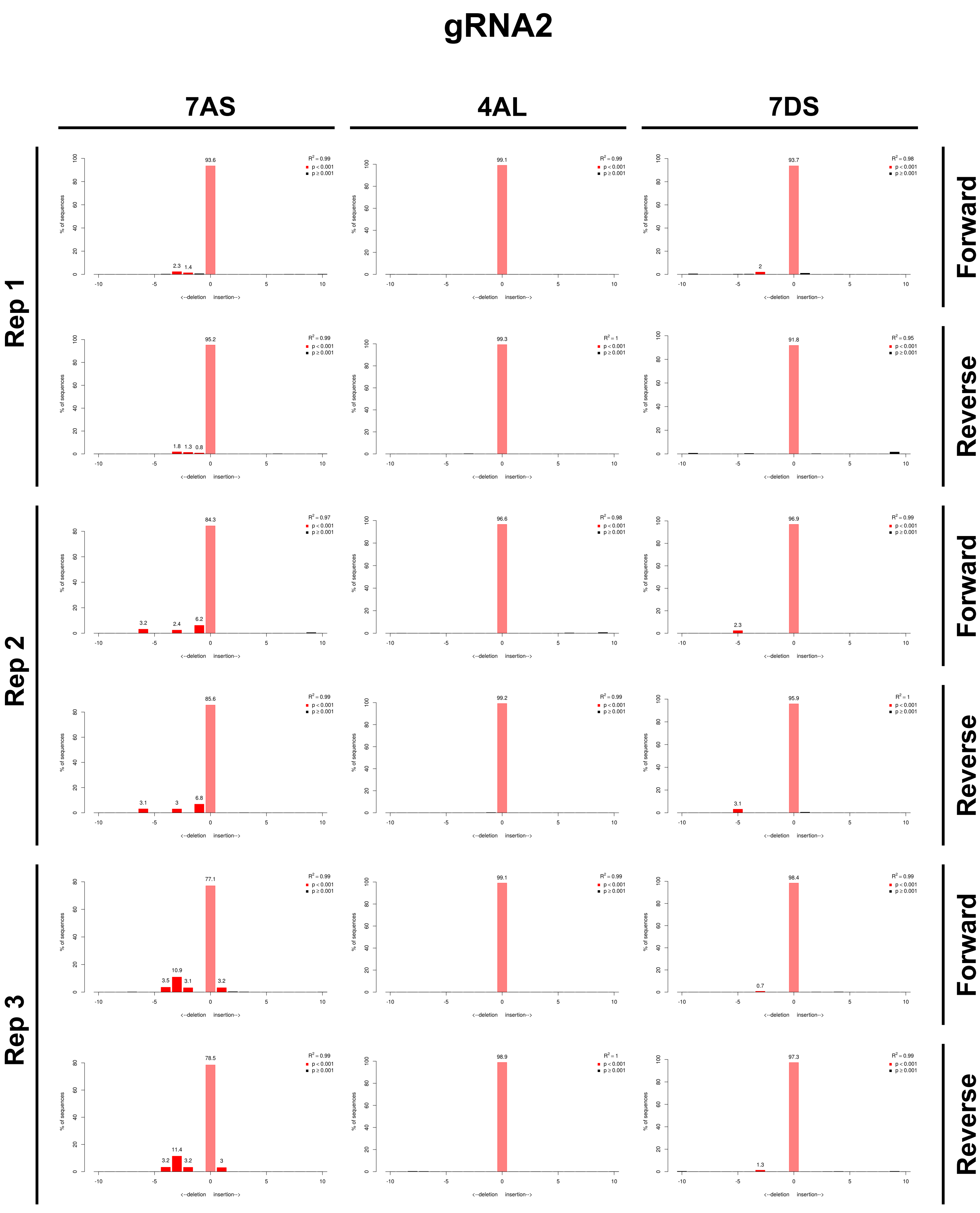

gRNA2
7AS
4AL
7DS
Rep 1
Rep 2
Rep 3
Forward
Reverse
Forward
Reverse
Forward
Reverse
% of sequences
<--deletion insertion-->
R^2 = 0.99
p < 0.001
p ≥ 0.001
93.6
2.3
1.4
99.1
93.7
2
95.2
1.8
1.3
0.8
99.3
91.8
84.3
3.2
2.4
6.2
96.6
96.9
2.3
85.6
3.1
3
6.8
99.2
95.9
3.1
77.1
3.5
10.9
3.1
3.2
99.1
98.4
0.7
78.5
3.2
11.4
3.2
3
98.9
97.3
1.3

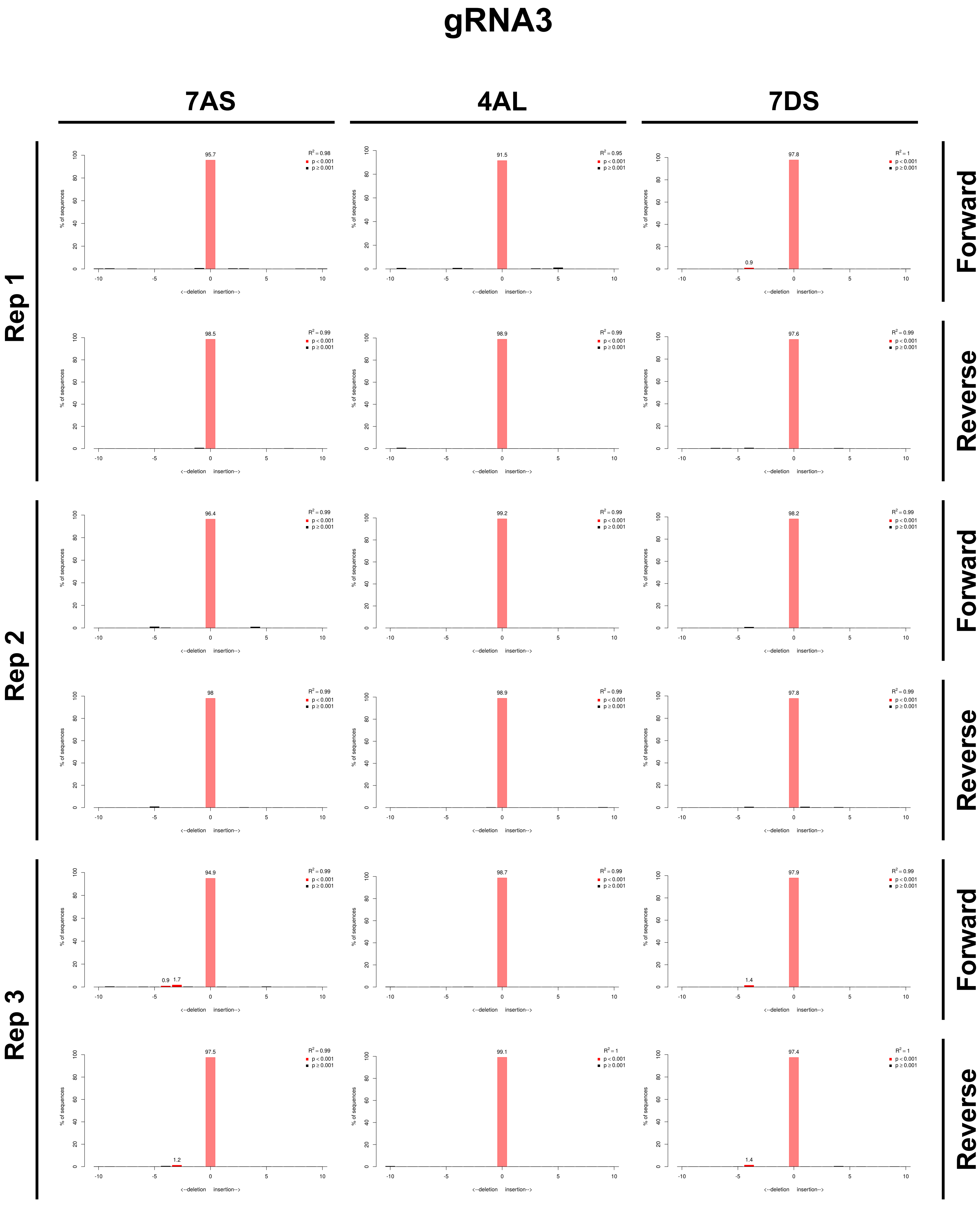
gRNA3
7AS
4AL
7DS
Rep 1
Rep 2
Rep 3
Forward
Reverse
Forward
Reverse
Forward
Reverse
% of sequences
<--deletion insertion-->
R^2 = 0.98
R^2 = 0.95
R^2 = 1
R^2 = 0.99
p < 0.001
p ≥ 0.001
95.7
91.5
97.8
0.9
98.5
98.9
97.6
96.4
99.2
98.2
98
98.9
97.8
94.9
0.9
1.7
98.7
97.9
1.4
97.5
1.2
99.1
97.4
1.4

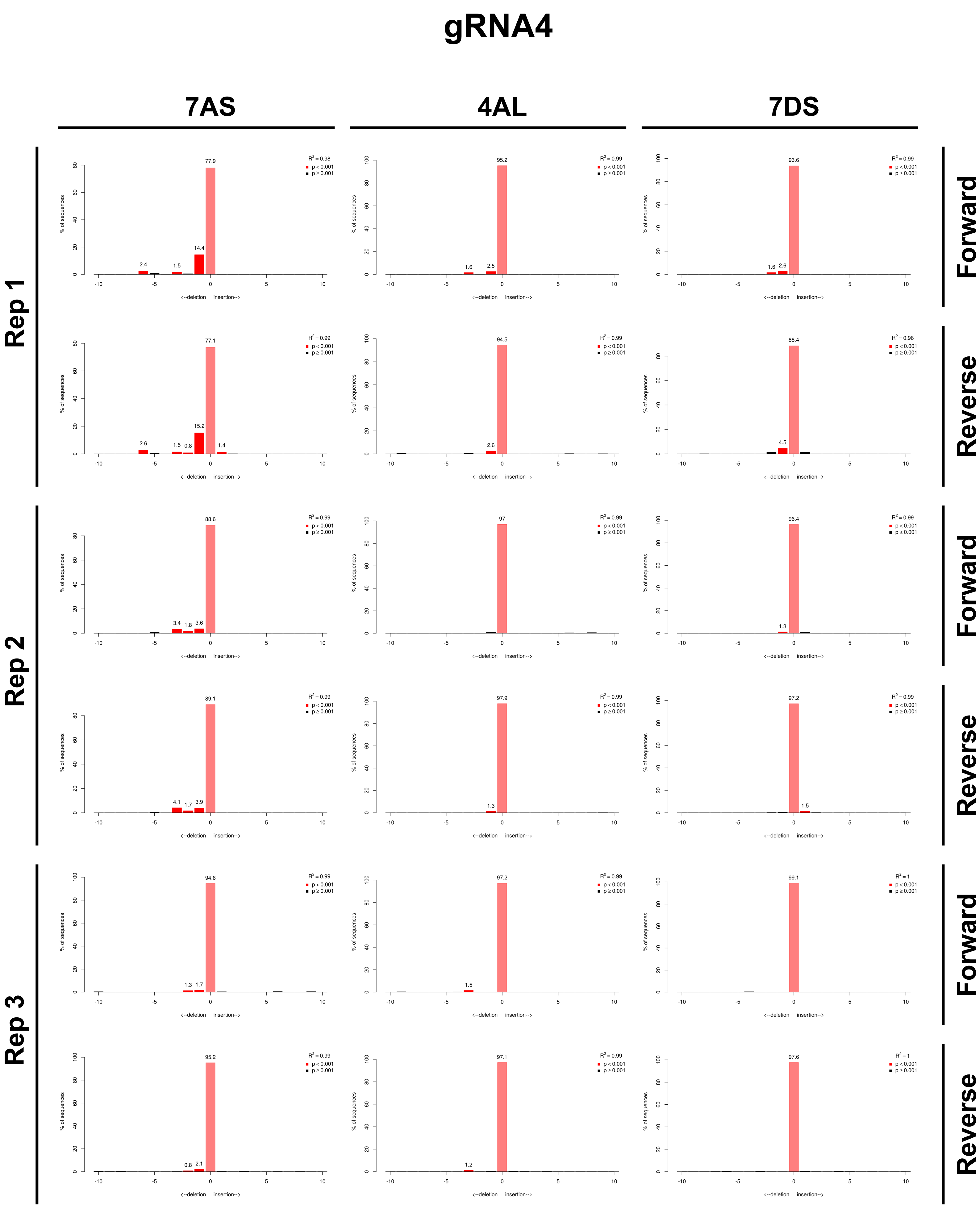

gRNA4
7AS
4AL
7DS
Rep 1
Rep 2
Rep 3
Forward
Reverse
Forward
Reverse
Forward
Reverse
% of sequences
<--deletion insertion-->
p < 0.001
p ≥ 0.001
R² = 0.98
2.4 1.5 14.4 77.9
R² = 0.99
1.6 2.5 95.2
R² = 0.99
1.6 2.6 93.6
R² = 0.99
2.6 1.5 0.8 15.2 77.1 1.4
R² = 0.99
2.6 94.5
R² = 0.96
4.5 88.4
R² = 0.99
3.4 1.8 3.6 88.6
R² = 0.99
97
R² = 0.99
1.3 96.4
R² = 0.99
4.1 1.7 3.9 89.1
R² = 0.99
1.3 97.9
R² = 0.99
97.2 1.5
R² = 0.99
1.3 1.7 94.6
R² = 0.99
1.5 97.2
R² = 1
99.1
R² = 0.99
0.8 2.1 95.2
R² = 0.99
1.2 97.1
R² = 1
97.6

gRNA5

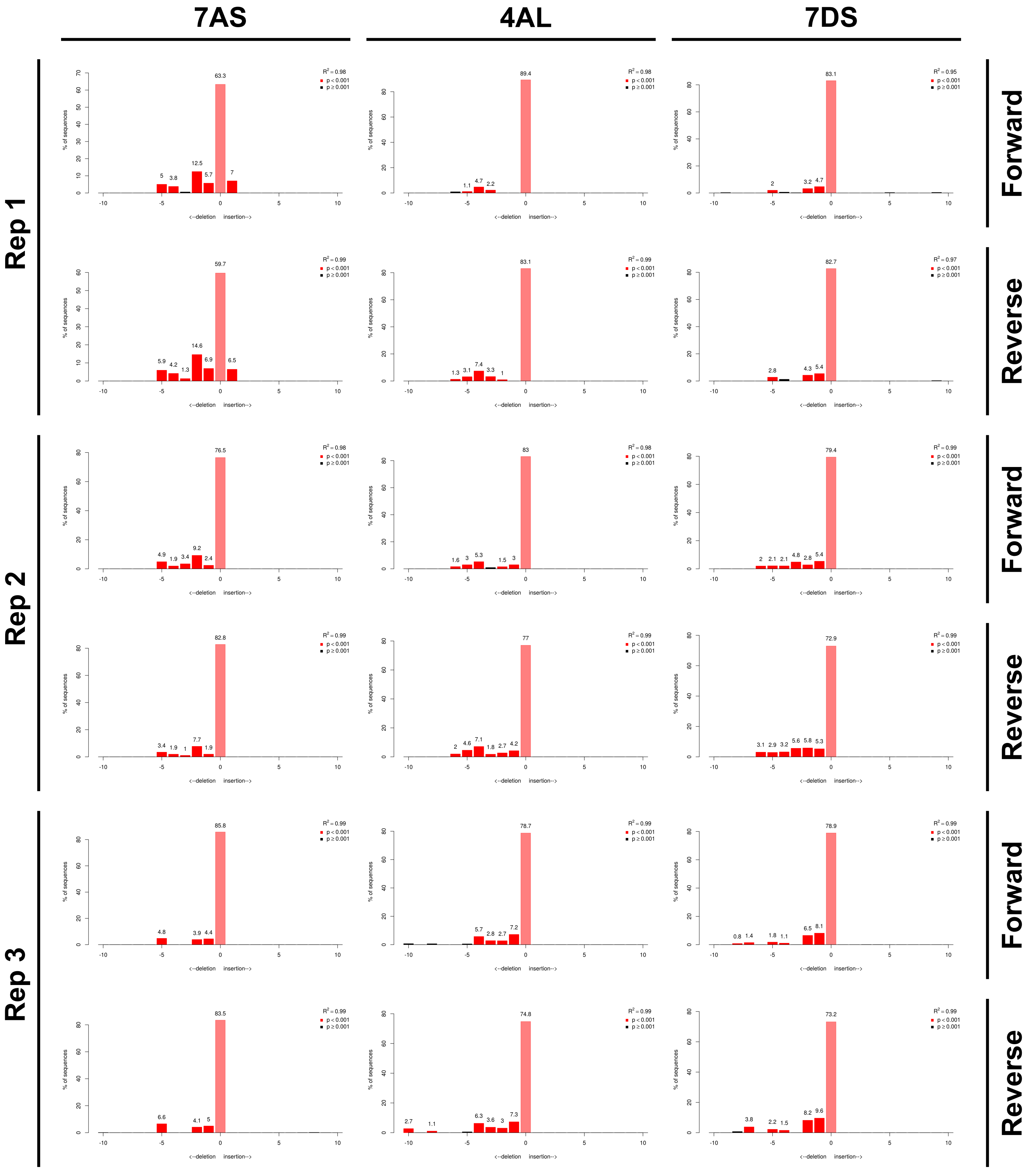

7AS
4AL
7DS
Rep 1
Rep 2
Rep 3
Forward
Reverse
% of sequences
<--deletion insertion-->
$R^2 = 0.98$
p < 0.001
p ≥ 0.001
Rep 1 Forward 7AS: $R^2 = 0.98$; 5, 3.8, 12.5, 5.7, 63.3, 7
Rep 1 Forward 4AL: $R^2 = 0.98$; 1.1, 4.7, 2.2, 89.4
Rep 1 Forward 7DS: $R^2 = 0.95$; 2, 3.2, 4.7, 83.1
Rep 1 Reverse 7AS: $R^2 = 0.99$; 5.9, 4.2, 1.3, 14.6, 6.9, 59.7, 6.5
Rep 1 Reverse 4AL: $R^2 = 0.99$; 1.3, 3.1, 7.4, 3.3, 1, 83.1
Rep 1 Reverse 7DS: $R^2 = 0.97$; 2.8, 4.3, 5.4, 82.7
Rep 2 Forward 7AS: $R^2 = 0.98$; 4.9, 1.9, 3.4, 9.2, 2.4, 76.5
Rep 2 Forward 4AL: $R^2 = 0.98$; 1.6, 3, 5.3, 1.5, 3, 83
Rep 2 Forward 7DS: $R^2 = 0.99$; 2, 2.1, 2.1, 4.8, 2.8, 5.4, 79.4
Rep 2 Reverse 7AS: $R^2 = 0.99$; 3.4, 1.9, 1, 7.7, 1.9, 82.8
Rep 2 Reverse 4AL: $R^2 = 0.99$; 2, 4.6, 7.1, 1.8, 2.7, 4.2, 77
Rep 2 Reverse 7DS: $R^2 = 0.99$; 3.1, 2.9, 3.2, 5.6, 5.8, 5.3, 72.9
Rep 3 Forward 7AS: $R^2 = 0.99$; 4.8, 3.9, 4.4, 85.8
Rep 3 Forward 4AL: $R^2 = 0.99$; 5.7, 2.8, 2.7, 7.2, 78.7
Rep 3 Forward 7DS: $R^2 = 0.99$; 0.8, 1.4, 1.8, 1.1, 6.5, 8.1, 78.9
Rep 3 Reverse 7AS: $R^2 = 0.99$; 6.6, 4.1, 5, 83.5
Rep 3 Reverse 4AL: $R^2 = 0.99$; 2.7, 1.1, 6.3, 3.6, 3, 7.3, 74.8
Rep 3 Reverse 7DS: $R^2 = 0.99$; 3.8, 2.2, 1.5, 8.2, 9.6, 73.2

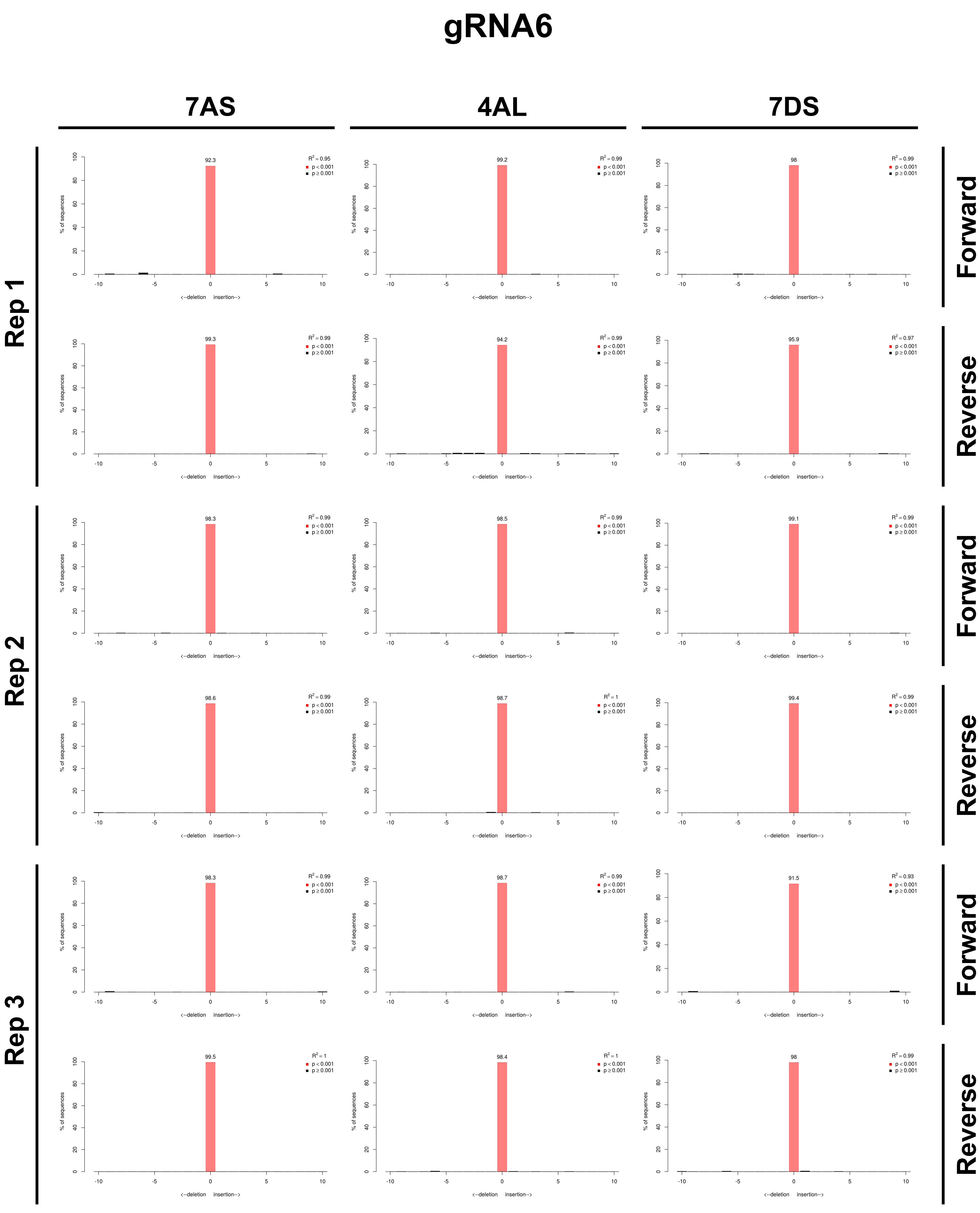

gRNA6
7AS
4AL
7DS
Rep 1
Rep 2
Rep 3
Forward
Reverse
Forward
Reverse
Forward
Reverse
% of sequences
<--deletion insertion-->
p < 0.001
p ≥ 0.001
92.3
99.2
98
99.3
94.2
95.9
98.3
98.5
99.1
98.6
98.7
99.4
98.3
98.7
91.5
99.5
98.4
98

gRNA7

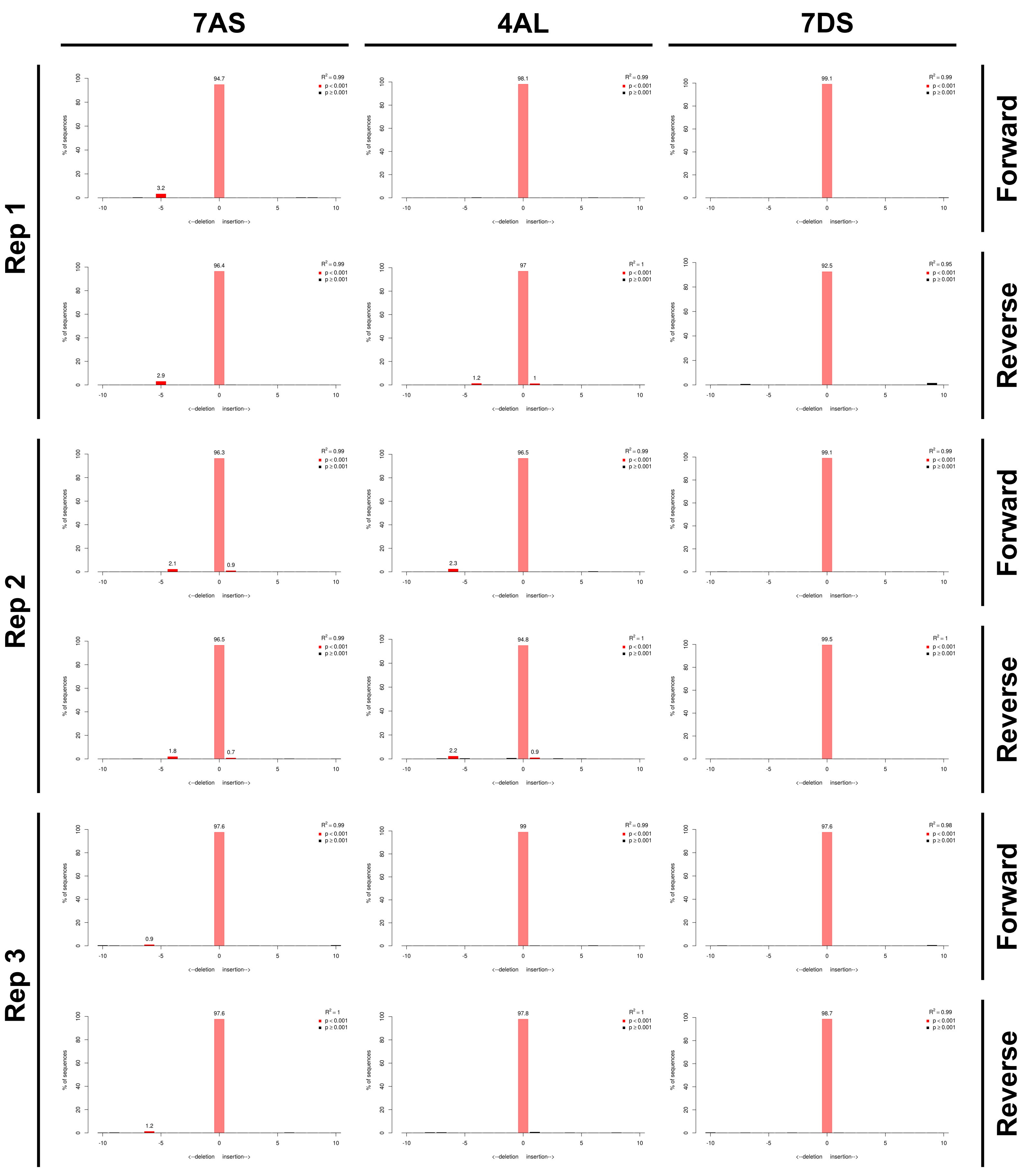
7AS
4AL
7DS
Rep 1
Rep 2
Rep 3
Forward
Reverse
% of sequences
<--deletion insertion-->
p < 0.001
p ≥ 0.001
R^2 = 0.99
94.7
3.2
98.1
99.1
R^2 = 1
R^2 = 0.95
96.4
2.9
97
1.2
1
92.5
96.3
2.1
0.9
96.5
2.3
96.5
1.8
0.7
94.8
2.2
0.9
99.5
97.6
0.9
99
R^2 = 0.98
97.6
1.2
97.8
98.7
