# Supplementary figures and images for: gRNA validation for wheat genome editing with the CRISPR-Cas9 system (part 2 of 2)
Source: BMC Biotechnol. 2019 Oct 30;19:71. doi: 10.1186/s12896-019-0565-z (PMC6829922; doi:10.1186/s12896-019-0565-z)

Unmodified  
(20034 reads)

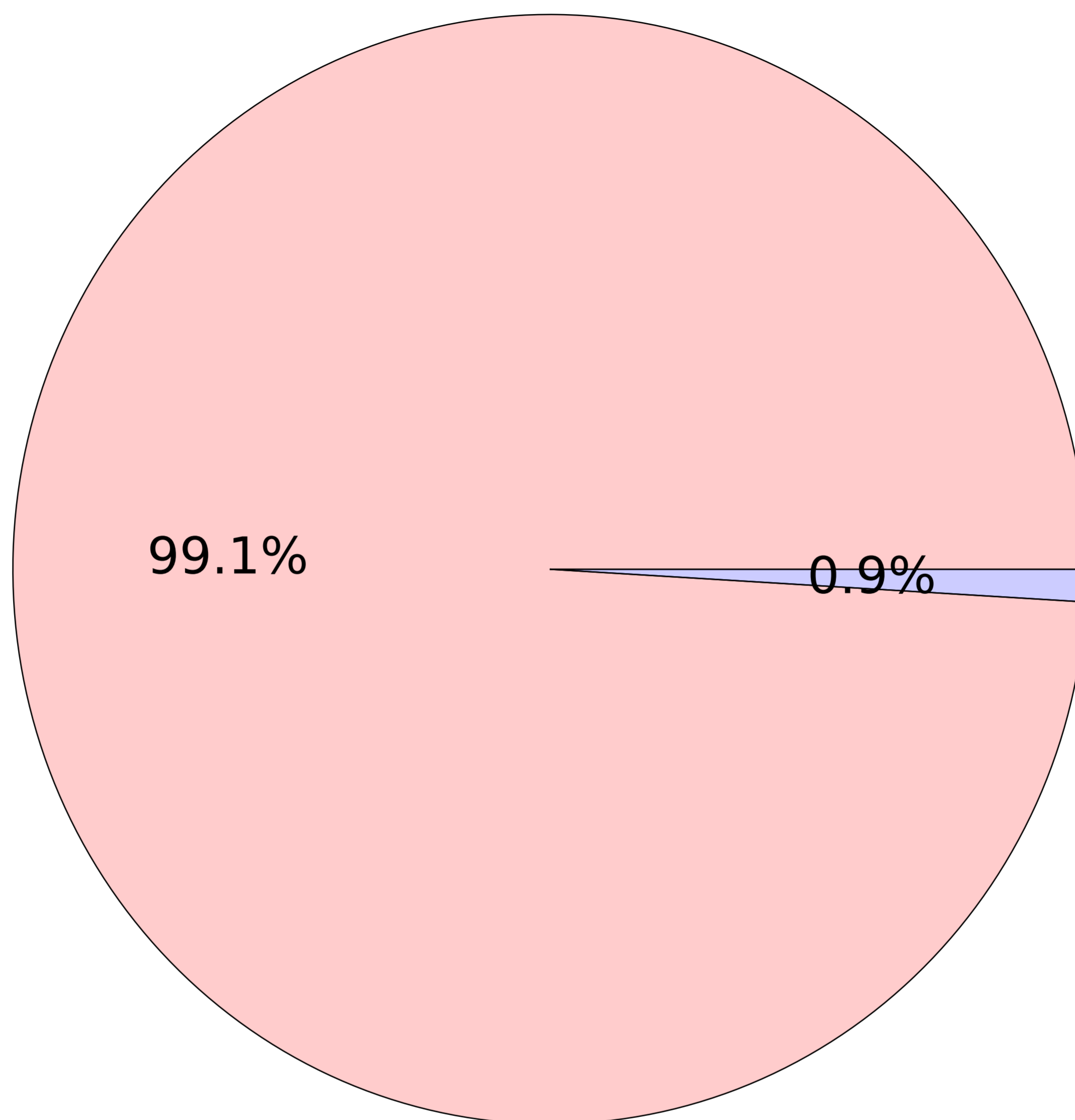

NHEJ  
(190 reads)

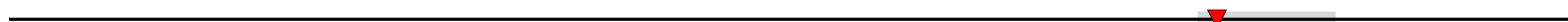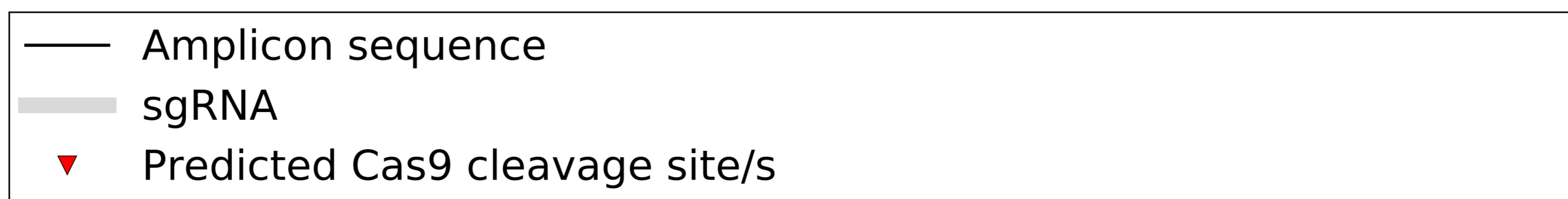

Supplement: Supplementary file 14 — Additional file 14. CRISPResso NHEJ pie charts. [file 12896_2019_565_MOESM14_ESM.zip › CRISPResso_EPSPS-7DS-gRNA3-rep1.pdf]

Unmodified  
(14981 reads)

100.0%

0.0%

NHEJ  
(3 reads)

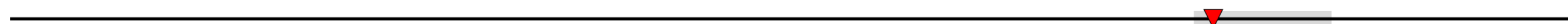

- Amplicon sequence
- sgRNA
- ▼ Predicted Cas9 cleavage site/s

Supplement: Supplementary file 14 — Additional file 14. CRISPResso NHEJ pie charts. [file 12896_2019_565_MOESM14_ESM.zip › CRISPResso_EPSPS-7DS-gRNA3-rep1-negative.pdf]

Unmodified  
(17838 reads)

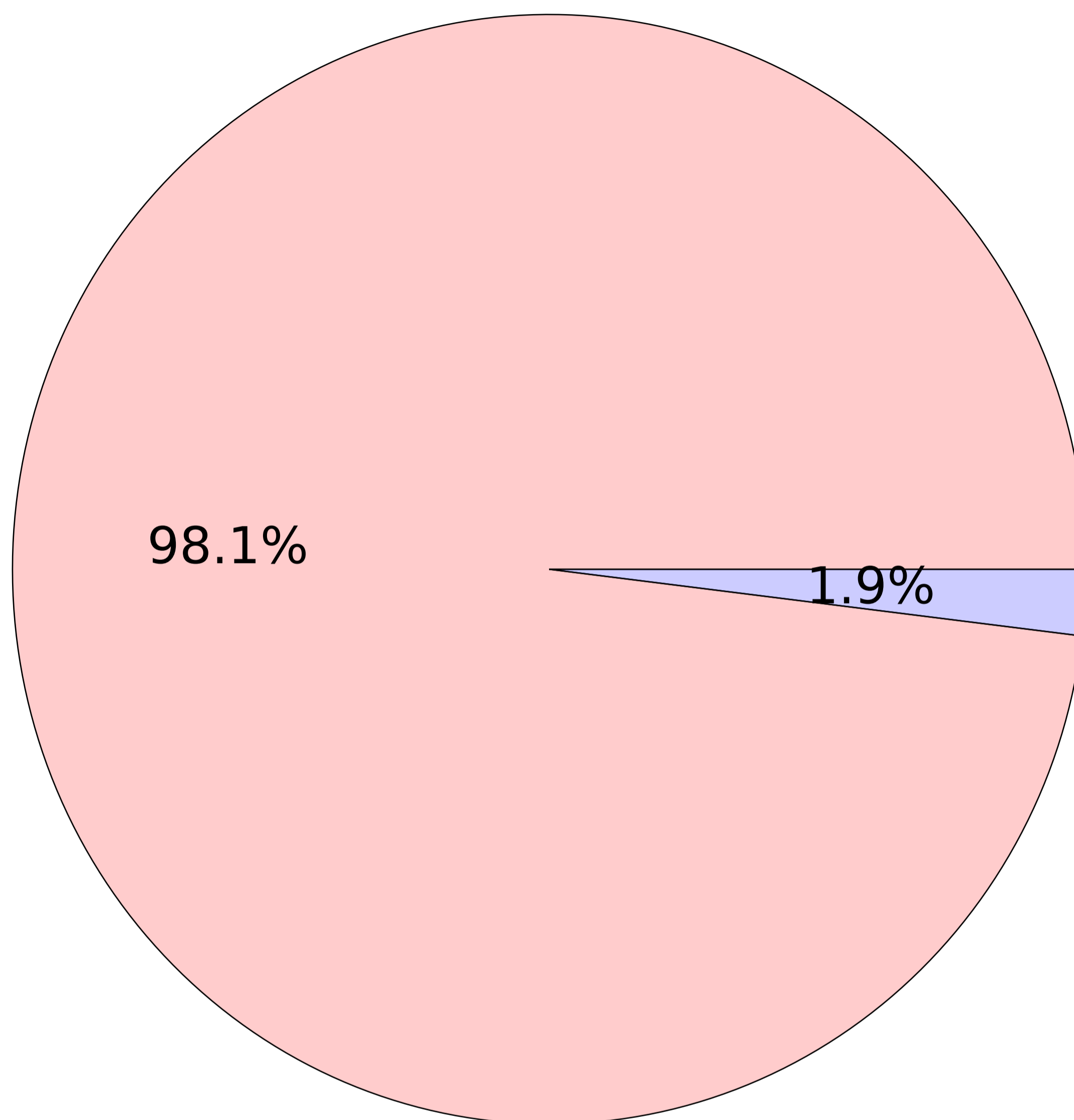

NHEJ  
(349 reads)

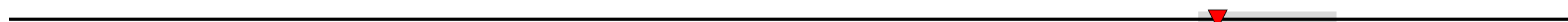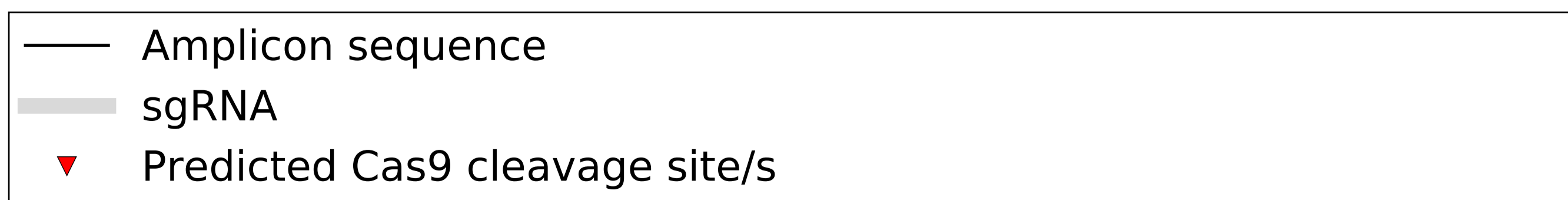

Supplement: Supplementary file 14 — Additional file 14. CRISPResso NHEJ pie charts. [file 12896_2019_565_MOESM14_ESM.zip › CRISPResso_EPSPS-7DS-gRNA3-rep2.pdf]

Unmodified  
(12967 reads)

100.0%

0.0%

NHEJ  
(1 reads)

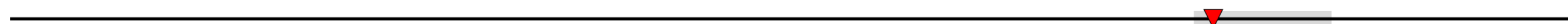

- Amplicon sequence
- sgRNA
- ▼ Predicted Cas9 cleavage site/s

Supplement: Supplementary file 14 — Additional file 14. CRISPResso NHEJ pie charts. [file 12896_2019_565_MOESM14_ESM.zip › CRISPResso_EPSPS-7DS-gRNA3-rep2-negative.pdf]

Unmodified  
(16566 reads)

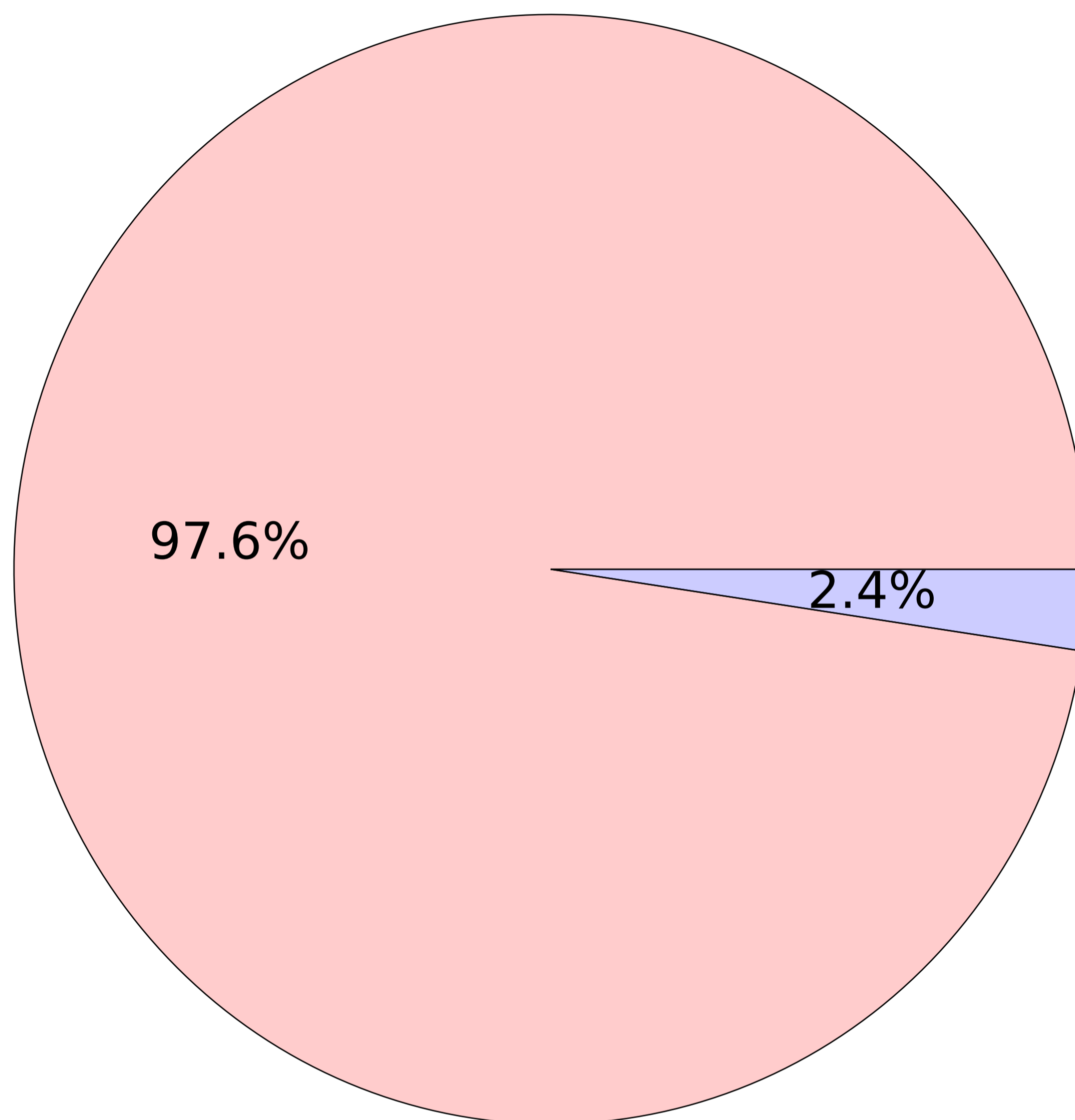

NHEJ  
(399 reads)

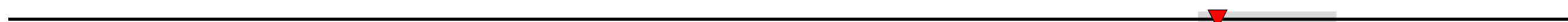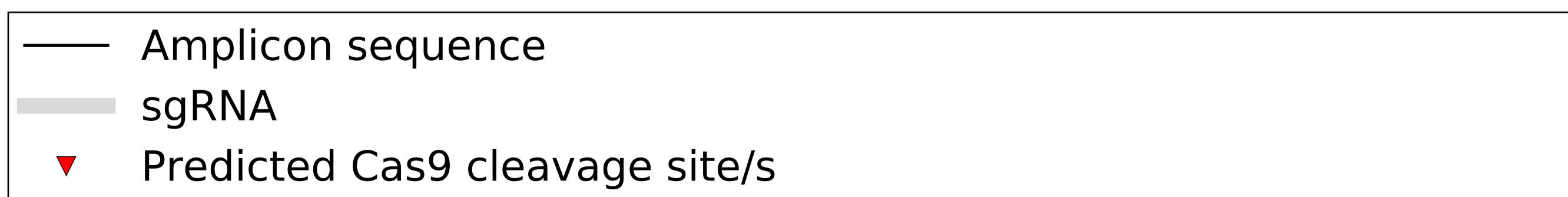

Supplement: Supplementary file 14 — Additional file 14. CRISPResso NHEJ pie charts. [file 12896_2019_565_MOESM14_ESM.zip › CRISPResso_EPSPS-7DS-gRNA3-rep3.pdf]

Unmodified  
(18921 reads)

100.0%

0.0%

NHEJ  
(2 reads)

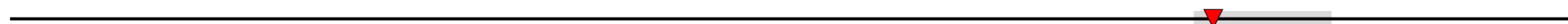

- Amplicon sequence
- sgRNA
- ▼ Predicted Cas9 cleavage site/s

Supplement: Supplementary file 14 — Additional file 14. CRISPResso NHEJ pie charts. [file 12896_2019_565_MOESM14_ESM.zip › CRISPResso_EPSPS-7DS-gRNA3-rep3-negative.pdf]

Unmodified  
(17512 reads)

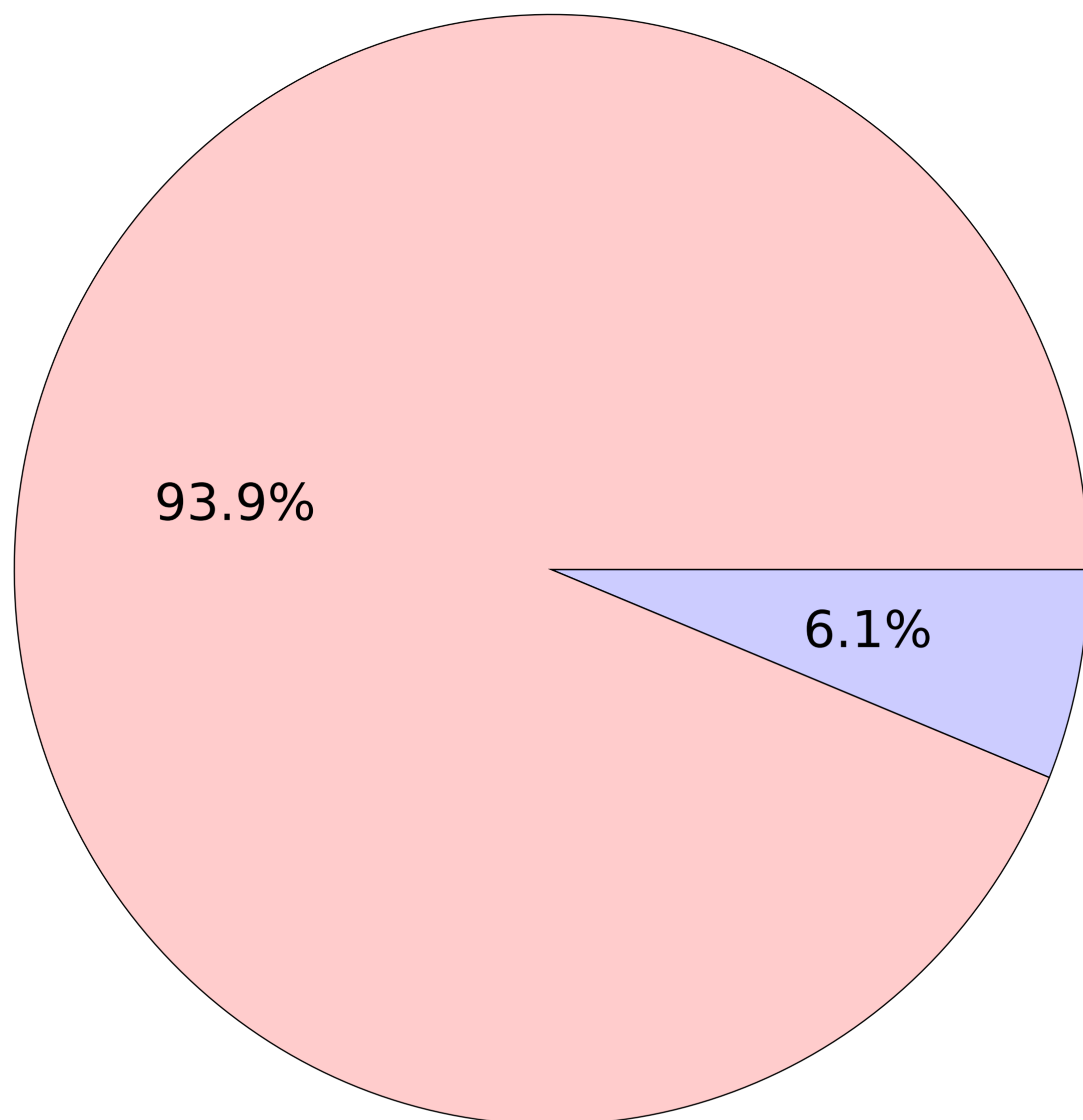

NHEJ  
(1140 reads)

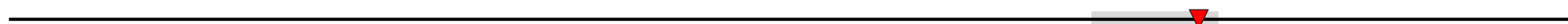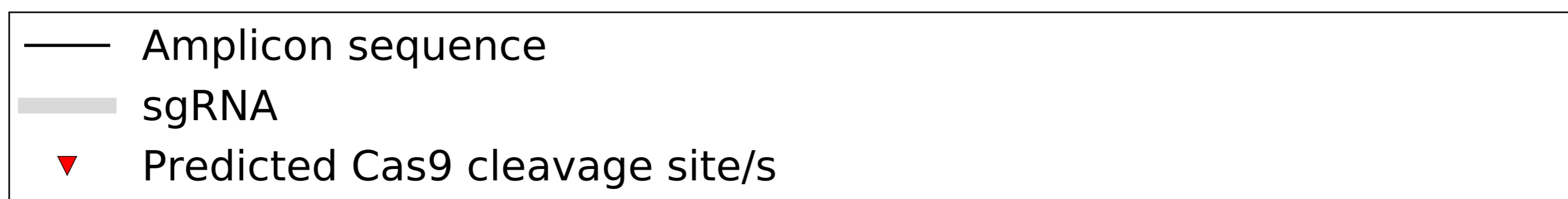

Supplement: Supplementary file 14 — Additional file 14. CRISPResso NHEJ pie charts. [file 12896_2019_565_MOESM14_ESM.zip › CRISPResso_EPSPS-7DS-gRNA4-rep1.pdf]

Unmodified  
(14981 reads)

100.0%

0.0%

NHEJ  
(3 reads)

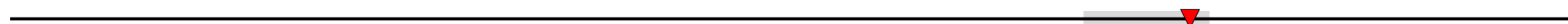

- Amplicon sequence
- sgRNA
- ▼ Predicted Cas9 cleavage site/s

Supplement: Supplementary file 14 — Additional file 14. CRISPResso NHEJ pie charts. [file 12896_2019_565_MOESM14_ESM.zip › CRISPResso_EPSPS-7DS-gRNA4-rep1-negative.pdf]

Unmodified  
(21599 reads)

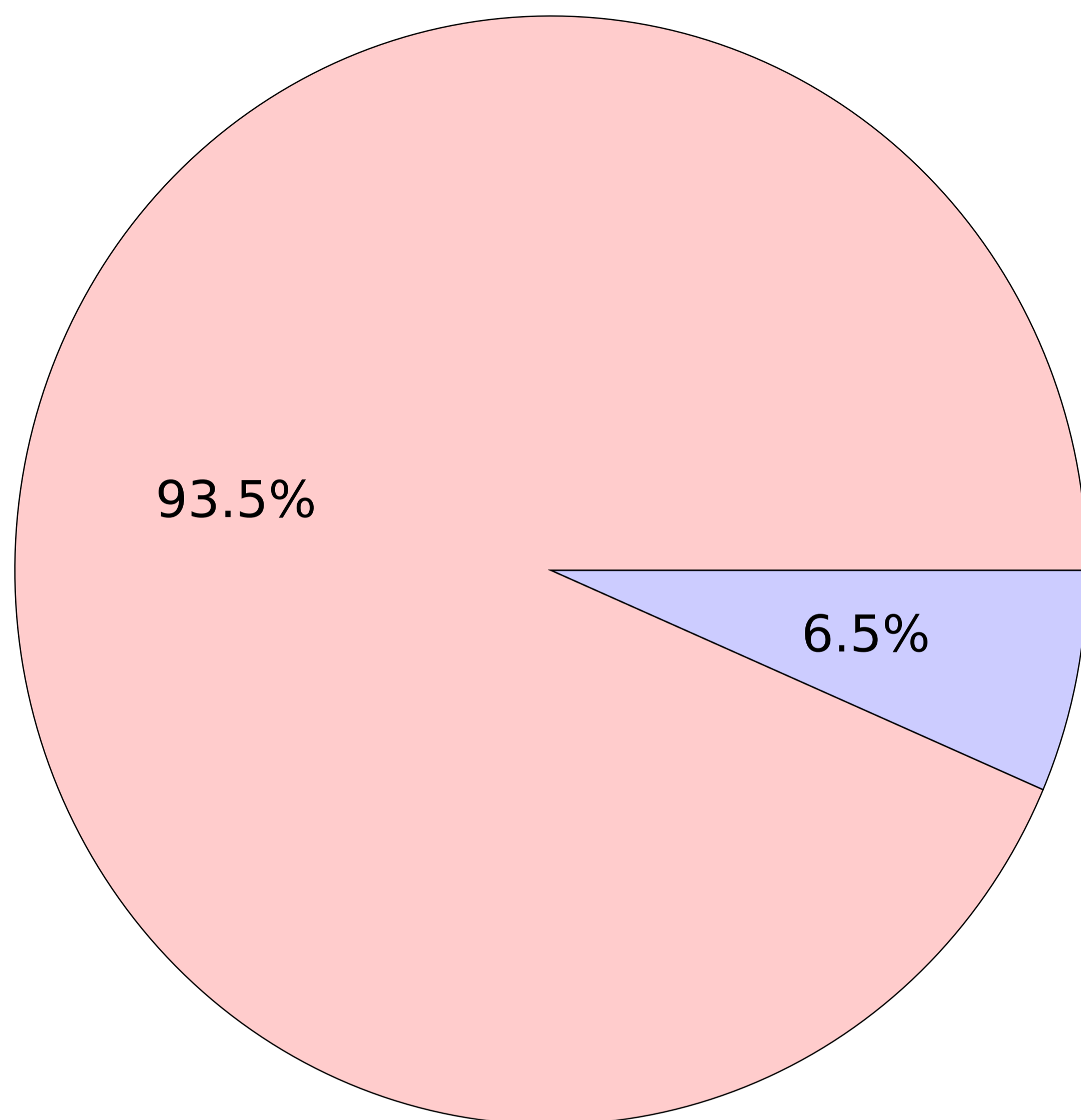

NHEJ  
(1496 reads)

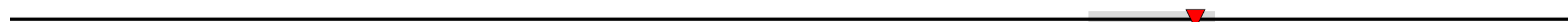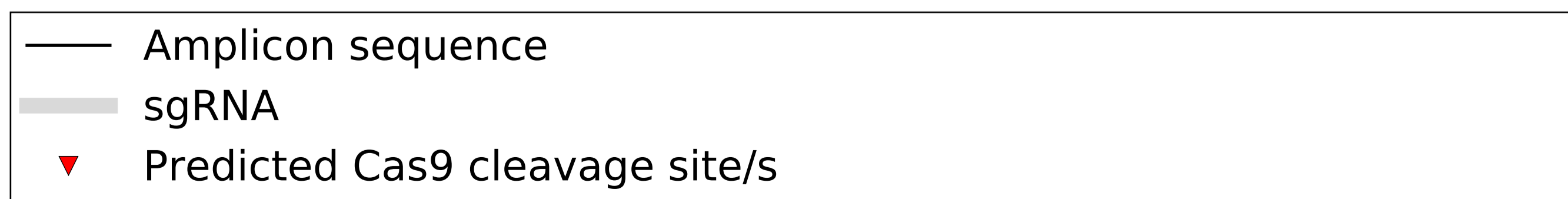

Supplement: Supplementary file 14 — Additional file 14. CRISPResso NHEJ pie charts. [file 12896_2019_565_MOESM14_ESM.zip › CRISPResso_EPSPS-7DS-gRNA4-rep2.pdf]

Unmodified  
(12967 reads)

100.0%

0.0%

NHEJ  
(1 reads)

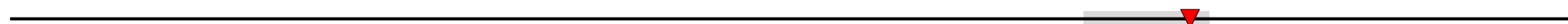

- Amplicon sequence
- sgRNA
- ▼ Predicted Cas9 cleavage site/s

Supplement: Supplementary file 14 — Additional file 14. CRISPResso NHEJ pie charts. [file 12896_2019_565_MOESM14_ESM.zip › CRISPResso_EPSPS-7DS-gRNA4-rep2-negative.pdf]

Unmodified  
(15540 reads)

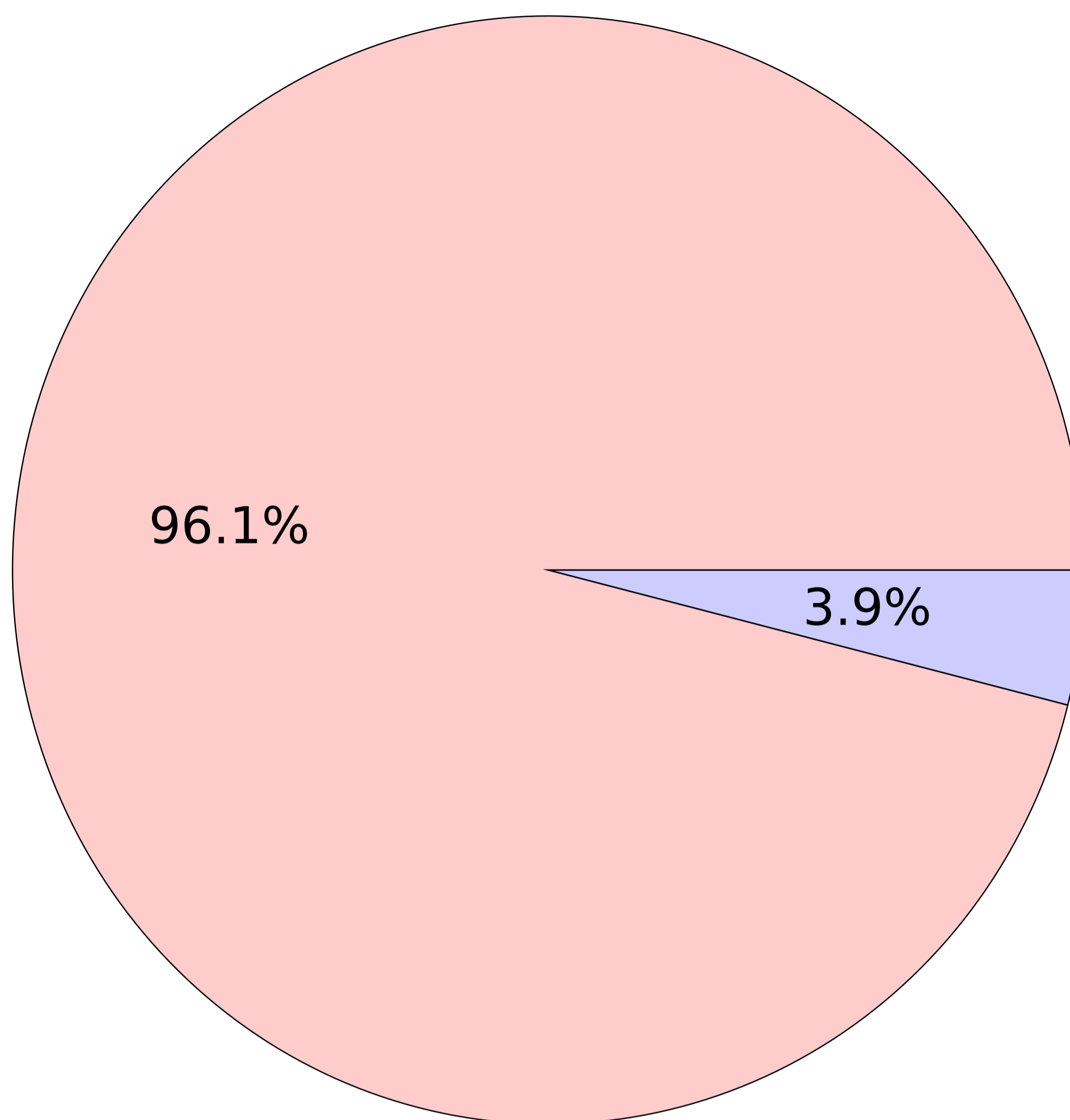

NHEJ  
(634 reads)

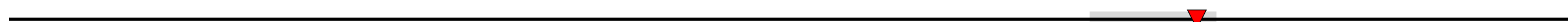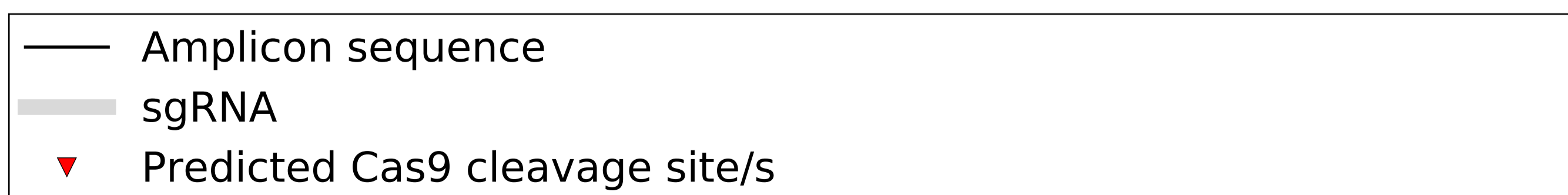

Supplement: Supplementary file 14 — Additional file 14. CRISPResso NHEJ pie charts. [file 12896_2019_565_MOESM14_ESM.zip › CRISPResso_EPSPS-7DS-gRNA4-rep3.pdf]

Unmodified  
(18922 reads)

100.0%

0.0%

NHEJ  
(1 reads)

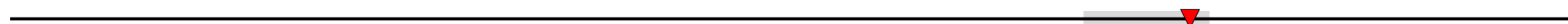

- Amplicon sequence
- sgRNA
- ▼ Predicted Cas9 cleavage site/s

Supplement: Supplementary file 14 — Additional file 14. CRISPResso NHEJ pie charts. [file 12896_2019_565_MOESM14_ESM.zip › CRISPResso_EPSPS-7DS-gRNA4-rep3-negative.pdf]

Unmodified  
(15478 reads)

80.5%

19.5%

NHEJ  
(3739 reads)

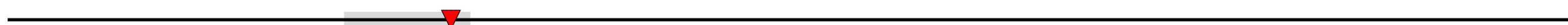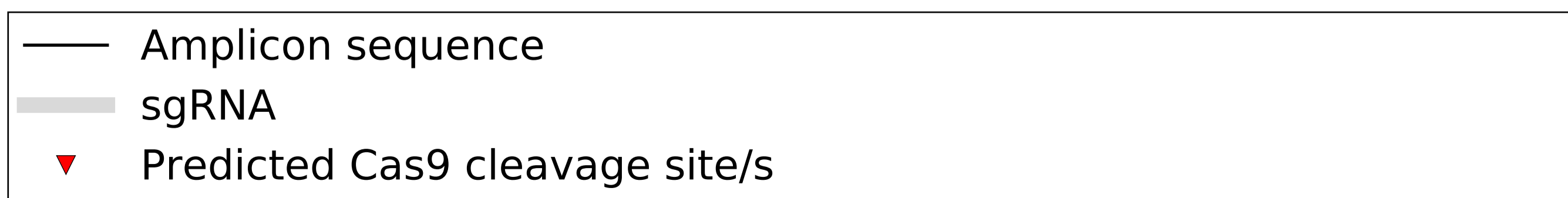

Supplement: Supplementary file 14 — Additional file 14. CRISPResso NHEJ pie charts. [file 12896_2019_565_MOESM14_ESM.zip › CRISPResso_EPSPS-7DS-gRNA5-rep1.pdf]

Unmodified  
(14979 reads)

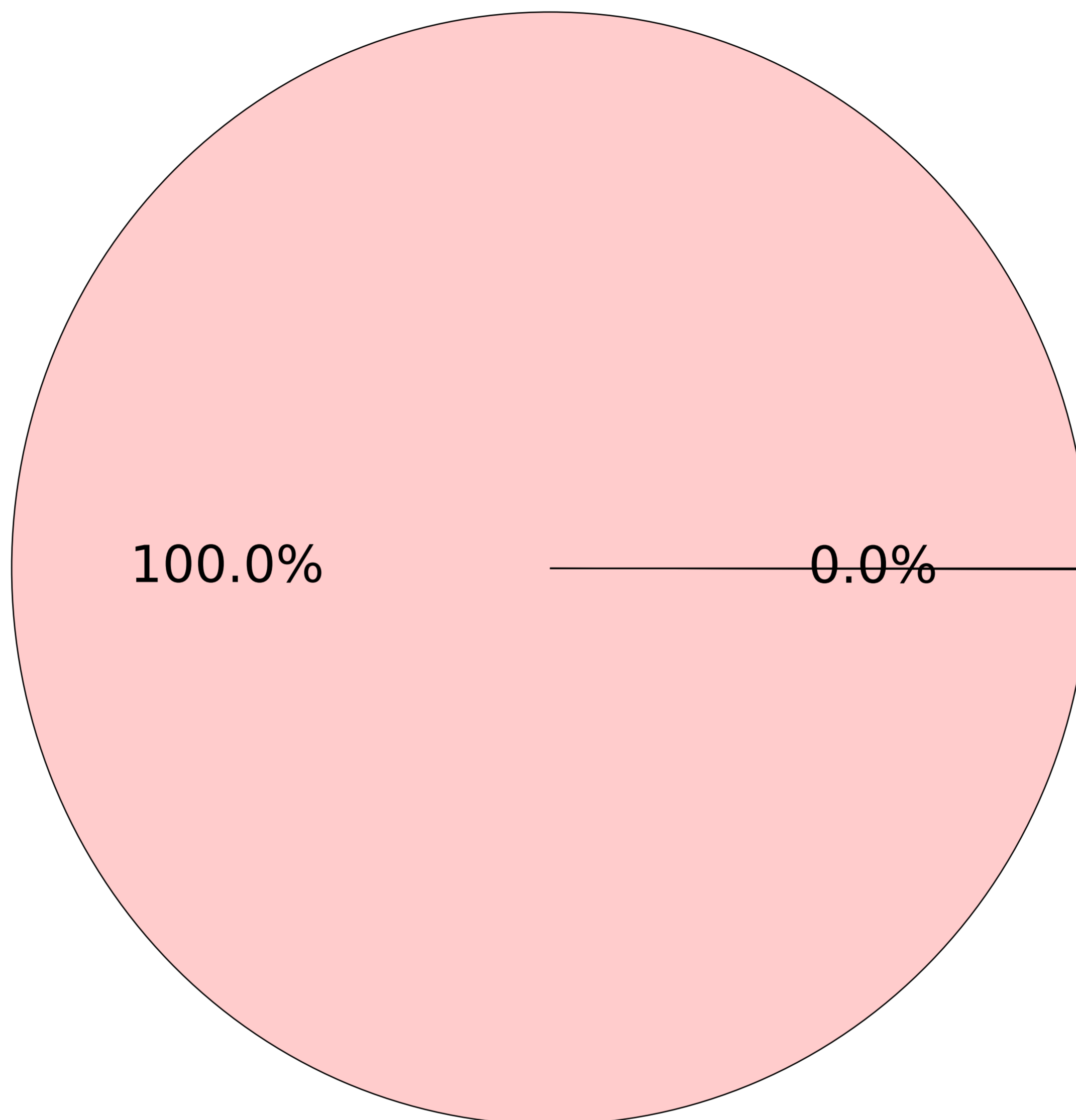

NHEJ  
(5 reads)

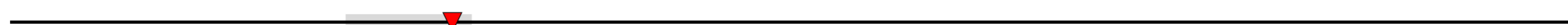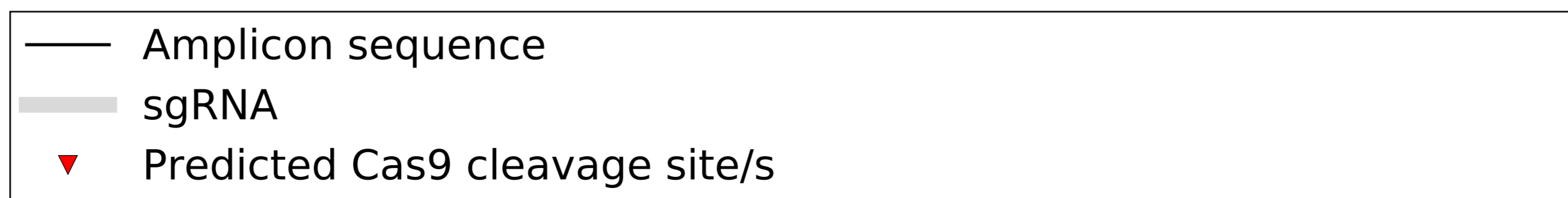

Supplement: Supplementary file 14 — Additional file 14. CRISPResso NHEJ pie charts. [file 12896_2019_565_MOESM14_ESM.zip › CRISPResso_EPSPS-7DS-gRNA5-rep1-negative.pdf]

Unmodified  
(12820 reads)

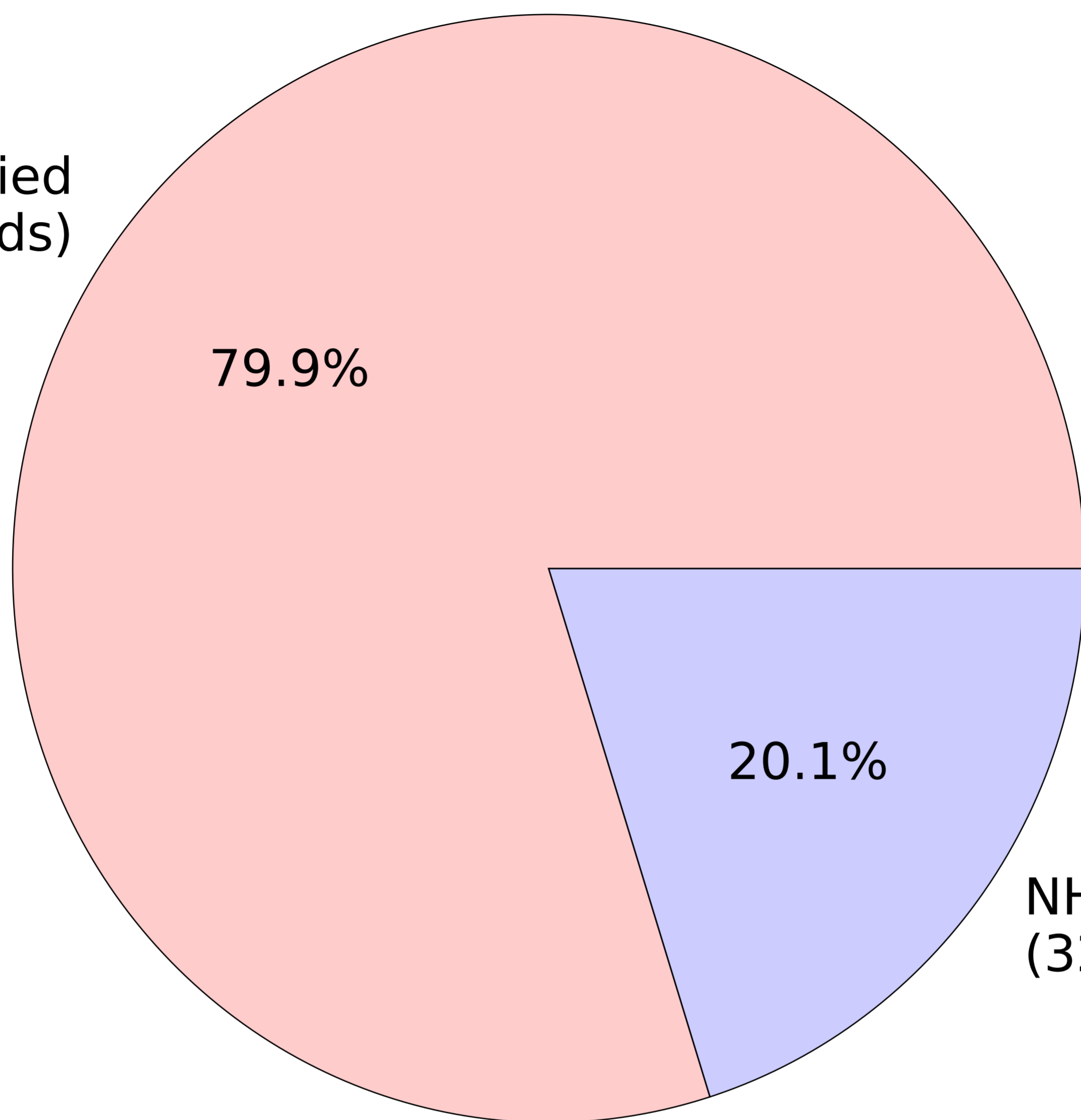

NHEJ  
(3232 reads)

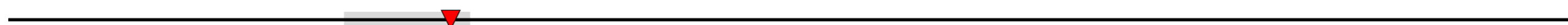

- Amplicon sequence
- sgRNA
- ▼ Predicted Cas9 cleavage site/s

Supplement: Supplementary file 14 — Additional file 14. CRISPResso NHEJ pie charts. [file 12896_2019_565_MOESM14_ESM.zip › CRISPResso_EPSPS-7DS-gRNA5-rep2.pdf]

Unmodified  
(12962 reads)

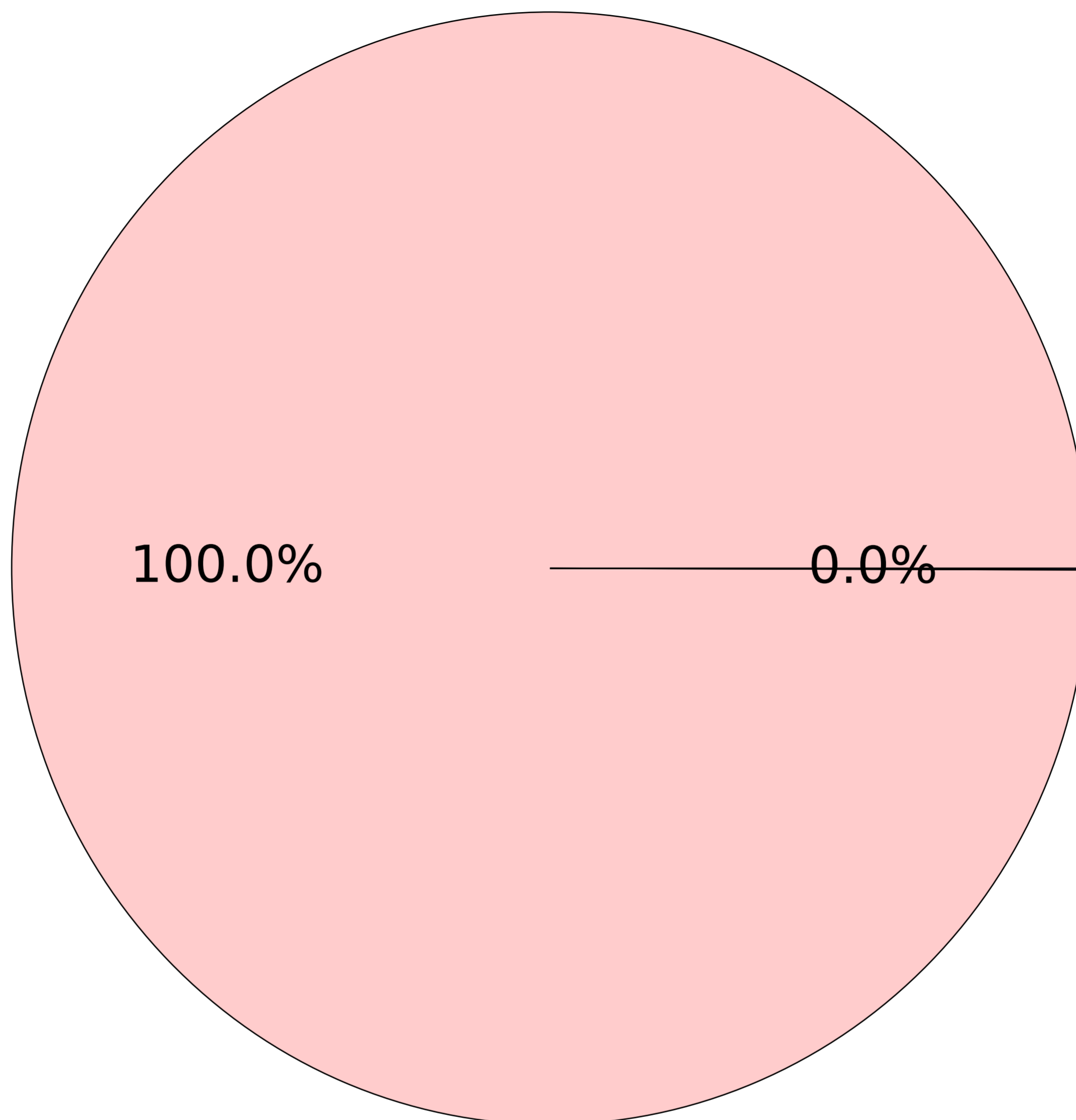

NHEJ  
(6 reads)

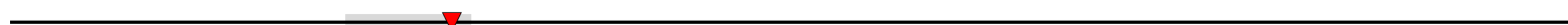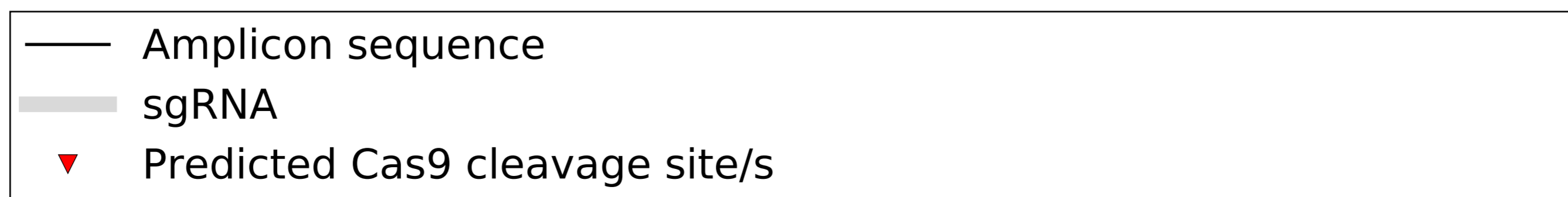

Supplement: Supplementary file 14 — Additional file 14. CRISPResso NHEJ pie charts. [file 12896_2019_565_MOESM14_ESM.zip › CRISPResso_EPSPS-7DS-gRNA5-rep2-negative.pdf]

Unmodified  
(10986 reads)

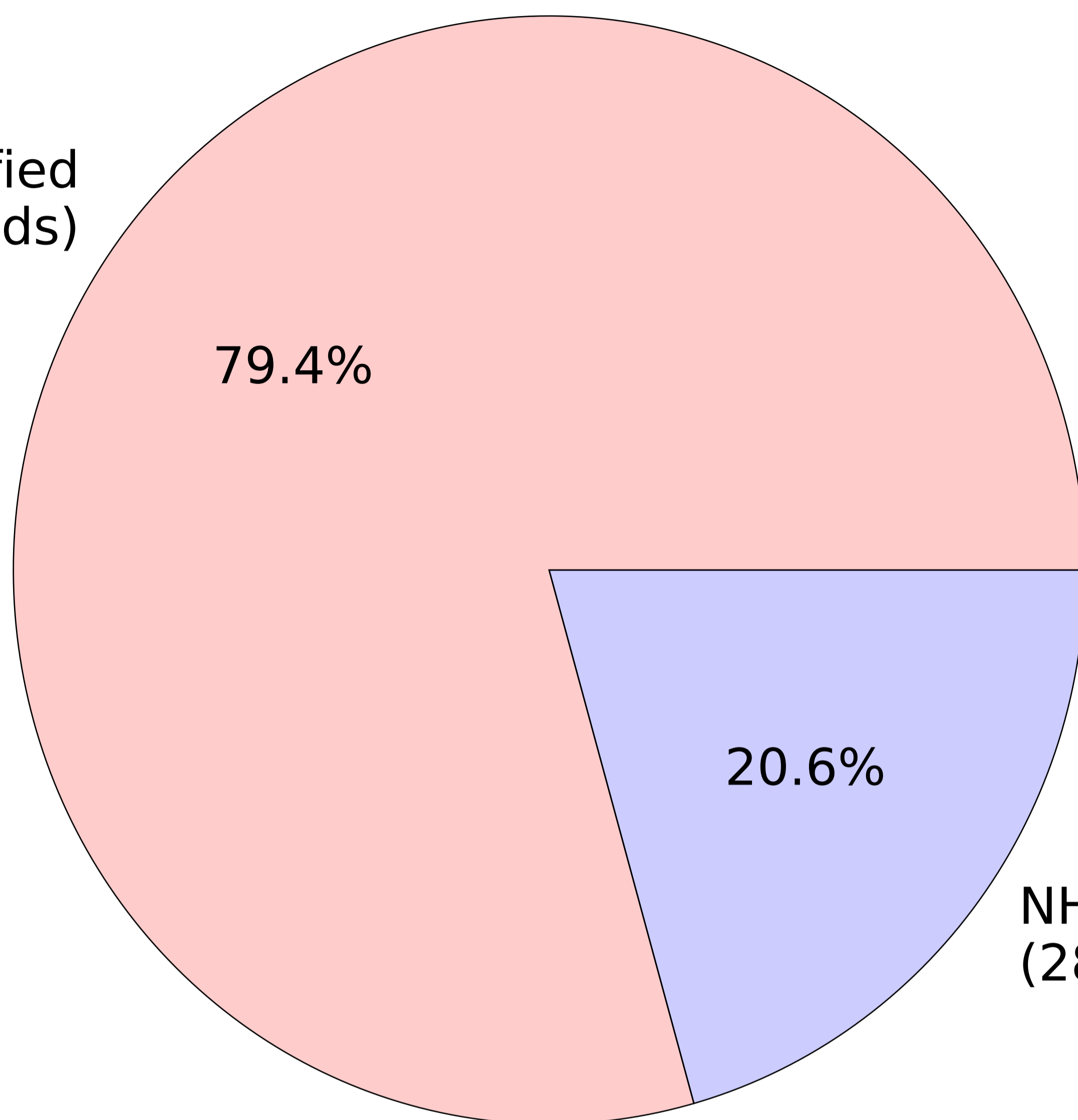

NHEJ  
(2858 reads)

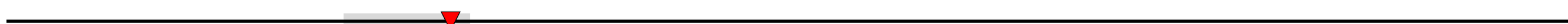

- Amplicon sequence
- sgRNA
- ▼ Predicted Cas9 cleavage site/s

Supplement: Supplementary file 14 — Additional file 14. CRISPResso NHEJ pie charts. [file 12896_2019_565_MOESM14_ESM.zip › CRISPResso_EPSPS-7DS-gRNA5-rep3.pdf]

Unmodified  
(18919 reads)

100.0%

0.0%

NHEJ  
(4 reads)

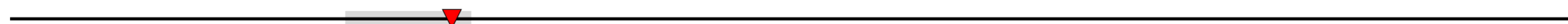

- Amplicon sequence
- sgRNA
- ▼ Predicted Cas9 cleavage site/s

Supplement: Supplementary file 14 — Additional file 14. CRISPResso NHEJ pie charts. [file 12896_2019_565_MOESM14_ESM.zip › CRISPResso_EPSPS-7DS-gRNA5-rep3-negative.pdf]

Unmodified  
(12569 reads)

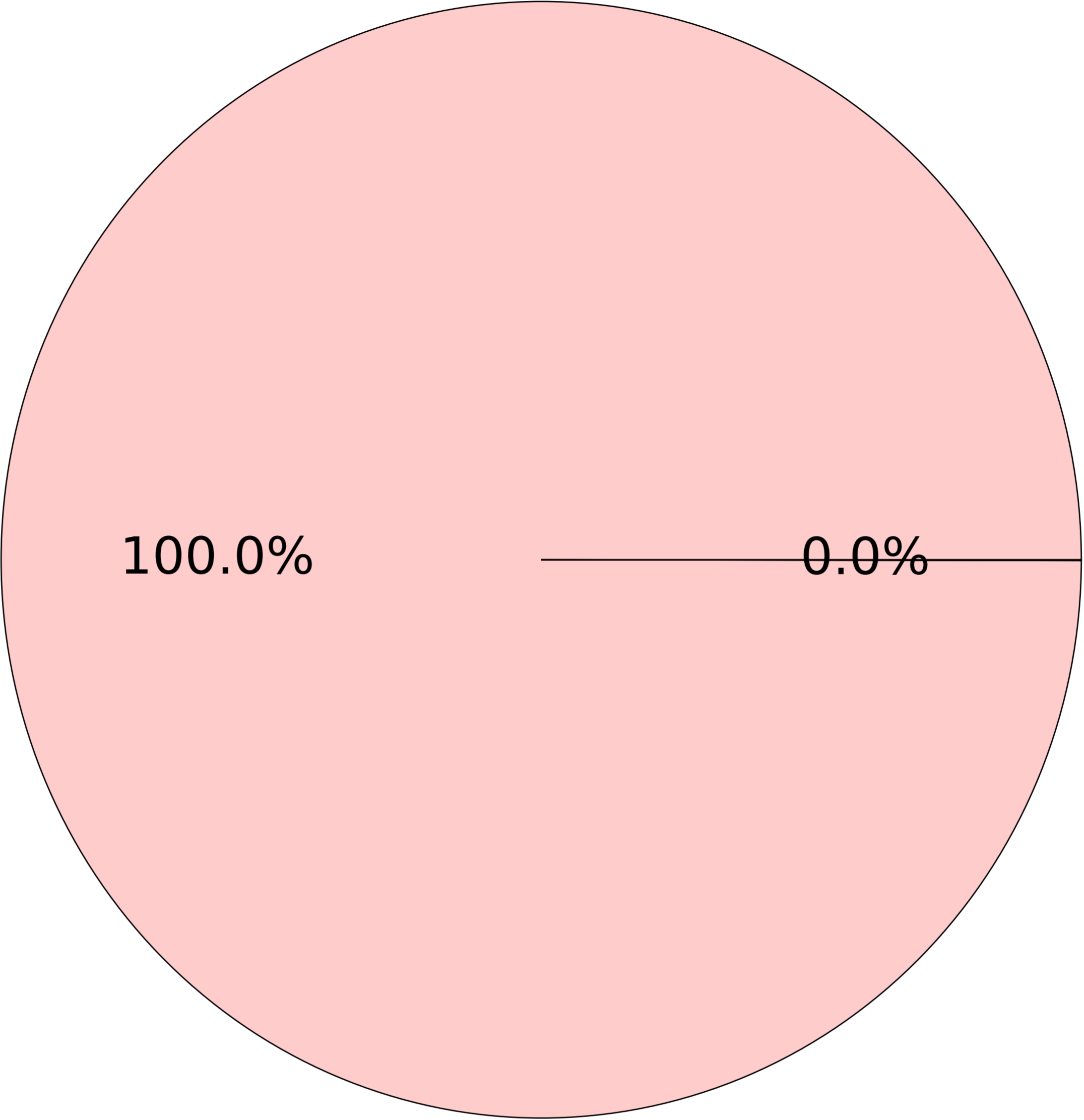

NHEJ  
(3 reads)

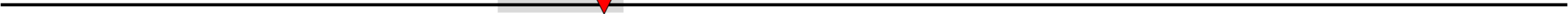

—

Amplicon sequence

—

sgRNA

▼

Predicted Cas9 cleavage site/s

Supplement: Supplementary file 14 — Additional file 14. CRISPResso NHEJ pie charts. [file 12896_2019_565_MOESM14_ESM.zip › CRISPResso_EPSPS-7DS-gRNA6-rep1.pdf]

Unmodified  
(14982 reads)

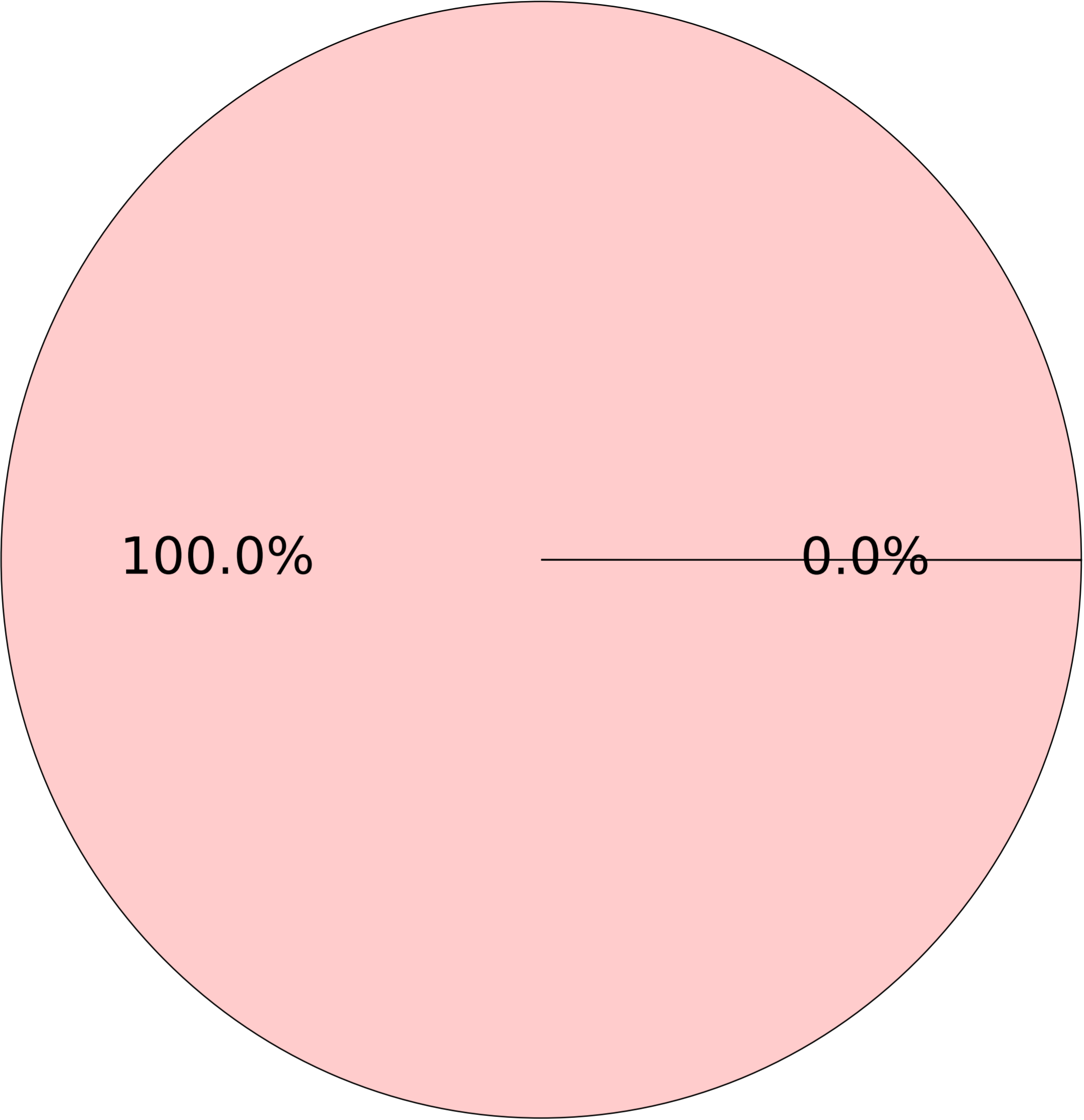

NHEJ  
(2 reads)

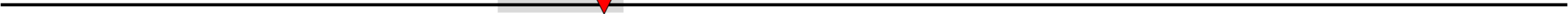

—

Amplicon sequence

—

sgRNA

▼

Predicted Cas9 cleavage site/s

Supplement: Supplementary file 14 — Additional file 14. CRISPResso NHEJ pie charts. [file 12896_2019_565_MOESM14_ESM.zip › CRISPResso_EPSPS-7DS-gRNA6-rep1-negative.pdf]

Unmodified  
(11371 reads)

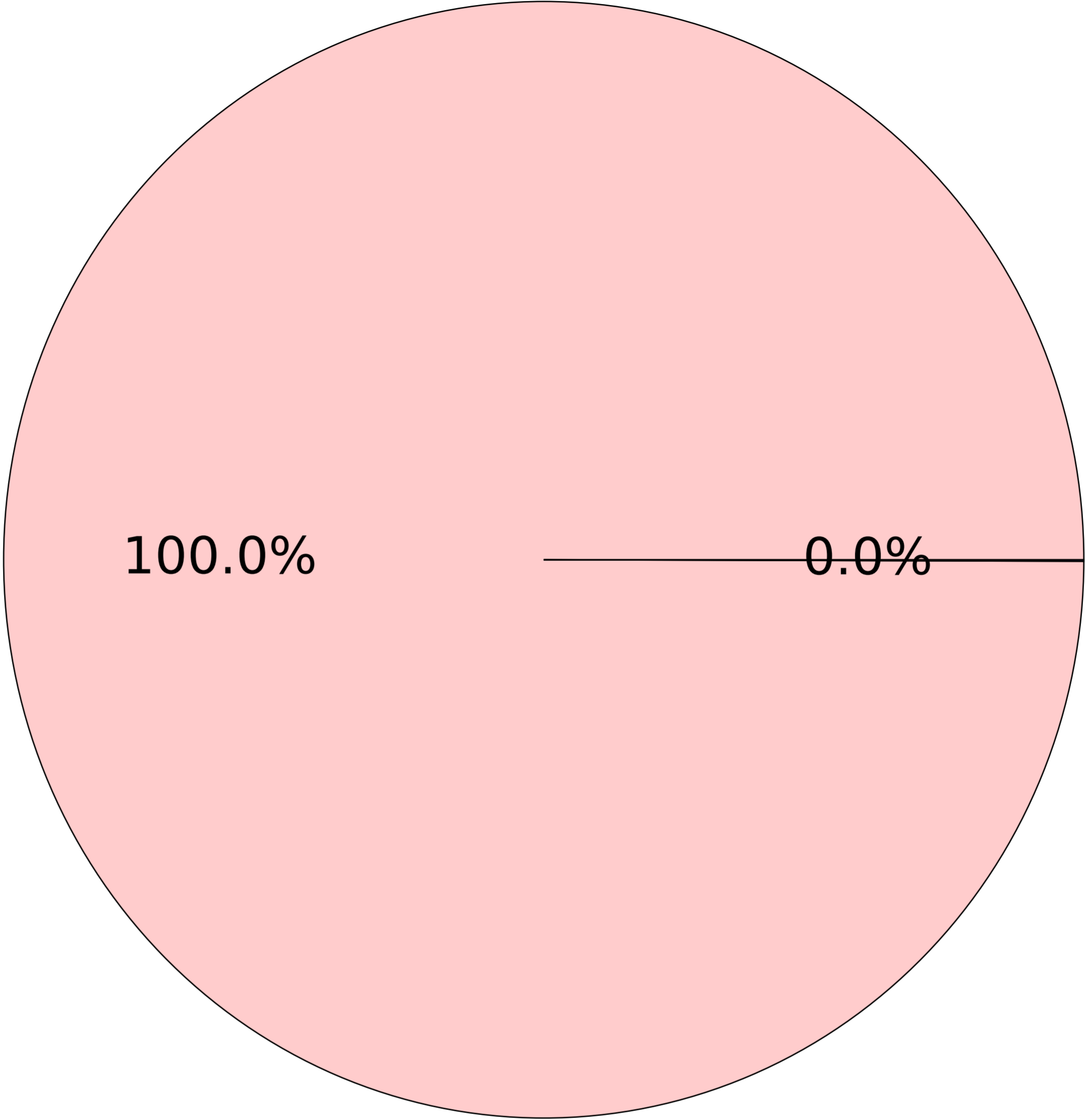

NHEJ  
(5 reads)

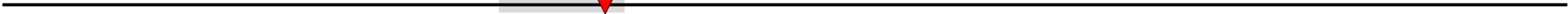

—

Amplicon sequence

—

sgRNA

▼

Predicted Cas9 cleavage site/s

Supplement: Supplementary file 14 — Additional file 14. CRISPResso NHEJ pie charts. [file 12896_2019_565_MOESM14_ESM.zip › CRISPResso_EPSPS-7DS-gRNA6-rep2.pdf]

Unmodified  
(12966 reads)

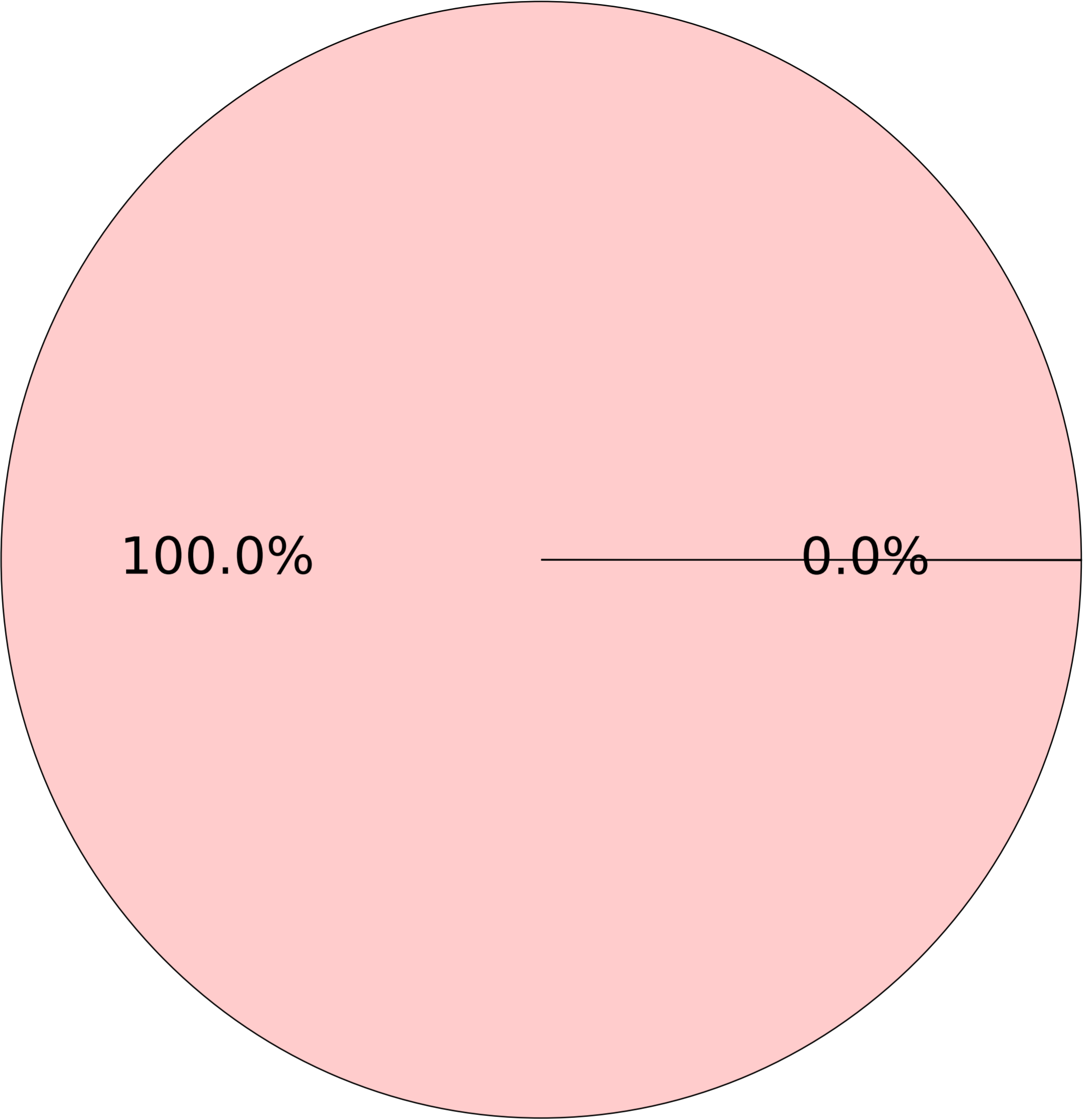

NHEJ  
(2 reads)

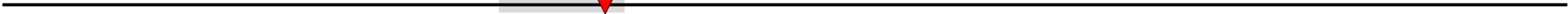

—

Amplicon sequence

—

sgRNA

▼

Predicted Cas9 cleavage site/s

Supplement: Supplementary file 14 — Additional file 14. CRISPResso NHEJ pie charts. [file 12896_2019_565_MOESM14_ESM.zip › CRISPResso_EPSPS-7DS-gRNA6-rep2-negative.pdf]

Unmodified  
(11411 reads)

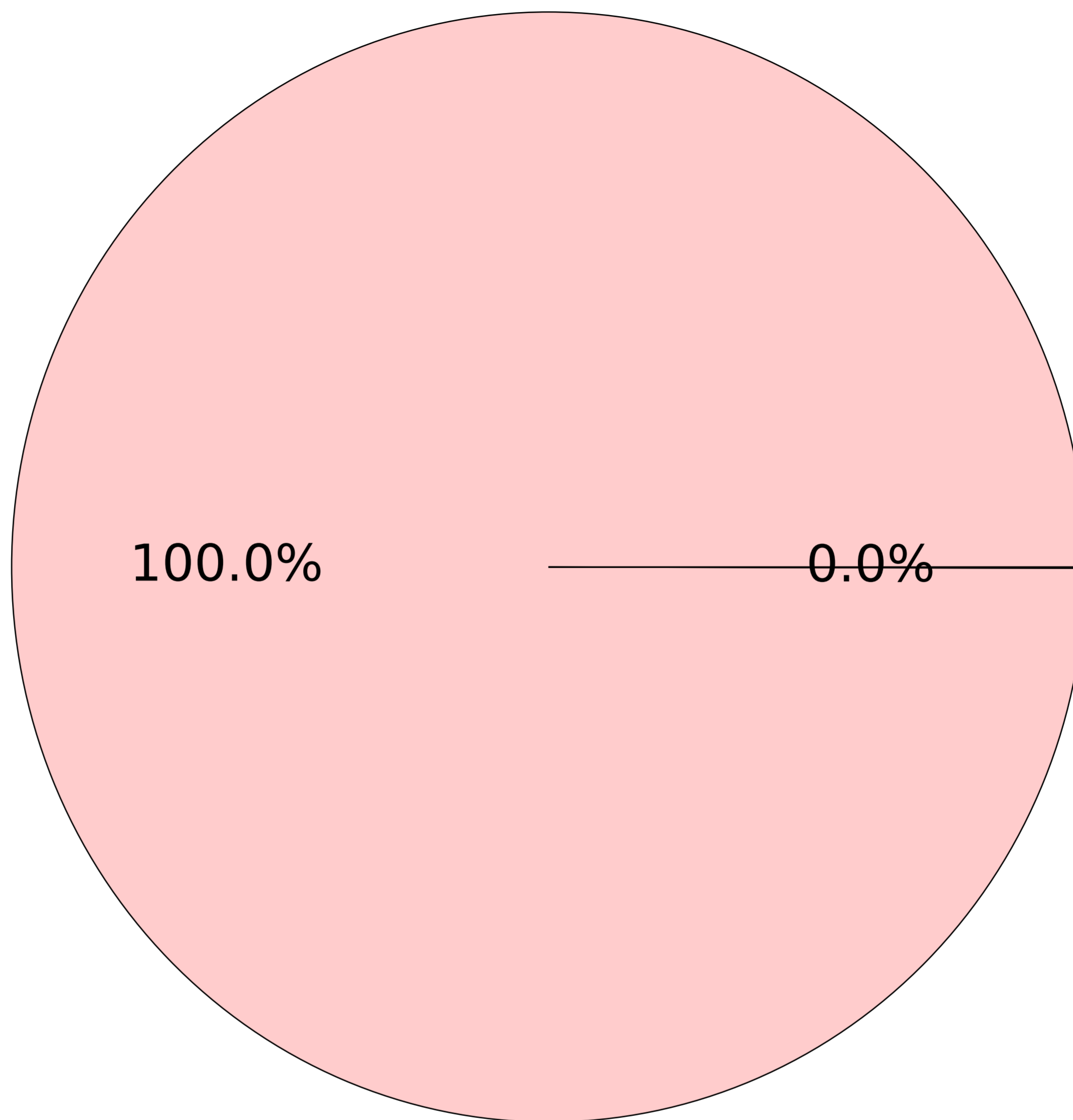

NHEJ  
(4 reads)

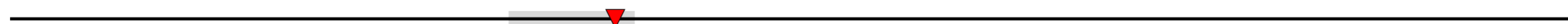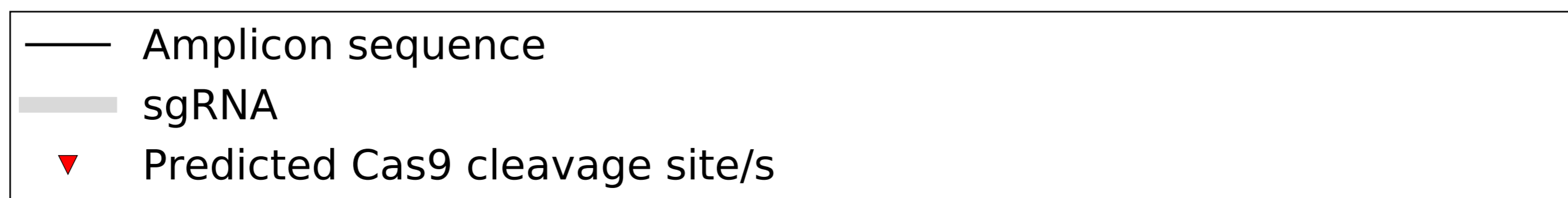

Supplement: Supplementary file 14 — Additional file 14. CRISPResso NHEJ pie charts. [file 12896_2019_565_MOESM14_ESM.zip › CRISPResso_EPSPS-7DS-gRNA6-rep3.pdf]

Unmodified  
(18916 reads)

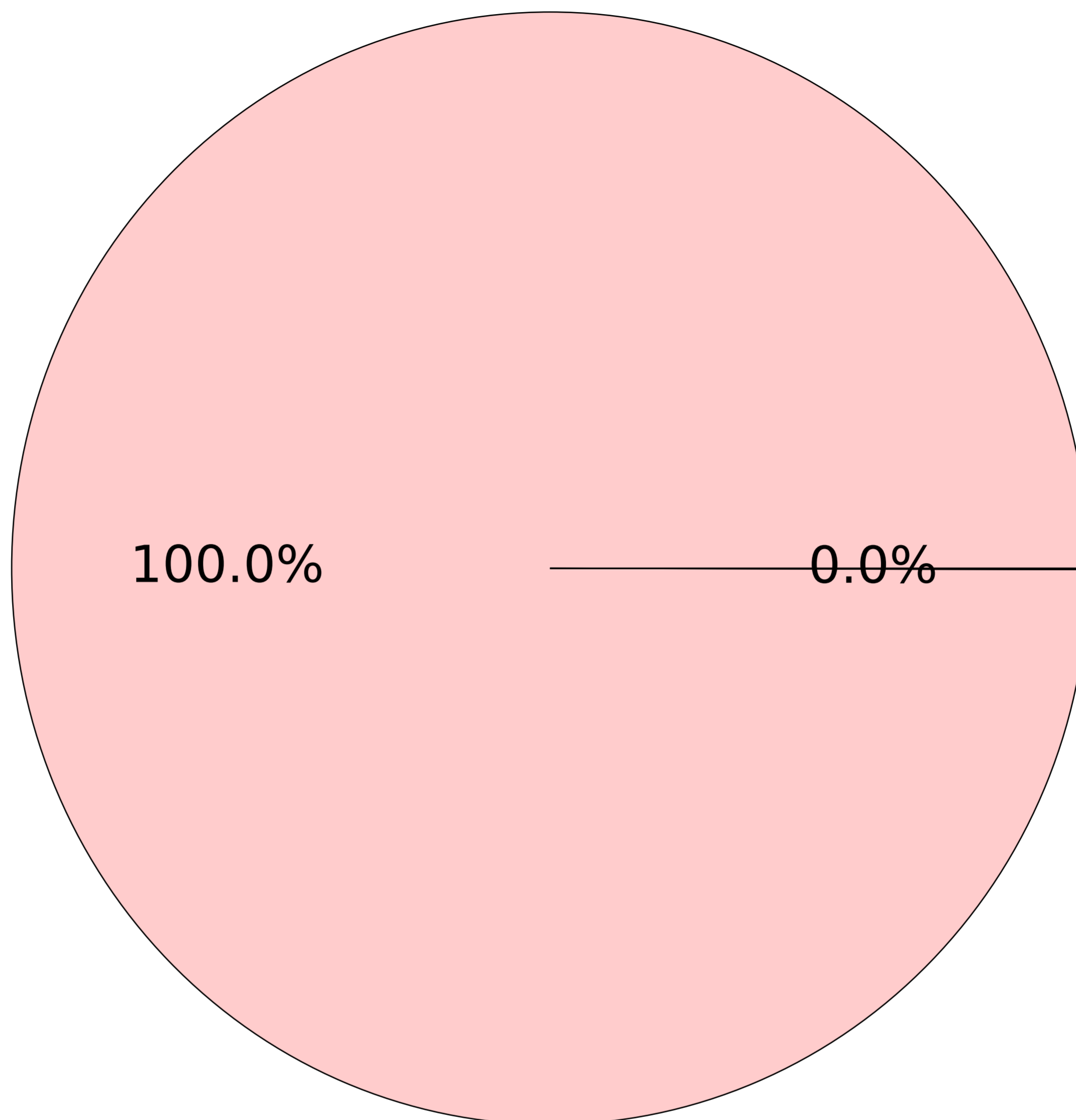

NHEJ  
(7 reads)

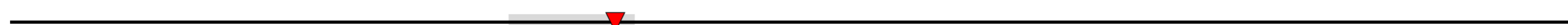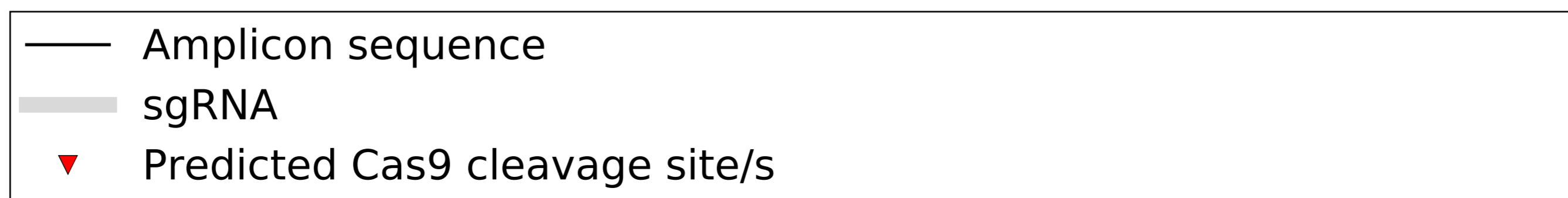

Supplement: Supplementary file 14 — Additional file 14. CRISPResso NHEJ pie charts. [file 12896_2019_565_MOESM14_ESM.zip › CRISPResso_EPSPS-7DS-gRNA6-rep3-negative.pdf]

Unmodified  
(20531 reads)

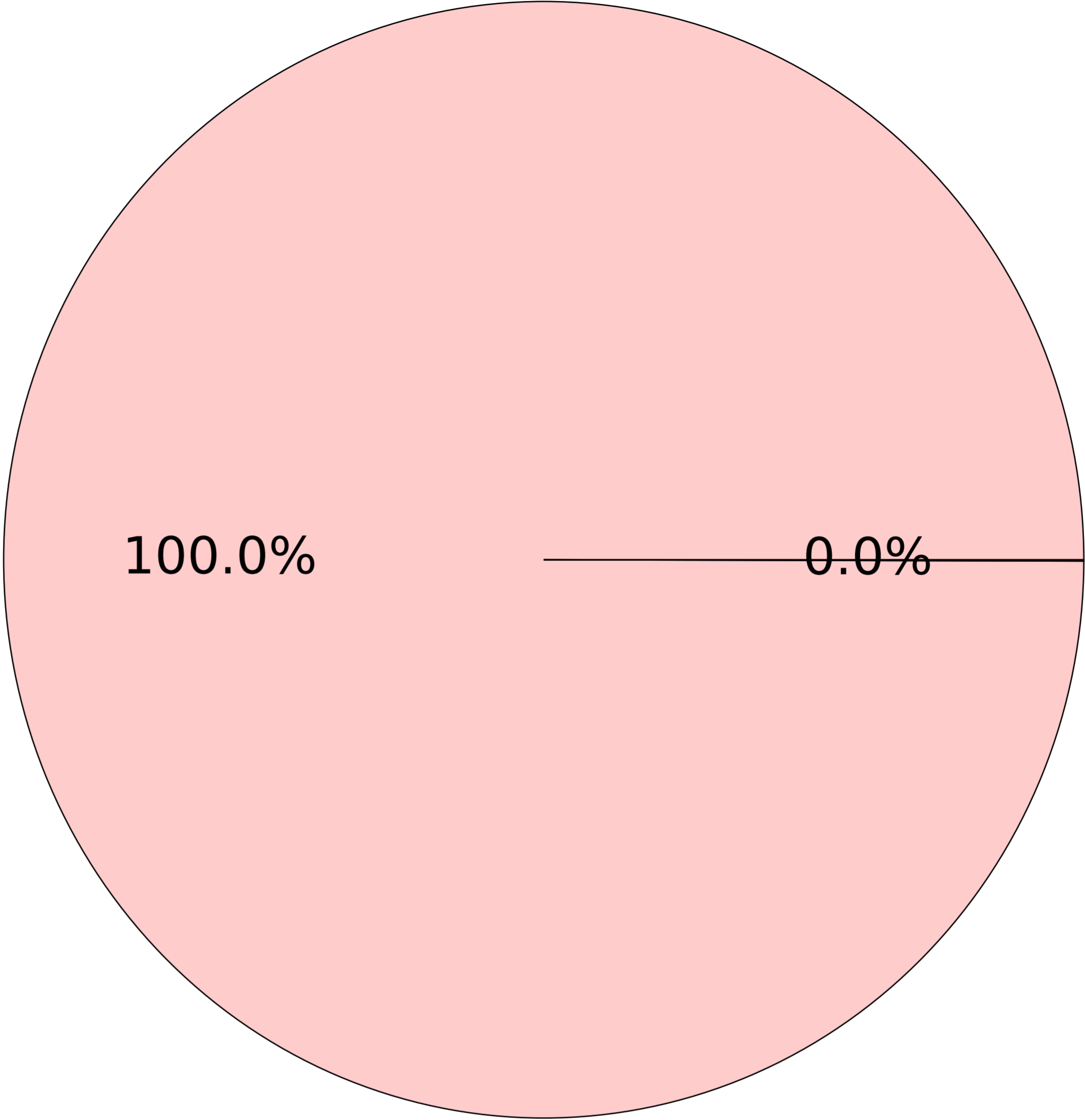

NHEJ  
(8 reads)

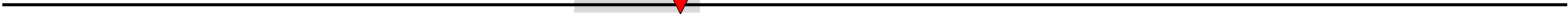

—

Amplicon sequence

—

sgRNA

▼

Predicted Cas9 cleavage site/s

Supplement: Supplementary file 14 — Additional file 14. CRISPResso NHEJ pie charts. [file 12896_2019_565_MOESM14_ESM.zip › CRISPResso_EPSPS-7DS-gRNA7-rep1.pdf]

Unmodified  
(14983 reads)

100.0%

0.0%

NHEJ  
(1 reads)

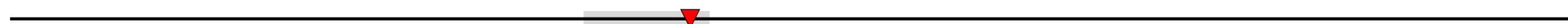

- Amplicon sequence
- sgRNA
- ▼ Predicted Cas9 cleavage site/s

Supplement: Supplementary file 14 — Additional file 14. CRISPResso NHEJ pie charts. [file 12896_2019_565_MOESM14_ESM.zip › CRISPResso_EPSPS-7DS-gRNA7-rep1-negative.pdf]

Unmodified  
(18459 reads)

100.0%

0.0%

NHEJ  
(3 reads)

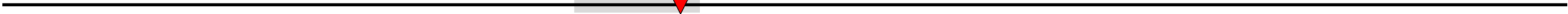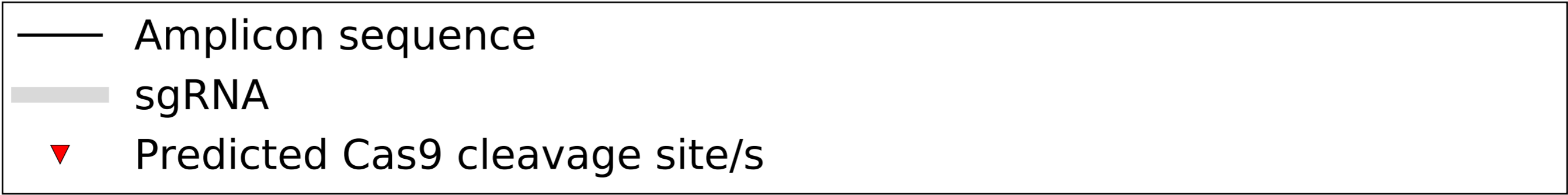

Supplement: Supplementary file 14 — Additional file 14. CRISPResso NHEJ pie charts. [file 12896_2019_565_MOESM14_ESM.zip › CRISPResso_EPSPS-7DS-gRNA7-rep2.pdf]

Unmodified  
(12966 reads)

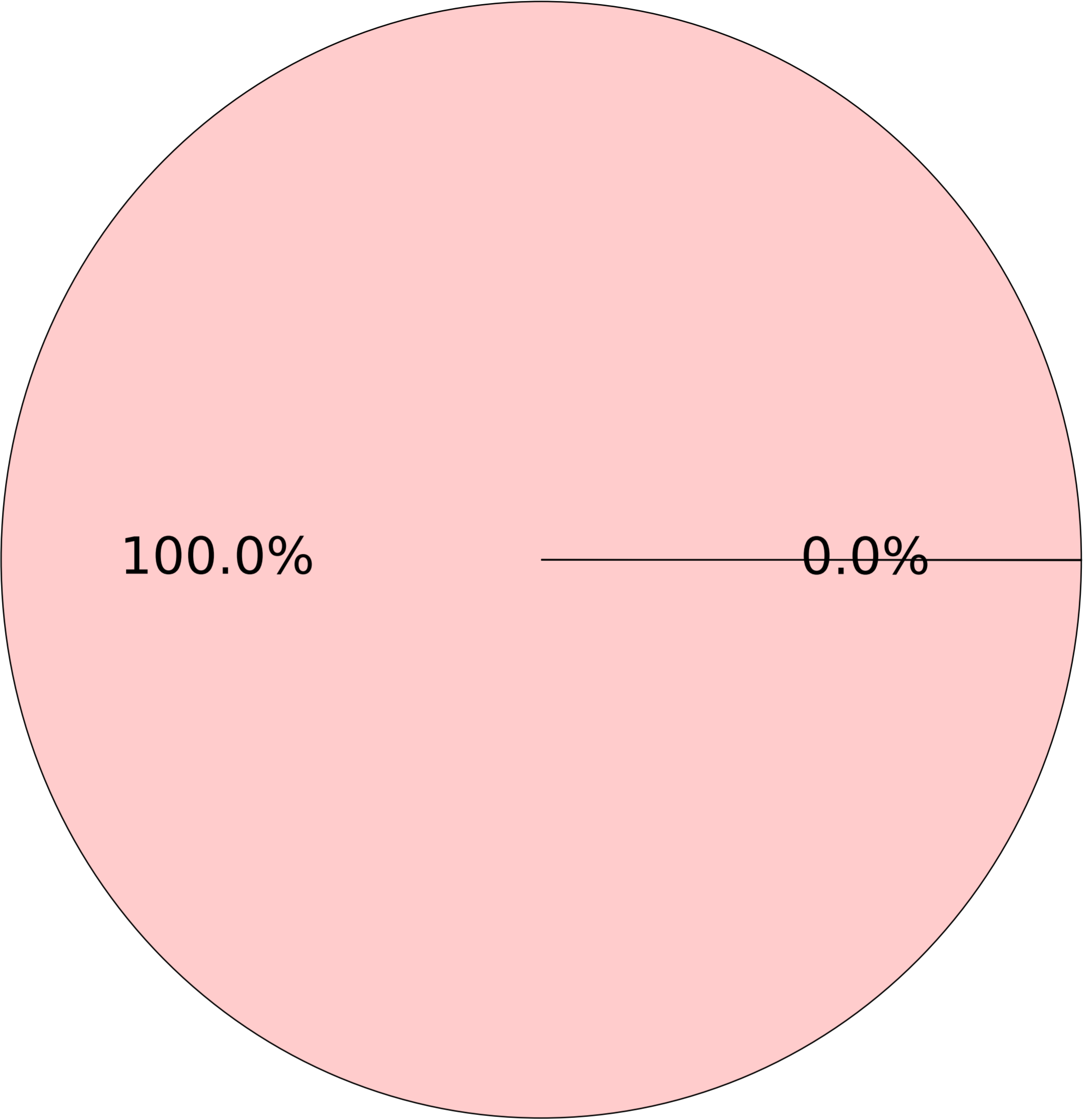

NHEJ  
(2 reads)

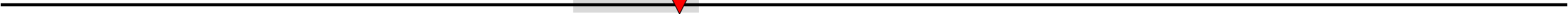

—

Amplicon sequence

—

sgRNA

▼

Predicted Cas9 cleavage site/s

Supplement: Supplementary file 14 — Additional file 14. CRISPResso NHEJ pie charts. [file 12896_2019_565_MOESM14_ESM.zip › CRISPResso_EPSPS-7DS-gRNA7-rep2-negative.pdf]

Unmodified  
(21044 reads)

100.0%

0.0%

NHEJ  
(3 reads)

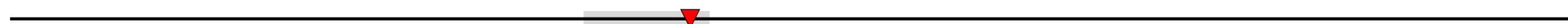

- Amplicon sequence
- sgRNA
- ▼ Predicted Cas9 cleavage site/s

Supplement: Supplementary file 14 — Additional file 14. CRISPResso NHEJ pie charts. [file 12896_2019_565_MOESM14_ESM.zip › CRISPResso_EPSPS-7DS-gRNA7-rep3.pdf]
