# Supplementary material for: gRNA validation for wheat genome editing with the CRISPR-Cas9 system
Source: BMC Biotechnol. 2019 Oct 30;19:71. doi: 10.1186/s12896-019-0565-z (PMC6829922; doi:10.1186/s12896-019-0565-z)
Supplement: Supplementary file 16 — Additional file 16. Primers and oligonucleotides used in this study. [file 12896_2019_565_MOESM16_ESM.pdf]

Additional file 16. Primers and oligonucleotides used in this study

Oligo name	Sequence 5'-3' (non-binding in red)	Use
TaEPSPS_Ex1_3'_F2	GCTCCAAGTCGCTCTCCAAC	Cloning of EPSPS on 7AS/4AL
TaEPSPS_Ex5_R2	TTCGACAGTCACAGTCCCTCC	Cloning of EPSPS on 7AS/4AL and Sanger seq of pCR8-TaEPSPS-FL clones
TaEPSPS_D_Int6_R	CGCACGTGCTAGATTGC	Cloning of EPSPS on 7DS
TaEPSPS_Ex2_F	CTGTGGTTGTTGGCTGTGG	Sanger seq of pCR8-TaEPSPS-FL clones
TaEPSPS_Ex3_R2	CGCTCCCTCATTCTTGGTAC	Sanger seq of pCR8-TaEPSPS-FL clones
TaEPSPS-gRNA1_F	CTTGAACTGCAATGCGGCCACTGA	Sense oligo for gRNA1
TaEPSPS-gRNA1_R	AAACTCAGTGGCCGCATTGCAGTT	Antisense oligo for gRNA1
TaEPSPS-gRNA2_F	CTTGCTACTACAGCTGCCGTCAG	Sense oligo for gRNA2
TaEPSPS-gRNA2_R	AAACCTGACGGCAGCTGTAGTAG	Antisense oligo for gRNA2
TaEPSPS-gRNA3_F	CTTGATCAGTCTCCAAACATACCCA	Sense oligo for gRNA3
TaEPSPS-gRNA3_R	AAACTGGGTATGTTTGGAGACTGAT	Antisense oligo for gRNA3
TaEPSPS-gRNA4_F	CTTGAAATAAGTATGAGATCCAT	Sense oligo for gRNA4
TaEPSPS-gRNA4_R	AAACATGGATCTCATACTTATTT	Antisense oligo for gRNA4
TaEPSPS-gRNA5_F	CTTGGTCGAAAAGGACGCCAAAG	Sense oligo for gRNA5
TaEPSPS-gRNA5_R	AAACCTTTGGCGTCCTTTTCGAC	Antisense oligo for gRNA5
TaEPSPS-gRNA6_F	CTTGAAGCTCTTCTTGGGTAATGC	Sense oligo for gRNA6
TaEPSPS-gRNA6_R	AAACGCATTACCCAAGAAGAGCTT	Antisense oligo for gRNA6
TaEPSPS-gRNA7_F	CTTGTAATGCTGGAACTGCAATG	Sense oligo for gRNA7
TaEPSPS-gRNA7_R	AAACCATTGCAGTTCCAGCATTA	Antisense oligo for gRNA7
TaEPSPS_A_Int1_F	AGCTTGTATTTGGATCAAGATATC	Amplification of EPSPS on 7AS
TaEPSPS_A_Int4_R	TCAGTAAAAGCGTAGATGTGAC	Amplification of EPSPS on 7AS
TaEPSPS_B_Int1_F	TTCTGATGGACCCTTTATGAG	Amplification of EPSPS on 4AL
TaEPSPS_B_Int4_R	CAAACAACTGCCGATGTT	Amplification of EPSPS on 4AL
TaEPSPS_D_Int1_F	CATTGGATTGTGTAGCTTGTAC	Amplification/cloning of EPSPS on 7DS
TaEPSPS_D_Int4_R	GCCAAACAACTGCCGATATC	Amplification of EPSPS on 7DS
TaEPSPS_A_Int1_F2	GATCCGTAGCTTGTTTCTGG	Sanger seq of amplicons from 7AS
TaEPSPS_A_Int3_R	ACAGAAGTGTGCACAAAGTAG	Sanger seq of amplicons from 7AS
TaEPSPS_B_Int1_F2	TCACTGTCATATCTAGCTGATCT	Sanger seq of amplicons from 4AL
TaEPSPS_B_Int3_R	GTACATAACATGCAATTTCACG	Sanger seq of amplicons from 4AL
TaEPSPS_D_Int1_F2	TAGCTGATCCGTAGCTTGTC	Sanger seq of amplicons from 7DS
TaEPSPS_D_Int3_R	GTACATAACATGCAAGTTCACA	Sanger seq of amplicons from 7DS
TaEPSPS_NGS_F_P5	TCGTCGGCAGCGTCAGATGTGTATAAGAGACAGGTGGAAGCAGATAAAGTTGC	First round of amplification for deep seq
TaEPSPS_NGS_R_P7	GTCTCGTGGGCTCGGAGATGTGTATAAGAGACAGCCATCAAGCACATAACTGAAG	First round of amplification for deep seq
